# Supplementary material for: Introduction of the difluoromethyl group at the meta- or para-position of pyridines through regioselectivity switch
Source: Nat Commun. 2024 May 15;15:4121. doi: 10.1038/s41467-024-48383-1 (PMC11096164; doi:10.1038/s41467-024-48383-1)
Supplement: Supplementary file 1 — Supplementary Information [file 41467_2024_48383_MOESM1_ESM.pdf]

Supplementary Information

Introduction of the difluoromethyl group at the meta- or para-position of pyridines through regioselectivity switch

Pengwei Xu[+], Zhe Wang[+], Shu-Min Guo and Armido Studer*

Organisch-Chemisches Institut
Universität Münster, Münster, Germany

Correspondence to: studer@uni-muenster.de

Table of Contents

Section		
1	Materials and methods	3
2	Synthetic method for preparation of 2-difluoro-2-iodo-1-phenylethan-1-one (**1**)	4
3	Synthesis and analytical data for oxazino pyridines	5
4	Condition optimization for the *meta*-difluoromethylation	26
5	Proposed mechanism	28
6	General procedure A and analytical data for *meta*-difluoromethylation	29
7	General procedure B and analytical data for *para*-difluoromethylation	45
8	Analytical data for late-stage difluoromethylation	52
9	Synthetic applications	55
10	NMR spectra	59
11	Supplementary References	184

Section 1. Materials and methods

Unless stated otherwise, all reactions were run under argon atmosphere. Acetonitrile (MeCN, 99.9%, Extra Dry over Molecular Sieves) and acetone (99.9%, Extra Dry over Molecular Sieves) were purchased from Acros Organics and used as received. Tetrahydrofuran (THF) was refluxed over Na and distilled from K afterwards. Otherwise noted, other commercially available reagents were purchased from ABCR, Acros Organics, Alfa Aesar, BLD pharma, Sigma Aldrich, Fluka, Fluorochem, TCI and were used as received. Thin layer chromatography (TLC) was run on Merck silica gel 60 F254 plates using UV light (254/366 nm) for detection. Flash chromatography (FC) was performed on Merck silica gel 60 (40-63 μm). Photoreactions were performed with a Kessil PR160L 465 nm lamp (40 W).

^{1}H NMR, ^{13}C NMR, and ^{19}F NMR spectra were recorded on a Bruker Avance II 300, Bruker Neo 400, Agilent DD2 500, and Agilent DD2 600 spectrometer (300/400/500/599 MHz for ^{1}H, 75/100/125/151 MHz for ^{13}C, 282/376/470/564 MHz for ^{19}F). Chemical shifts are reported as parts per million (ppm) with residual solvent signals as internal standard ($CHCl_3$, δ = 7.26 ppm for ^{1}H NMR, δ = 77.00 ppm for ^{13}C NMR). Data for ^{1}H NMR were presented as following: chemical shifts (δ, ppm), multiplicity (br = broad, s = singlet, d = doublet, t = triplet, q = quartet, dd = doublet of doublets, tt = triplet of triplets, td = triplet of doublets, m = multiplet), coupling constant (Hz), and integration. High-resolution EI mass spectra were recorded on a Waters-Micromass QuattroMicro GC-MS. High-resolution ESI-MS measurements were performed on a Bruker MicroTof. Accurate masses from high-resolution mass spectra were reported for the molecular ion $[M]^+$, $[M+H]^+$, $[M+Na]^+$. Melting points were determined with a Büchi Melting Point M-560 and are uncorrected.

Section 2. Synthetic method for preparation of 2-difluoro-2-iodo-1-phenylethan-1-one (1)

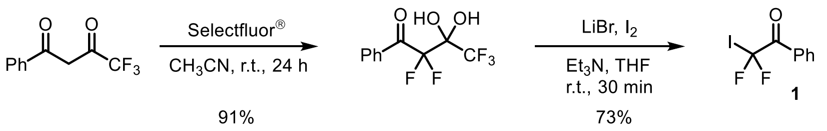

2-Difluoro-2-iodo-1-phenylethan-1-one (**1**) was prepared from 4,4,4-trifluoro-1-phenylbutane-1,3-dione with modified Colby's method[1,2]:

A solution of 4,4,4-trifluoro-1-phenylbutane-1,3-dione (14.0 g, 65 mmol) in CH_3CN (250 mL) was treated with Selectfluor® (65.2 g, 184 mmol) at room temperature. After 24 h, the reaction was diluted with EtOAc (50 ml) and filtered through Celite. The residue was concentrated in vacuo, dissolved in CH_2Cl_2 (150 mL), and washed with water (100 mL x 3). The combined organics were dried over with Na_2SO_4 and concentrated under reduced pressure to give 2,2,4,4,4-pentafluoro-3,3-dihydroxy-1-phenylbutan-1-one (16.0) as an oil in 91% yield.

To a solution of 2,2,4,4,4-pentafluoro-3,3-dihydroxy-1-phenylbutan-1-one (3.4 g, 12.5 mmol) in dry THF (120 mL) LiBr (7.0 g, 80 mmol) and I_2 (6.6 g, 26 mmol) were added sequentially. The reaction mixture was stirred for 1 min, and then Et_3N (3.5 mL, 25 mmol) was added. After stirring for 30 min at r.t., the reaction mixture was quenched with saturated aqueous $Na_2S_2O_3$ (50 mL). The mixture was diluted with water (150 mL) and extracted in EtOAc (50 mL x 3), and the combined organic phase was dried over Na_2SO_4 and concentrated under reduced pressure. Flash chromatography (pentane:EtOAc 25:1) afforded the 2-difluoro-2-iodo-1-phenylethan-1-one (**1**) as a pale purple liquid (2.55 g) in 73% yield.

2-Difluoro-2-iodo-1-phenylethan-1-one (**1**) is a known compound, the NMR data are in agreement with those reported.[3]

^{1}H NMR (300 MHz, $CDCl_3$) δ 8.17 (dd, J = 7.4, 1.1 Hz, 2H), 7.68 (t, J = 7.4 Hz, 1H), 7.53 (t, J = 7.7 Hz, 1H).

^{19}F NMR (282 MHz, $CDCl_3$) δ -54.21.

Section 3. Synthesis and analytical data for oxazino pyridines

Oxazino pyridines **3**, **S8**, **S13**, **6**, **53**, **S18-22**, **S32**, **S40**, **S48, S50-52** were synthesized according to our previous work.[4,5] The analytical data of the known substrates are in agreement with those reported in our previous work.[4,5]

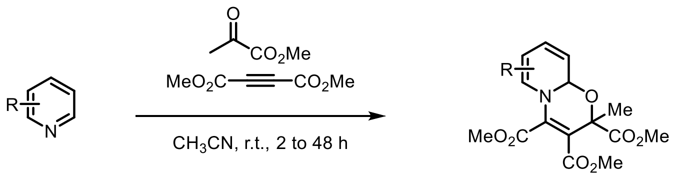

The oxazino pyridines were synthesized according to our previous work (**GP1**): To a 25 mL round-bottom flask with a magnetic stirring bar the corresponding pyridine (3 mmol, 1 equiv.), methyl pyruvate (616 mg, 6 mmol, 2 equiv.), and acetonitrile (6 mL, 0.5 M) were added under air atmosphere. Dimethyl acetylenedicarboxylate (852 mg, 6 mmol, 2 equiv.) was then added dropwise to the stirred reaction mixture. The reaction mixture was allowed to stir at room temperature for 2 to 48 h. After the reaction was complete, as monitored by TLC, the solvent was removed with a rotary evaporator under reduced pressure and the residue was submitted to flash column chromatography to give the corresponding oxazino pyridine.

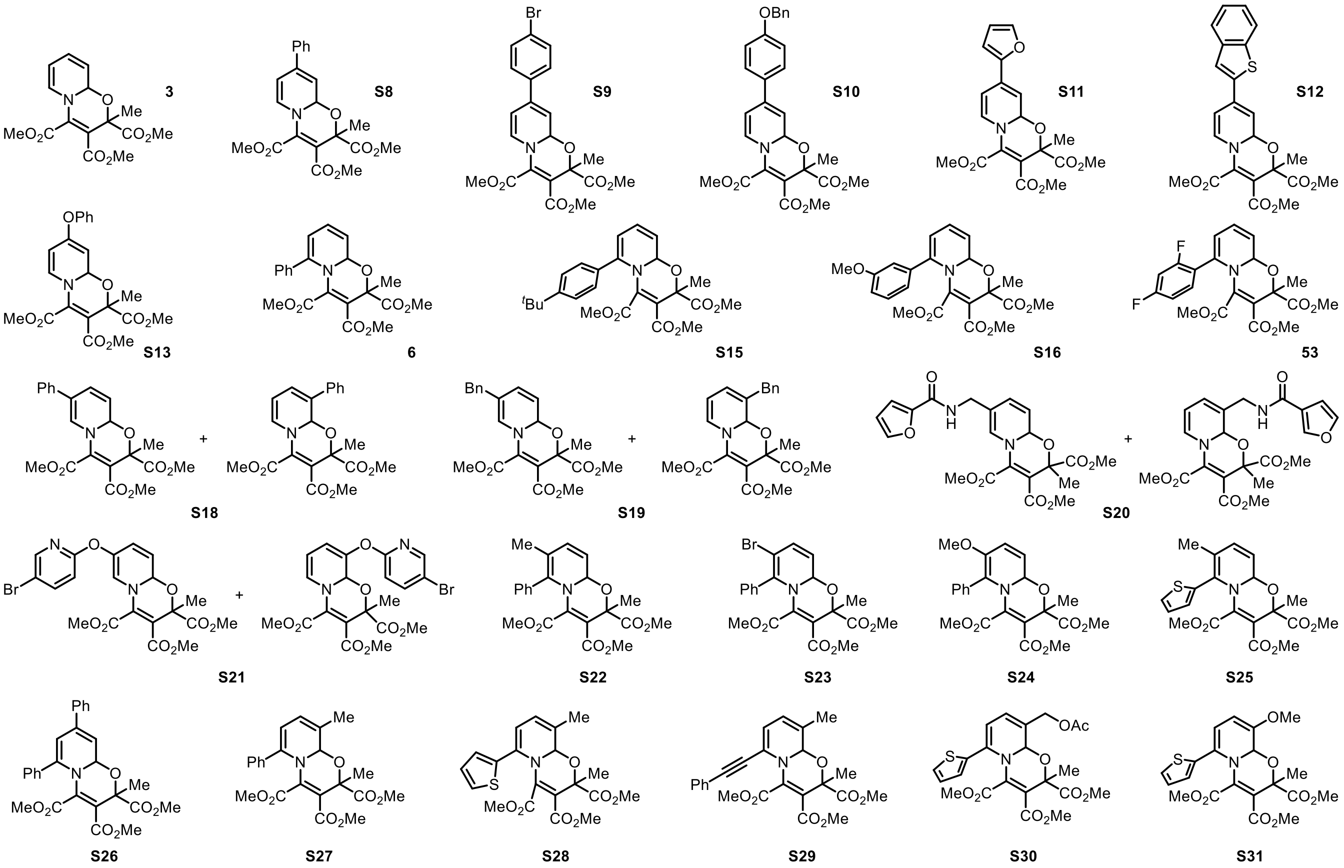

Supplementary Figure 1. Substrates used in this study (part 1).

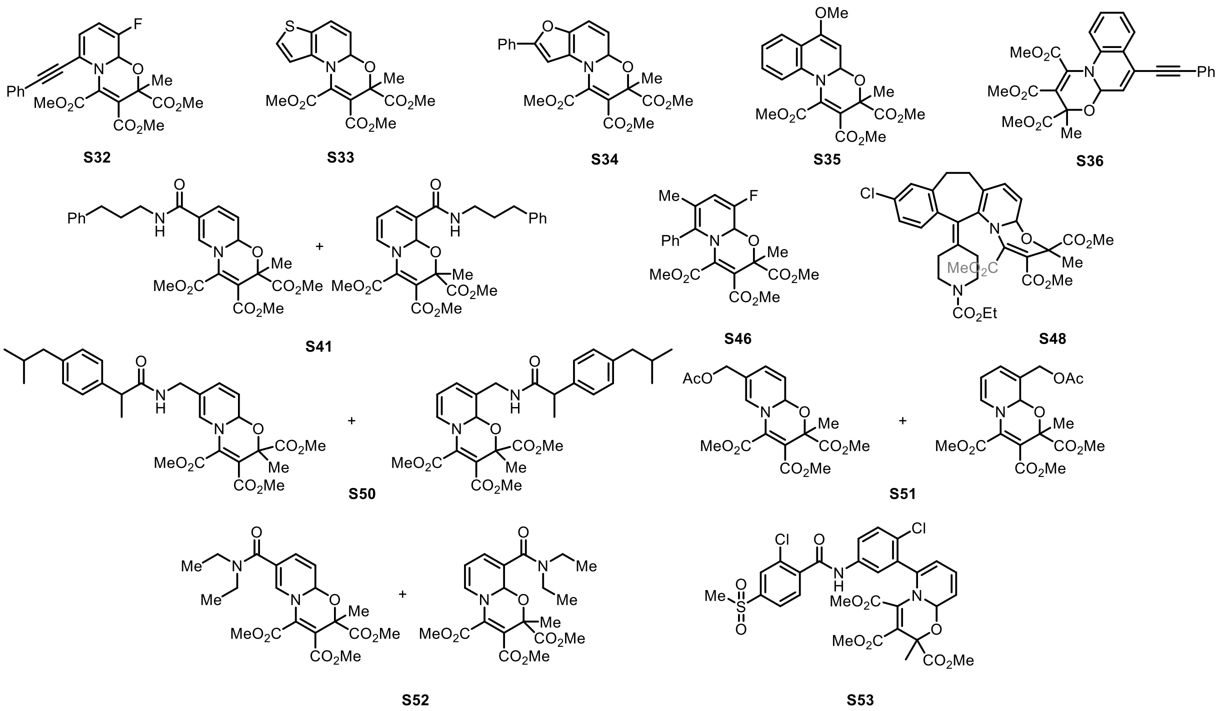

Supplementary Figure 2. Substrates used in this study (part 2).

Trimethyl 2-methyl-2*H*,9a*H*-pyrido[2,1-*b*][1,3]oxazine-2,3,4-tricarboxylate (3)

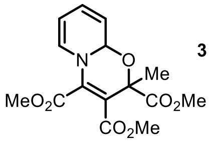

3

Oxazino pyridine **3** was synthesized as a yellow solid (601 mg, 93%, containing diastereomers, d.r. = 4/1) from pyridine (2 mmol scale, reaction time = 2 h) using **GP1**, the physical data is in agreement with those reported in our previous work.[4]

^{1}H NMR (300 MHz, $CDCl_3$) δ 6.35-6.18 (m, 2H), 5.94-5.50 (m, 2H), 5.35-5.24 (m, 1H), 3.93 (s, 3H), 3.83-3.65 (m, 6H), 1.81-1.70 (m, 3H).

Trimethyl 2-methyl-8-phenyl-2*H*,9a*H*-pyrido[2,1-*b*][1,3]oxazine-2,3,4-tricarboxylate (S8)

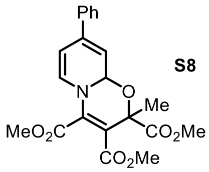

S8

Oxazino pyridine **S8** was synthesized as a yellow solid (782.8 mg, 98%, containing diastereomers, d.r. = 4/1) from 4-phenylpyridine (2 mmol scale, reaction time = 12 h) using **GP1**, the physical data is in agreement with those reported in our previous work.[4]

^{1}H NMR (400 MHz, $CDCl_3$): δ 7.44 (ddd, *J* = 6.1, 3.4, 1.5 Hz, 2H), 7.40 – 7.29 (m, 3H), 6.45 – 6.37 (m, 1H), 5.97 – 5.73 (m, 2H), 5.65 – 5.58 (m, 1H), 3.95 (s, 3H), 3.80 – 3.69 (m, 6H), 1.78 (s, 3H).

Trimethyl 8-(4-bromophenyl)-2-methyl-2H,9a*H*-pyrido[2,1-*b*][1,3]oxazine-2,3,4-tricarboxylate (S9)

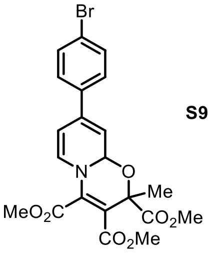

S9

Oxazino pyridine **S9** was synthesized as a yellow solid (816.1 mg, 79%, containing diastereomers, d.r. = 4/1) from 4-(4-bromophenyl)pyridine (2.25 mmol scale, reaction time = 12 h) using **GP1**. R_f = 0.3 (pentane/EtOAc 2:1).

^{1}H NMR (400 MHz, $CDCl_3$) δ 7.49 (d, *J* = 8.4 Hz, 2H), 7.31 (d, *J* = 8.4 Hz, 2H), 6.44 – 6.40 (m, 1H), 6.00 – 5.68 (m, 2H), 5.55 (dd, *J* = 7.6, 1.6 Hz, 1H), 3.94 (s, 3H), 3.77 (s, 2.4H), 3.73 (s, 3H), 3.72 (s, 0.6H), 1.78 (s, 2.4H), 1.76 (s, 0.6H).

^{13}C NMR (100MHz, $CDCl_3$) δ 171.2, 170.4, 164.6, 163.4, 141.8, 137.3, 137.2, 136.8, 136.5, 131.8, 131.7, 127.5(9), 127.5(7), 126.3, 126.2, 122.5(2), 122.4(6), 114.1, 111.7, 111.4, 101.5, 101.3, 79.5, 78.8, 78.0, 76.9, 53.3, 53.2, 52.7, 52.0, 23.8, 23.7.

HRMS (ESI) calcd $C_{21}H_{20}NO_7BrH^+$ $[M+H]^+$: 478.0496. Found: 478.0494.

Melting point: 72-74 °C.

Trimethyl 8-(4-(benzyloxy)phenyl)-2-methyl-2*H*,9a*H*-pyrido[2,1-*b*][1,3]oxazine-2,3,4-tricarboxylate (S10)

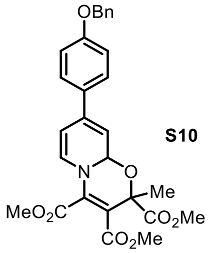

S10

Oxazino pyridine **S10** was synthesized as a yellow solid (568.3 mg, 56%, containing diastereomers, d.r. = 4/1) from 4-(4-(benzyloxy)phenyl)pyridine (2 mmol scale, reaction time = 12 h) using **GP1**. R_f = 0.3 (pentane/EtOAc 2:1).

^{1}H NMR (400 MHz, $CDCl_3$) δ 7.49 – 7.36 (m, 6H), 7.36 – 7.29 (m, 1H), 6.97 (d, *J* = 8.8 Hz, 2H), 6.42 – 6.38 (m, 1H), 5.92 (d, *J* = 3.6 Hz, 0.8H), 5.75 (d, *J* = 3.6 Hz, 0.2H), 5.74 – 5.67 (m, 1H), 5.60 (dd, *J* = 7.6, 1.2 Hz, 1H), 5.08 (s, 2H), 3.95 (d, *J* = 1.5 Hz, 3H), 3.78 (s, 2.4H), 3.73 (s, 3H), 3.72 (s, 0.6H), 1.79 (s, 2.4H), 1.77 (s, 0.6H).

^{13}C NMR (100 MHz, $CDCl_3$) δ 171.3, 164.6, 163.6, 159.0, 142.1, 136.9, 136.8, 136.6, 131.0, 130.9, 128.6, 128.0, 127.4, 127.2, 127.1, 125.8, 125.7, 114.9(0), 114.8(8), 113.6, 110.2, 109.9, 102.2, 102.0, 79.7, 79.0, 77.8(5), 76.8(3), 70.0, 53.3, 53.1, 52.7, 52.0(0), 51.9(8), 23.9, 23.7.

HRMS (ESI) calcd $C_{28}H_{27}NO_8H^+$ $[M+H]^+$: 507.1843. Found: 507.1844.

Melting point: 72-73 °C.

Trimethyl 8-(furan-2-yl)-2-methyl-2*H*,9a*H*-pyrido[2,1-*b*][1,3]oxazine-2,3,4-tricarboxylate (S11)

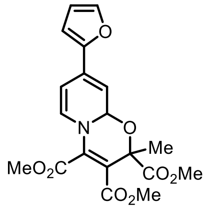

S11

Oxazino pyridine **S11** was synthesized as a yellow solid (465.1 mg, 52%, containing diastereomers, d.r. = 4/1) from 4-(furan-2-yl)pyridine (2.30 mmol scale, reaction time = 12 h) using **GP1**. R_f = 0.4 (pentane/EtOAc 2:1).

^{1}H NMR (400 MHz, $CDCl_3$) δ 7.40 (d, *J* = 1.6 Hz, 1H), 6.48 (d, *J* = 3.2 Hz, 1H), 6.41 (dd, *J* = 3.2, 1.6 Hz, 1H), 6.37 (d, *J* = 7.6 Hz, 1H), 5.99 – 5.70 (m, 2H), 5.59 (dd, *J* = 7.6, 1.6 Hz, 1H), 3.93 (s, 3H), 3.77 (s, 3H), 3.72 (s, 2.4H), 3.71 (s, 0.6H), 1.77 (s, 2.4H), 1.76 (s, 0.6H).

^{13}C NMR (100 MHz, $CDCl_3$) δ 171.2, 170.4, 164.8, 164.6, 163.5(0), 163.4(7), 151.3, 151.2, 142.9(4), 142.8(7), 142.5, 142.1, 127.1, 126.9, 126.0, 125.9, 113.8, 112.9, 111.4, 107.5, 107.3, 107.2, 99.1, 99.0,

79.3, 78.5, 77.9, 76.9, 53.3, 53.1, 52.7, 52.0(2), 52.0(0), 23.8, 23.6.

HRMS (ESI) calcd $C_{19}H_{19}NO_8H^+$ $[M+H]^+$: 390.1183. Found: 390.1178.

Melting point: 81-82 °C.

Trimethyl 8-(benzo[*b*]thiophen-2-yl)-2-methyl-2*H*,9a*H*-pyrido[2,1-*b*][1,3]oxazine-2,3,4-tricarboxylate (S12)

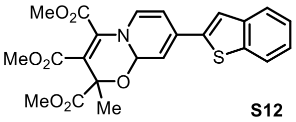

S12

Oxazino pyridine **S12** was synthesized as a yellow solid (381.3 mg, 60%, containing diastereomers, d.r. = 4/1) from 4-(benzo[*b*]thiophen-2-yl)pyridine (1.40 mmol scale, reaction time = 12 h) using **GP1**. R_f = 0.4 (pentane/EtOAc 2:1).

^{1}H NMR (400 MHz, $CDCl_3$) δ 7.82 – 7.75 (m, 1H), 7.74 – 7.72 (m, 1H), 7.39 (s, 1H), 7.36 – 7.30 (m, 2H), 6.51 – 6.32 (m, 1H), 6.07 – 5.60 (m, 3H), 3.95 (d, *J* = 1.8 Hz, 3H), 3.80 (s, 2.4H), 3.74 (s, 3H), 3.73 (s, 0.6H), 1.80 (s, 2.4H), 1.78 (s, 0.6H).

^{13}C NMR (100 MHz, $CDCl_3$) δ 171.1, 170.4, 164.7, 164.6, 163.4(0), 163.3(8), 142.3, 141.8, 141.4, 141.3, 139.9(3), 139.9(1), 139.2, 131.5, 131.2, 126.2, 126.0, 125.1(1), 125.0(7), 124.5(9), 124.5(7), 123.9, 123.8, 122.2(4), 121.2(6), 121.2(0), 114.1, 113.1, 111.3, 111.1, 100.5, 100.4, 79.4, 78.6, 78.1, 77.1, 53.4, 53.2, 52.8, 52.1, 52.0, 23.8, 23.7.

HRMS (ESI) calcd $C_{23}H_{21}NO_7SH^+$ $[M+H]^+$: 456.1112. Found: 456.1115.

Melting point: 89-90 °C.

Trimethyl 2-methyl-8-phenoxy-2*H*,9a*H*-pyrido[2,1-*b*][1,3]oxazine-2,3,4-tricarboxylate (S13)

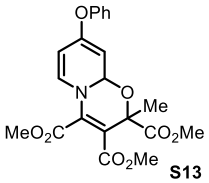

S13

Oxazino pyridine **S13** was synthesized as a yellow solid (822.5 mg, 99%, containing diastereomers, d.r. = 5/1) from 4-phenoxypyridine (2 mmol scale, reaction time = 12 h) using **GP1**, the physical data is in agreement with those reported in our previous work.[4]

^{1}H NMR (400 MHz, $CDCl_3$): δ 7.40 – 7.30 (m, 2H), 7.21 – 7.13 (m, 1H), 7.10 – 7.03 (m, 2H), 6.39 – 6.30 (m, 1H), 5.85 – 5.59 (m, 1H), 5.35 – 5.29 (m, 1H), 4.65 – 4.56 (m, 1H), 3.92 (s, 3H), 3.75 – 3.66 (m, 6H), 1.75 (s, 3H).

Trimethyl 2-methyl-6-phenyl-2*H*,9a*H*-pyrido[2,1-*b*][1,3]oxazine-2,3,4-tricarboxylate (6)

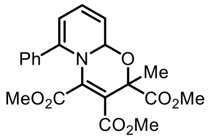

6

Oxazino pyridine **6** was synthesized as a yellow solid (663.0 mg, 83%, containing diastereomers, d.r. = 1/1) from 2-phenylpyridine (2 mmol scale, reaction time = 48 h) using **GP1**, the physical data is in agreement with those reported in our previous work.[4]

^{1}H NMR (300 MHz, $CDCl_3$) δ 7.50-7.15 (m, 5H), 6.56-6.35 (m, 1H), 5.79-5.63 (m, 1H), 5.57-5.18 (m, 2H), 3.90-3.57 (m, 6H), 3.13 (s, 3H), 1.96-1.61 (m, 3H).

Trimethyl 6-(4-(*tert*-butyl)phenyl)-2-methyl-2*H*,9a*H*-pyrido[2,1-*b*][1,3]oxazine-2,3,4-tricarboxylate (S15)

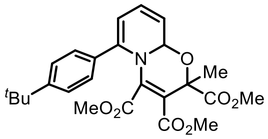

S15

Oxazino pyridine **S15** was synthesized as a yellow solid (796.6 mg, 67%, containing diastereomers, d.r. = 1/1) from 2-(4-(tert-butyl)phenyl)pyridine (3 mmol scale, reaction time = 48 h) using **GP1**, R_f = 0.5 (pentane/EtOAc 2:1).

^{1}H NMR (400 MHz, $CDCl_3$) δ 7.40 – 7.31 (m, 3H), 7.20 – 7.15 (m, 1H), 6.52 – 6.39 (m, 1H), 5.55 – 5.31 (m, 1H), δ 5.54 (dd, *J* = 4.0, 0.8 Hz, 0.5H), 5.33 (dd, *J* = 6.0, 0.8 Hz, 0.5H), 5.30 – 5.24 (m, 1H), 3.86 (s, 1.4H), 3.76 (s, 1.6H), 3.67 (s, 1.4H), 3.65 (s, 1.6H), 3.10 (s, 3H), 1.92 (s, 1.6H), 1.64 (s, 1.4H), 1.31 (s, 9H).

^{13}C NMR (100 MHz, $CDCl_3$) δ 170.9, 170.2, 165.8, 165.3, 163.1, 162.8, 151.7, 151.6, 142.1, 141.0, 139.5, 138.7, 133.2, 132.9, 128.1, 127.3, 126.7, 125.1, 124.1, 123.0, 114.1, 113.3, 103.6, 103.6, 81.6, 80.6, 78.6, 78.0, 53.2, 52.7, 52.3, 52.1(2), 52.1(1), 52.0, 34.6(8), 34.6(6), 31.2(3), 31.2(1), 24.6, 22.8

HRMS (ESI) calcd. for $C_{25}H_{29}NO_7Na^+$ $[M+Na]^+$: 478.1836. found: 478.1834.

Melting point: 69-71 °C.

Trimethyl 6-(3-methoxyphenyl)-2-methyl-2*H*,9a*H*-pyrido[2,1-*b*][1,3]oxazine-2,3,4-tricarboxylate (S16)

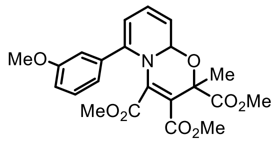

S16

Oxazino pyridine **S16** was synthesized as a yellow solid (1.1 g, 86%, containing diastereomers, d.r. = 1.5/1) from 2-(3-methoxyphenyl)pyridine (3 mmol scale, reaction time = 48 h) using **GP1**, R_f = 0.3 (pentane/EtOAc 2:1).

^{1}H NMR (500 MHz, $CDCl_3$) δ 7.22 (m, 1H), 7.11 – 6.97 (m, 1H), 6.87 – 6.72 (m, 2H), 6.51 – 6.38 (m, 1H), 5.76 – 5.64 (m, 1H), 5.53 (dd, *J* = 4.5, 1.0 Hz, 0.6H), 5.37 (dd, *J* = 6.0, 1.0 Hz, 0.4H), 5.32 (dd, *J* = 6.0, 1.0 Hz, 0.6H), 5.26 (dd, *J* = 4.5, 1.0 Hz, 0.4H), 3.83 (s, 1.2H), 3.80 (s, 1.2H), 3.78 (s, 1.8H), 3.75 (s, 1.8H), 3.67 (s, 1.2H), 3.65 (s, 1.8H), 3.19 (s, CH_3 major), 3.18 (s, CH_3 minor), 1.91 (s, 1.7H), 1.64 (s, 1.3H).

^{13}C NMR (125 MHz, $CDCl_3$) δ 170.8, 170.1, 165.8, 165.3, 162.8, 162.5, 159.3, 141.6, 140.6, 139.3, 138.6, 137.4, 137.0, 129.4, 127.1, 126.5, 124.5, 123.4, 120.6, 114.7, 114.6, 114.4, 113.6, 103.6(5), 103.5(6), 81.4, 80.5, 78.7, 78.0, 55.4, 55.2, 53.1, 52.7, 52.3, 52.1(2), 52.0(6), 24.5, 22.7.

HRMS (ESI) calcd. for $C_{22}H_{23}NO_8Na^+$ $[M+Na]^+$, 452.1316. found: 452.1312.

Melting point: 47-50 °C.

Trimethyl 6-(2,4-difluorophenyl)-2-methyl-2*H*,9a*H*-pyrido[2,1-*b*][1,3]oxazine-2,3,4-tricarboxylate (53)

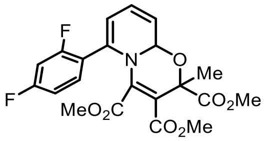

53

Oxazino pyridine **53** was synthesized as a yellow solid (818.5 mg, 94%, containing diastereomers, d.r. = 1/1) from 2-(2,4-difluorophenyl)pyridine (2 mmol scale, reaction time = 48 h) using **GP1**, the physical data is in agreement with those reported in our previous work.[4]

^{1}H NMR (400 MHz, $CDCl_3$) δ 7.59 – 7.20 (m, 1H), 6.92 – 6.73 (m, 2H), 6.52 – 6.36 (m, 1H), 5.80 – 5.69 (m, 1H), 5.57 – 5.21 (m, 2H), 3.90 – 3.14 (m, 9H), 1.91 – 1.61 (m, 3H).

^{19}F NMR (376 MHz, $CDCl_3$): δ -107.4, -107.4, -107.6, -107.6.

Trimethyl 2-methyl-7-phenyl-2*H*,9a*H*-pyrido[2,1-*b*][1,3]oxazine-2,3,4-tricarboxylate and Trimethyl 2-methyl-9-phenyl-2*H*,9a*H*-pyrido[2,1-*b*][1,3]oxazine-2,3,4-tricarboxylate (S18)

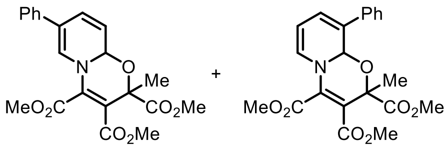

S18

Oxazino pyridine **S18** was synthesized as a yellow solid (766.8 mg, 96%, containing regioisomers and diastereomers, ratio = 6/5/1/1) from 3-phenyl pyridine (2 mmol scale, reaction time = 48 h) using **GP1**, the physical data is in agreement with those reported in our previous work.[4]

^{1}H NMR (300 MHz, $CDCl_3$) δ 7.55 – 7.17 (m, 5H), 6.75 – 6.31 (m, 2H), 6.30 – 5.40 (m, 2H), 4.03 – 3.87 (m, 3H), 3.81-3.63 (m, 6H), 1.84 – 1.67 (m, 3H).

Trimethyl 7-benzyl-2-methyl-2*H*,9a*H*-pyrido[2,1-*b*][1,3]oxazine-2,3,4-tricarboxylate and Trimethyl 9-benzyl-2-methyl-2*H*,9a*H*-pyrido[2,1-*b*][1,3]oxazine-2,3,4-tricarboxylate (S19)

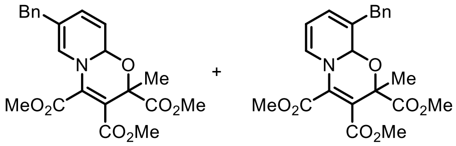

S19

Oxazino pyridine **S19** was synthesized as a yellow solid (785.5 mg, 95%, containing regioisomers and diastereomers, ratio = 19/5/3/1) from 3-benzylpyridine (2 mmol scale, reaction time = 24 h) using **GP1**, the physical data is in agreement with those reported in our previous work.[4]

^{1}H NMR (300 MHz, $CDCl_3$) δ 7.33 – 7.26 (m, 2H), 7.24 – 7.13 (m, 3H), 6.22 – 5.96 (m, 2H), 5.81 – 5.50 (m, 1H), 5.28 – 5.18 (m, 1H), 3.94 – 3.83 (m, 3H), 3.77 – 3.50 (m, 8H), 1.83 – 1.65 (m, 3H).

trimethyl 7-((furan-2-carboxamido)methyl)-2-methyl-2*H*,9a*H*-pyrido[2,1-*b*][1,3]oxazine-2,3,4-tricarboxylate and trimethyl 9-((furan-3-carboxamido)methyl)-2-methyl-2*H*,9a*H*-pyrido[2,1-*b*][1,3]oxazine-2,3,4-tricarboxylate (S20)

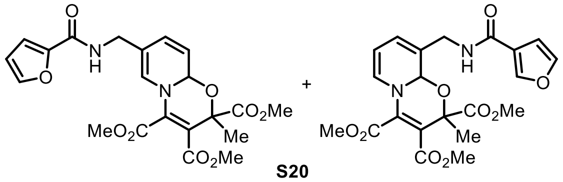

S20

Oxazino pyridine **S20** was synthesized as a yellow solid (866 mg, 97%, containing regioisomers and diastereomers, ratio = 12/3/2/1) from *N*-(pyridin-3-ylmethyl)furan-2-carboxamide (2 mmol scale, reaction time = 24 h) using **GP1**, the physical data is in agreement with those reported in our previous work.[4]

^{1}H NMR (300 MHz, $CDCl_3$) δ 7.43 – 7.37 (m, 1H), 7.09 – 7.03 (m, 1H), 7.02 – 6.91 (m, 1H), 6.48 – 6.42 (m, 1H), 6.33-6.13 (m, 2H), 5.76 – 5.08 (m, 2H), 4.43 – 3.98 (m, 2H), 3.88 – 3.59 (m, 9H), 2.01 – 1.72

(m, 3H).

Trimethyl 7-((5-bromopyridin-2-yl)oxy)-2-methyl-2*H*,9a*H*-pyrido[2,1-*b*][1,3]oxazine-2,3,4-tricarboxylate and **trimethyl 9-((5-bromopyridin-2-yl)oxy)-2-methyl-2*H*,9a*H*-pyrido[2,1-*b*][1,3]oxazine-2,3,4- tricarboxylate (S21)**

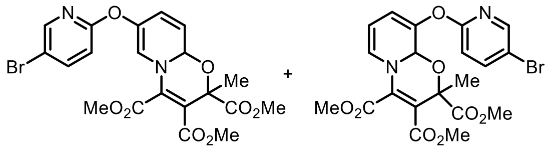

S21

Oxazino pyridine **S21** was synthesized as a yellow solid (971.7 mg, 88%, containing regioisomers and diastereomers, ratio = 22/5/2/1) from 5-bromo-2-(pyridin-3-yloxy)pyridine (2 mmol scale, reaction time = 48 h) using **GP1**, the physical data is in agreement with those reported in our previous work.[4]

¹H NMR (400 MHz, $CDCl_3$): δ 8.24 – 7.70 (m, 2H), 6.90 – 6.83 (m, 1H), 6.25 – 5.94 (m, 3H), 5.29 – 5.22 (m, 1H), 3.96 – 3.35 (m, 9H), 1.74 – 1.37 (m, 3H).

Trimethyl 2,7-dimethyl-6-phenyl-2*H*,9a*H*-pyrido[2,1-*b*][1,3]oxazine-2,3,4-tricarboxylate (S22)

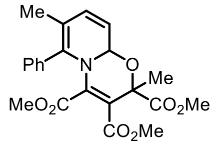

S22

Oxazino pyridine **S22** was synthesized as a yellow solid (702.8 mg, 85%, containing diastereomers, d.r. = 2/1) from 3-methyl-2-phenylpyridine (2 mmol scale, reaction time = 48 h) using **GP1**, the physical data is in agreement with those reported in our previous work.[4]

¹H NMR (400 MHz, $CDCl_3$) δ 7.50 – 6.98 (m, 5H), 6.54 – 6.22 (m, 1H), 5.92 – 5.64 (m, 1H), 5.52 – 5.36 (m, 1H), 3.82 – 2.99 (m, 9H), 1.99 – 1.74 (m, 3H), 1.74 – 1.57 (m, 3H).

Trimethyl 7-bromo-2-methyl-6-phenyl-2*H*,9a*H*-pyrido[2,1-*b*][1,3]oxazine-2,3,4-tricarboxylate (S23)

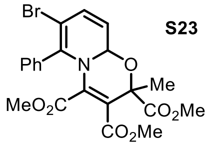

S23

Oxazino pyridine **S23** was synthesized as a yellow solid (1.20 g, 84%, containing diastereomers, d.r. = 1/1) from 3-bromo-2-phenylpyridine (3 mmol scale, reaction time = 48 h) using **GP1**, R_f = 0.5 (pentane/EtOAc 2:1).

^{1}H NMR (400 MHz, $CDCl_3$) δ 7.40 – 7.37 (m, 4.5H), 7.08 (s, 0.5H), 6.62 – 6.56 (m, 1H), 5.72 – 5.65 (m, 1H), 5.50 (d, *J* = 4.4 Hz, 0.5H), 5.23 (d, *J* = 4.4 Hz, 0.5H), 3.87 (s, 1.4H), 3.74 (s, 1.6H), 3.63 (s, CH_3 minor), 3.61 (s, CH_3 major), 3.18 (s, CH_3 major), 3.17 (s, CH_3 minor), 1.88 (s, 1.6H), 1.61 (s, 1.4H).

^{13}C NMR (100 MHz, $CDCl_3$) δ 170.6, 169.8, 165.2, 164.8, 162.9, 141.7, 140.7, 136.8, 136.1, 134.0(4), 133.9(5), 132.6, 132.0, 131.5, 129.2, 129.1, 128.5, 128.0, 127.7, 124.7, 124.1, 115.6, 114.8, 97.5, 97.4, 80.2, 79.3, 78.7, 78.1, 53.2, 52.8, 52.4(0), 52.3(5), 52.3, 52.2, 24.4, 22.6.

HRMS (ESI) calcd $C_{21}H_{20}NO_7BrNa^+$ $[M+Na]^+$: 500.0315. Found: 500.0311.

Melting point: 75-77 °C.

Trimethyl 7-methoxy-2-methyl-6-phenyl-2*H*,9a*H*-pyrido[2,1-*b*][1,3]oxazine-2,3,4-tricarboxylate (S24)

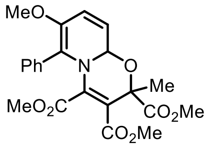

S24

Oxazino pyridine **S24** was synthesized as a yellow solid (826.4 mg, 84%, containing diastereomers, d.r. = 5/1) from 3-methoxy-2-phenylpyridine (2.28 mmol scale, reaction time = 48 h) using **GP1**, R_f = 0.2 (pentane/EtOAc 1:1).

^{1}H NMR (400 MHz, $CDCl_3$) δ 7.59 – 7.46 (m, 2H), 7.38 – 7.19 (m, 3H), 6.55 (d, *J* = 10.0, 0.16H), 6.21 (dd, *J* = 7.6 Hz, 0.84H), 5.82 (dd, *J* = 10.4, 4.4 Hz, 0.16H), 5.45 (d, *J* = 4.4 Hz, 0.16H), 5.31 – 5.17 (m, 0.84H), 5.10 – 4.88 (m, 0.84H), 3.99 (s, CH_3 major), 3.94 (s, CH_3 minor), 3.72 (s, CH_3 minor), 3.62 (s, CH_3 major), 3.61 (s, CH_3 minor), 3.58 (s, CH_3 major), 3.34 (s, CH_3 minor), 3.15 (s, CH_3 major), 1.89 (s, CH_3 minor), 3.86 (s, CH_3 major).

^{13}C NMR (100 MHz, $CDCl_3$) δ 171.0, 170.1, 165.1, 164.4, 150.9, 141.8, 137.1, 128.8, 128.6, 128.1(1), 128.0(5), 126.7, 126.4, 119.2, 107.3, 102.2, 101.3, 92.3, 91.9, 88.0, 74.6, 59.2, 55.9, 55.8, 53.4, 53.2, 53.2(3), 53.1(8), 53.1, 52.9, 52.0, 51.8, 51.6(2), 51.5(6), 26.3, 24.5, 23.5.

HRMS (ESI) calcd $C_{22}H_{23}NO_8H^+$ $[M+H]^+$: 430.1496. Found: 430.1499.

Melting point: 67-68 °C.

Trimethyl 2,7-dimethyl-6-(thiophen-2-yl)-2*H*,9a*H*-pyrido[2,1-*b*][1,3]oxazine-2,3,4-tricarboxylate (S25)

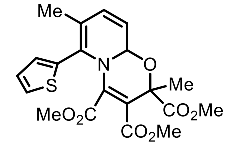

S25

Oxazino pyridine **S25** was synthesized as a yellow solid (899.5 mg, 71%, containing diastereomers, d.r. = 2/1) from 3-methyl-2-(thiophen-2-yl)pyridine (3 mmol scale, reaction time = 48 h) using **GP1**, R_f = 0.4 (pentane/EtOAc 2:1).

^{1}H NMR (300 MHz, $CDCl_3$) δ 7.50 – 7.38 (m, 1H), 7.14 – 6.83 (m, 2H), 6.48 – 6.21 (m, 1H), 5.97 – 5.65 (m, 1H), 5.45 (d, *J* = 4.5 Hz, 0.65H), 5.19 (d, *J* = 4.8 Hz, 0.35H), 3.84 (s, 1H), 3.74 (s, 2H), 3.65 (s, 1H), 3.63 (s, 2H), 3.38 (s, 2H), 3.36 (s, 1H), 1.85 (s, 2H), 1.74 (s, 1H), 1.71 (s, 2H), 1.60 (s, 1H).

^{13}C NMR (100 MHz, $CDCl_3$) δ 170.8, 170.2, 165.2, 163.2, 142.8, 134.8, 130.9, 128.0, 126.8, 123.0, 115.9, 114.9, 112.6, 80.2, 79.5, 78.3, 77.8, 53.0, 52.7, 52.6, 52.5 52.2, 52.0, 24.4, 22.6, 17.7.

HRMS (ESI) calcd $C_{20}H_{21}NO_7SH^+$ $[M+H]^+$: 420.1112. Found: 420.1110.

Melting point: 66-68 °C.

Trimethyl 2-methyl-6,8-diphenyl-2*H*,9a*H*-pyrido[2,1-*b*][1,3]oxazine-2,3,4-tricarboxylate (S26)

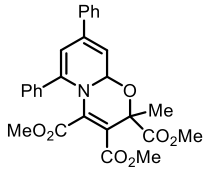

S26

Oxazino pyridine **S26** was synthesized as a yellow solid (282.5 mg, 19%, containing diastereomers, d.r. = 5/1) from 2,4-diphenylpyridine (3.12 mmol scale, reaction time = 48 h) using **GP1**, R_f = 0.5 (pentane/EtOAc 2:1).

^{1}H NMR (400 MHz, $CDCl_3$) δ 7.58 – 7.47 (m, 3H), 7.40 – 7.27 (m, 7H), 5.95 – 5.84 (m, 1H), 5.72 – 5.54 (m, 1.6H), 5.41 (d, *J* = 4.7 Hz, 0.4H), 3.88 (s, 1.2H), 3.79 (s, 1.8H), 3.69 (s, 1.2H), 3.67 (s, 1.8H), 3.20 – 3.15 (m, 3H), 1.96 (s, 1.8H), 1.69 (s, 1.2H).

^{13}C NMR (100 MHz, $CDCl_3$) δ 171.0, 170.2, 165.8, 165.3, 163.0, 162.7, 141.3, 140.4, 139.9, 139.3, 139.2, 138.7, 138.5, 136.1, 135.8, 128.8, 128.7, 128.5, 128.3, 128.2, 126.2(4), 126.1(9), 123.7, 110.0, 109.2, 105.1, 82.1, 81.2, 78.8, 78.2, 53.2, 52.8, 52.4, 52.2, 52.1, 24.6, 22.8.

HRMS (ESI) calcd $C_{27}H_{25}NO_7H^+$ $[M+H]^+$: 476.1704. Found: 476.1704.

Melting point: 73-74 °C.

Trimethyl 2,9-dimethyl-6-phenyl-2*H*,9a*H*-pyrido[2,1-*b*][1,3]oxazine-2,3,4-tricarboxylate (S27)

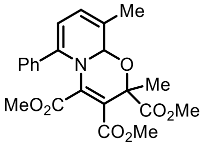

S27

Oxazino pyridine **S27** was synthesized as a yellow solid (1.14 g, 73%, containing diastereomers, d.r. = 1.2/1) from 5-methyl-2-phenylpyridine (3.77 mmol scale, reaction time = 48 h) using **GP1**, R_f = 0.3 (pentane/EtOAc 2:1).

^{1}H NMR (400 MHz, $CDCl_3$) δ 7.48 – 7.38 (m, 1H), 7.35 – 7.27 (m, 3H), 7.25 – 7.20 (m, 1H), 6.30 – 6.09 (m, 1H), 5.37 (s, 0.55H), 5.24 (dd, *J* = 18.4, 6.2 Hz, 1H), 5.08 (s, 0.45H), 3.85 (s, 1.3H), 3.77 (s, 1.7H), 3.66 – 3.65 (m, 3H), 3.13 (s, 3H), 2.02 – 1.95 (m, 3H), 1.92 (s, 1.7H), 1.66 (s, 1.3H).

^{13}C NMR (100 MHz, $CDCl_3$) δ 171.2, 170.3, 165.9, 165.5, 163.2, 162.8, 141.5, 141.0, 136.8, 136.2, 136.1, 135.8, 128.4(3), 128.3(6), 128.3, 128.2, 123.9, 123.7, 123.0, 122.9, 122.3, 104.3, 104.3, 85.0, 84.2, 78.8, 78.4, 53.1, 52.6, 52.3, 52.1(0), 52.0(6), 24.5, 22.5, 18.8, 18.7.

HRMS (ESI) calcd $C_{22}H_{23}NO_7H^+$ $[M+H]^+$: 414.1547. Found: 414.1543.

Melting point: 69-70 °C.

Trimethyl 2,9-dimethyl-6-(thiophen-2-yl)-2*H*,9a*H*-pyrido[2,1-*b*][1,3]oxazine-2,3,4-tricarboxylate (S28)

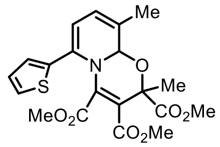

S28

Oxazino pyridine **S28** was synthesized as a yellow solid (1.06 g, 52%, containing diastereomers, d.r. = 1.5/1) from 5-methyl-2-(thiophen-2-yl)pyridine (4.87 mmol scale, reaction time = 48 h) using **GP1**, R_f = 0.5 (pentane/EtOAc 2:1).

^{1}H NMR (400 MHz, $CDCl_3$) δ 7.26 – 7.21 (m, 1H), 7.16 (dd, *J* = 3.6, 1.2 Hz, 0.4H), 6.96 – 6.88 (m, 1.6H), 6.19 – 6.09 (m, 1H), 5.54 – 5.38 (m, 1H), 5.34 (s, 0.6H), 5.06 (s, 0.4H), 3.82 (s, 1.2H), 3.76 (s, 1.8H), 3.70 (s, 1.2H), 3.68 (s, 1.8H), 3.33 (s, 3H), 1.96 (s, 3H), 1.89 (s, 1.8H), 1.63 (s, 1.2H).

^{13}C NMR (100 MHz, $CDCl_3$) δ 171.0, 170.1, 166.0, 165.6, 162.8, 162.4, 140.4, 139.8, 137.3, 137.1, 130.2, 129.5, 127.2, 127.1, 126.9, 126.7, 126.4, 126.1, 126.0, 125.3, 124.6, 123.6, 122.8, 122.1, 105.7, 105.4, 85.1, 84.3, 78.8, 78.6, 53.1, 52.6, 52.4, 52.3(8), 52.3(2), 52.2, 24.6, 22.2, 19.0, 18.8.

HRMS (ESI) calcd $C_{20}H_{21}NO_7SH^+$ $[M+H]^+$: 420.1112. Found: 420.1112.

Melting point: 63-65 °C.

Trimethyl 2,9-dimethyl-6-(phenylethynyl)-2*H*,9a*H*-pyrido[2,1-*b*][1,3]oxazine-2,3,4-tricarboxylate (S29)

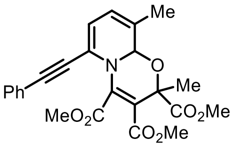

S29

Oxazino pyridine **S29** was synthesized as a yellow solid (1.08 g, 52%, containing diastereomers, d.r. = 2/1) from 5-methyl-2-(phenylethynyl)pyridine (4.77 mmol scale, reaction time = 48 h) using **GP1**, R_f = 0.5 (pentane/EtOAc 2:1).

^{1}H NMR (400 MHz, $CDCl_3$) δ 7.60 – 7.41 (m, 2H), 7.38 – 7.29 (m, 3H), 6.22 – 6.06 (m, 1H), 5.88 – 5.73 (m, 1H), 5.26 (s, 0.63H), 5.01 (s, 0.34H), 3.76 (s, 2H), 3.75 (s, 3H), 3.72 (s, 1H), 3.69 (s, 2H), 3.69 (s, 1H), 2.07 – 1.93 (m, 3H), 1.84 (s, 2H), 1.62 (s, 1H).

^{13}C NMR (100 MHz, $CDCl_3$) δ 171.2, 169.9, 165.8, 165.7, 163.6, 163.2, 139.7, 139.4, 131.3(4), 131.3(2), 128.9, 128.8, 128.4, 128.3, 126.3, 125.6, 125.2, 125.0, 122.3, 122.0, 121.9, 121.8, 119.9, 119.4, 110.6, 110.5, 95.2, 94.9, 83.7, 83.6, 83.5, 82.6, 78.3, 78.1, 53.2, 53.0, 52.9, 52.6, 52.4, 52.3, 24.5, 22.2, 19.4, 19.2.

HRMS (ESI) calcd $C_{24}H_{23}NO_7Na^+$ $[M+Na]^+$: 460.1367. Found: 460.1365.

Melting point: 64-65 °C.

Trimethyl 9-(acetoxymethyl)-2-methyl-6-(thiophen-2-yl)-2*H*,9a*H*-pyrido[2,1-*b*][1,3]oxazine-2,3,4-tricarboxylate (S30)

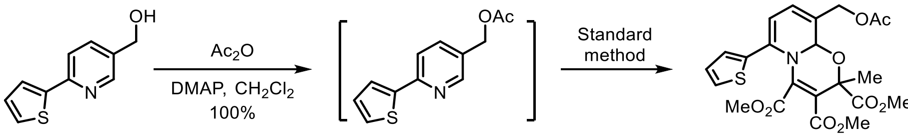

S30

Oxazino pyridine **S30** was synthesized as a yellow gum (961.3 mg, 67%, containing diastereomers, d.r. = 1.5/1) from (6-(thiophen-2-yl)pyridin-3-yl)methanol (reaction time = 48 h) using **GP1**, R_f = 0.5 (pentane/EtOAc 2:1).

^{1}H NMR (400 MHz, $CDCl_3$) δ 7.33 – 7.24 (m, 1H), 7.20 (d, *J* = 3.2 Hz, 0.4H), 7.03 – 6.85 (m, 1.6H), 6.50 – 6.35 (m, 1H), 5.53 (s, 0.6H), 5.51 – 5.42 (m, 1H), 5.28 (s, 0.4H), 4.93 – 4.65 (m, 2H), 3.81 (s, 1.2H), 3.75 (s, 1.8H), 3.71 (s, 1.2H), 3.69 (s, 1.8H), 3.33 (s, 3H), 2.08 (s, 1.2H), 2.07 (s, 1.8H), 1.89 (s, 1.8H), 1.61 (s, 1.2H).

^{13}**C NMR** (100 MHz, $CDCl_3$) δ 170.7(3), 170.6(9), 170.6, 169.8, 166.7, 165.7, 165.4, 164.0, 162.5, 162.1, 139.8, 139.3, 138.7, 136.7, 136.5, 134.1, 133.4, 132.6, 130.0, 127.5, 127.4, 127.2(5), 127.1(7), 127.0, 126.9, 126.8, 126.7, 126.5, 126.4, 121.8, 120.8, 104.4, 104.1, 82.4, 81.6, 79.0, 78.7, 63.9, 63.7, 53.4, 53.1(9), 53.1(7), 53.1(4), 53.1(2), 52.7, 52.5, 52.4(2), 52.3(6), 52.3, 24.5, 22.1, 21.0, 20.9.

HRMS (ESI) calcd $C_{22}H_{23}NO_9SH^+$ $[M+H]^+$: 478.1166. Found: 478.1169.

Trimethyl 9-methoxy-2-methyl-6-(thiophen-2-yl)-2*H*,9a*H*-pyrido[2,1-*b*][1,3]oxazine-2,3,4-tricarboxylate (S31)

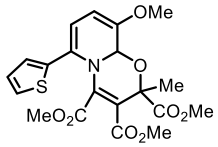

S31

Oxazino pyridine **S31** was synthesized as yellow solid (367.1 mg, 33%, containing diastereomers, d.r. = 1.5/1) from 5-methoxy-2-(thiophen-2-yl)pyridine (2.55 mmol scale, reaction time = 48 h) using **GP1**, R_f = 0.5 (pentane/EtOAc 2:1).

^{1}**H NMR** (400 MHz, $CDCl_3$) δ 7.25 – 7.20 (m, 1H), 7.15 (dd, J = 3.6, 1.2 Hz, 0.4H), 6.96 – 6.88 (m, 1.6H), 5.57 – 5.45 (m, 2H), 5.44 (s, 0.6H), 5.13 (s, 0.4H), 3.81 (s, 1.2H), 3.77 (s, 1.8H), 3.71 (s, 3H), 3.70 (s, CH_3 minor), 3.68 (s, CH_3 major), 3.35-3.32 (m, 3H), 1.92 (s, 1.8H), 1.65 (s, 1.2H).

^{13}**C NMR** (100 MHz, $CDCl_3$) δ 170.7, 169.8, 166.0, 165.6, 162.8, 162.4, 147.7, 146.8, 140.2, 139.3, 137.2, 137.0, 127.5, 127.4, 126.9, 126.7, 126.4, 126.3(4), 126.2(8), 125.8, 125.6, 125.5, 105.2, 104.7, 96.8, 96.3, 82.0, 81.4, 78.8, 78.5, 55.6, 55.5, 53.1, 52.8, 52.5, 52.4(4), 52.3(5), 52.3, 24.5, 22.1.

HRMS (ESI) calcd $C_{20}H_{21}NO_8SH^+$ $[M+H]^+$: 436.1061. Found: 436.1059.

Melting point: 73-75 °C.

Trimethyl 9-fluoro-2-methyl-6-(phenylethynyl)-2*H*,9a*H*-pyrido[2,1-*b*][1,3]oxazine-2,3,4-tricarboxylate (S32)

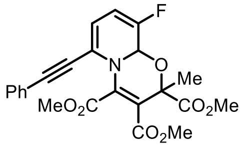

S32

Oxazino pyridine **S32** was synthesized as a yellow solid (741.6 mg, 84%, containing diastereomers, d.r. = 2/1) from 5-fluoro-2-(phenylethynyl)pyridine (2 mmol scale, reaction time = 96 h) using **GP1**, the physical data is in agreement with those reported in our previous work.[4]

^{1}H NMR (400 MHz, $CDCl_3$): δ 7.49 – 7.42 (m, 2H), 7.36 – 7.29 (m, 3H), 6.13 – 6.03 (m, 1H), 5.79 – 5.70 (m, 1H), 5.59 – 5.24 (m, 1H), 3.80 – 3.65 (m, 9H), 1.89 – 1.62 (m, 3H).

^{19}F NMR (282 MHz, $CDCl_3$): δ -121.12, -121.57.

Trimethyl 7-methyl-5a*H*,7*H*-thieno[2',3':5,6]pyrido[2,1-*b*][1,3]oxazine-7,8,9-tricarboxylate (S33)

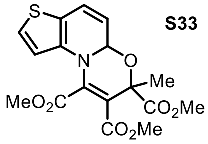

S33

Oxazino pyridine **S33** was synthesized as a yellow solid (1.15 g, 61%, containing diastereomers, d.r. = 2/1) from thieno[3,2-*b*]pyridine (5 mmol scale, reaction time = 24 h) using **GP1**, R_f = 0.3 (pentane/EtOAc 2:1).

^{1}H NMR (400 MHz, $CDCl_3$) δ 7.18 – 7.16 (m, 1H), 6.82 – 6.77 (m, 1H), 6.59 (d, *J* = 5.6 Hz, 1H), 5.75 – 5.70 (m, 1H), 5.62 – 5.33 (m, 1H), 3.91 (s, 3H), 3.76 (s, 2H), 3.75 (s, 3H), 3.66 (s, 1H), 1.76 (s, 2H), 1.68 (s, 1H).

^{13}C NMR (100 MHz, $CDCl_3$) δ 171.0, 169.9, 165.2, 164.9, 164.2, 163.9, 140.7, 140.3, 135.4, 135.1, 125.3, 125.2, 123.2, 122.7, 121.0, 120.7, 116.6, 116.3(0), 116.2(8), 116.2, 114.5, 114.0, 80.0, 79.2, 78.3, 77.7, 53.3, 53.2, 53.2, 52.7, 52.3, 52.2, 24.4, 22.9.

HRMS (ESI) calcd $C_{17}H_{17}NO_7SH^+$ $[M+H]^+$: 380.0798. Found: 380.0795.

Melting point: 138-140 °C.

Trimethyl 7-methyl-2-phenyl-5a*H*,7*H*-furo[2',3':5,6]pyrido[2,1-*b*][1,3]oxazine-7,8,9-tricarboxylate (S34)

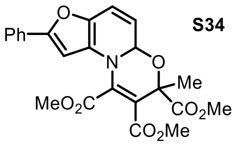

S34

Oxazino pyridine **S34** was synthesized as an orange solid (420.1 mg, 24%, single diastereomer were isolated and used (other part containing both diastereomers was also obtained in 436.9 mg)) from 2-phenylfuro[3,2-*b*]pyridine (4 mmol scale, , reaction time = 24 h) using **GP1**, R_f = 0.3 (pentane/EtOAc 2:1).

^{1}H NMR (400 MHz, $CDCl_3$) δ 7.60 – 7.58 (m, 2H), 7.37 (t, *J* = 7.6 Hz, 2H), 7.30 – 7.26 (m, 1H), 6.83 (d, *J* = 10.4 Hz, 1H), 6.29 (s, 1H), 5.75 (dd, *J* = 4.0, 1.2 Hz, 1H), 5.59 (dd, *J* = 10.0, 4.0 Hz, 1H), 4.00 (s, 3H), 3.78 (s, 3H), 3.75 (s, 3H), 1.78 (s, 3H).

^{13}C NMR (100 MHz, $CDCl_3$) δ 171.1, 164.7, 164.1, 153.3, 141.0, 137.0, 129.8, 128.8, 128.2, 126.1, 123.9, 118.8, 116.1, 113.8, 96.2, 80.5, 77.2, 53.3, 52.7, 52.0, 24.1.

HRMS (ESI) calcd $C_{23}H_{21}NO_8Na^+$ $[M+Na]^+$: 462.1159. Found: 462.1160.

Melting point: 146-149 °C.

Trimethyl 6-methoxy-3-methyl-3*H*,4a*H*-[1,3]oxazino[3,2-*a*]quinoline-1,2,3-tricarboxylate (S35)

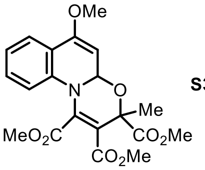

S35

Oxazino pyridine **S35** was synthesized as a light yellow solid (766.0 mg, 76%, containing diastereomers, d.r. = 5/1) from 4-methoxyquinoline (2.5 mmol scale, reaction time = 48 h) using **GP1**, R_f = 0.5 (pentane/EtOAc 2:1).

^{1}H NMR (400 MHz, $CDCl_3$) δ 7.32 – 7.29 (m, 1H), 7.16 – 7.10 (m, 1H), 6.53 (d, *J* = 8.3 Hz, 1H), 6.36 (d, *J* = 8.3 Hz, 1H), 5.99 – 5.77 (m, 1H), 5.45 (d, *J* = 4.4 Hz, 0.16H), 5.18 (d, *J* = 4.4 Hz, 0.84H), 3.87 – 3.77 (m, 9H), 3.77 (s, 0.5H), 3.62 (s, 2.5H), 1.75 (s, 0.5H), 1.63 (s, 2.5H).

^{13}C NMR (100 MHz, $CDCl_3$) δ 169.6, 165.7, 163.8, 156.1, 156.1, 139.1, 137.1, 136.7, 129.8, 129.7, 127.7, 127.1, 123.6, 123.1, 116.6, 116.2, 110.5, 107.7, 107.1, 104.3, 104.1, 79.1, 78.7, 78.1, 55.8, 55.7, 53.2, 53.1(1), 53.0(7), 52.8, 52.5, 52.4, 24.9, 22.4.

HRMS (ESI) calcd $C_{20}H_{21}NO_8Na^+$ $[M+Na]^+$: 426.1159. Found: 426.1160.

Melting point: 177-179 °C.

Trimethyl 3-methyl-6-(phenylethynyl)-3*H*,4a*H*-[1,3]oxazino[3,2-*a*]quinoline-1,2,3-tricarboxylate (S36)

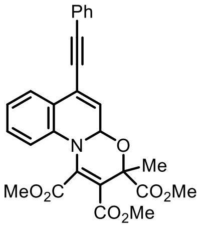

S36

Oxazino pyridine **S36** was synthesized as a light yellow solid (1.04 g, 55%, containing diastereomers, d.r. = 2/1) from 4-(phenylethynyl)quinoline (4.0 mmol scale, reaction time = 48 h) using **GP1**, R_f = 0.6 (pentane/EtOAc 2:1).

^{1}H NMR (400 MHz, $CDCl_3$) δ 7.83 (dd, *J* = 7.6, 1.6 Hz, 1H), 7.58 – 7.54 (m, 2H), 7.42 – 7.34 (m, 3H), 7.31 – 7.22 (m, 1H), 7.08 (t, *J* = 7.5 Hz, 1H), 6.79 – 6.68 (m, 1H), 6.34 (d, *J* = 4.8 Hz, 0.66H), 6.30 (d, *J* = 4.8 Hz, 0.34H), 5.56 (d, *J* = 4.8 Hz, 0.66H), 5.27 (d, *J* = 4.8 Hz, 0.34H), 3.85 (s, 2H), 3.85 (s, 1H), 3.80 (s, 1H), 3.79 (s, 2H), 3.78 (s, 2H), 3.62 (s, 1H), 1.77 (s, 2H), 1.66 (s, 1H).

^{13}C NMR (100 MHz, $CDCl_3$) δ 170.8, 169.3, 165.6, 165.2, 164.0, 163.6, 139.3, 138.4, 136.2, 135.8, 131.8, 130.1, 130.0, 128.8(9), 128.8(7), 128.4, 127.9, 127.4, 127.2, 123.8, 123.4, 122.5, 122.4, 122.2, 122.0, 121.9, 121.8, 120.2(2), 120.1(8), 114.9, 114.3, 93.9, 84.5, 84.5, 78.9, 78.8, 78.7, 77.8, 53.2(2), 53.1(6), 53.1, 52.8, 52.6, 52.4, 24.8, 22.3.

HRMS (ESI) calcd $C_{27}H_{23}NO_7Na^+$ $[M+Na]^+$: 496.1367. Found: 496.1362.

Melting point: 81-82 °C.

Trimethyl 2-methyl-7-((3-phenylpropyl)carbamoyl)-2*H*,9a*H*-pyrido[2,1-*b*][1,3]oxazine-2,3,4-tricarboxylate and Trimethyl 2-methyl-9-((3-phenylpropyl)carbamoyl)-2*H*,9a*H*-pyrido[2,1-*b*][1,3]oxazine-2,3,4-tricarboxylate (S41)

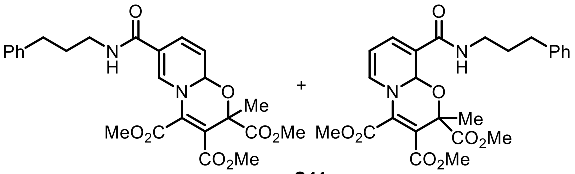

S41

Oxazino pyridine **S41** was synthesized as a yellow solid (852.7 mg, 88% yield, containing regioisomers and diastereomers, ratio = 17/5/1/1) from *N*-(3-phenylpropyl)nicotinamide (2 mmol scale, reaction time = 48 h) using **GP1**, the physical data is in agreement with those reported in our previous work.[4]

^{1}H NMR (300 MHz, $CDCl_3$) δ 7.86 (t, *J* = 5.32 Hz, 1H), 7.49 – 7.10 (m, 6H), 6.49 – 6.28 (m, 1H), 5.86 – 5.41 (m, 2H), 3.98 – 3.67 (m, 9H), 3.50 – 3.30 (m, 2H), 2.76 – 2.62 (m, 2H), 1.99 – 1.58 (m, 5H).

Trimethyl 9-fluoro-2,7-dimethyl-6-phenyl-2*H*,9a*H*-pyrido[2,1-*b*][1,3]oxazine-2,3,4-tricarboxylate (S46)

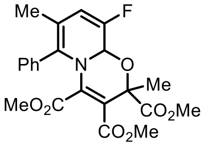

S46

Oxazino pyridine **S46** was synthesized as a yellow gum (991.6 mg, 92%, containing diastereomers, d.r. = 4/1) from 5-fluoro-3-methyl-2-phenylpyridine (reaction time = 48 h) using **GP1**, R_f = 0.5 (pentane/EtOAc 2:1).

^{1}H NMR (500 MHz, $CDCl_3$) δ 7.45 (s, 0.4H), 7.39 – 7.28 (m, 3H), 7.19 (s, 1H), 7.06 (s, 0.6H), 6.06 (d, *J* = 11.1 Hz, 0.4H), 6.02 (d, *J* = 11.1 Hz, 0.6H), 5.57 (d, *J* = 5.2 Hz, 0.6H), 5.26 (d, *J* = 5.5 Hz, 0.4H), 3.86

(s, 1.2H), 3.76 (s, 1.8H), 3.62 (s, 1.2H), 3.61 (s, 1.8H), 3.16 (s, 1.8H), 3.15 (s, 1.2H), 1.90 (s, 1.8H), 1.64 (s, 1.2H), 1.63 (s, 1.2H), 1.60 (s, 1.8H).

^{13}C NMR (125 MHz, $CDCl_3$) δ 170.5, 169.9, 165.5, 165.0, 163.2, 162.9, 150.3, 149.6, 148.2, 147.5 142.1, 141.0, 133.4, 133.3, 132.5, 132.3, 130.8(5), 130.8(2), 130.2, 130.1, 129.6, 128.8, 128.6, 128.4, 128.0, 127.7, 123.6, 123.0, 109.9, 109.8, 109.4, 109.3, 106.4(5), 106.4(2), 106.2, 106.2, 79.4, 79.1, 78.8, 78.4(9), 78.4(7), 78.0, 53.1, 52.8, 52.4, 52.3, 52.2, 52.1, 24.3, 22.4, 17.6(5), 17.6(3), 17.6(1).

^{19}F NMR (470 MHz, $CDCl_3$) δ -125.94, -126.82.

HRMS (ESI) calcd. for $C_{22}H_{22}NO_7FNa^+$ $[M+Na]^+$: 454.1272. found: 454.1272.

Trimethyl 10-chloro-13-(1-(ethoxycarbonyl)piperidin-4-ylidene)-3-methyl-4a,7,8,13-tetrahydro-3*H*-benzo[4',5']cyclohepta[1',2':5,6]pyrido[2,1-*b*][1,3]oxazine-1,2,3-tricarboxylate (S48)

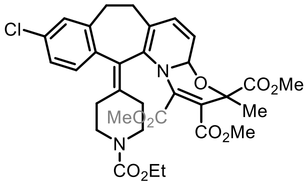

S48

Oxazino pyridine **S48** was synthesized as a yellow solid (1217 mg, 97% yield, containing diastereomers, d.r. = 2/1) from loratadine (2 mmol scale, reaction time = 12 h) using **GP1**, the physical data is in agreement with those reported in our previous work.[4]

^{1}H NMR (400 MHz, $CDCl_3$) δ 7.20 – 7.11 (m, 2H), 7.01 – 6.92 (m, 1H), 6.22 – 6.12 (m, 1H), 5.72 – 5.53 (m, 1H), 5.45 – 5.25 (m, 1H), 4.19 – 4.09 (m, 2H), 4.04 – 3.63 (m, 11H), 3.14 – 2.92 (m, 2H), 2.88 – 2.61 (m, 3H), 2.43 – 2.26 (m, 3H), 2.24 – 2.13 (m, 1H), 2.07 – 1.96 (m, 1H), 1.90 – 1.50 (m, 3H), 1.31 – 1.18 (m, 3H).

Trimethyl 7-((2-(4-isobutylphenyl)propanamido)methyl)-2-methyl-2*H*,9a*H*-pyrido[2,1-*b*][1,3]oxazine-2,3,4-tricarboxylate and trimethyl 9-((2-(4-isobutylphenyl)propanamido)methyl)-2-methyl-2*H*,9a*H*-pyrido[2,1-*b*][1,3]oxazine-2,3,4-tricarboxylate (S50)

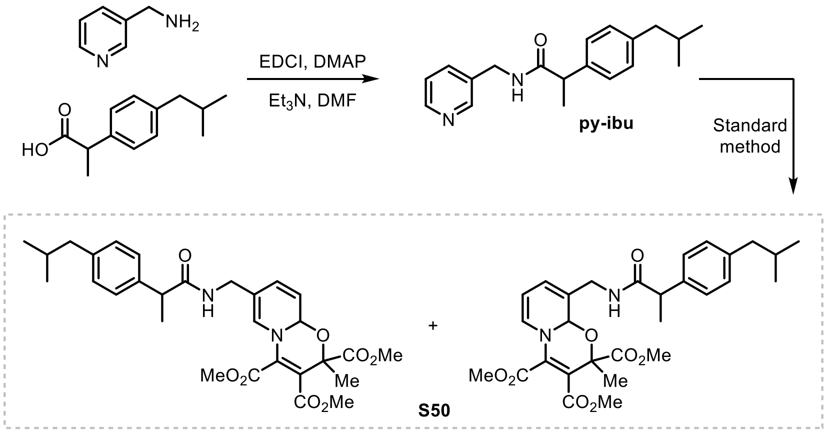

Pyridine **py-ibu** was synthesized from pyridin-3-ylmethanamine and ibuprofen. To a 100 mL round bottle flask ibuprofen (10 mmol, 2.06 g, 1 equiv.), 1-Ethyl-3-(3-dimethylaminopropyl)carbodiimide (EDCI, 10.2 mmol, 1.96 g, 1.02 equiv.), DMF (30 mL), Et_3N (15 mmol, 2.08 mL, 1.5 equiv.) and pyridin-3-ylmethanamine (1.08 g, 10 mmol, 1 equiv.) were added sequentially. The resultant mixture was stirred at r.t. for 24 h. Afterwards, the mixture was diluted with EtOAc (50 mL) and washed with water (50 mL x 3). The combined organic phase was dried over Na_2SO_4, filtered, concentrated and submitted to flash column chromatography (R_f = 0.2, EtOAc) to give **py-ibu** as a white solid (935.0 mg, 32%)

^{1}H NMR (300 MHz, $CDCl_3$) δ 8.46 (dd, *J* = 4.8, 1.8 Hz, 1H), 8.42 (d, *J* = 2.4 Hz, 1H), 7.49 (d, *J* = 7.8 Hz, 1H), 7.22 – 7.17 (m, 3H), 7.11 (d, *J* = 8.1 Hz, 2H), 5.83 (s, 1H), 4.39 (d, *J* = 6.0 Hz, 2H), 3.58 (q, *J* = 7.2 Hz, 1H), 2.44 (d, *J* = 7.2 Hz, 2H), 1.84 (dq, *J* = 7.2, 6.6 Hz, 1H), 1.53 (d, *J* = 7.2 Hz, 3H), 0.89 (d, *J* = 6.6 Hz, 6H).

^{13}C NMR (75 MHz, $CDCl_3$) δ 174.59, 148.54, 148.35, 140.95, 138.21, 135.57, 134.31, 129.73, 127.31, 123.56, 46.69, 44.96, 40.95, 30.13, 22.33, 18.34.

HRMS (ESI) calcd $C_{19}H_{24}N_2OH^+$ $[M+H]^+$: 297.1954. Found: 297.1961.

Melting point: 98-100 °C.

Oxazino pyridine **S50** was synthesized as a yellow solid (1.14 g, 70%, containing regio-isomers and diastereomers, the ratio was not determined) from **py-ibu** (reaction time = 24 h) using **GP1**, R_f = 0.3 (pentane/EtOAc 1:1).

^{1}H NMR (300 MHz, $CDCl_3$) δ 7.21 – 7.14 (m, 2H), 7.11 – 7.07 (m, 2H), 6.20 – 6.04 (m, 2H), 5.72 – 5.15 (m, 2H), 4.28 – 3.67 (m, 12H), 3.54 (q, *J* = 7.2 Hz, 1H), 2.43 (dd, *J* = 7.2, 4.5 Hz, 2H), 1.85 – 1.70 (m, 4H), 1.71 (d, *J* = 6.9 Hz, 3H), 0.88 (d, *J* = 6.6 Hz, 6H).

^{13}C NMR (75 MHz, $CDCl_3$) δ 174.2, 174.1, 171.8, 171.7, 164.8, 163.2, 141.1, 141.0, 140.5, 138.6, 129.6, 129.5, 127.3(1), 127.2(6), 124.9, 124.8, 124.0, 123.9, 123.0, 122.7, 116.8, 114.8, 114.6, 100.5, 100.4, 81.2(4), 81.1(9), 53.2, 52.8, 52.7, 52.0, 46.8, 46.7, 45.0, 41.7, 40.7, 30.1, 23.8, 22.3, 18.5, 18.4.

HRMS (ESI) calcd $C_{29}H_{36}N_2O_8Na^+$ $[M+Na]^+$: 563.2358. Found: 563.2364.

Trimethyl 7-(acetoxymethyl)-2-methyl-2*H*,9a*H*-pyrido[2,1-*b*][1,3]oxazine-2,3,4-tricarboxylate and trimethyl 9-(acetoxymethyl)-2-methyl-2*H*,9a*H*-pyrido[2,1-*b*][1,3]oxazine-2,3,4-tricarboxylate (S51)

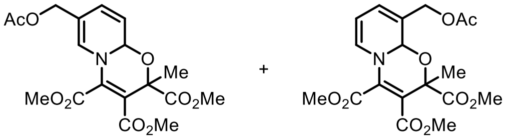

S51

Oxazino pyridine **S51** was a synthesized as a yellow oil (735.4 mg, 93%, containing regioisomers and diastereomers, ratio = 4/4/1/1) from nicotinyl alcohol acetate (2 mmol scale, reaction time = 24 h) using **GP1**, the physical data is in agreement with those reported in our previous work.[4]

^{1}H NMR (400 MHz, $CDCl_3$): δ 6.38 – 6.20 (m, 2H), 5.83 – 5.21 (m, 2H), 4.83 – 4.52 (m, 2H), 3.96 – 3.88 (m, 3H), 3.79 – 3.67 (m, 6H), 2.07 – 2.01 (m, 3H), 1.78 – 1.70 (m, 3H).

Trimethyl 7-(diethylcarbamoyl)-2-methyl-2*H*,9a*H*-pyrido[2,1-*b*][1,3]oxazine-2,3,4-tricarboxylate and Trimethyl 9-(diethylcarbamoyl)-2-methyl-2*H*,9a*H*-pyrido[2,1-*b*][1,3]oxazine-2,3,4-tricarboxylate (S52)

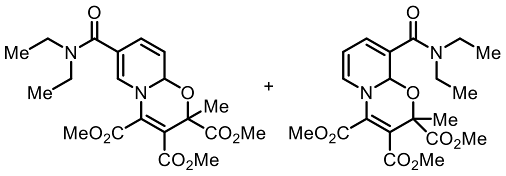

S52

Oxazino pyridine **S52** was synthesized as a yellow solid (836 mg, 99%, containing regioisomers and diastereomers, ratio = 6/3/2/1) from nikethamide (2 mmol scale, reaction time = 48 h) using **GP1**, the physical data is in agreement with those reported in our previous work.[4]

^{1}H NMR (400 MHz, $CDCl_3$) δ 6.75-5.17 (m, 4H), 4.04-3.85 (m, 3H), 3.82-3.66 (m, 6H), 3.56-3.18 (m, 4H), 1.80-1.57 (m, 3H), 1.25-1.10 (m, 6H).

Trimethyl 6-(2-chloro-5-(2-chloro-4-(methylsulfonyl)benzamido)phenyl)-2-methyl-2*H*,9a*H*-pyrido[2,1-*b*][1,3]oxazine-2,3,4-tricarboxylate (S53)

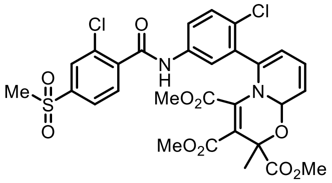

S53

Oxazino pyridine **S53** was synthesized as a yellow solid (1.16 g, 87% yield, containing diastereomers and rotamers, ratio = 8/4/2/1) from vismodegib (2 mmol scale, reaction time = 48 h) using **GP1**, the physical data is in agreement with those reported in our previous work.[4]

^{1}H NMR (400 MHz, $CDCl_3$): δ 8.67 – 8.39 (m, 1H), 8.11 – 7.31 (m, 6H), 6.56 – 6.36 (m, 1H), 5.79 – 5.22 (m, 3H), 3.84 – 3.57 (m, 6H), 3.42 – 3.23 (m, 3H), 3.12 – 3.02 (m, 3H), 1.92 – 1.59 (m, 3H).

Section 4. Condition optimization for the *meta*-difluoromethylation

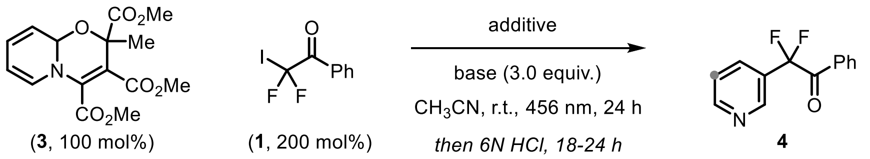

entry	additive	base	solvent	yield (%)	*mono*:*di*
1	PPh_3 (20 mol%)	K_2CO_3	CH_3CN	25	>20:1
2	P(4-CF_3Ph)$_3$ (20 mol%)	K_2CO_3	CH_3CN	25	13:1
3	PPh_3 (20 mol%)	Cs_2CO_3	CH_3CN	21	14:1
4	PPh_3 (20 mol%)	$NaHCO_3$	CH_3CN	28	>20:1
5	PPh_3 (20 mol%)	K_3PO_4	CH_3CN	20	>20:1
6	PPh_3 (20 mol%)	^{t}BuOK	CH_3CN	n.d.	-
7	PPh_3 (20 mol%)	DBU	CH_3CN	trace	-
8	PPh_3 (20 mol%)	DABCO	CH_3CN	21	>20:1
9	PPh_3 (20 mol%)	DMPU)	CH_3CN	23	5:1
10	PPh_3 (20 mol%)	TMEDA	CH_3CN	13	>20:1
11	PPh_3 (20 mol%)	2,6-lutidine	CH_3CN	40	12:1
12	PPh_3 (20 mol%)	KOAc	CH_3CN	35	>20:1
13	PPh_3 (20 mol%)	KOAc (3.0 equiv.) + Et_3N (3.0 equiv.)	CH_3CN	39	20:1
14	PPh_3 (20 mol%)	KOAc (1.0 equiv.) + Et_3N (2.0 equiv.)	CH_3CN	57	12:1
15	KOAc (100 mol%)	Et_3N (2.0 equiv.)	CH_3CN	52	15:1
16	KOAc (100 mol%)	DIPEA (2.0 equiv.)	CH_3CN	44	11:1
17	KOAc (100 mol%)	TEMP (2.0 equiv.)	CH_3CN	65	20:1
18	KOAc (50 mol%)	TMP (2.0 equiv.)	CH_3CN	69	>20:1
19	HOAc (50 mol%)	TMP (2.5 equiv.)	CH_3CN	77(74)	18:1
20	-	TMP (2.0 equiv.)	CH_3CN	64	11:1
21	-	-	CH_3CN	27	6:1
22	PPh_3 (20 mol%)	TMP (2.0 equiv.)	CH_3CN	56	16:1
23	HOAc (50 mol%)	K_2CO_3 (2.0 equiv.)	CH_3CN	35	>20:1
24	HOAc (50 mol%)	DBU (2.0 equiv.)	CH_3CN	19	>20:1
25	HOAc (50 mol%)	TMP (2.0 equiv.)	DCE	64	5:1
26	HOAc (50 mol%)	TMP (2.0 equiv.)	THF	53	8:1
27	HOAc (25 mol%)	TMP (2.25 equiv.)	CH_3CN	67	12:1
28	HOAc (100 mol%)	TMP (3.0 equiv.)	CH_3CN	73	>20:1
29	HOAc (50 mol%)	TMP (3.5 equiv.)	CH_3CN	69	20:1
30	HOAc (50 mol%)	TMP (2.0 equiv.)	CH_3CN	n.d. (no light)	-

Supplementary Figure 3. Condition optimization for the *meta*-difluoromethylation. Oxazino pyridine (0.1 mmol), **1** (0.2 mmol), and CH_3CN (2 mL) under irradiation with 456 nm LEDs (40 W) for 24 h; Work up: added 6N HCl (2 mL) and heated at 60 °C for 24 h, then neutralized with Na_2CO_3 and extracted with EtOAc. Yield was determined by ^{1}H NMR using 1,3,5-mesitylene as the internal standard. Yield in brackets corresponds tp isolated yield.

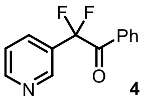

4

Pyridine **4** was obtained as a yellow oil (17.3 mg, 74%) from oxazino pyridine **3** (32.3 mg, 0.1 mmol). R_f = 0.5 (pentane/EtOAc 5:1). The analytical data agree with those reported in the literature.[6]

^{1}H NMR (300 MHz, $CDCl_3$) δ 8.87 (s, 1H), 8.75 (d, *J* = 4.8 Hz, 1H), 8.08 (d, *J* = 7.2 Hz, 2H), 7.90 (d, *J* = 8.7 Hz, 1H), 7.64 (t, *J* = 7.5 Hz, 1H), 7.49 (t, *J* = 7.8 Hz, 2H), 7.42 (dd, *J* = 9.0, 5.1 Hz, 1H).

^{13}C NMR (76 MHz, $CDCl_3$) δ 188.2, 151.8, 147.1 (t, *J* = 6.6 Hz), 134.7, 133.9 (t, *J* = 5.9 Hz), 131.6, 130.3 (t, *J* = 3.2 Hz), 129.1 (t, *J* = 25.2 Hz),128.8, 123.4, 116.4 (t, *J* = 256.2 Hz).

^{19}F NMR (282 MHz, $CDCl_3$) δ -98.01.

Section 5. Proposed mechanism

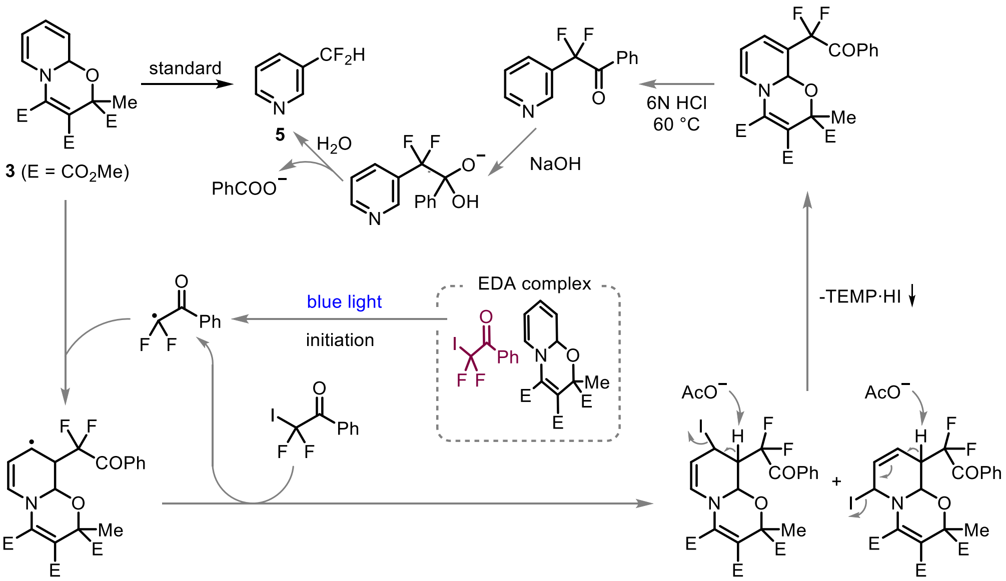

Supplementary Figure 4. Proposed mechanism for *meta*-difluoromethylation.

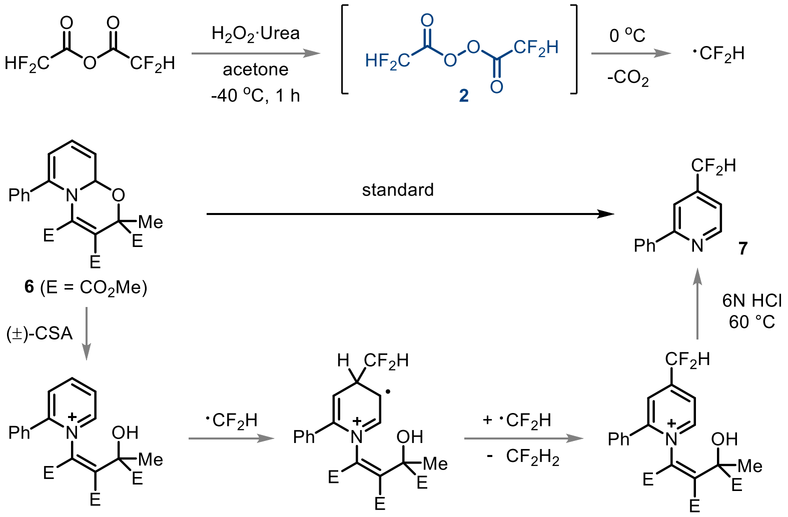

Supplementary Figure 5. Proposed mechanism for *para*-difluoromethylation.

Section 6. General procedure A and analytical data for *meta*-difluoromethylation

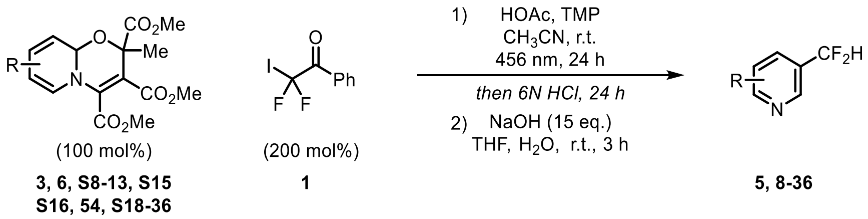

To an oven-dried 10 mL Schlenk tube an oxazino pyridine (**3, 6, S8-13, S15, S16, 53, S18-36**) (0.20 mmol, 100 mol%) was added. The tube was capped and evacuated/refilled with argon for three times. Under an argon flow, CH_3CN (2 mL), 2,2,6,6-tetramethylpiperidine (TMP, 0.50 mmol, 85 µL, 250 mol%), HOAc (0.10 mmol, 5.6 µL, 50 mol%) and 2,2-difluoro-2-iodo-1-phenylethan-1-one **1** (0.40 mmol, 64 µL, 200 mol%) were sequentially added via syringe. The tube was capped again and placed in a photoreactor, stirred and irradiated for 24 h. The temperature was maintained below 30 °C using a fan.

Afterwards, 6N HCl (2 mL) was added to the reaction mixture and the tube was heated at 60 °C for 24 h. The reaction mixture was basified with saturated aqueous Na_2CO_3 solution (30 mL) and extracted with EtOAc (10 mL x 3). The combined organic phase was dried over Na_2SO_4, filtered and concentrated under vacuum. The residue was the dissolved with THF and treated with water (100 µL) and NaOH (3 mmol, 120 mg, 15 equiv.). After stirring at r.t. for 1 h, the reaction mixture was diluted with brine (30 mL) and exacted with Et_2O or EtOAc (10 mL x 3). The combined organic phase was dried over Na_2SO_4, filtered, concentrated and submitted to flash column chromatography (pentane/EtOAc) to yield the *meta*-difluoromethylated pyridine **5**, **8-36**.

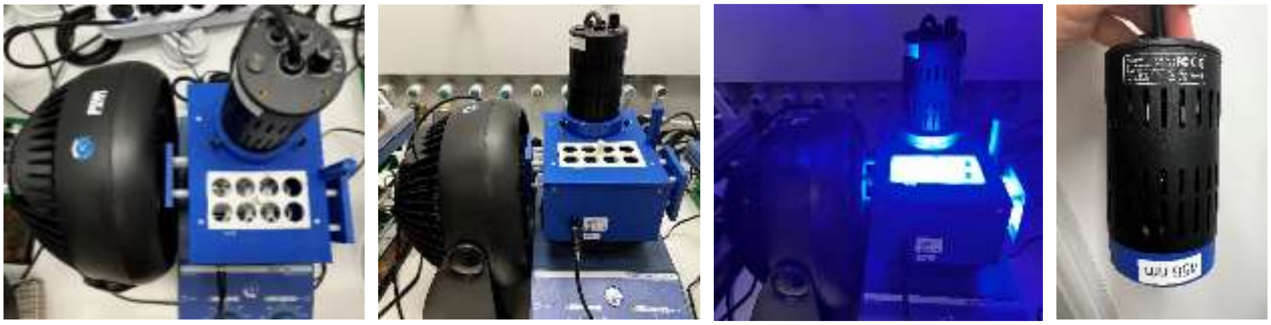

Supplementary Figure 6. Apparatus used for photoreaction and reaction set-up.

The unperfect yields are caused by the following three points:

a) Two times of extraction are conducted, the yields are lost during multi-step operation.

b) Most difluoromethyl pyridines synthesized in this work are volatile, part of the product is lost when concentrating on rota-vap and drying on vacuum pump.

c) Because the acidity of silica-gel, the basic pyridines are partly lost when isolating.

d) In the case of *meta*-substituted pyridines, the corresponding oxazino pyridines are formed as diastereoisomeric mixtures of the two regioisomers. The minor regioisomer was found to be unreactive, and the starting pyridine derived from that regioisomer could be recovered after hydrolysis.

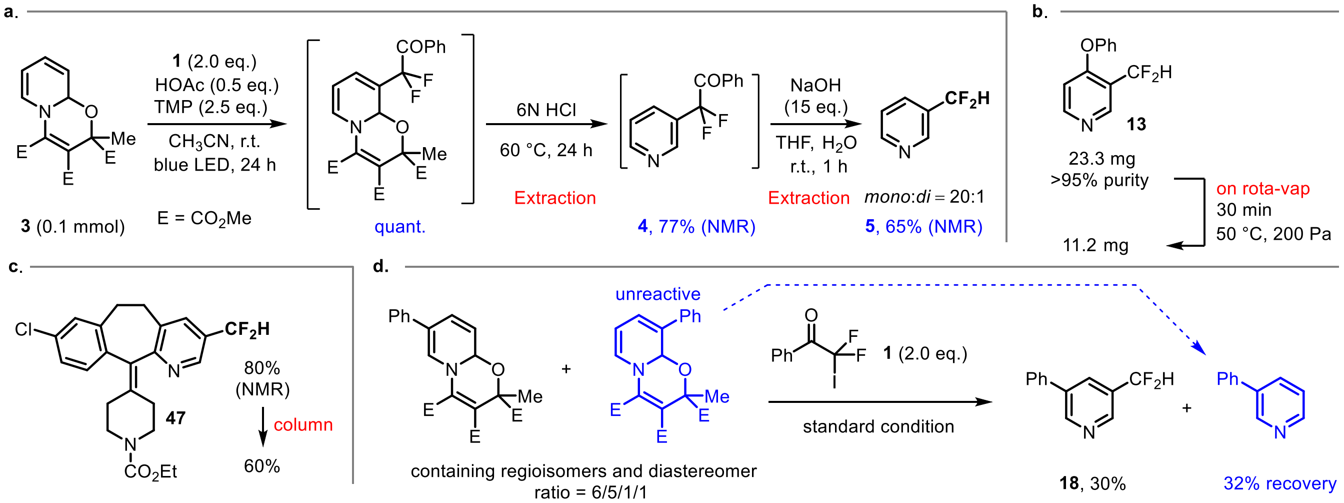

Supplementary Figure 7. Reasons for unperfect yields. **a.** Loss during multi-step operation. **b**. Because of volatility. **c**. Loss on column. **d.** Case of meta-substituted pyridine.

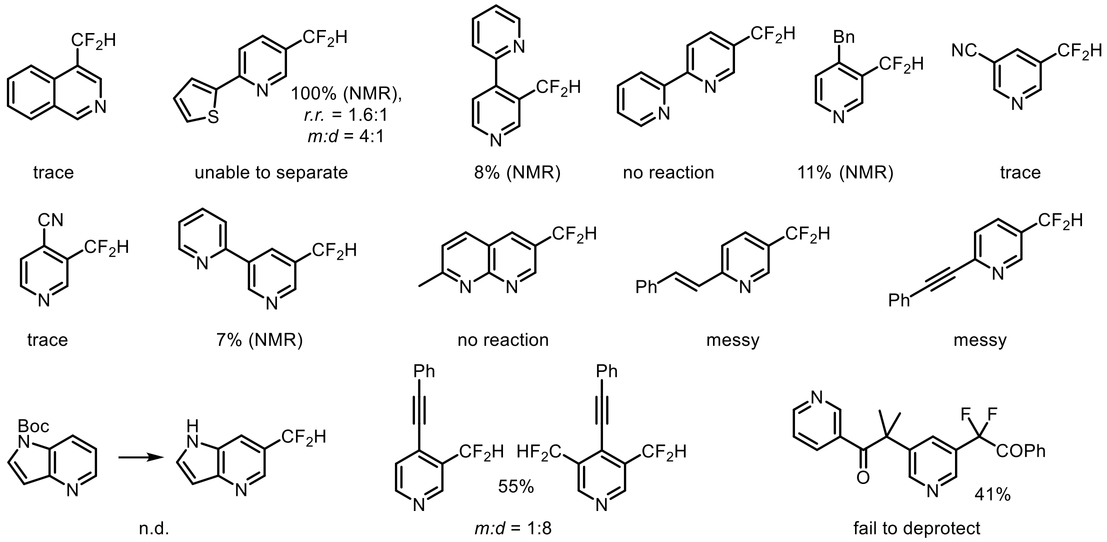

Supplementary Figure 8. Unsuccessful examples.

3-(Difluoromethyl)pyridine (5)

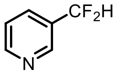

5

Pyridine **5** was obtained as a light yellow oil (68% yield, *mono*:*di* = 17:1) from oxazino pyridine **3** (64.6 mg, 0.20 mmol) using the general procedure A. The yield was determinded by ^{19}F NMR with 1-bromo-4-fluorobenzene as the internal standard. R_f = 0.3 (pentane/EtOAc 2:1). The analytical data agree with those reported in the literature.[7]

^{1}H NMR (300 MHz, $CDCl_3$) δ 8.75 (d, *J* = 6.3 Hz, 2H), 7.85 (d, *J* = 8.4 Hz, 1H), 7.41 (dd, *J* = 8.0, 4.9 Hz, 1H), 6.72 (t, *J* = 55.8 Hz, 1H).

^{13}C NMR (75 MHz, $CDCl_3$) δ 152.0 (t, *J* = 2.2 Hz), 147.3 (t, *J* = 6.7 Hz), 133.3 (t, *J* = 5.5 Hz), 130.1, 123.5, 113.3 (t, *J* = 241.1 Hz).

^{19}F NMR (282 MHz, $CDCl_3$) δ -112.39.

3-(Difluoromethyl)-4-phenylpyridine (8)

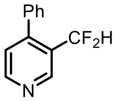

8

Pyridine **8** was obtained as a light yellow oil (27.0 mg, 66%, *mono*:*di* = 15:1) from oxazino pyridine **S8** (79.8 mg, 0.20 mmol) using the general procedure A. R_f = 0.3 (pentane/EtOAc 7:1).

^{1}H NMR (300 MHz, $CDCl_3$) δ 9.00 (s, 1H), 8.73 (d, *J* = 5.1 Hz, 1H), 7.52 – 7.48 (m, 3H), 7.38 – 7.35 (m, 2H), 7.29 (dd, *J* = 5.1, 0.9 Hz, 1H), 6.61 (t, *J* = 54.0 Hz, 1H).

^{13}C NMR (100 MHz, $CDCl_3$) δ 151.4, 148.9, 147.9 (t, *J* = 5.9 Hz), 136.0, 129.1, 128.9, 128.9, 128.8, 124.3, 112.6 (t, *J* = 235.5 Hz).

^{19}F NMR (282 MHz, $CDCl_3$) δ -108.78.

HRMS (EI) calcd $C_{12}H_9NF_2^+$ $[M]^+$: 205.0698. Found: 205.0698.

4-(4-Bromophenyl)-3-(difluoromethyl)pyridine (9)

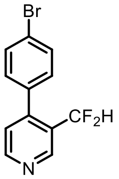

9

Pyridine **9** was obtained as a light yellow oil (39.7 mg, 70%, *mono*:*di* > 20:1) from oxazino pyridine **S9** (95.4 mg, 0.20 mmol) using the general procedure A. R_f = 0.3 (pentane/EtOAc 2:1).

¹H NMR (400 MHz, $CDCl_3$) δ 9.01 (s, 1H), 8.77 (d, *J* = 5.2 Hz, 1H), 7.66 (d, *J* = 8.4 Hz, 2H), 7.37 – 7.18 (m, 3H), 6.60 (t, *J* = 54.0 Hz, 1H).

¹³C NMR (100 MHz, $CDCl_3$) δ 151.6, 148.0 (t, *J* = 6.2 Hz), 147.7 (t, *J* = 5.9 Hz), 134.9, 132.0, 130.4 (d, *J* = 1.2 Hz), 127.1 (t, *J* = 22.3 Hz), 124.1, 123.8, 112.5 (t, *J* = 236.2 Hz).

¹⁹F NMR (282 MHz, $CDCl_3$) δ -108.73.

HRMS (EI) calcd $C_{12}H_8NBrF_2^+$ $[M]^+$: 282.9803. Found: 282.9800.

4-(4-(Benzyloxy)phenyl)-3-(difluoromethyl)pyridine (10)

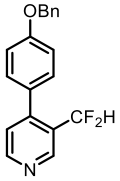

10

Pyridine **10** was obtained as a light yellow solid (34.4 mg, 55%, *mono*:*di* = 11:1) from oxazino pyridine **S10** (101.0 mg, 0.20 mmol) using the general procedure A. R_f = 0.3 (pentane/EtOAc 2:1).

¹H NMR (400 MHz, $CDCl_3$) δ 8.99 (s, 1H), 8.71 (d, *J* = 4.8 Hz, 1H), 7.50 – 7.39 (m, 4H), 7.39 – 7.30 (m, 3H), 7.27 (d, *J* = 5.6 Hz, 1H), 7.09 (d, *J* = 8.8 Hz, 2H), 6.63 (t, *J* = 54.0 Hz, 1H), 5.14 (s, 2H).

¹³C NMR (100 MHz, $CDCl_3$) δ 159.6, 151.3 (t, *J* = 1.7 Hz), 148.5 (t, *J* = 6.1 Hz), 148.0 (t, *J* = 6.0 Hz), 136.5, 130.3, 128.7, 128.4, 128.2, 127.5, 127.2 (t, *J* = 22.2 Hz), 124.3, 115.2, 112.7 (t, *J* = 235.3 Hz), 70.2.

¹⁹F NMR (376 MHz, $CDCl_3$) δ -108.53.

HRMS (EI) calcd $C_{19}H_{15}NOF_2^+$ $[M]^+$: 311.1116. Found: 311.1116.

Melting point: 121-122 °C.

3-(Difluoromethyl)-4-(furan-2-yl)pyridine (11)

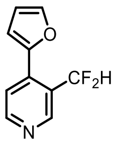

11

Pyridine **11** was obtained as a light yellow oil (13.9 mg, 36%, *mono*:*di* = 3:1) from oxazino pyridine **S11** (77.8 mg, 0.20 mmol) using the general procedure A. R_f = 0.5 (pentane/EtOAc 3:1).

^{1}H NMR (400 MHz, $CDCl_3$) δ 8.97 (s, 1H), 8.71 (d, *J* = 5.2 Hz, 1H), 7.65 (dd, *J* = 1.6, 0.8 Hz, 1H), 7.61 (dd, *J* = 5.2, 0.8 Hz, 1H), 7.12 (t, *J* = 54.4 Hz, 1H), 6.93 (dd, *J* = 3.6, 0.8 Hz, 1H), 6.59 (dd, *J* = 3.6, 2.0 Hz, 1H).

^{13}C NMR (100 MHz, $CDCl_3$) δ 151.7 (t, *J* = 2.1 Hz), 148.7, 148.0 (t, *J* = 8.4 Hz), 145.0, 136.0 (t, *J* = 5.6 Hz), 124.0, 120.1, 113.1 (t, *J* = 2.3 Hz), 112.6 (t, *J* = 236.4 Hz), 112.5.

^{19}F NMR (376 MHz, $CDCl_3$) δ -113.30.

HRMS (EI) calcd $C_{10}H_7NOF_2^+$ $[M]^+$: 195.0490. Found: 195.0491.

4-(Benzo[*b*]thiophen-2-yl)-3-(difluoromethyl)pyridine (12)

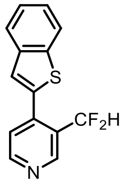

12

Pyridine **12** was obtained as a light yellow solid (30.6 mg, 59%, *mono*:*di* > 20:1) from oxazino pyridine **S12** (91.0 mg, 0.20 mmol) using the general procedure A. R_f = 0.4 (pentane/EtOAc 3:1).

^{1}H NMR (400 MHz, $CDCl_3$) δ 9.06 (s, 1H), 8.77 (d, *J* = 4.8 Hz, 1H), 8.03 – 7.80 (m, 2H), 7.51 – 7.47 (m, 2H), 7.46 – 7.42 (m, 2H), 6.88 (t, *J* = 54.0 Hz, 1H).

^{13}C NMR (100 MHz, $CDCl_3$) δ 151.6 (t, *J* = 1.9 Hz), 148.2 (t, *J* = 6.8 Hz), 141.5 (t, *J* = 5.9 Hz), 140.6, 139.8, 136.3, 128.4, 126.9 (t, *J* = 22.3 Hz), 126.4 (t, *J* = 2.2 Hz), 125.7, 125.1, 124.5, 122.2, 112.4 (t, *J* = 236.1 Hz).

^{19}F NMR (376 MHz, $CDCl_3$) δ -109.38.

HRMS (EI) calcd $C_{14}H_9NSF_2^+$ $[M]^+$: 261.0418. Found: 261.0414.

Melting point: 88-90 °C.

3-(Difluoromethyl)-4-phenoxypyridine (13)

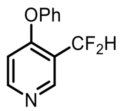

13

Pyridine **13** was obtained as a light yellow oil (23.3 mg, 53%, *mono*:*di* > 20:1) from oxazino pyridine **S13** (83.0 mg, 0.20 mmol) using the general procedure A. R_f = 0.3 (pentane/EtOAc 7:1).

^{1}H NMR (400 MHz, $CDCl_3$) δ 8.79 (s, 1H), 8.49 (d, *J* = 5.6 Hz, 1H), 7.45 (dd, *J* = 8.8, 7.6 Hz, 2H), 7.30 (t, *J* = 7.2 Hz, 1H), 7.12 – 7.10 (m, 2H), 7.10 (t, *J* = 54.4 Hz, 1H), 6.62 (d, *J* = 6.0 Hz, 1H).

^{13}C NMR (100 MHz, $CDCl_3$) δ 162.7 (t, *J* = 5.4 Hz), 153.4 (t, *J* = 1.9 Hz), 153.3, 148.6 (t, *J* = 6.3 Hz), 130.4, 126.1, 120.9, 119.7 (t, *J* = 22.8 Hz), 111.0 (t, *J* = 235.8 Hz), 110.0.

^{19}F NMR (376 MHz, $CDCl_3$) δ -116.30.

HRMS (EI) calcd $C_{12}H_9NOF_2^+$ $[M]^+$: 221.0647. Found: 221.0648.

5-(Difluoromethyl)-2-phenylpyridine (14)

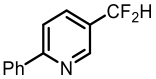

14

Pyridine **14** was obtained as a light yellow solid (25.5 mg, 62%, r.r. = 11:1, *mono*:*di* = 11:1) from oxazino pyridine **6** (79.8 mg, 0.20 mmol) using the general procedure A. R_f = 0.5 (pentane/EtOAc 12:1).

^{1}H NMR (400 MHz, $CDCl_3$) δ 8.82 (d, *J* = 1.2 Hz, 1H), 8.03 (dd, *J* = 8.4, 1.6 Hz, 2H), 7.90 (dd, *J* = 8.4, 1.2 Hz, 1H), 7.82 (d, *J* = 8.4 Hz, 1H), 7.53 – 7.46 (m, 3H), 6.76 (t, *J* = 56.0 Hz, 1H).

^{13}C NMR (100 MHz, $CDCl_3$) δ 159.8, 147.2 (t, *J* = 6.8 Hz), 138.4, 134.1 (t, *J* = 5.2 Hz), 129.7, 128.9, 128.3 (t, *J* = 22.9 Hz), 127.1, 120.2, 113.5 (t, *J* = 237.5 Hz).

^{19}F NMR (376 MHz, $CDCl_3$) δ -111.84.

HRMS (EI) calcd $C_{12}H_9NF_2^+$ $[M]^+$: 205.0698. Found: 205.0696.

Melting point: 54-55 °C.

2-(4-(*Tert*-butyl)phenyl)-5-(difluoromethyl)pyridine (15)

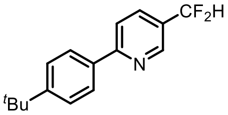

15

Pyridine **15** was obtained as a light yellow solid (20.5 mg, 39%, r.r. = 11:1, *mono*:*di* > 20:1) from oxazino pyridine **S15** (91.1 mg, 0.20 mmol) using the general procedure A. R_f = 0.3 (pentane/EtOAc 12:1).

^{1}H NMR (400 MHz, $CDCl_3$) δ 8.79 (d, *J* = 1.2 Hz, 1H), 7.97 (d, *J* = 8.4 Hz, 2H), 7.89 (d, *J* = 8.4 Hz, 1H), 7.81 (d, *J* = 8.0 Hz, 1H), 7.52 (d, *J* = 8.4 Hz, 2H), 6.75 (t, *J* = 55.6 Hz, 1H), 1.37 (s, 9H).

^{13}C NMR (100 MHz, $CDCl_3$) δ 159.8, 153.0, 147.2 (t, *J* = 6.7 Hz), 135.6, 134.0 (t, *J* = 5.1 Hz), 128.0 (t, *J* = 23.0 Hz), 126.9, 125.9, 119.9, 113.6 (t, *J* = 237.4 Hz), 34.8, 31.2.

^{19}F NMR (376 MHz, $CDCl_3$) δ -111.71.

HRMS (EI) calcd $C_{16}H_{17}NF_2^+$ $[M]^+$: 261.1324. Found: 261.1324.

Melting point: 74-75 °C.

5-(Difluoromethyl)-2-(3-methoxyphenyl)pyridine (16)

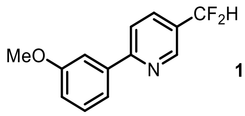

16

Pyridine **16** was obtained as a light yellow oil (20.1 mg, 43%, r.r. = 6:1, *mono*:*di* = 19:1) from oxazino pyridine **S16** (85.9 mg, 0.20 mmol) using the general procedure A. R_f = 0.3 (pentane/EtOAc 12:1).

^{1}H NMR (400 MHz, $CDCl_3$) δ 8.81 (d, *J* = 1.2 Hz, 1H), 7.90 (d, *J* = 8.0 Hz, 1H), 7.82 (d, *J* = 8.4 Hz, 1H), 7.62 (dd, *J* = 2.3, 1.2 Hz, 1H), 7.57 (d, *J* = 7.6 Hz, 1H), 7.41 (t, *J* = 8.0 Hz, 1H), 7.03 – 7.00 (m, 1H), 6.76 (t, *J* = 55.6 Hz, 1H), 3.90 (s, 3H).

^{13}C NMR (100 MHz, $CDCl_3$) δ 160.2, 159.6, 147.1 (t, *J* = 6.8 Hz), 139.8, 134.1 (t, *J* = 5.3 Hz), 129.9, 128.4 (t, *J* = 23.0 Hz), 120.4, 119.5, 115.8, 113.5 (t, *J* = 237.7 Hz), 112.3, 55.4.

^{19}F NMR (376 MHz, $CDCl_3$) δ -111.90.

HRMS (EI) calcd $C_{13}H_{11}NOF_2^+$ $[M]^+$: 235.0803. Found: 235.0809.

Melting point: 52-54 °C.

5-(Difluoromethyl)-2-(2,4-difluorophenyl)pyridine (17)

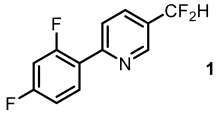

17

Pyridine **17** was obtained as a white solid (31.3 mg, 65%, *mono*:*di* > 20:1) from oxazino pyridine **53** (87.0 mg, 0.20 mmol) using the general procedure A. R_f = 0.3 (pentane/EtOAc 7:1).

^{1}H NMR (400 MHz, $CDCl_3$) δ 8.83 (d, *J* = 1.2 Hz, 1H), 8.06 (td, *J* = 8.8, 6.4 Hz, 1H), 7.96 – 7.77 (m, 2H), 7.03 (dddd, *J* = 8.8, 7.6, 2.4, 1.2 Hz, 1H), 6.94 (ddd, *J* = 11.2, 8.8, 2.4 Hz, 1H), 6.76 (t, *J* = 56.0 Hz, 1H).

^{13}C NMR (100 MHz, $CDCl_3$) δ 163.7 (dd, *J* = 249.8, 12.1 Hz), 160.8 (dd, *J* = 251.7, 12.0 Hz), 154.8, 147.2 (t, *J* = 6.7 Hz), 133.9 (t, *J* = 5.3 Hz), 132.3 (dd, *J* = 9.7, 4.3 Hz), 128.6 (t, *J* = 23.0 Hz), 123.9 (d, *J* = 10.2 Hz), 122.8 (dd, *J* = 11.0, 3.8 Hz), 113.4 (t, *J* = 237.8 Hz), 112.1 (dd, *J* = 21.0, 3.6 Hz), 104.5 (dd, *J* = 26.8, 25.3 Hz).

^{19}F NMR (376 MHz, $CDCl_3$) δ -107.9 (d, *J* = 8.9 Hz), -112.2, -112.3 (d, *J* = 9.0 Hz).

HRMS (EI) calcd $C_{12}H_7NF_4^+$ $[M]^+$: 241.0509. Found: 241.0501.

Melting point: 59-60 °C.

3-(Difluoromethyl)-5-phenylpyridine (18)

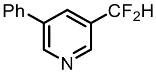

18

Pyridine **18** was obtained as a white solid (12.2 mg, 30%) from oxazino pyridine **S18** (79.8 mg, 0.20 mmol) using the general procedure A. R_f = 0.3 (pentane/EtOAc 5:1).

^{1}H NMR (400 MHz, $CDCl_3$) δ 8.97 (s, 1H), 8.73 (s, 1H), 8.02 (s, 1H), 7.62 – 7.60 (m, 2H), 7.53 – 7.49 (m, 2H), 7.47 – 7.43 (m, 1H), 6.79 (t, *J* = 56.0 Hz, 1H).

^{13}C NMR (100 MHz, $CDCl_3$) δ 150.5 (t, *J* = 2.1 Hz), 145.8 (t, *J* = 6.7 Hz), 136.8, 136.7, 131.5 (t, *J* = 5.4 Hz), 130.1 (t, *J* = 22.6 Hz), 129.3, 128.7, 127.2, 113.4 (t, *J* = 238.3 Hz).

^{19}F NMR (376 MHz, $CDCl_3$) δ -112.35.

HRMS (EI) calcd $C_{12}H_9NF_2^+$ $[M]^+$: 205.0698. Found: 205.0697.

Melting point: 63-65 °C.

3-Benzyl-5-(difluoromethyl)pyridine (19)

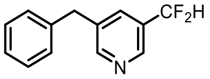

19

Pyridine **19** was obtained as a light yellow oil (22.0 mg, 50%) from oxazino pyridine **S19** (82.6 mg, 0.20 mmol) using the general procedure A. R_f = 0.5 (pentane/EtOAc 5:1).

^{1}H NMR (400 MHz, $CDCl_3$) δ 8.62 – 8.60 (m, 2H), 7.61 (s, 1H), 7.34 – 7.31 (m, 2H), 7.27 – 7.23 (m, 1H), 7.19 – 7.17 (m, 2H), 6.66 (t, *J* = 56.0 Hz, 1H), 4.04 (s, 2H).

^{13}C NMR (100 MHz, $CDCl_3$) δ 152.4 (t, *J* = 2.1 Hz), 145.1 (t, *J* = 6.7 Hz), 138.9, 136.8, 133.3 (t, *J* = 5.4 Hz), 129.9 (t, *J* = 22.8 Hz), 128.9, 128.8, 126.8, 113.4 (t, *J* = 238.0 Hz), 38.8.

^{19}F NMR (376 MHz, $CDCl_3$) δ -112.25.

HRMS (EI) calcd $C_{13}H_{11}NF_2^+$ $[M]^+$: 219.0854. Found: 219.0849.

N-((5-(difluoromethyl)pyridin-3-yl)methyl)furan-2-carboxamide (20)

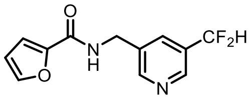

20

Pyridine **20** was obtained as a light yellow oil (15.7 mg, 31%) from oxazino pyridine **S20** (89.2 mg, 0.20 mmol) using the general procedure A. R_f = 0.4 (EtOAc).

^{1}H NMR (300 MHz, $CDCl_3$) δ 8.71 (d, *J* = 12.3 Hz, 2H), 7.84 (s, 1H), 7.45 (dd, *J* = 1.8, 0.9 Hz, 1H), 7.18 (dd, *J* = 3.6, 0.9 Hz, 1H), 6.83 (s, 1H), 6.70 (t, *J* = 55.8 Hz, 1H), 6.53 (dd, *J* = 3.6, 1.8 Hz, 1H), 4.69 (d, *J* = 6.2 Hz, 2H).

^{13}C NMR (100 MHz, $CDCl_3$) δ 158.5, 151.5 (t, *J* = 2.1 Hz), 147.4, 146.4 (t, *J* = 6.6 Hz), 144.2, 134.1, 132.8 (t, *J* = 5.5 Hz), 130.1 (t, *J* = 22.9 Hz), 115.0, 113.2 (t, *J* = 238.4 Hz), 112.36, 40.3.

^{19}F NMR (376 MHz, $CDCl_3$) δ -112.39.

HRMS (EI) calcd $C_{12}H_{10}N_2O_2F_2^+$ $[M]^+$: 252.0705. Found: 252.0704.

5-Bromo-2-((5-(difluoromethyl)pyridin-3-yl)oxy)pyridine (21)

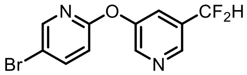

21

Pyridine **21** was obtained as a light yellow oil (20.5 mg, 34%) from oxazino pyridine **S21** (98.8 mg, 0.20 mmol) using the general procedure A. R_f = 0.3 (EtOAc).

^{1}H NMR (300 MHz, $CDCl_3$) δ 8.60 (s, 2H), 8.19 (dd, *J* = 2.4, 0.6 Hz, 1H), 7.85 (dd, *J* = 8.7, 2.4 Hz, 1H), 7.68 (s, 1H), 6.97 (dd, *J* = 8.7, 0.6 Hz, 1H), 6.75 (t, *J* = 55.5 Hz, 1H).

^{13}C NMR (100 MHz, $CDCl_3$) δ 161.2, 148.1, 145.8, 143.1 (t, *J* = 6.8 Hz), 142.5, 132.1, 130.4, 125.8 (t, *J* = 5.6 Hz), 114.7, 114.0 (d, *J* = 238.9 Hz), 113.5.

^{19}F NMR (282 MHz, $CDCl_3$) δ -112.57.

HRMS (EI) calcd $C_{11}H_7N_2OBrF_2^+$ $[M]^+$: 299.9704. Found: 299.9697.

5-(Difluoromethyl)-3-methyl-2-phenylpyridine (22)

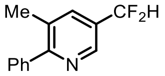

22

Pyridine **22** was obtained as a light yellow oil (24.7 mg, 56%) from oxazino pyridine **S22** (82.6 mg, 0.20 mmol) using the general procedure A. R_f = 0.3 (pentane/EtOAc 15:1).

^{1}H NMR (400 MHz, $CDCl_3$) δ 8.65 (d, *J* = 2.0 Hz, 1H), 7.74 (d, *J* = 1.2 Hz, 1H), 7.54 – 7.12 (m, 2H), 7.49 – 7.42 (m, 3H), 6.74 (t, *J* = 55.6 Hz, 1H), 2.42 (s, 3H).

^{13}C NMR (100 MHz, $CDCl_3$) δ 161.2, 144.3 (t, *J* = 6.7 Hz), 139.7, 135.6 (t, *J* = 5.2 Hz), 131.2, 128.8, 128.4, 128.4 (t, *J* = 22.8 Hz), 128.3, 113.5 (t, *J* = 237.6 Hz), 20.1.

^{19}F NMR (376 MHz, $CDCl_3$) δ -111.75.

HRMS (EI) calcd $C_{13}H_{11}NF_2^+$ $[M]^+$: 219.0854. Found: 219.0810.

3-Bromo-5-(difluoromethyl)-2-phenylpyridine (23)

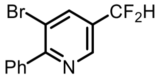

23

Pyridine **23** was obtained as a light yellow oil (26.8 mg, 47%) from oxazino pyridine **S23** (95.4 mg, 0.20 mmol) using the general procedure A. R_f = 0.5 (pentane/EtOAc 12:1).

^{1}H NMR (400 MHz, $CDCl_3$) δ 8.75 (d, *J* = 1.2 Hz, 1H), 8.24 – 8.04 (m, 1H), 7.78 – 7.59 (m, 2H), 7.59 – 7.39 (m, 3H), 6.75 (t, *J* = 55.6 Hz, 1H).

^{13}C NMR (100 MHz, $CDCl_3$) δ 160.5 (t, *J* = 2.0 Hz), 145.4 (t, *J* = 6.5 Hz), 138.7, 138.6 (t, *J* = 5.7 Hz), 129.7 (t, *J* = 23.4 Hz), 129.3, 129.3, 128.1, 119.7, 112.3 (t, *J* = 239.1 Hz).

^{19}F NMR (376 MHz, $CDCl_3$) δ -112.54.

HRMS (EI) calcd $C_{12}H_8NBrF_2^+$ $[M]^+$: 282.9803. Found: 282.9802.

5-(Difluoromethyl)-3-methoxy-2-phenylpyridine (24)

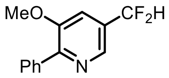

24

Pyridine **24** was obtained as a light yellow oil (23.3 mg, 45%) from oxazino pyridine **S24** (85.8 mg, 0.20 mmol) using the general procedure A. R_f = 0.5 (pentane/EtOAc 6:1).

^{1}H NMR (400 MHz, $CDCl_3$) δ 8.42 (d, *J* = 1.6 Hz, 1H), 7.92 (dd, *J* = 8.0, 1.6 Hz, 2H), 7.54 – 7.31 (m, 4H), 6.75 (t, *J* = 55.6 Hz, 1H), 3.92 (s, 3H).

^{13}C NMR (100 MHz, $CDCl_3$) δ 153.6, 150.8, 138.4 (t, *J* = 7.2 Hz), 136.8, 129.4, 129.3 (t, *J* = 22.7 Hz), 129.0, 128.0, 115.0 (t, *J* = 5.1 Hz), 113.3 (t, *J* = 237.6 Hz), 55.7.

^{19}F NMR (282 MHz, $CDCl_3$) δ -111.36.

HRMS (EI) calcd $C_{13}H_{11}NOF_2^+$ $[M]^+$: 235.0803. Found: 235.0782.

5-(Difluoromethyl)-3-methyl-2-(thiophen-2-yl)pyridine (25)

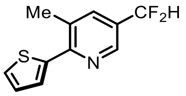

25

Pyridine **25** was obtained as a light yellow solid (26.2 mg, 58%) from oxazino pyridine **S25** (80.6 mg, 0.20 mmol) using the general procedure A. R_f = 0.5 (pentane/EtOAc 9:1).

^{1}H NMR (400 MHz, $CDCl_3$) δ 8.57 (s, 1H), 7.69 (s, 1H), 7.56 (dd, *J* = 4.0, 1.2 Hz, 1H), 7.48 (dd, *J* = 5.2, 1.2 Hz, 1H), 7.16 (dd, *J* = 5.2, 4.0 Hz, 1H), 6.70 (t, *J* = 56.0 Hz, 1H), 2.63 (s, 3H).

^{13}C NMR (100 MHz, $CDCl_3$) δ 153.7, 144.2 (t, *J* = 6.7 Hz), 136.5 (t, *J* = 5.3 Hz), 129.6, 128.5, 127.9, 127.8, 127.7, 127.4, 113.4 (t, *J* = 237.6 Hz), 21.5.

^{19}F NMR (376 MHz, $CDCl_3$) δ -111.82.

HRMS (EI) calcd $C_{11}H_9NSF_2^+$ [M]$^+$: 225.0418. Found: 225.0407.

Melting point: 37-38 °C.

5-(Difluoromethyl)-2,4-diphenylpyridine (26)

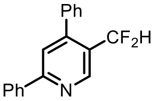

26

Pyridine **26** was obtained as a light yellow oil (30.9 mg, 55%) from oxazino pyridine **S26** (95.0 mg, 0.20 mmol) using the general procedure A. R_f = 0.5 (pentane/EtOAc 9:1).

^{1}H NMR (400 MHz, $CDCl_3$) δ 9.07 (s, 1H), 8.06 (dd, *J* = 8.0, 1.6 Hz, 2H), 7.71 (s, 1H), 7.60 – 7.38 (m, 8H), 6.65 (t, *J* = 54.4 Hz, 1H).

^{13}C NMR (100 MHz, $CDCl_3$) δ 159.1, 149.7 (t, *J* = 6.1 Hz), 147.8 (t, *J* = 5.9 Hz), 138.3, 136.4, 129.7, 129.1, 128.9, 128.9, 128.8, 127.2, 125.6 (t, *J* = 22.4 Hz), 121.1, 112.8 (t, *J* = 235.1 Hz).

^{19}F NMR (282 MHz, $CDCl_3$) δ -108.59.

HRMS (EI) calcd $C_{18}H_{13}NF_2^+$ [M]$^+$: 281.1010. Found: 281.0989.

3-(Difluoromethyl)-5-methyl-2-phenylpyridine (27)

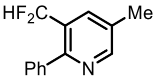

27

Pyridine **27** was obtained as a light yellow oil (21.2 mg, 48%) from oxazino pyridine **S27** (82.6 mg, 0.20 mmol) using the general procedure A. R_f = 0.3 (pentane/EtOAc 15:1).

^{1}H NMR (400 MHz, $CDCl_3$) δ 8.63 (s, 1H), 7.92 (s, 1H), 7.56 – 7.51 (m, 2H), 7.51 – 7.45 (m, 3H), 6.61 (t, *J* = 54.8 Hz, 1H), 2.46 (s, 3H).

^{13}C NMR (100 MHz, $CDCl_3$) δ 155.2 (t, *J* = 6.7 Hz), 152.1 (t, *J* = 2.1 Hz), 137.8, 134.5 (t, *J* = 4.7 Hz), 132.3, 129.3 (t, *J* = 1.1 Hz), 128.9, 128.5, 127.1 (t, *J* = 22.8 Hz), 112.8 (t, *J* = 235.4 Hz), 18.1.

^{19}F NMR (376 MHz, $CDCl_3$) δ -107.79.

HRMS (EI) calcd $C_{13}H_{11}NF_2^+$ $[M]^+$: 219.0854. Found: 219.0815.

3-(Difluoromethyl)-5-methyl-2-(thiophen-2-yl)pyridine (28)

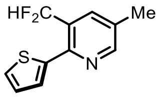

28

Pyridine **28** was obtained as a light yellow solid (35.2 mg, 78%) from oxazino pyridine **S28** (83.8 mg, 0.20 mmol) using the general procedure A. R_f = 0.5 (pentane/EtOAc 9:1).

^{1}H NMR (400 MHz, $CDCl_3$) δ 8.55 (s, 1H), 7.90 (s, 1H), 7.50 (dd, *J* = 5.2, 1.2 Hz, 1H), 7.34 (dd, *J* = 3.6, 1.2 Hz, 1H), 7.14 – 7.06 (m, 1H), 6.84 (t, *J* = 54.4 Hz, 1H), 2.43 (s, 3H).

^{13}C NMR (100 MHz, $CDCl_3$) δ 151.9 (t, *J* = 2.1 Hz), 148.6 (t, *J* = 6.2 Hz), 141.4, 134.6 (t, *J* = 6.0 Hz), 132.1, 128.8, 128.0 (t, *J* = 2.5 Hz), 127.9, 125.6 (d, *J* = 22.4 Hz), 112.4 (t, *J* = 235.9 Hz), 18.2.

^{19}F NMR (282 MHz, $CDCl_3$) δ -109.63.

HRMS (EI) calcd $C_{11}H_9NSF_2^+$ $[M]^+$: 225.0418. Found: 225.0404.

Melting point: 53-55 °C.

3-(Difluoromethyl)-5-methyl-2-(phenylethynyl)pyridine (29)

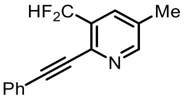

29

Pyridine **29** was obtained as a white solid (26.1 mg, 54%) from oxazino pyridine **S29** (87.4 mg, 0.20 mmol) using the general procedure A. R_f = 0.4 (pentane/EtOAc 5:1).

^{1}H NMR (400 MHz, $CDCl_3$) δ 8.55 (s, 1H), 7.79 (s, 1H), 7.68 – 7.56 (m, 2H), 7.49 – 7.35 (m, 3H), 7.09 (t, *J* = 55.2 Hz, 1H), 2.44 (s, 3H).

^{13}C NMR (100 MHz, $CDCl_3$) δ 152.5, 138.8 (t, *J* = 6.3 Hz), 133.6 (t, *J* = 4.9 Hz), 133.3, 132.0, 131.1 (t, *J* = 22.9 Hz), 129.4, 128.5, 121.6, 112.6 (t, *J* = 237.2 Hz), 94.2, 84.6, 18.5.

^{19}F NMR (282 MHz, $CDCl_3$) δ -114.07.

HRMS (EI) calcd $C_{15}H_{11}NF_2^+$ $[M]^+$: 243.0854. Found: 243.0848.

Melting point: 94-96 °C.

(5-(Difluoromethyl)-6-(thiophen-2-yl)pyridin-3-yl)methanol (30)

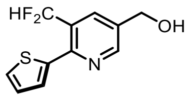

30

Pyridine **30** was obtained as a light yellow oil (23.6 mg, 49%) from oxazino pyridine **S30** (95.4 mg, 0.20 mmol) using the general procedure A. R_f = 0.5 (pentane/EtOAc 1:1).

^{1}H NMR (400 MHz, $CDCl_3$) δ 8.68 (s, 1H), 8.11 (d, *J* = 2.0 Hz, 1H), 7.53 (dd, *J* = 5.2, 1.2 Hz, 1H), 7.37 (dd, *J* = 3.6, 1.2 Hz, 1H), 7.15 (dd, *J* = 5.2, 3.6 Hz, 1H), 6.85 (t, *J* = 54.4 Hz, 1H), 4.81 (s, 2H), 2.16 (s, 1H).

^{13}C NMR (100 MHz, $CDCl_3$) δ 150.3 (t, *J* = 6.2 Hz), 150.0 (t, *J* = 2.1 Hz), 141.0, 134.9, 133.0 (t, *J* = 6.1 Hz), 129.3, 128.5 (t, *J* = 2.6 Hz), 128.0, 126.0 (t, *J* = 22.7 Hz), 112.2 (t, *J* = 236.1 Hz), 62.1.

^{19}F NMR (282 MHz, $CDCl_3$) δ -109.61.

HRMS (EI) calcd $C_{11}H_9NOSF_2^+$ $[M]^+$: 241.0367. Found: 241.0357.

3-(Difluoromethyl)-5-methoxy-2-(thiophen-2-yl)pyridine (31)

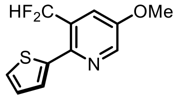

31

Pyridine **31** was obtained as a light yellow oil (29.0 mg, 60%) from oxazino pyridine **S31** (87.0 mg, 0.20 mmol) using the general procedure A. R_f = 0.4 (pentane/EtOAc 9:1).

^{1}H NMR (400 MHz, $CDCl_3$) δ 8.44 (d, *J* = 2.8 Hz, 1H), 7.56 (d, *J* = 3.2 Hz, 1H), 7.48 (dd, *J* = 5.2, 1.2 Hz, 1H), 7.27 (dd, *J* = 3.6, 1.2 Hz, 1H), 7.13 (dd, *J* = 5.2, 3.6 Hz, 1H), 6.83 (t, *J* = 54.4 Hz, 1H), 3.94 (s, 3H).

^{13}C NMR (100 MHz, $CDCl_3$) δ 154.7, 143.7 (t, *J* = 6.4 Hz), 141.1, 140.1 (t, *J* = 2.2 Hz), 128.3, 127.8, 127.3 (t, *J* = 2.4 Hz), 126.8 (t, *J* = 22.3 Hz), 117.3 (t, *J* = 6.3 Hz), 112.2 (t, *J* = 236.3 Hz), 55.9.

^{19}F NMR (282 MHz, $CDCl_3$) δ -109.56.

HRMS (EI) calcd $C_{11}H_9NOSF_2^+$ $[M]^+$: 241.0367. Found: 241.0363.

3-(Difluoromethyl)-5-fluoro-2-(phenylethynyl)pyridine (32)

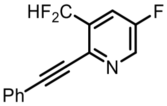

32

Pyridine **32** was obtained as a white solid (25.1 mg, 51%) from oxazino pyridine **S32** (88.3 mg, 0.20 mmol) using the general procedure A. R_f = 0.4 (pentane/EtOAc 12:1).

^{1}H NMR (400 MHz, $CDCl_3$) δ 8.59 (d, *J* = 2.8 Hz, 1H), 7.71 (dd, *J* = 8.0, 2.8 Hz, 1H), 7.62 – 7.60 (m, 2H), 7.43 – 7.37 (m, 3H), 7.09 (td, *J* = 54.8, 1.2 Hz, 1H).

^{13}C NMR (100 MHz, $CDCl_3$) δ 159.6, 157.0, 140.8 (dt, *J* = 24.2, 2.1 Hz), 133.1 (td, *J* = 23.2, 4.1 Hz), 132.0, 129.7, 128.6, 121.2, 120.5 (dt, *J* = 20.8, 5.5 Hz), 111.6 (t, *J* = 238.7 Hz), 94.7 (d, *J* = 1.7 Hz), 83.7.

^{19}F NMR (376 MHz, $CDCl_3$) δ -114.68 (d, J = 1.3 Hz), -123.23.

HRMS (EI) calcd $C_{14}H_8NF_3^+$ $[M]^+$: 247.0603. Found: 247.0601.

Melting point: 63-65 °C.

6-(Difluoromethyl)thieno[3,2-*b*]pyridine (33)

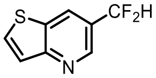

33

Pyridine **33** was obtained as a light yellow oil (23.8 mg, 64%) from oxazino pyridine **S33** (75.8 mg, 0.20 mmol) using the general procedure A. R_f = 0.3 (pentane/EtOAc 3:1).

^{1}H NMR (400 MHz, $CDCl_3$) δ 8.82 (s, 1H), 8.37 (d, *J* = 1.2 Hz, 1H), 7.90 (d, *J* = 5.6 Hz, 1H), 7.63 (dd, *J* = 5.6, 0.8 Hz, 1H), 6.86 (t, *J* = 56.0 Hz, 1H).

^{13}C NMR (100 MHz, $CDCl_3$) δ 157.7, 144.9 (t, *J* = 6.2 Hz), 133.3, 132.7, 128.0 (t, *J* = 6.1 Hz), 125.1 (t, *J* = 22.9 Hz), 125.0, 113.8 (t, *J* = 238.2 Hz).

^{19}F NMR (376 MHz, $CDCl_3$) δ -110.22.

HRMS (EI) calcd $C_8H_5NSF_2^+$ $[M]^+$: 185.0105. Found: 185.0102.

6-(Difluoromethyl)-2-phenylfuro[3,2-b]pyridine (34)

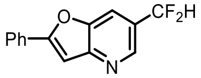

34

Pyridine **34** was obtained as a light yellow solid (25.5 mg, 52%) from oxazino pyridine **S34** (87.8 mg, 0.20 mmol) using the general procedure A. R_f = 0.3 (pentane/EtOAc 3:1).

^{1}H NMR (400 MHz, $CDCl_3$) δ 8.65 (d, *J* = 1.6 Hz, 1H), 7.94 – 7.91 (m, 3H), 7.53 – 7.46 (m, 3H), 7.26 (d, *J* = 0.6 Hz, 1H), 6.85 (t, *J* = 56.0 Hz, 1H).

^{13}C NMR (100 MHz, $CDCl_3$) δ 162.0, 151.4, 147.2, 143.9 (t, *J* = 6.8 Hz), 130.2, 129.2, 129.0, 125.6, 125.5 (t, *J* = 22.9 Hz), 115.0 (t, *J* = 5.7 Hz), 113.9 (t, *J* = 237.9 Hz), 102.3.

^{19}F NMR (376 MHz, $CDCl_3$) δ -109.15.

HRMS (EI) calcd $C_{14}H_9NOF_2^+$ $[M]^+$: 245.0647. Found: 245.0648.

Melting point: 134-135 °C.

3-(Difluoromethyl)-4-methoxyquinoline (35)

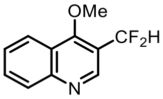

35

Quinoline **35** was obtained as a light yellow oil (14.5 mg, 35%) from oxazino pyridine **S35** (80.6 mg, 0.20 mmol) using the general procedure A. R_f = 0.5 (pentane/EtOAc 2:1).

^{1}H NMR (400 MHz, $CDCl_3$) δ 9.02 (s, 1H), 8.73 (s, 1H), 7.87 – 7.56 (m, 2H), 6.93 (dd, *J* = 7.2, 1.2 Hz, 1H), 6.87 (t, *J* = 55.6 Hz, 1H), 4.03 (s, 3H).

^{13}C NMR (100 MHz, $CDCl_3$) δ 155.5, 149.8, 147.4 (t, *J* = 5.2 Hz), 131.1, 129.1 (t, *J* = 6.9 Hz), 126.2 (t, *J* = 22.8 Hz), 121.5, 119.5, 114.0 (t, *J* = 237.7 Hz), 105.1, 55.9.

^{19}F NMR (376 MHz, $CDCl_3$) δ -110.85.

HRMS (EI) calcd $C_{11}H_9NOF_2^+$ $[M]^+$: 209.0647. Found: 209.0644.

3-(Difluoromethyl)-4-(phenylethynyl)quinoline (36)

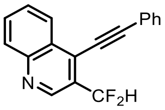

36

Quinoline **36** was obtained as a white solid (23.0 mg, 41%) from oxazino pyridine **S36** (91.0 mg, 0.20 mmol) using the general procedure A. R_f = 0.4 (pentane/EtOAc 9:1).

^{1}H NMR (400 MHz, $CDCl_3$) δ 9.17 (s, 1H), 8.42 (dd, *J* = 8.4, 1.6 Hz, 1H), 8.19 (dd, *J* = 8.4, 1.2 Hz, 1H), 7.98 – 7.80 (m, 1H), 7.76 – 7.67 (m, 3H), 7.56 – 7.42 (m, 3H), 7.31 (t, *J* = 54.7 Hz, 1H).

^{13}C NMR (100 MHz, $CDCl_3$) δ 148.6, 146.5 (t, *J* = 5.2 Hz), 132.0, 131.3, 130.1, 130.0, 129.1 (t, *J* = 6.9 Hz), 128.7, 128.1, 127.0 (t, *J* = 22.9 Hz), 126.5, 126.4, 121.4, 113.4 (t, *J* = 236.3 Hz), 105.0, 80.9.

^{19}F NMR (282 MHz, $CDCl_3$) δ -112.24.

HRMS (EI) calcd $C_{18}H_{11}NF_2^+$ $[M]^+$: 279.0854. Found: 279.0849.

Melting point: 95-97 °C.

Section 7. General procedure B and analytical data for *para*-difluoromethylation

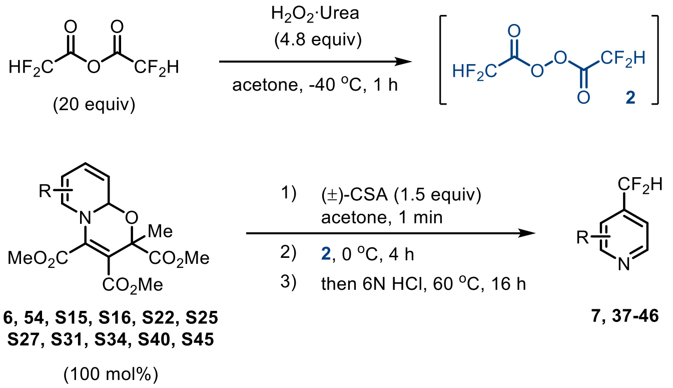

Under argon, difluoroacetic anhydride (0.50 mL, 4.0 mmol, 20 equiv.) was slowly added to a suspension of urea·H_2O_2 (90.4 mg, 0.96 mmol, 4.8 equiv.) in dry acetone (1.0 mL) in a 10 mL Schlenk tube equipped with a magnetic stirring bar at -40 °C, and the mixture was stirred for 1 h at the same temperature.

Another 10 mL oven-dried Schlenk tube equipped with a magnetic stirring bar was charged with oxazino pyridine (0.2 mmol, 1.0 equiv.) and (±)-camphorsulfonic acid ((±)-CSA, 69.7 mg, 0.3 mmol, 1.5 equiv.) and subjected to three cycles of vacuum/argon backfill. Then dry acetone (1.0 mL) was added and the reaction mixture was cooled down to 0 °C using an ice/water bath. Afterwards, the former reaction mixture at -40 °C was transferred to the later tube at the ice/water bath using a glass pipette under the argon flow. The reaction mixture was stirred for 4 h at 0 °C. After completion, 6N HCl (4 mL) was added to the reaction mixture and the tube was heated at 60 °C for 16 h. The mixture was basified with saturated aqueous Na_2CO_3 solution and extracted with EtOAc (10 mL x 3). The combined organic layer was dried (over Na_2SO_4), filtered and concentrated under reduced pressure. The residue was subjected to flash column chromatography over silica gel to give the corresponding product. Where necessary, further purification was accomplished by preparative medium pressure liquid chromatography.

4-(difluoromethyl)-2-phenylpyridine (7)

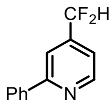

7

Pyridine **7** was obtained as a colorless oil (27.5 mg, 67%) from oxazino pyridine **6** (79.8 mg, 0.20 mmol) using the general procedure B. R_f = 0.4 (pentane/EtOAc 5:1). The analytical data agree with those reported in the literature.[8]

^{1}H NMR (300 MHz, $CDCl_3$) δ 8.82 (d, *J* = 5.1 Hz, 1H), 8.07 – 7.97 (m, 2H), 7.84 (s, 1H), 7.57 – 7.40 (m, 3H), 7.39 – 7.31 (m, 1H), 6.70 (t, *J* = 55.8 Hz, 1H).

^{13}C NMR (75 MHz, $CDCl_3$) δ 158.4, 150.4, 143.0 (t, *J* = 22.9 Hz), 138.5, 129.6, 128.9, 127.0, 118.2 (t, *J*

= 5.7 Hz), 116.6 (t, J = 6.0 Hz), 113.1 (t, J = 239.4 Hz).

^{19}F NMR (282 MHz, $CDCl_3$) δ -115.58.

4-(difluoromethyl)pyridine (37)

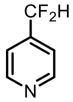

37

Pyridine **37** was prepared from oxazino pyridine **3** (64.6 mg, 0.20 mmol) using the general procedure B. *Yield was determined by ^{19}F NMR due to the high volatility of the product.* The work up step for this reaction was slightly modified. After basifing with saturated aqueous Na_2CO_3 solution, the reaction was extracted by Et_2O (15 mL x 3). The combined organic layer was dried (over Na_2SO_4), filtered and concentrated to 1-2 mL solution under reduced pressure. The residue was added 1-bromo-4-fluorobenzene (10 µL) and α,α,α-trifluorotoluene (7 µL) as internal standard for ^{19}F NMR analysis in $CDCl_3$. The yield was determined respected to 1-bromo-4-fluorobenzene. A yield of 61% was observed (average of two tiials: 60% and 62% yield). The ^{19}F NMR data of Pyridine **37** agrees with those reported in the literature.[9] The 2,4-bis(difluoromethyl)pyridine was detected by GC-MS (M^+ = 179).

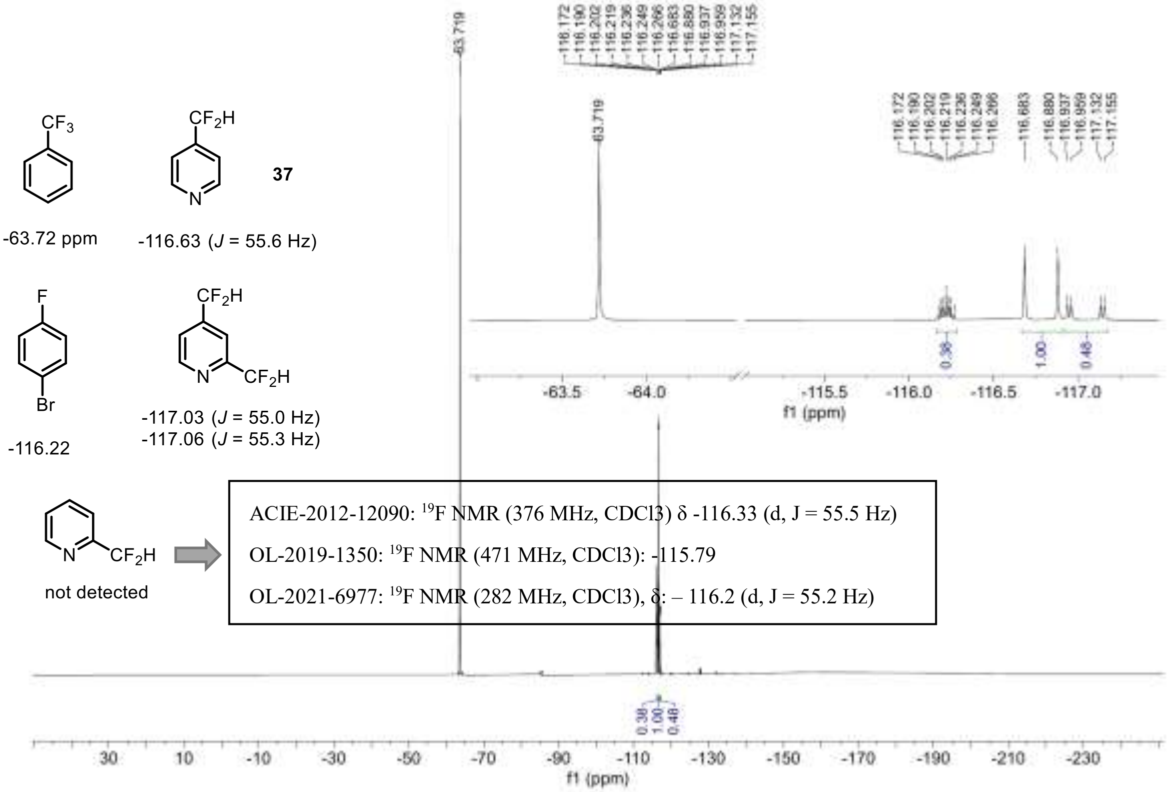

Supplementary Figure 9. ^{19}F NMR (282 MHz, $CDCl_3$) assay for pyridine **37** (trial 1).

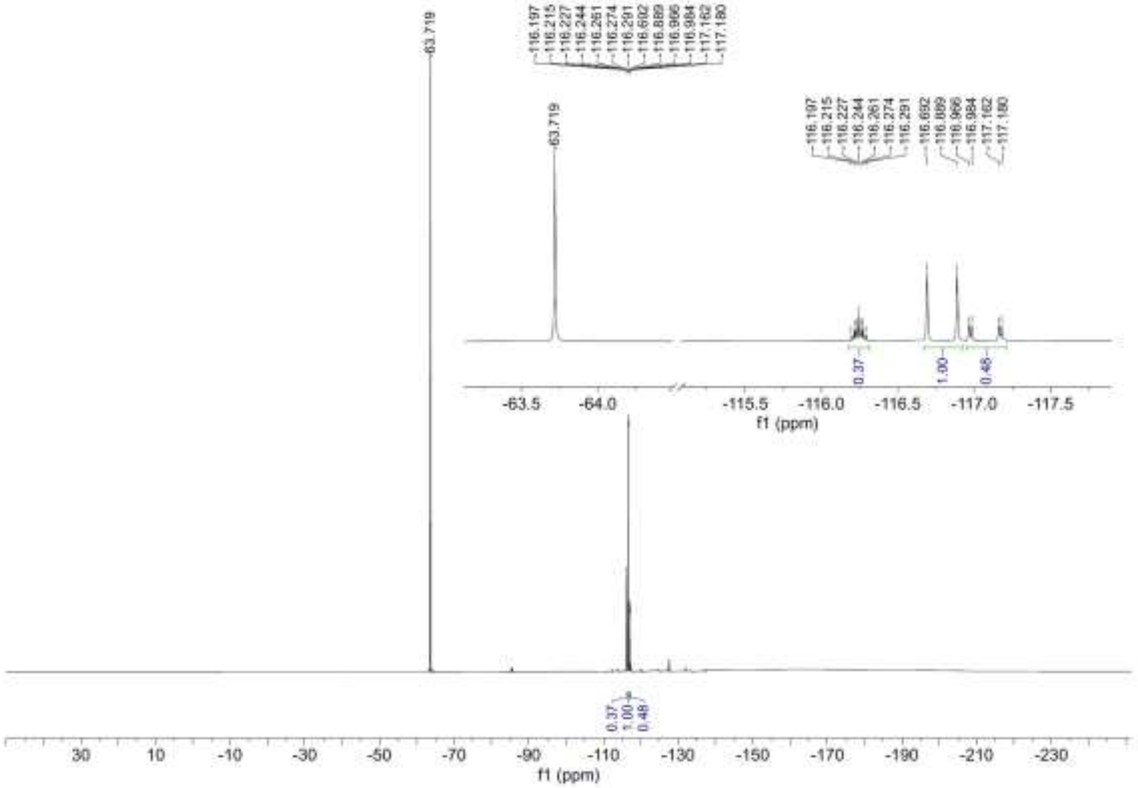

Supplementary Figure 10. ^{19}F NMR (282 MHz, $CDCl_3$) assay for pyridine **37** (trial 2).

2-(4-(*tert*-butyl)phenyl)-4-(difluoromethyl)pyridine (38)

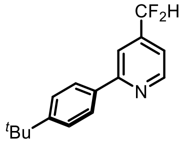

38

Pyridine **38** was obtained as a colorless oil (30.8 mg, 59%) from oxazino pyridine **S15** (91.1 mg, 0.20 mmol) using the general procedure B. R_f = 0.5 (pentane/EtOAc 5:1).

^{1}H NMR (300 MHz, $CDCl_3$) δ 8.83 – 8.74 (m, 1H), 8.00 – 7.90 (m, 2H), 7.84 – 7.78 (m, 1H), 7.57 – 7.48 (m, 2H), 7.35 – 7.29 (m, 1H), 6.69 (t, *J* = 55.8 Hz, 1H), 1.37 (s, 9H).

^{13}C NMR (75 MHz, $CDCl_3$) δ 158.4, 152.8, 150.3, 142.8 (t, *J* = 23.1 Hz), 135.6, 126.7, 125.8, 117.9 (t, *J* = 5.7 Hz), 116.2 (t, *J* = 6.2 Hz), 110.0 (t, *J* = 239.2 Hz), 34.7, 31.2.

^{19}F NMR (282 MHz, $CDCl_3$) δ -115.56.

HRMS (ESI) calcd. for $C_{16}H_{17}NF_2H^+$ $[M+H]^+$: 262.1402. found: 262.1400.

4-(Difluoromethyl)-2-(3-methoxyphenyl)pyridine (39)

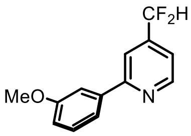

39

Pyridine **39** was obtained as a colorless oil (23.8 mg, 51%) from oxazino pyridine **S16** (85.9 mg, 0.20 mmol) using the general procedure B. R_f = 0.6 (pentane/EtOAc 5:1).

^{1}H NMR (400 MHz, $CDCl_3$) δ 8.81 (dd, *J* = 4.8, 0.8 Hz, 1H), 7.84 – 7.80 (m, 1H), 7.61 (dd, *J* = 2.8, 1.6 Hz, 1H), 7.59 – 7.55 (m, 1H), 7.40 (t, *J* = 8.0 Hz, 1H), 7.35 (dd, *J* = 4.8, 1.2 Hz, 1H), 7.02 – 6.98 (m, 1H), 6.69 (t, *J* = 56.0 Hz, 1H), 3.90 (s, 3H).

^{13}C NMR (100 MHz, $CDCl_3$) δ 160.2, 158.2, 150.3, 143.0 (t, *J* = 23.1 Hz), 139.9, 129.9, 119.3, 118.3 (t, *J* = 5.7 Hz), 116.8 (t, *J* = 6.1 Hz), 115.5, 112.1, 111.9 (t, *J* = 239.4 Hz), 55.4.

^{19}F NMR (282 MHz, $CDCl_3$) δ -115.59.

HRMS (ESI) calcd. for $C_{13}H_{11}NOF_2H^+$ $[M+H]^+$: 236.0882. found: 236.0881.

4-(Difluoromethyl)-2-(2,4-difluorophenyl)pyridine (40)

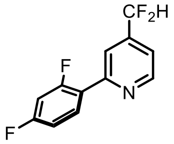

40

Pyridine **40** was obtained as a colorless oil (34.8 mg, 73%) from oxazino pyridine **55** (87.1 mg, 0.20 mmol) using the general procedure B. R_f = 0.3 (pentane/EtOAc 5:1).

^{1}H NMR (300 MHz, $CDCl_3$) δ 8.83 (d, *J* = 5.1 Hz, 1H), 8.04 (td, *J* = 8.7, 6.6 Hz, 1H), 7.87 (d, *J* = 2.1 Hz, 1H), 7.39 (dd, *J* = 5.1, 1.5 Hz, 1H), 7.06 – 6.99 (m, 1H), 6.94 (ddd, *J* = 11.4, 8.7, 2.7 Hz, 1H), 6.69 (t, *J* = 55.5 Hz, 1H).

^{13}C NMR (75 MHz, $CDCl_3$) δ 165.2 (dd, *J* = 250.4, 12.0 Hz), 162.3 (dd, *J* = 251.6, 12.0 Hz), 153.4 (d, *J* = 2.5 Hz), 150.4, 142.8 (t, *J* = 23.2 Hz), 132.2 (dd, *J* = 9.7, 4.3 Hz), 122.9 (dd, *J* = 11.2, 3.8 Hz), 120.4 (dt, *J* = 10.1, 6.1 Hz), 118.5 (t, *J* = 5.7 Hz), 113.0 (t, *J* = 239.5 Hz), 112.1 (dd, *J* = 21.0, 3.6 Hz), 104.5 (dd, *J* = 26.7, 25.1 Hz).

^{19}F NMR (282 MHz, $CDCl_3$) δ -108.07 (d, *J* = 8.7 Hz), -112.57 (d, *J* = 8.7 Hz), -115.57.

HRMS (ESI) calcd. for $C_{12}H_7NF_4H^+$ $[M+H]^+$: 242.0587. found: 242.0586.

4-(Difluoromethyl)-*N*-(3-phenylpropyl)nicotinamide (41)

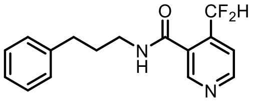 **41**

Pyridine **41** was obtained as a colorless oil (22.1 mg, 38%) from oxazino pyridine **S41** (96.9 mg, 0.20 mmol) using the general procedure B. R_f = 0.5 (EtOAc).

^{1}H NMR (300 MHz, $CDCl_3$) δ 8.80 (s, 1H), 8.67 (s, 1H), 7.65 (d, *J* = 5.0 Hz, 1H), 7.52 – 7.03 (m, 6H), 6.17 (s, 1H), 3.51 (q, *J* = 6.3 Hz, 2H), 2.74 (t, *J* = 7.5 Hz, 2H), 1.99 (p, *J* = 7.2 Hz, 2H).

^{13}C NMR (75 MHz, $CDCl_3$) δ 165.5, 152.3, 147.6, 141.3 (t, *J* = 23.1 Hz), 141.1, 129.7 (t, *J* = 5.0 Hz), 128.6, 128.3, 126.2, 119.8 (t, *J* = 6.8 Hz), 110.9 (t, *J* = 238.2 Hz), 40.0, 33.4, 30.9.

^{19}F NMR (282 MHz, $CDCl_3$) δ -117.27.

HRMS (ESI) calcd. for $C_{16}H_{16}N_2OF_2Na^+$ $[M+Na]^+$: 313.1123. found: 313.1122.

4-(Difluoromethyl)-3-methyl-2-phenylpyridine (42)

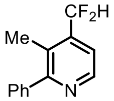 **42**

Pyridine **42** was obtained as a colorless oil (18.0 mg, 41%) from oxazino pyridine **S22** (92.7 mg, 0.20 mmol) using the general procedure B. R_f = 0.4 (pentane/Et_2O 5/1).

^{1}H NMR (300 MHz, $CDCl_3$) δ 8.66 (d, *J* = 5.0 Hz, 1H), 7.49 – 7.46 (m, 4H), 7.45 – 7.40 (m, 2H), 6.82 (t, *J* = 54.7 Hz, 1H), 2.38 (s, 3H).

^{13}C NMR (100 MHz, $CDCl_3$) δ 160.5, 147.3, 141.0 (t, *J* = 21.4 Hz), 139.8, 129.1, 128.4 (t, *J* = 4.6 Hz), 128.3, 128.3, 118.2 (t, *J* = 6.9 Hz), 112.7 (t, *J* = 238.4 Hz), 15.4.

^{19}F NMR (282 MHz, $CDCl_3$) δ -117.14.

HRMS (ESI) calcd. for $C_{13}H_{11}NF_2H^+$ $[M+H]^+$: 220.0932. found: 220.0931.

4-(Difluoromethyl)-3-methyl-2-(thiophen-2-yl)pyridine (43)

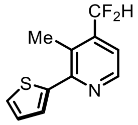 **43**

Pyridine **43** was obtained as a colorless oil (22.5 mg, 50%) from oxazino pyridine **S25** (80.6 mg, 0.20 mmol) using the general procedure B. R_f = 0.3 (pentane/Et_2O 20/1).

^{1}H NMR (400 MHz, $CDCl_3$) δ 8.60 (d, *J* = 4.8 Hz, 1H), 7.46 (dd, *J* = 5.2, 0.8 Hz, 1H), 7.42 (dd, *J* = 3.6, 0.8 Hz, 1H), 7.36 (d, *J* = 5.2 Hz, 1H), 7.14 (dd, *J* = 5.2, 3.6 Hz, 1H), 6.83 (t, *J* = 54.8 Hz, 1H), 2.59 (s, 3H).

^{13}C NMR (100 MHz, $CDCl_3$) δ 153.3, 147.3, 143.4, 141.4 (t, *J* = 21.4 Hz), 128.0, 127.9, 127.8 (t, *J* = 4.6 Hz), 127.5, 118.0 (t, *J* = 7.1 Hz), 112.7 (t, *J* = 238.6 Hz), 15.9.

^{19}F NMR (376 MHz, $CDCl_3$) δ -116.69.

HRMS (ESI) calcd. for $C_{11}H_9NSF_2H^+$ $[M+H]^+$: 226.0496. found: 226.0496.

4-(Difluoromethyl)-5-methyl-2-phenylpyridine (44)

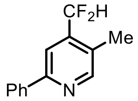

44

Pyridine **44** was obtained as a colorless oil (20.2 mg, 46%) from oxazino pyridine **S27** (82.6 mg, 0.20 mmol) using the general procedure B. R_f = 0.3 (pentane/Et_2O 20/1).

^{1}H NMR (300 MHz, $CDCl_3$) δ 8.59 (s, 1H), 8.04 – 7.95 (m, 2H), 7.83 (s, 1H), 7.53 – 7.37 (m, 3H), 6.78 (t, *J* = 54.6 Hz, 1H), 2.44 (s, 3H).

^{13}C NMR (75 MHz, $CDCl_3$) δ 156.1, 151.8, 140.7 (t, *J* = 21.7 Hz), 138.5, 129.2, 129.0 (t, *J* = 4.3 Hz), 128.8, 126.7, 116.1 (t, *J* = 7.0 Hz), 111.2 (t, *J* = 238.5 Hz), 15.2.

^{19}F NMR (282 MHz, $CDCl_3$) δ -117.33.

HRMS (ESI) calcd. for $C_{13}H_{11}NF_2H^+$ $[M+H]^+$: 220.0932. found: 220.0931.

4-(Difluoromethyl)-5-methoxy-2-(thiophen-2-yl)pyridine (45)

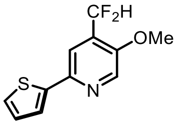

45

Pyridine **45** was obtained as a colorless oil (15.4 mg, 32%) from oxazino pyridine **S31** (87.0 mg, 0.20 mmol) using the general procedure B. R_f = 0.5 (pentane/Et_2O 10/1).

^{1}H NMR (400 MHz, $CDCl_3$) δ 8.33 (d, *J* = 1.2 Hz, 1H), 7.80 (s, 1H), 7.51 (dd, *J* = 4.0, 1.2 Hz, 1H), 7.36 (dd, *J* = 5.2, 1.2 Hz, 1H), 7.10 (dd, *J* = 5.2, 3.6 Hz, 1H), 6.91 (t, *J* = 54.8 Hz, 1H), 3.99 (s, 3H).

^{13}C NMR (100 MHz, $CDCl_3$) δ 151.4 (t, *J* = 5.7 Hz), 146.6, 143.9, 133.5, 130.6 (t, *J* = 22.8 Hz), 128.1, 127.1, 124.0, 115.2 (t, *J* = 5.6 Hz), 110.1 (t, *J* = 236.7 Hz), 56.5.

^{19}F NMR (376 MHz, $CDCl_3$) δ -119.04.

HRMS (ESI) calcd. for $C_{11}H_9NSF_2H^+$ $[M+H]^+$: 242.0446. found: 242.0444.

4-(Difluoromethyl)-5-fluoro-3-methyl-2-phenylpyridine (46)

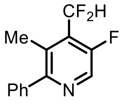

46

Pyridine **46** was obtained as a colorless oil (15.4 mg, 47%) from oxazino pyridine **S46** (86.2 mg, 0.20 mmol) using the general procedure B. R_f = 0.4 (pentane/Et_2O 10/1).

^{1}H NMR (400 MHz, $CDCl_3$) δ 8.49 (s, 1H), 7.51 – 7.38 (m, 5H), 7.08 (t, *J* = 53.6 Hz, 1H), 2.48 (t, *J* = 2.0 Hz, 3H).

^{13}C NMR (100 MHz, $CDCl_3$) δ 157.3 – 157.1 (m), 154.7 (t, *J* = 5.7 Hz), 139.3, 135.6 (d, *J* = 23.9 Hz), 131.1, 129.1, 128.4, 128.4, 127.8 – 127.1 (m), 110.2 (td, *J* = 237.3, 10.1 Hz), 16.3 (td, $J_1 = J_2$ = 2.4 Hz).

^{19}F NMR (376 MHz, $CDCl_3$) δ -116.21 (d, *J* = 3.0 Hz), -137.69.

HRMS (ESI) calcd. for $C_{13}H_{10}NF_3H^+$ $[M+H]^+$: 238.0838. found: 238.0837.

7-(Difluoromethyl)-2-phenylfuro[3,2-*b*]pyridine (47)

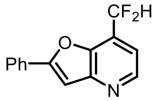

47

Pyridine **47** was obtained as a colorless oil (33.2 mg, 68%) from oxazino pyridine **S34** (87.8 mg, 0.20 mmol) using the general procedure B. R_f = 0.6 (pentane/EtOAc 20/1). The analytical data agree with those reported in the literature.[10]

^{1}H NMR (400 MHz, $CDCl_3$) δ 8.64 (d, *J* = 4.8 Hz, 1H), 7.96 – 7.88 (m, 2H), 7.55 – 7.40 (m, 3H), 7.39 – 7.32 (m, 1H), 7.28 (s, 1H), 7.09 (t, *J* = 54.8 Hz, 1H).

^{13}C NMR (100 MHz, $CDCl_3$) δ 160.8, 150.3, 146.4, 144.4 – 143.7 (m), 130.1, 129.1, 129.0, 125.5, 124.4 (t, *J* = 24.8 Hz), 114.5 (t, *J* = 5.7 Hz), 110.8 (t, *J* = 238.7 Hz), 102.3.

^{19}F NMR (282 MHz, $CDCl_3$) δ -116.05.

Section 8. Analytical data for late-stage difluoromethylation

Meta-difluoromethylated loratadine (48)

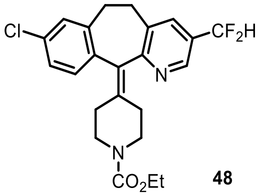

48

Meta-difluoromethylated loratadine **48** was obtained as a light yellow solid (52.1 mg, 60%) from oxazino pyridine **S48** (125.2 mg, 0.20 mmol) using the general procedure A. R_f = 0.3 (pentane/EtOAc 2:1).

^{1}H NMR (400 MHz, $CDCl_3$) δ 8.53 (s, 1H), 7.62 (s, 1H), 7.18 – 7.14 (m, 2H), 7.11 (d, *J* = 8.0 Hz, 1H), 6.65 (t, *J* = 55.6 Hz, 1H), 4.13 (q, *J* = 7.2 Hz, 2H), 3.80 (s, br, 2H), 3.44 – 3.37 (m, 2H), 3.19 – 3.15 (m, 2H), 2.94 – 2.88 (m, 1H), 2.86 – 2.80 (m, 1H), 2.52 – 2.45 (m, 1H), 2.38 – 2.25 (m, 3H), 1.25 (t, *J* = 7.2 Hz, 3H).

^{13}C NMR (100 MHz, $CDCl_3$) δ 159.4, 155.4, 143.9, 139.2, 138.9, 137.0, 135.0, 133.8, 133.3, 133.2, 130.6, 129.0, 128.7 (t, *J* = 22.9 Hz), 126.4, 113.2 (t, *J* = 237.8 Hz), 61.4, 44.7, 44.7, 31.5, 31.4, 30.8, 30.6, 14.6.

^{19}F NMR (376 MHz, $CDCl_3$) δ -111.91, -112.02.

HRMS (ESI) calcd $C_{23}H_{23}N_2O_2ClF_2Na^+$ $[M+Na]^+$: 455.1308. Found: 455.1308.

Melting point: 76-78 °C.

Para-difluoromethylated loratadine (49)

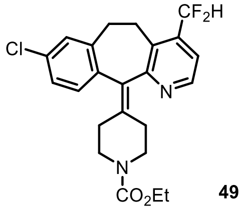

49

*P*ara-difluoromethylated loratadine **49** was obtained as a light yellow solid (49.3 mg, 57%) from oxazino pyridine **S48** (125.2 mg, 0.20 mmol) using the general procedure B. R_f = 0.3 (pentane/EtOAc 2:1). The analytical data agree with those reported in the literature.[10]

^{1}H NMR (400 MHz, $CDCl_3$) δ 8.54 (d, *J* = 5.2 Hz, 1H), 7.32 (d, *J* = 5.2 Hz, 1H), 7.17 – 7.07 (m, 3H), 6.76 (t, *J* = 54.4 Hz, 1H), 4.14 (q, *J* = 7.2 Hz, 2H), 3.85 – 3.75 (m, 2H), 3.48 – 3.29 (m, 2H), 3.26-3.11 (m, 2H), 3.09-2.99 (m, 1H), 2.95 – 2.83 (m, 1H), 2.46 – 2.36 (m, 3H), 2.21 – 2.16 (m, 1H), 1.25 (t, *J* = 7.2 Hz, 3H).

^{13}C NMR (100 MHz, $CDCl_3$) δ 160.3, 155.5, 147.5, 140.0 (t, *J* = 21.7 Hz), 138.6, 137.8, 135.8, 133.7, 133.2, 131.2, 131.1, 129.7, 126.2, 118.8 (t, *J* = 7.2 Hz), 112.7 (t, *J* = 238.9 Hz), 61.4, 44.8, 44.6, 31.6, 30.7, 30.6, 26.3, 14.7.

^{19}F NMR (376 MHz, $CDCl_3$) δ -112.59 – -118.10 (m).

N-((5-(difluoromethyl)pyridin-3-yl)methyl)-2-(4-isobutylphenyl)propanamide (50)

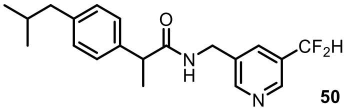

50

Pyridine **50** was obtained as a light yellow oil (24.6 mg, 36%) from oxazino pyridine **S50** (108.0 mg, 0.20 mmol) using the general procedure A. R_f = 0.5 (pentane/EtOAc 1:2).

^{1}H NMR (400 MHz, $CDCl_3$) δ 8.57 (d, *J* = 26.0 Hz, 2H), 7.61 (s, 1H), 7.18 (d, *J* = 8.4 Hz, 2H), 7.12 (d, *J* = 8.0 Hz, 2H), 6.63 (t, *J* = 55.6 Hz, 1H), 5.93 (s, 1H), 4.50 – 4.38 (m, 2H), 3.60 (q, *J* = 7.2 Hz, 1H), 2.44 (d, *J* = 7.2 Hz, 2H), 1.84 (dt, *J* = 13.6, 6.8 Hz, 1H), 1.53 (d, *J* = 7.2 Hz, 3H), 0.88 (d, *J* = 6.8 Hz, 6H).

^{13}C NMR (100 MHz, $CDCl_3$) δ 174.8, 150.7, 145.8 (d, *J* = 6.8 Hz), 141.1, 138.1, 134.7, 132.5 (t, *J* = 5.5 Hz), 129.8, 129.7, 127.2, 113.1 (t, *J* = 238.4 Hz), 46.6, 44.9, 40.6, 30.1, 22.3, 18.3.

^{19}F NMR (376 MHz, $CDCl_3$) δ -112.50.

HRMS (ESI) calcd $C_{20}H_{24}N_2OF_2Na^+$ $[M+Na]^+$: 369.1750. Found: 369.1749.

Meta-difluoromethylated nicotinyl alcohol (51)

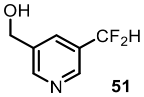

51

Meta-difluoromethylated nicotinyl alcohol **51** was obtained as a light yellow oil (8.0 mg, 25%) from oxazino pyridine **S51** (80.6 mg, 0.20 mmol) using the general procedure A. In the final extraction step, the reaction mixture was extracted with EtOAc (10 mL x 3). R_f = 0.3 (pentane/EtOAc 1:1).

^{1}H NMR (400 MHz, $CDCl_3$) δ 8.68 (d, *J* = 17.2 Hz, 2H), 7.90 (s, 1H), 6.72 (t, *J* = 55.6 Hz, 1H), 4.81 (s, 2H), 2.41 (s, 1H).

^{13}C NMR (100 MHz, $CDCl_3$) δ 150.3 (t, *J* = 2.0 Hz), 146.1 (t, *J* = 6.6 Hz), 136.6, 132.0 (t, *J* = 5.4 Hz), 130.1 (t, *J* = 23.2 Hz), 113.2 (t, *J* = 238.3 Hz), 62.1.

^{19}F NMR (282 MHz, $CDCl_3$) δ -112.38.

HRMS (EI) calcd $C_7H_7NOF_2^+$ $[M]^+$: 159.0490. Found: 159.0489.

Para-difluoromethylated nikethamide (52)

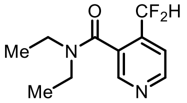

52

Para-difluoromethylated nikethamide **52** was obtained as a colorless oil (15.5 mg, 34%) from oxazino pyridine **S52** (84.5 mg, 0.20 mmol) using the general procedure B. R_f = 0.4 (EtOAc).

^{1}H NMR (300 MHz, $CDCl_3$) δ 8.77 (d, *J* = 5.1 Hz, 1H), 8.60 (s, 1H), 7.58 (d, *J* = 5.1 Hz, 1H), 6.89 (t, *J* = 55.5 Hz, 1H), 3.58 (q, *J* = 7.2 Hz, 2H), 3.20 (q, *J* = 7.2 Hz, 2H), 1.26 (t, *J* = 7.2 Hz, 3H), 1.13 (t, *J* = 7.2 Hz, 3H).

^{13}C NMR (100 MHz, $CDCl_3$) δ 166.4, 150.8, 146.8, 139.6 (t, *J* = 22.9 Hz), 130.7, 119.7 (t, *J* = 6.2 Hz), 111.5 (t, *J* = 239.4 Hz), 43.2, 39.2, 13.8, 12.4.

^{19}F NMR (470 MHz, d_8-toluene, 193 K) δ -110.47, -111.10, -128.12, -128.74.

^{19}F NMR (470 MHz, d_8-toluene, 353 K) δ -117.91.

HRMS (ESI) calcd $C_{11}H_{14}N_2OF_2Na^+$ $[M+Na]^+$: 251.0966. Found: 251.0964.

Para-difluoromethylated vismodegib (53)

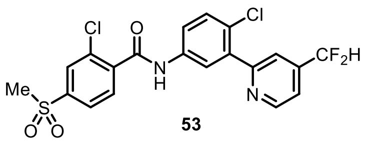

53

Para-difluoromethylated vismodegib **53** was obtained as a colorless oil (31.0 mg, 33%) from oxazino pyridine **S53** (132.9 mg, 0.20 mmol) using the general procedure B. R_f = 0.3 (EtOAc). The analytical data agree with those reported in the literature.[10]

^{1}H NMR (400 MHz, $CDCl_3$) δ 8.96 (s, 1H), 8.67 (d, *J* = 5.2 Hz, 1H), 7.91 – 7.86 (m, 2H), 7.81 (s, 1H), 7.79 (d, *J* = 2.4 Hz, 1H), 7.74 (dd, *J* = 8.0, 2.0 Hz, 1H), 7.68 (d, *J* = 8.00 Hz, 1H), 7.50 (d, *J* = 8.8 Hz, 1H), 7.38 (dd, *J* = 5.2, 1.6 Hz, 1H), 6.71 (t, *J* = 55.6 Hz, 1H), 3.03 (s, 3H).

^{13}C NMR (100 MHz, $CDCl_3$) δ 163.4, 156.8, 149.9, 142.9, 142.6 (t, *J* = 23.5 Hz), 140.3, 138.3, 136.7, 132.2, 131.1, 130.5, 129.0, 127.7, 125.8, 122.8, 121.9, 121.4 (t, *J* = 6.3 Hz), 119.0 (t, *J* = 5.8 Hz), 112.7 (t, *J* = 239.7 Hz), 44.3.

^{19}F NMR (376 MHz, $CDCl_3$) δ -116.05.

Section 9. Synthetic applications

One-pot meta-difluoromethylation of loratadine

***Meta*-difluoromethylated loratadine (48)**

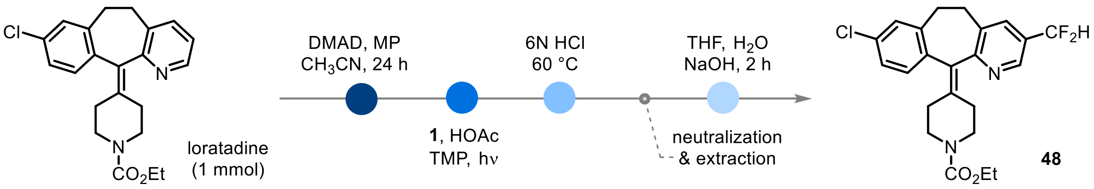

To an oven-dried 50 mL Schlenk tube with a magnetic stirring bar loratadine (383 mg, 1.0 mmol, 100 mol%) was added. The tube was capped and evacuated/refilled with argon for three times. Under an argon flow, methyl pyruvate (122.5 mg, 1.2 mmol, 120 mol%) and acetonitrile (2 mL) were added, followed by dropwise addition of dimethyl acetylenedicarboxylate (170.5 mg, 1.2 mmol, 120 mol%). The reaction mixture was stirred at room temperature under argon for 24 h, and the mixture became a dark homogeneous solution. Still under an argon flow, the CH_3CN (8 mL), 2,2,6,6-tetramethylpiperidine (TMP, 2.5 mmol, 425 µL, 250 mol%), HOAc (0.5 mmol, 28 µL, 50 mol%) and 2,2-difluoro-2-iodo-1-phenylethan-1-one **1** (2 mmol, 320 µL, 200 mol%) were sequentially added via syringe. The tube was capped again and placed in a photoreactor, stirred and irradiated for 24 h. The temperature was maintained below 30 °C using a fan.

Afterwards, 6N HCl (10 mL) was added to the reaction mixture and the vial was heated at 60 °C for 24 h. The reaction mixture was basified with saturated Na_2CO_3 (150 mL) aqueous solution and extracted with EtOAc (50 mL x 3). The combined organic phase was dried over Na_2SO_4, filtered and concentrated under vacuum. The residue was the dissolved with THF and treated with water (100 µL) and NaOH (15 mmol, 600 mg, 15 equiv.). After stirring at r.t. for 1 h, the reaction mixture was diluted with brine (100 mL) and exacted with EtOAc (50 mL x 3). The combined organic phase was dried over Na_2SO_4, filtered, concentrated and submitted to flash column chromatography (pentane/EtOAc 2:1) to obtain the *meta*-difluoromethylated loratadine **48** (221.0 mg, 51% yield).

One-pot para-difluoromethylation *of loratadine*

***Para*-difluoromethylated loratadine (49)**

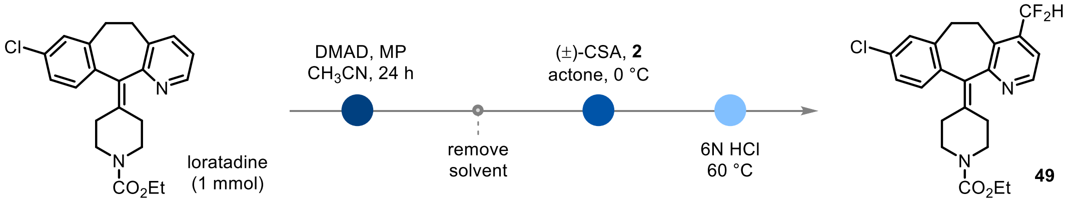

To an oven-dried 50 mL Schlenk tube with a magnetic stirring bar loratadine (383 mg, 1.0 mmol, 100 mol %) was added. The tube was capped and evacuated/refilled with argon for three times. Under an argon flow, methyl pyruvate (122.5 mg, 1.2 mmol, 120 mol%), acetonitrile (2 mL) were added, followed by dropwise addition of dimethyl acetylenedicarboxylate (170.5 mg, 1.2 mmol, 120 mol%). The reaction mixture was stirred at room temperature under argon for 24 h, and the mixture became a dark homogeneous solution. The solvent was removed by a vacuum pump and the tube was refilled with argon.

Then, (±)-CSA (348.5.7 mg, 1.5 mmol, 1.5 equiv.) and acetone (5.0 mL) were added and the reaction mixture was cooled down to 0 °C using an ice/water bath. Afterwards, the difluoromethyl reagent **2** (4.8 equiv. in 5.0 mL acetone) which was *in situ* prepared from difluoroacetic anhydride and urea·H_2O_2 at -40 °C, was transferred into the reaction mixture. The reaction mixture was stirred for 4 h at 0 °C. When completed, 6 N HCl (20 mL) was added to the reaction mixture and the tube was heated at 60 °C for 16 h. The mixture was basified with saturated Na_2CO_3 aqueous solution and extracted with EtOAc (15 mL x 3). The combined organic phase was dried over Na_2SO_4, filtered, concentrated under reduced pressure. The residue was subjected to flash column chromatography over silica gel to give the corresponding product **49** (233.3 mg, 54% yield).

Consecutive meta- or/and para-C–H functionalization of a ligand

3-Chloro-5-(difluoromethyl)-2-(2,4-difluorophenyl)pyridine (55)

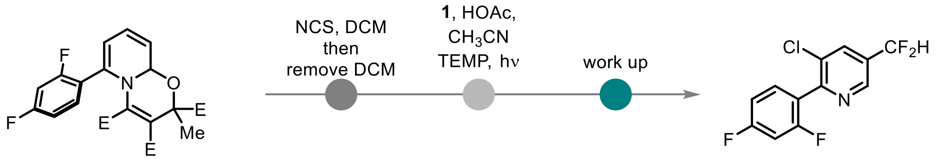

54 (E = CO_2Me) **55**

To a 10 mL oven-dried Schlenk tube equipped with a magnetic stirring bar oxazino pyridine **54** (87 mg, 0.20 mmol, 100 mol%) and NCS (32 mg, 0.24 mmol, 120 mol%) were added, followed by addition of DCM (2 mL). The reaction mixture was stirred at 40 °C for 24 h under air atmosphere. The solvent was then removed by a vacuum pump and the tube was refilled with argon. Afterwards, the reaction mixture was subjected to *meta*-difluoromethylation using the general procedure A. After purification by flash chromatography (pentane/EtOAc = 15/1), the desired *meta*,*meta*-functionalized pyridine **55** was obtained as a colorless oil (20.8 mg, 38% overall yield based on **54**). R_f = 0.4 (pentane/EtOAc= 15:1).

^{1}H NMR (400 MHz, $CDCl_3$) δ 8.73 (d, *J* = 1.6 Hz, 1H), 7.98 (s, 1H), 7.46 (td, *J* = 8.4, 6.0 Hz, 1H), 7.08 – 6.99 (m, 1H), 6.95 (ddd, *J* = 10.0, 8.8, 2.4 Hz, 1H), 6.77 (t, *J* = 55.6 Hz, 1H).

^{13}C NMR (100 MHz, $CDCl_3$) δ 165.0 (d, *J* = 11.7 Hz), 162.6 (d, *J* = 11.6 Hz), 161.3 (d, *J* = 12.2 Hz), 158.8 (d, *J* = 12.1 Hz), 154.5, 144.8 (t, *J* = 6.5 Hz), 134.8 (t, *J* = 5.6 Hz), 132.1 (dd, *J* = 9.9, 4.6 Hz), 130.6 (t, *J* = 23.2 Hz), 112.3 (t, *J* = 239.5 Hz), 111.7 (dd, *J* = 21.5, 3.7 Hz), 104.3 (t, *J* = 25.3 Hz).

^{19}F NMR (376 MHz, $CDCl_3$) δ -107.50 (d, *J* = 9.0 Hz), -108.66 (d, *J* = 9.0 Hz), -112.95.

HRMS (EI) calcd $C_{12}H_6NClF_4^+$ $[M]^+$: 275.0119. Found: 275.0112.

3-Chloro-4-(difluoromethyl)-2-(2,4-difluorophenyl)pyridine (56)

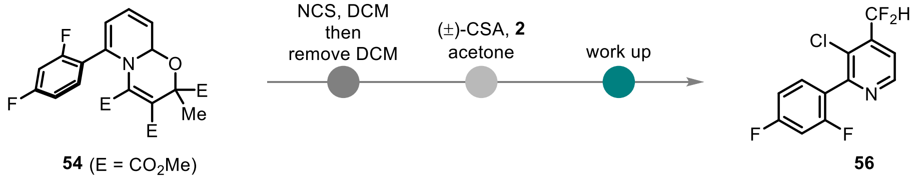

To a 10 mL oven-dried Schlenk tube equipped with a magnetic stirring bar oxazino pyridine **54** (87 mg, 0.2 mmol, 100 mol%) and NCS (40 mg, 0.3 mmol, 120 mol%) were added, followed by addition of DCM (2 mL). The reaction mixture was stirred at 40 °C for 24 h under air atmosphere. The solvent was then removed by a vacuum pump and the tube was refilled with argon. Afterwards, the reaction mixture was subjected to *para*-difluoromethylation using the general procedure B. After purification by flash chromatography (pentane/EtOAc = 15/1), the desired meta,para-functionalized pyridine **56** was obtained as a colorless oil (17.7 mg, 32% overall yield based on **54**). R_f = 0.33 (pentane/EtOAc= 10:1).

^{1}H NMR (300 MHz, $CDCl_3$) δ 8.74 (d, *J* = 5.1 Hz, 1H), 7.62 (d, *J* = 4.8 Hz, 1H), 7.45 (td, *J* = 8.1, 6.3 Hz, 1H), 7.20 – 6.69 (m, 3H).

^{13}C NMR (75 MHz, $CDCl_3$) δ 163.7 (dd, *J* = 249.9, 11.7 Hz), 160.0 (dd, *J* = 250.5, 12.0 Hz), 153.5, 148.2, 140.5 (t, *J* = 23.8 Hz), 132.2 (dd, *J* = 9.8, 4.6 Hz), 129.8 (t, *J* = 6.0 Hz), 122.0 (dd, *J* = 15.2, 3.7 Hz), 120.2 (t, *J* = 5.7 Hz), 111.7 (dd, *J* = 21.5, 3.8 Hz), 110.8 (t, *J* = 239.1 Hz), 104.3 (t, *J* = 25.4 Hz).

^{19}F NMR (282 MHz, $CDCl_3$) δ -107.6 (d, *J* = 9.0 Hz), -108.8 (d, *J* = 9.3 Hz), -118.60.

HRMS calcd $C_{12}H_6NClF_4H^+$ $[M+H]^+$: 276.0198. Found: 276.0198.

4,5-Bis(difluoromethyl)-2-(2,4-difluorophenyl)pyridine (57)

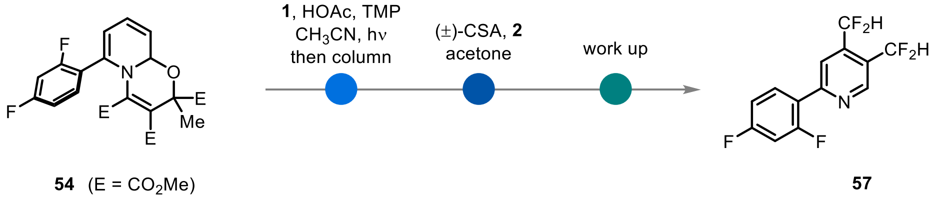

To an oven-dried 10 mL Schlenk tube oxazino pyridine **54** (87 mg, 0.2 mmol, 100 mol%) were added. The tube was capped and evacuated/refilled with argon for three times. Under an argon flow, the CH_3CN (2 mL), 2,2,6,6-tetramethylpiperidine (TMP, 0.5 mmol, 85 µL, 250 mol%), HOAc (0.1 mmol, 5.6 µL, 50 mol%) and 2,2-difluoro-2-iodo-1-phenylethan-1-one **1** (0.4 mmol, 64 µL, 200 mol%) were sequentially added via syringe. The tube was capped again and placed in a photoreactor, stirred and irradiated for 24 h. The temperature was maintained below 30 °C using a fan. Afterwards, the solvent was removed on a rotavapor and the mixture was submitted to flash chromatography (pentane/EtOAc = 2/1) to obtain the intermediate.

Another 10 mL oven-dried Schlenk tube equipped with a magnetic stirring bar was charged with the obtained intermediate and (±)-CSA (69.7 mg, 0.3 mmol, 1.5 equiv.). After being evacuated/refilled with

argon for three times, dry acetone (1.0 mL) was added and the reaction mixture was cooled down to 0 ºC using an ice/water bath. Afterwards, the difluoromethyl reagent **2** (4.8 equiv. in 1.0 mL acetone) which was *in situ* prepared from difluoroacetic anhydride and urea·H_2O_2 at -40 ºC, was transferred into the reaction mixture under an argon flow. The resulting reaction mixture was stirred for 4 h at 0 ºC. When completed, 6 N HCl (4 mL) was added to the reaction mixture and the tube was heated at 60 ºC for 16 h. The mixture was basified with saturated Na_2CO_3 aqueous solution and extracted with EtOAc (10 mL x 3).

The combined organic phase was dried over Na_2SO_4, filtered and concentrated under vacuum. The residue was then dissolved with THF and treated with water (100 µL) and NaOH (3 mmol, 120 mg, 15 equiv.). After stirring at r.t. for 1 h, the reaction mixture was diluted with brine (30 mL) and exacted with Et_2O (10 mL x 3). The combined organic phase was dried over Na_2SO_4, filtered, concentrated and subjected to flash column chromatography (pentane/EtOAc= 20/1) to obtain the double-difluoromethylated pyridine **57** was obtained as a colorless oil. (18.1 mg, 31% overall yield based on **54**).

^{1}H{^{19}F} NMR (599 MHz, $CDCl_3$) δ 8.97 (t, *J* = 0.6 Hz, 1H), 8.11 (d, *J* = 9.0 Hz, 1H), 8.06 (t, *J* = 0.6 Hz, 1H), 7.05 (dd, *J* = 8.4, 2.4 Hz, 1H), 6.99 (d, *J* = 0.6 Hz, 1H), 6.97 – 6.95 (m, 2H).

^{13}C{^{1}H, ^{19}F} NMR (151 MHz, $CDCl_3$) δ 164.1, 161.0, 156.0, 148.0(3) – 148.0(1) (m), 140.7, 132.4, 125.1, 122.1, 120.4, 112.5, 112.4, 111.6, 104.7.

^{19}F{^{19}F } NMR (564 MHz, $CDCl_3$) δ -106.60 (d, *J* = 9.55 Hz), -111.73 (d, *J* = 9.55 Hz), -112.16 – 112.18 (m), -115.26 – 115.28 (m).

HRMS calcd $C_{13}H_7NF_6^+$ $[M]^+$: 291.0477. Found: 291.0472.

Section 10. NMR spectra.

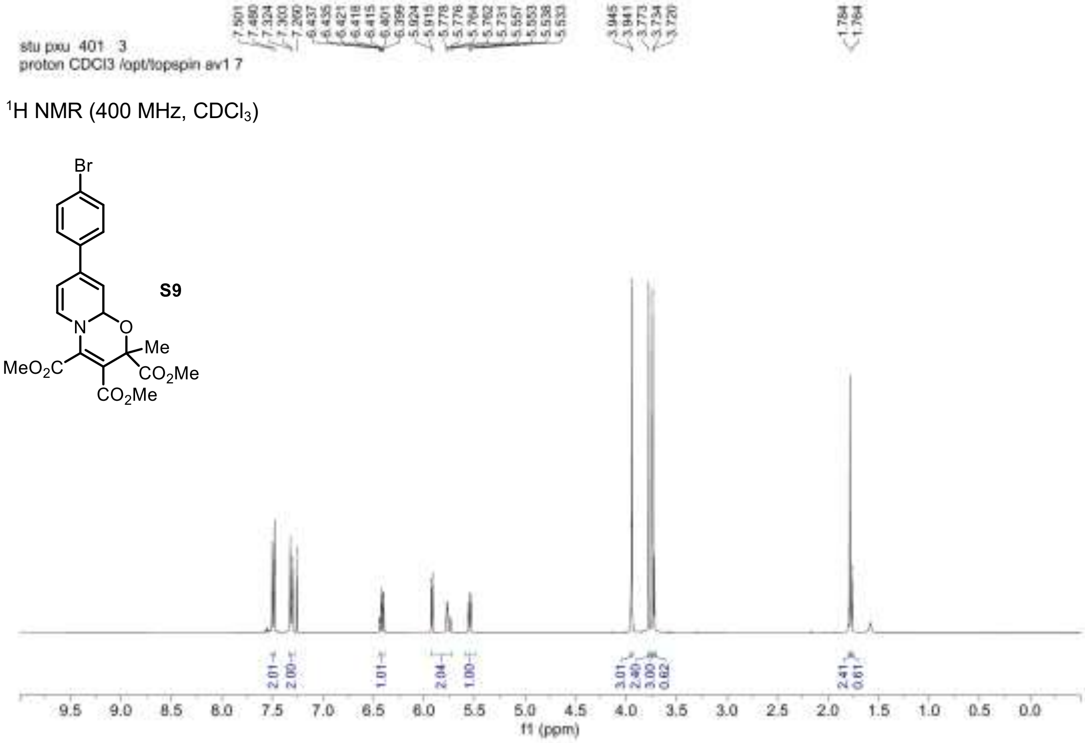

Supplementary Figure 11. ^{1}H NMR (400 MHz, $CDCl_3$) spectrum of compound **S9**

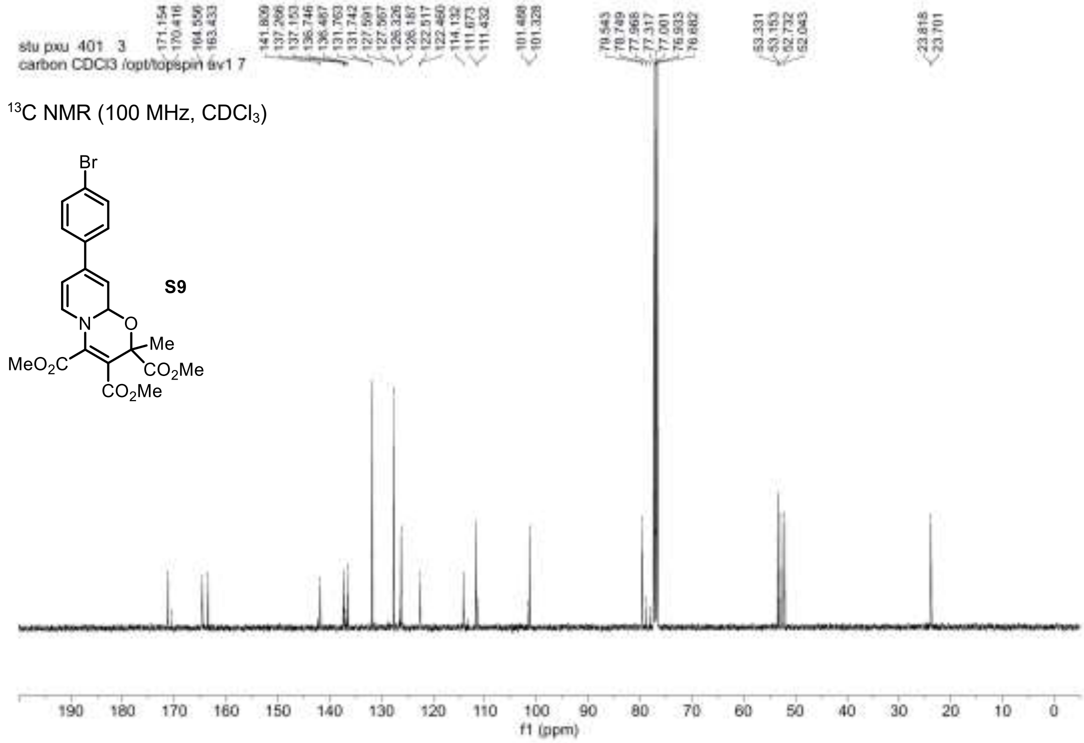

Supplementary Figure 12. ^{13}C NMR (100 MHz, $CDCl_3$) spectrum of compound **S9**

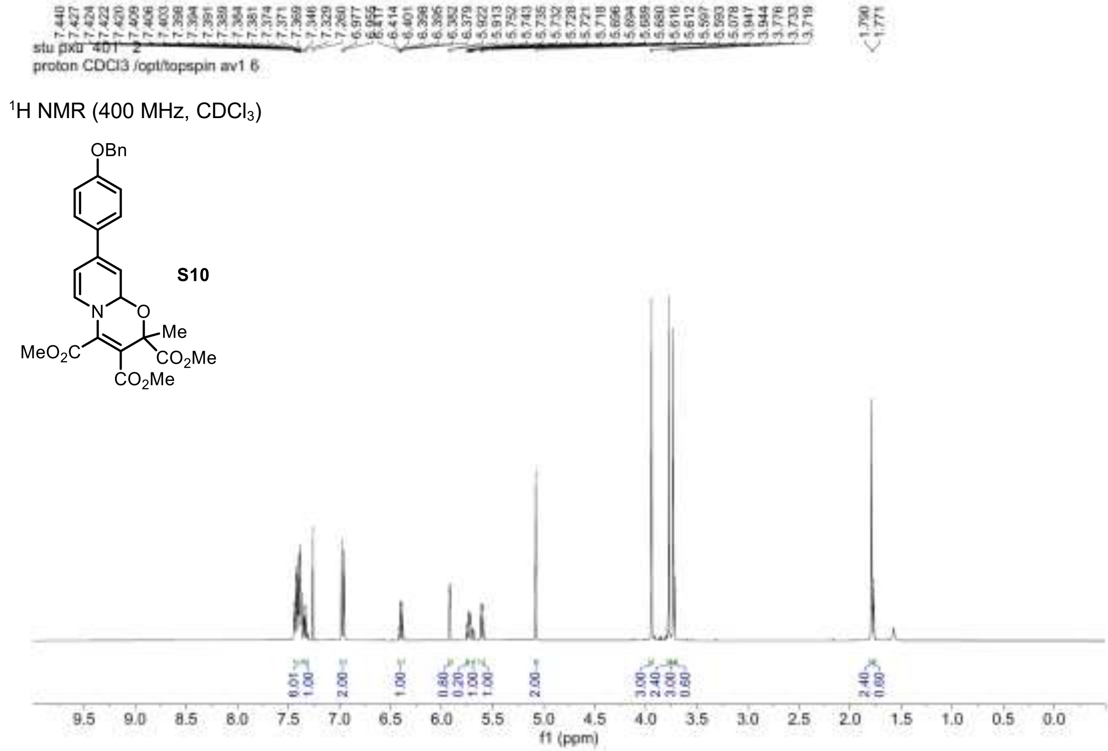

Supplementary Figure 13. ^{1}H NMR (400 MHz, $CDCl_3$) spectrum of compound **S10**

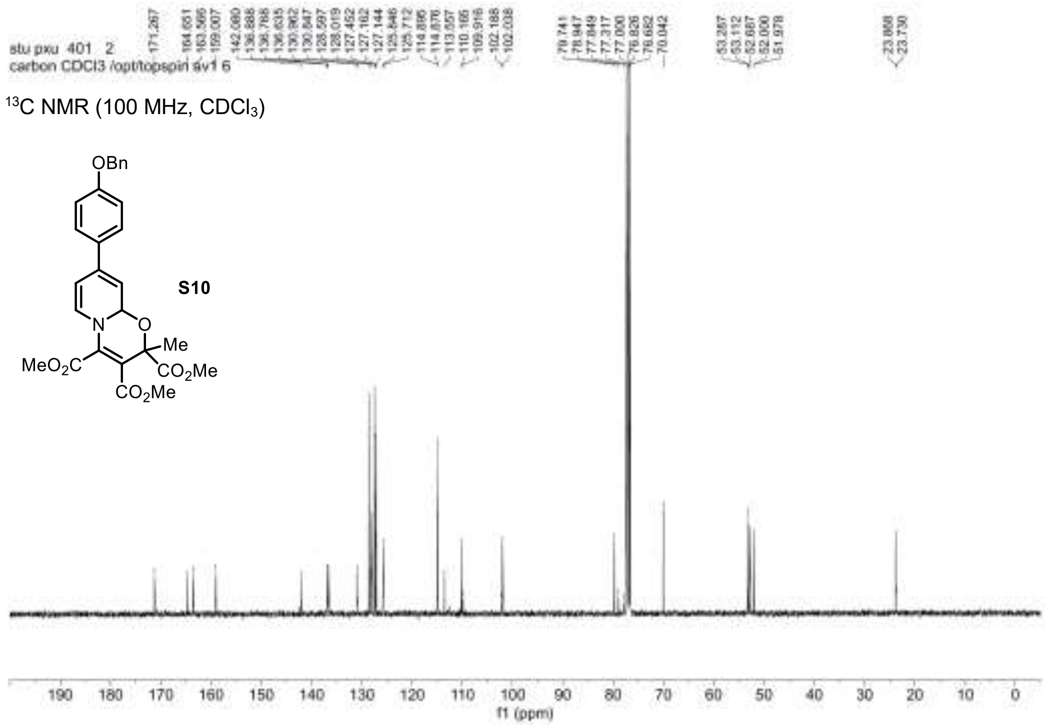

Supplementary Figure 14. ^{13}C NMR (100 MHz, $CDCl_3$) spectrum of compound **S10**

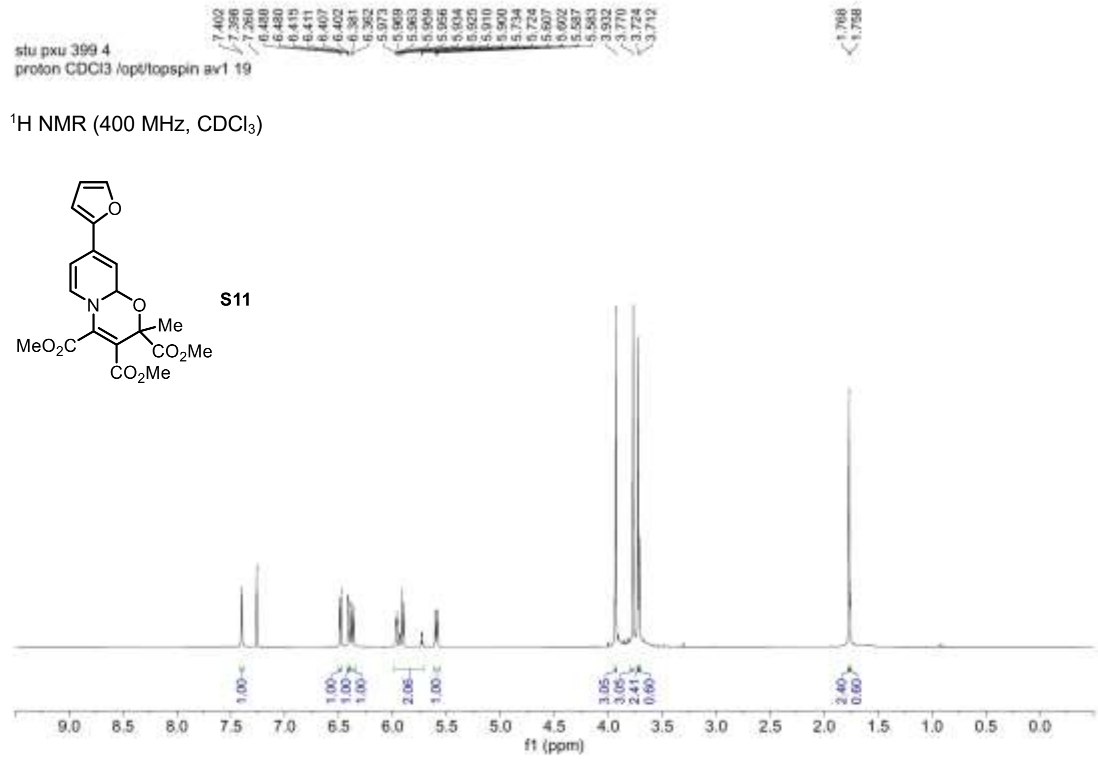

Supplementary Figure 15. ^{1}H NMR (400 MHz, $CDCl_3$) spectrum of compound **S11**

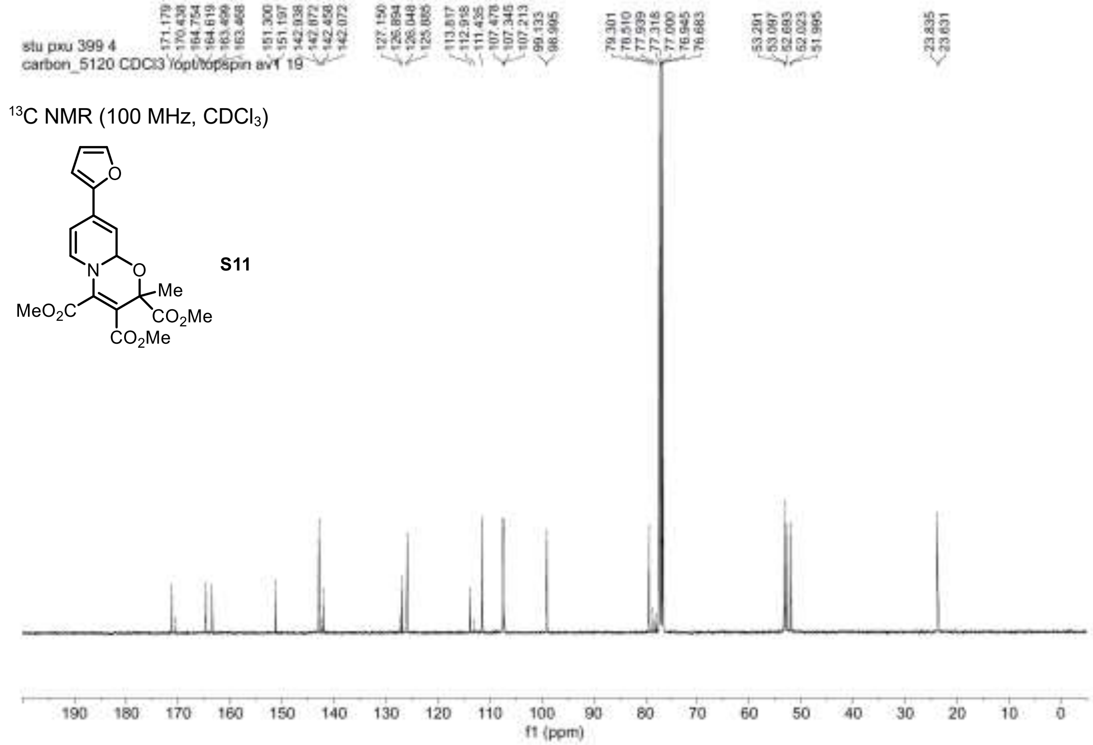

Supplementary Figure 16. ^{13}C NMR (100 MHz, $CDCl_3$) spectrum of compound **S11**

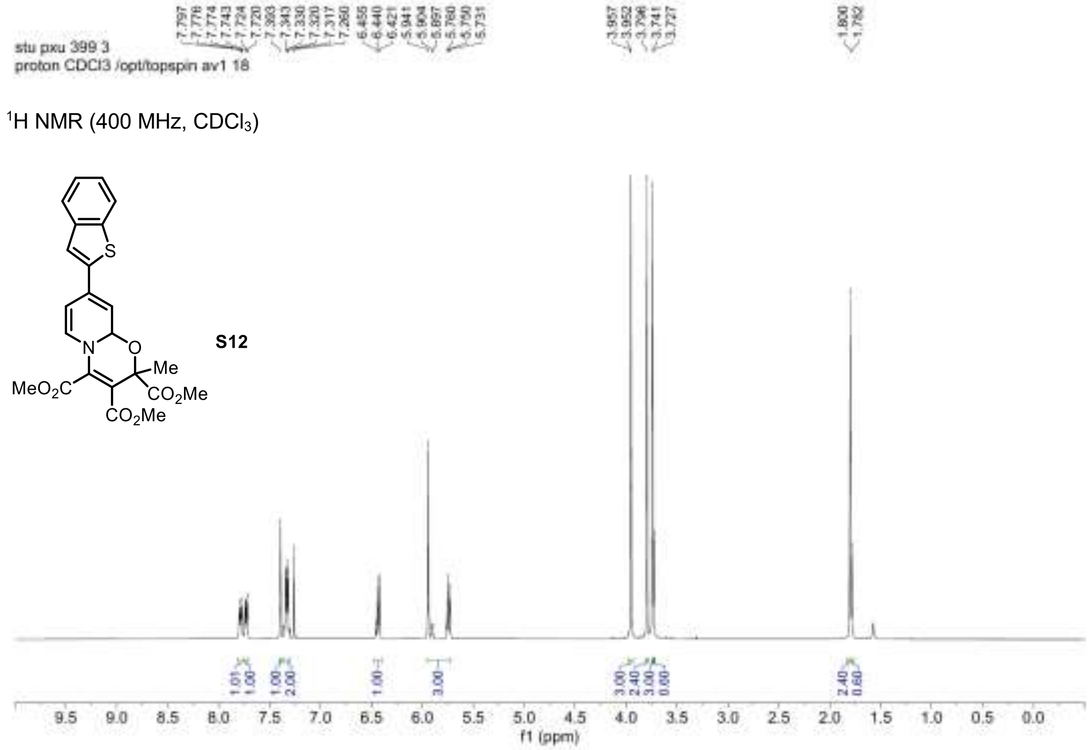

Supplementary Figure 17. ^{1}H NMR (400 MHz, $CDCl_3$) spectrum of compound **S12**

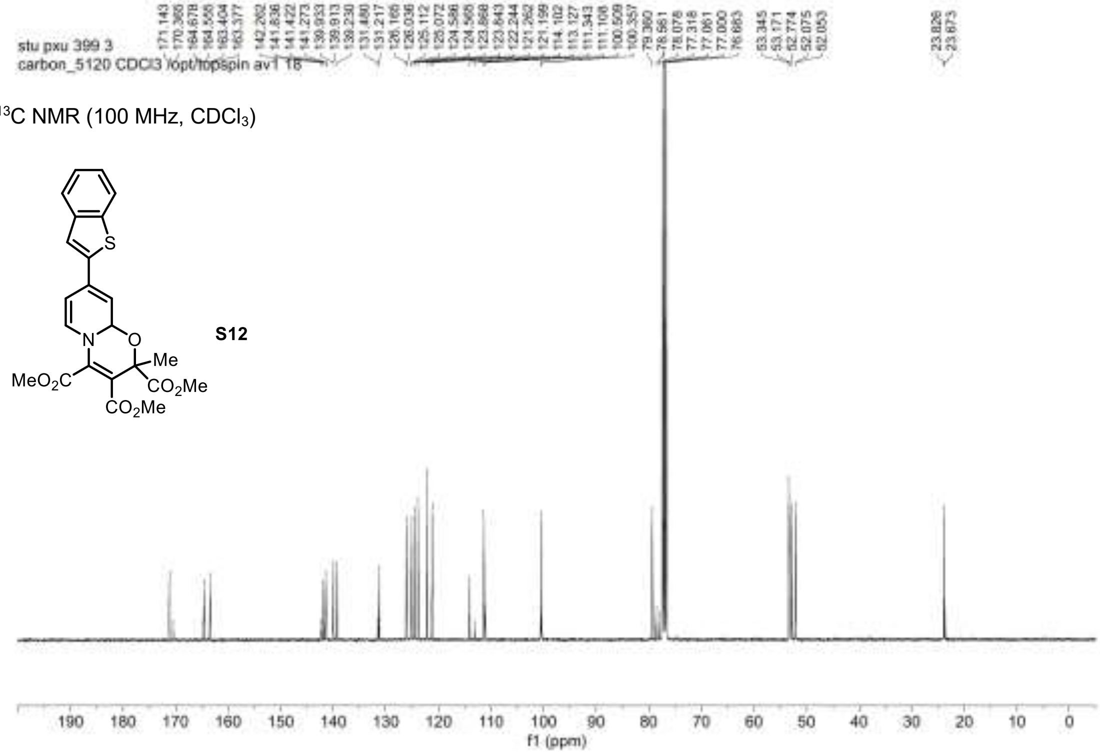

Supplementary Figure 18. ^{13}C NMR (100 MHz, $CDCl_3$) spectrum of compound **S12**

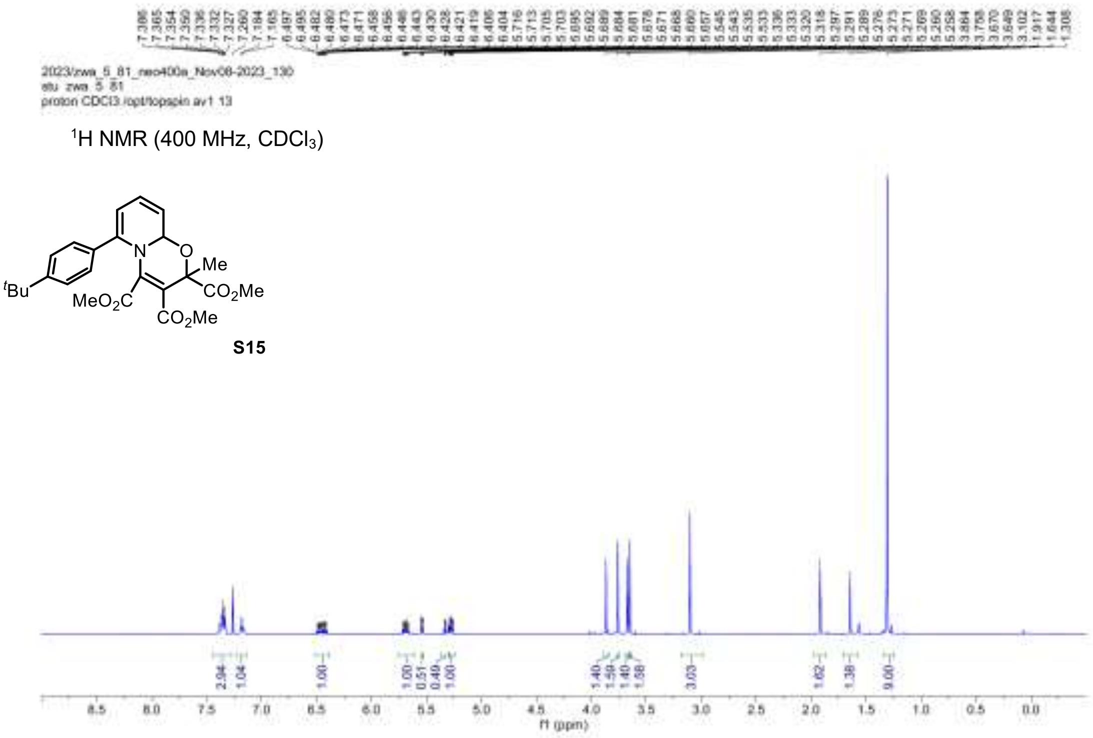

Supplementary Figure 19. ^{1}H NMR (400 MHz, $CDCl_3$) spectrum of compound **S15**

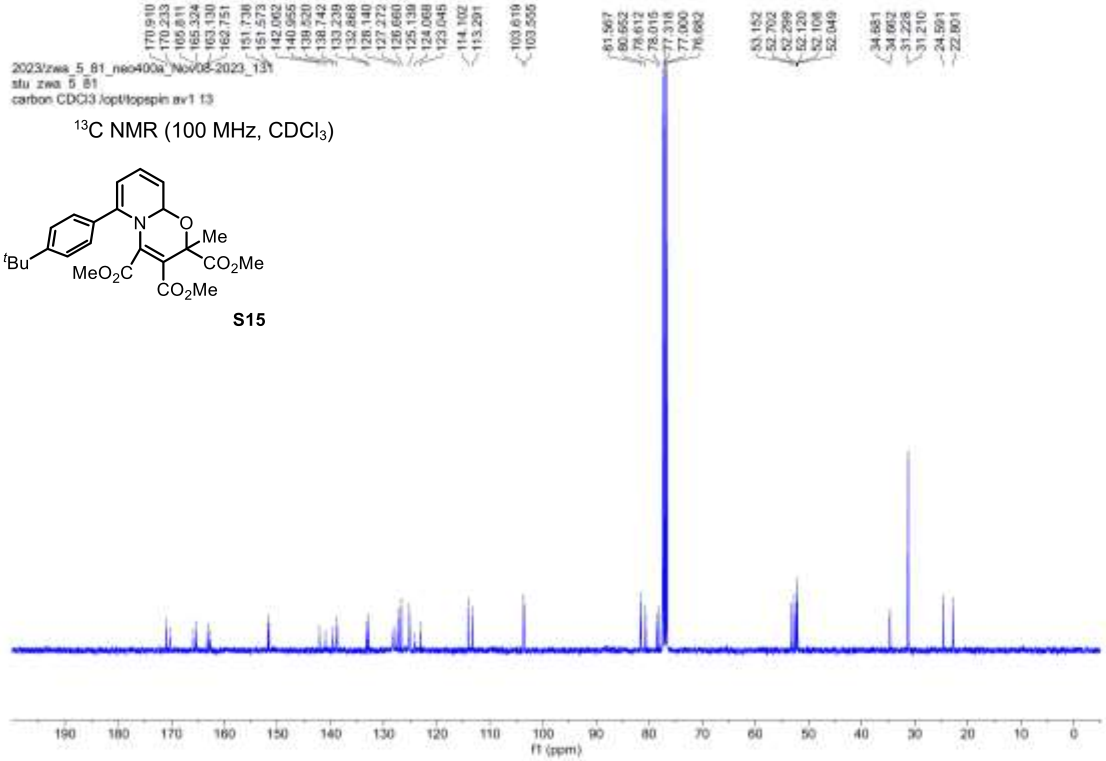

Supplementary Figure 20. ^{13}C NMR (100 MHz, $CDCl_3$) spectrum of compound **S15**

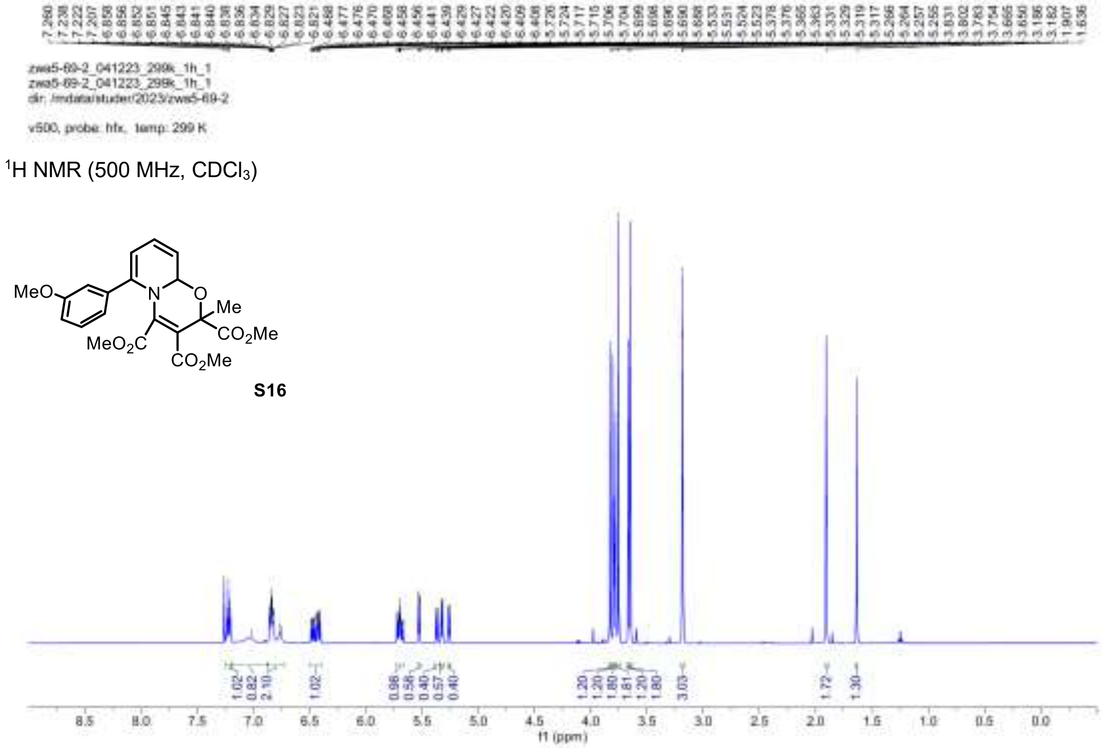

Supplementary Figure 21. ^{1}H NMR (500 MHz, $CDCl_3$) spectrum of compound **S16**

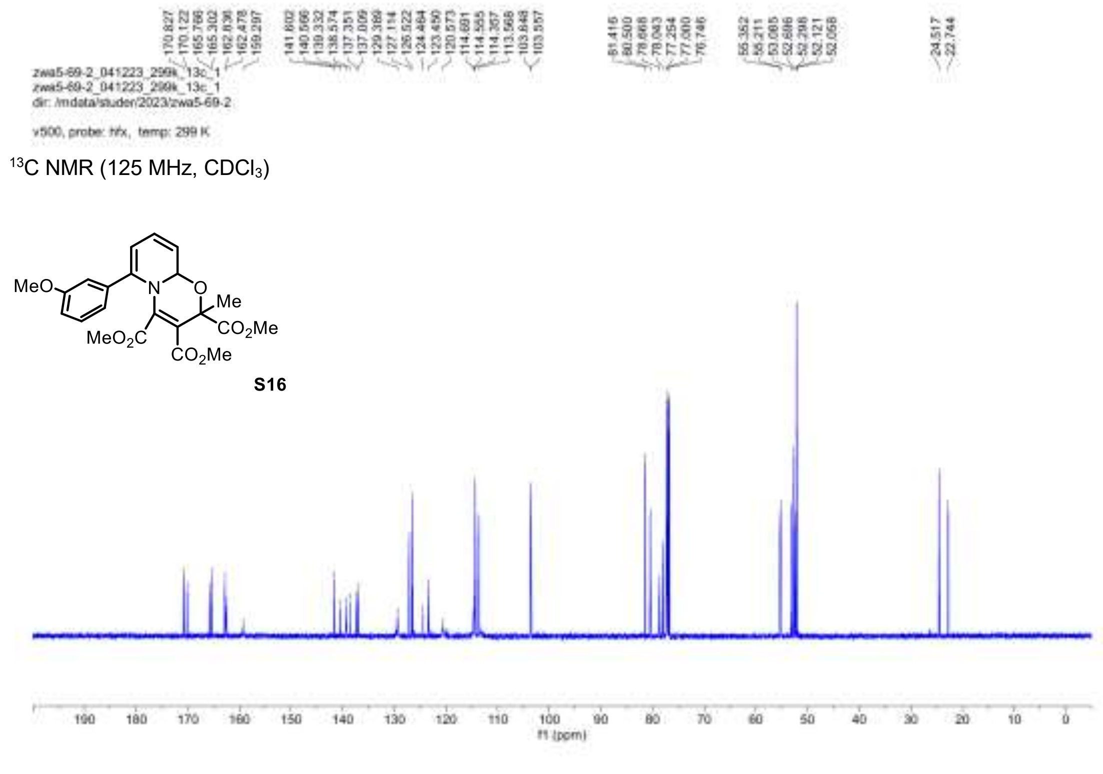

Supplementary Figure 22. ^{13}C NMR (125 MHz, $CDCl_3$) spectrum of compound **S16**

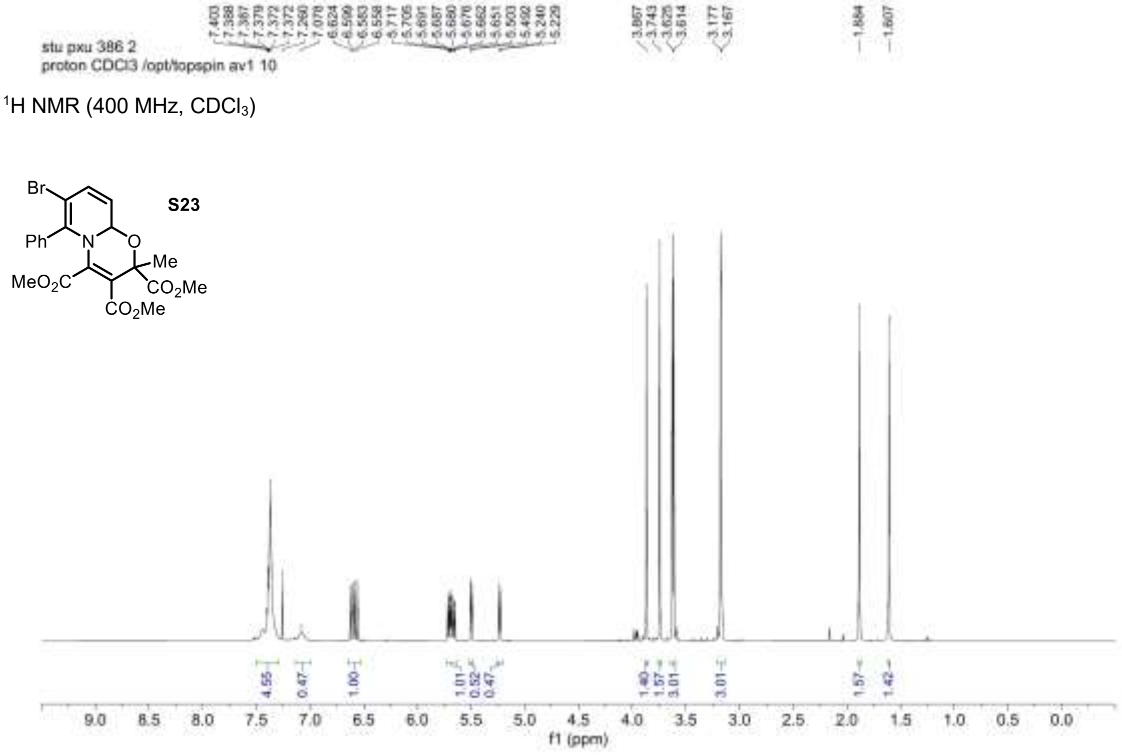

Supplementary Figure 23. ^{1}H NMR (400 MHz, $CDCl_3$) spectrum of compound **S23**

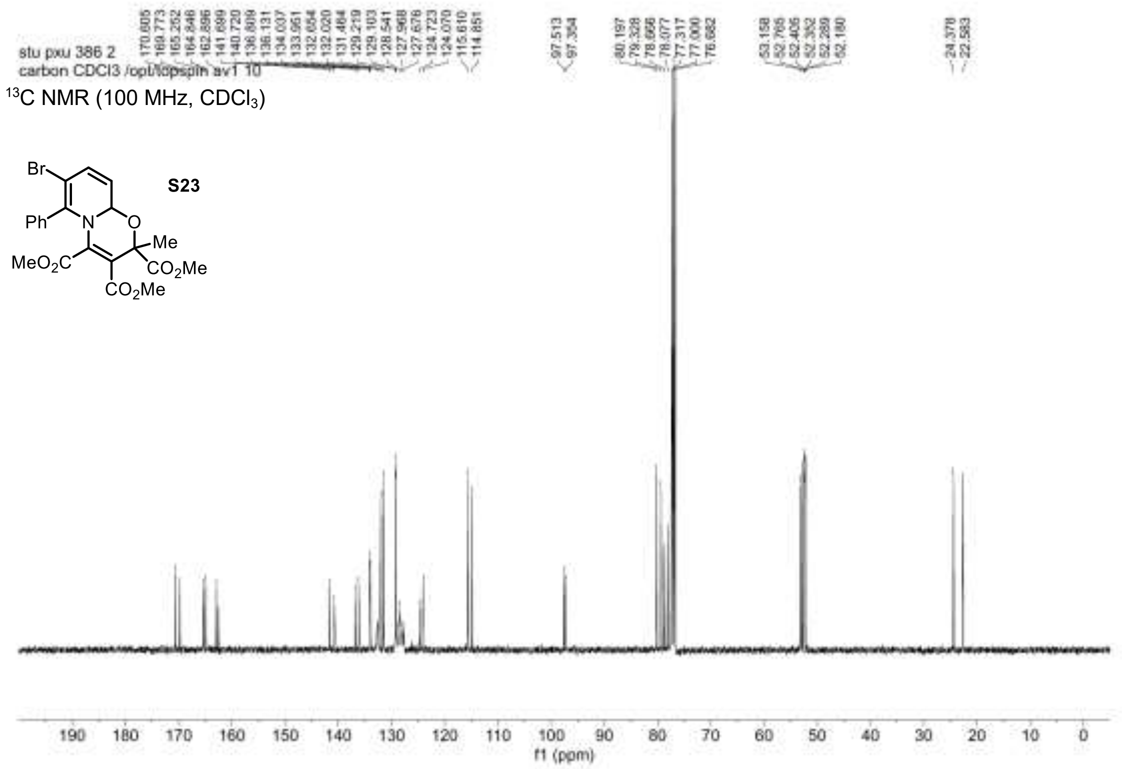

Supplementary Figure 24. ^{13}C NMR (100 MHz, $CDCl_3$) spectrum of compound **S23**

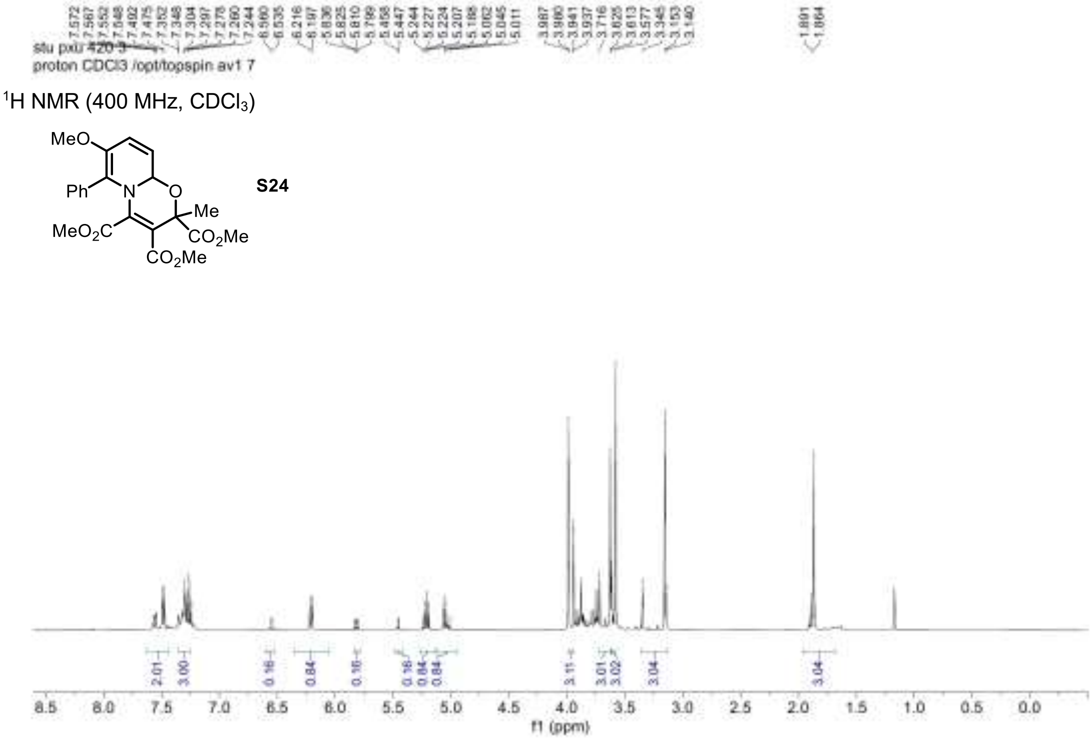

Supplementary Figure 25. ^{1}H NMR (400 MHz, $CDCl_3$) spectrum of compound **S24**

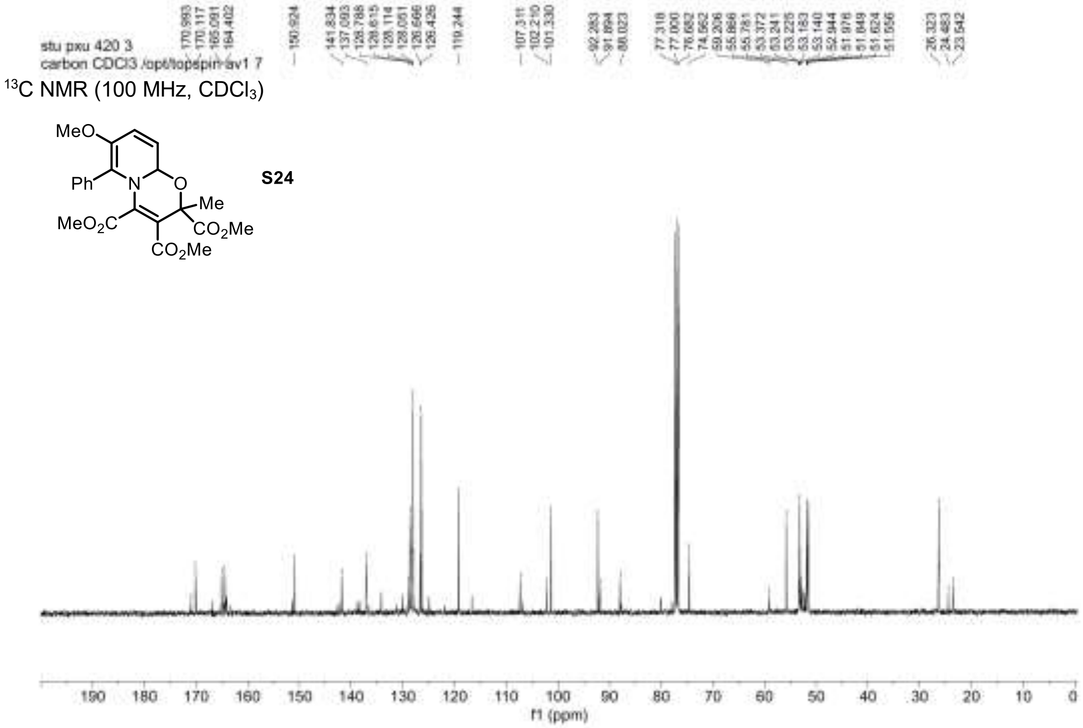

Supplementary Figure 26. ^{13}C NMR (100 MHz, $CDCl_3$) spectrum of compound **S24**

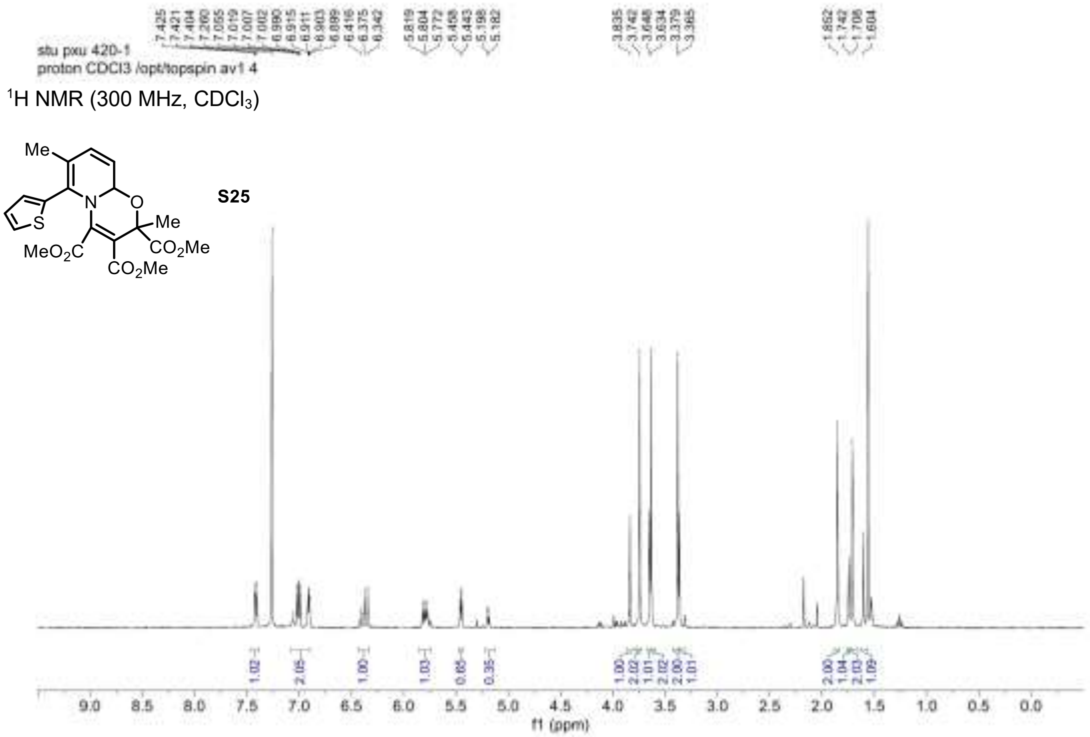

Supplementary Figure 27. ^{1}H NMR (300 MHz, $CDCl_3$) spectrum of compound **S25**

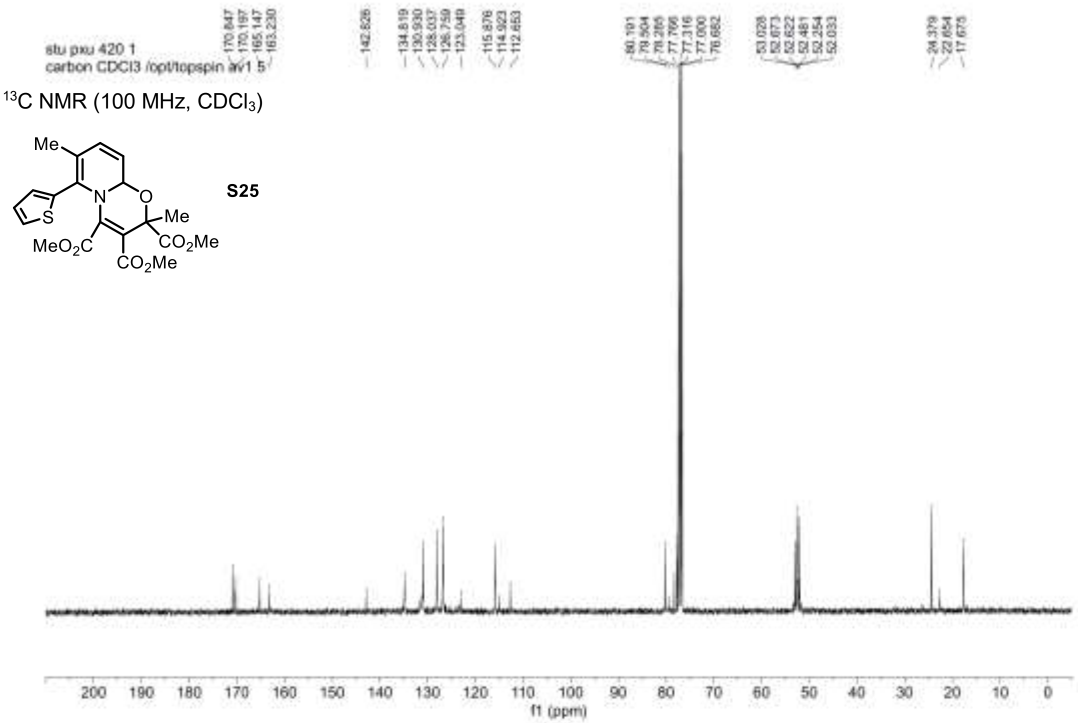

Supplementary Figure 28. ^{13}C NMR (100 MHz, $CDCl_3$) spectrum of compound **S25**

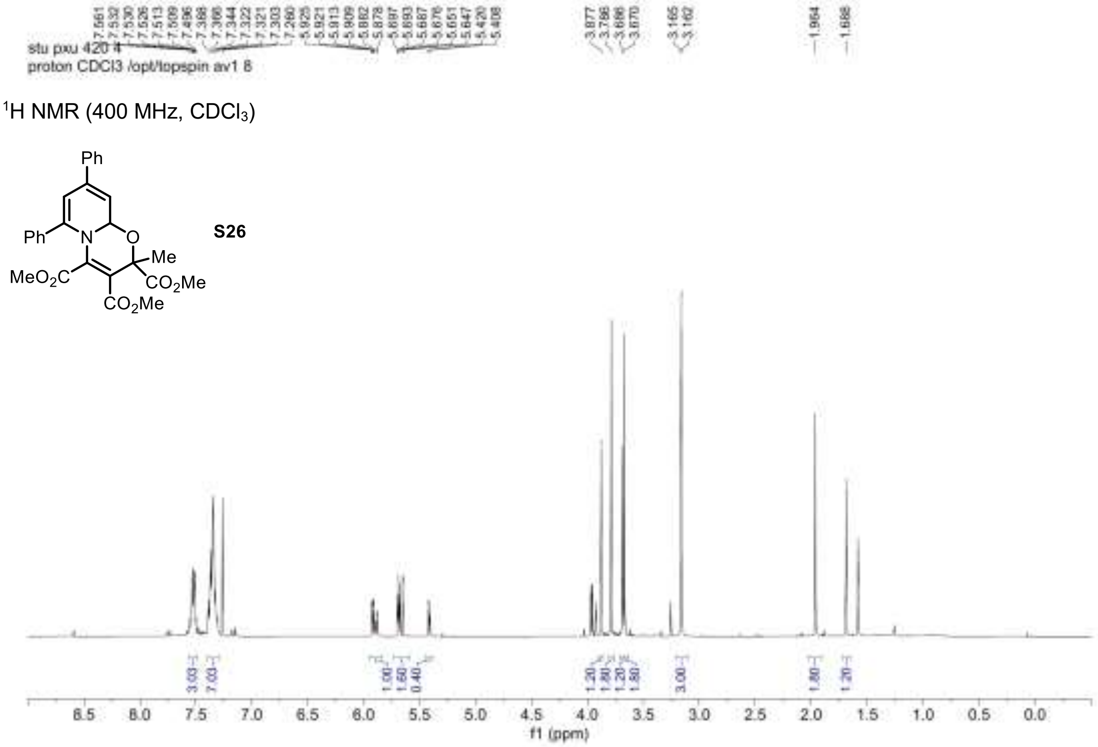

Supplementary Figure 29. ^{1}H NMR (400 MHz, $CDCl_3$) spectrum of compound **S26**

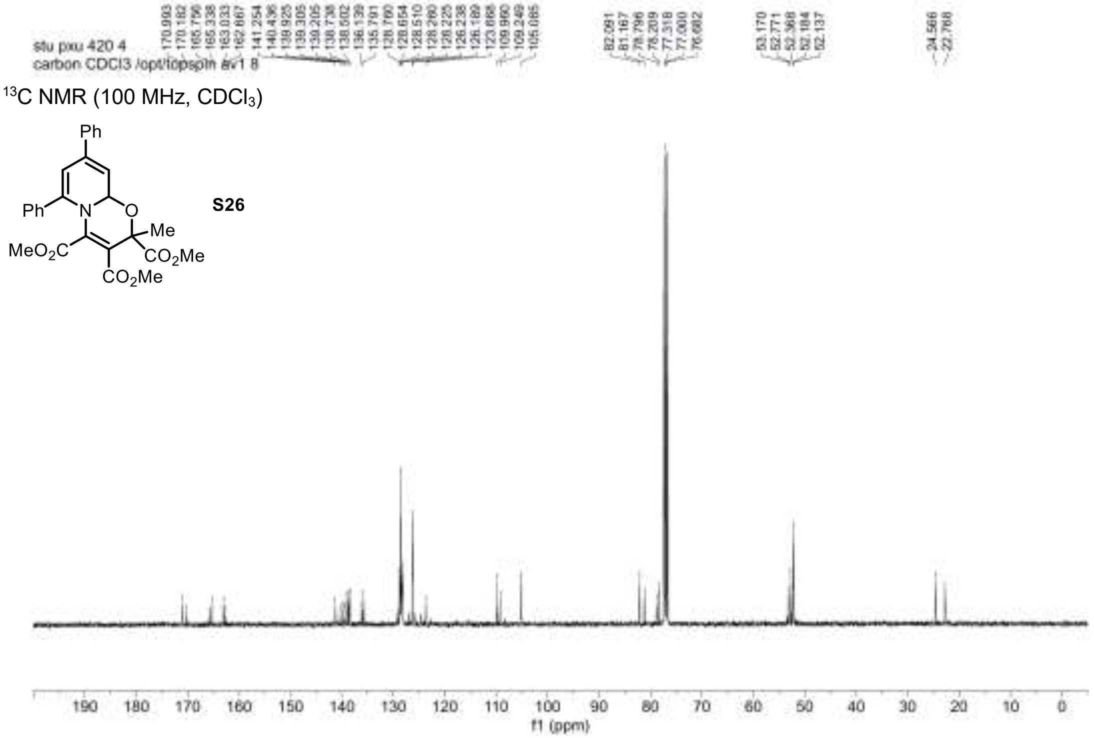

Supplementary Figure 30. ^{13}C NMR (100 MHz, $CDCl_3$) spectrum of compound **S26**

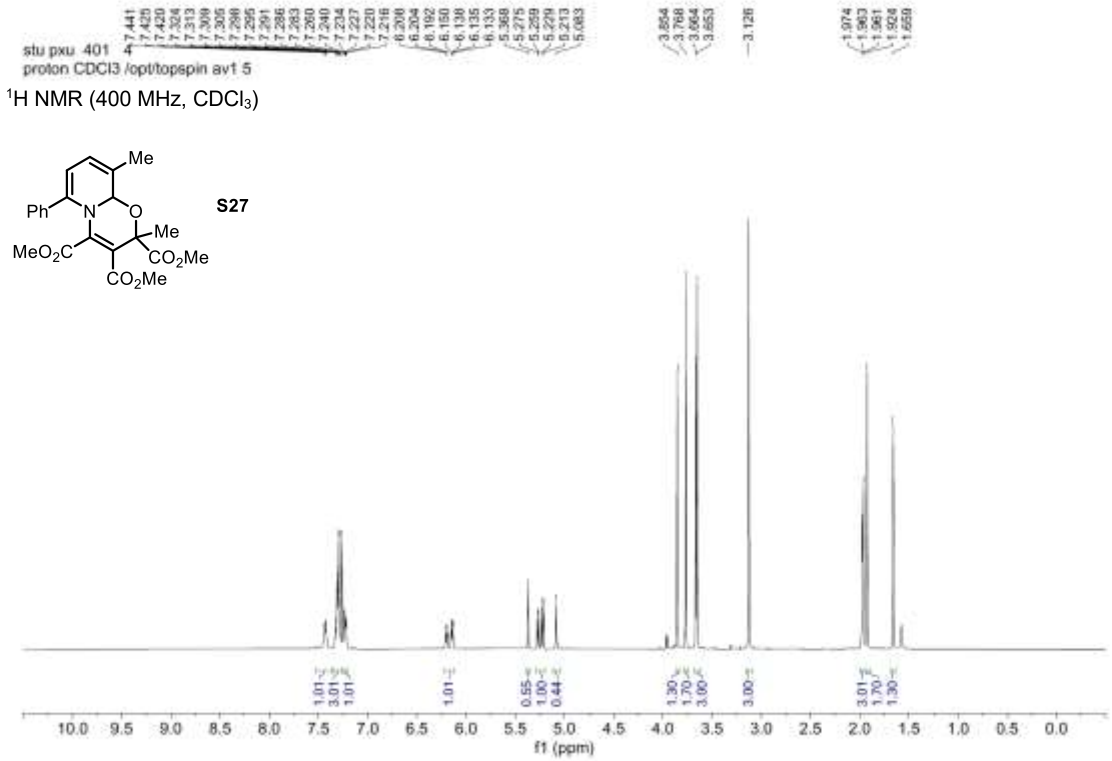

Supplementary Figure 31. ^{1}H NMR (400 MHz, $CDCl_3$) spectrum of compound **S27**

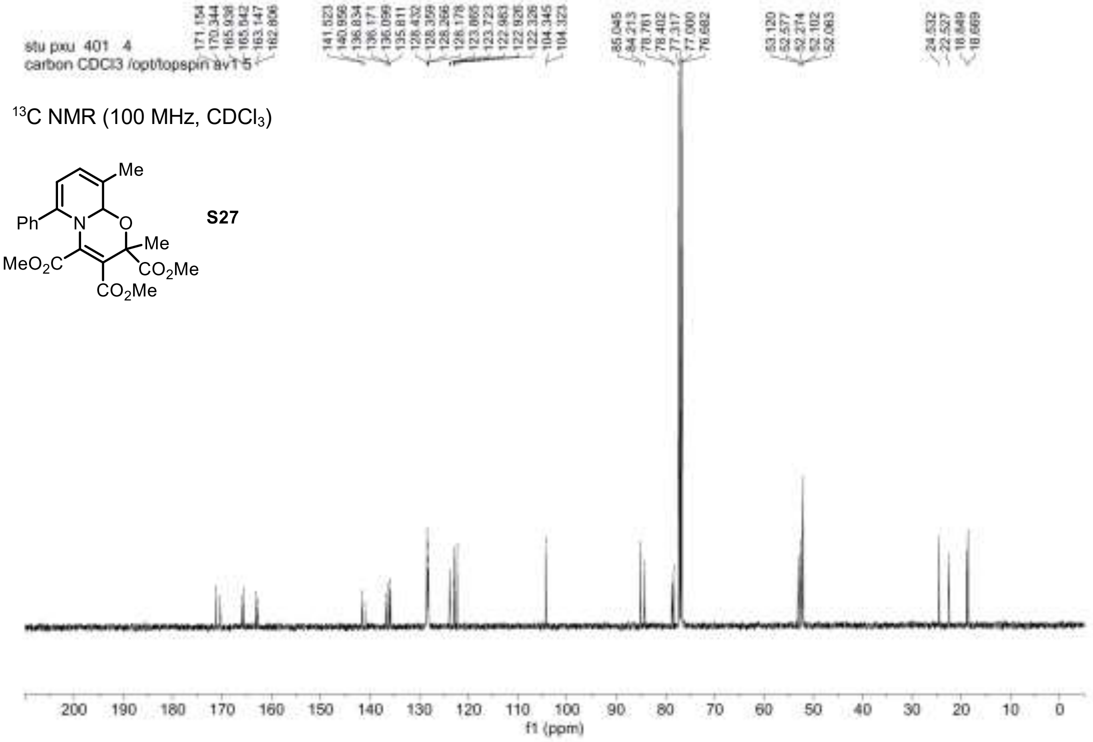

Supplementary Figure 32. ^{13}C NMR (100 MHz, $CDCl_3$) spectrum of compound **S27**

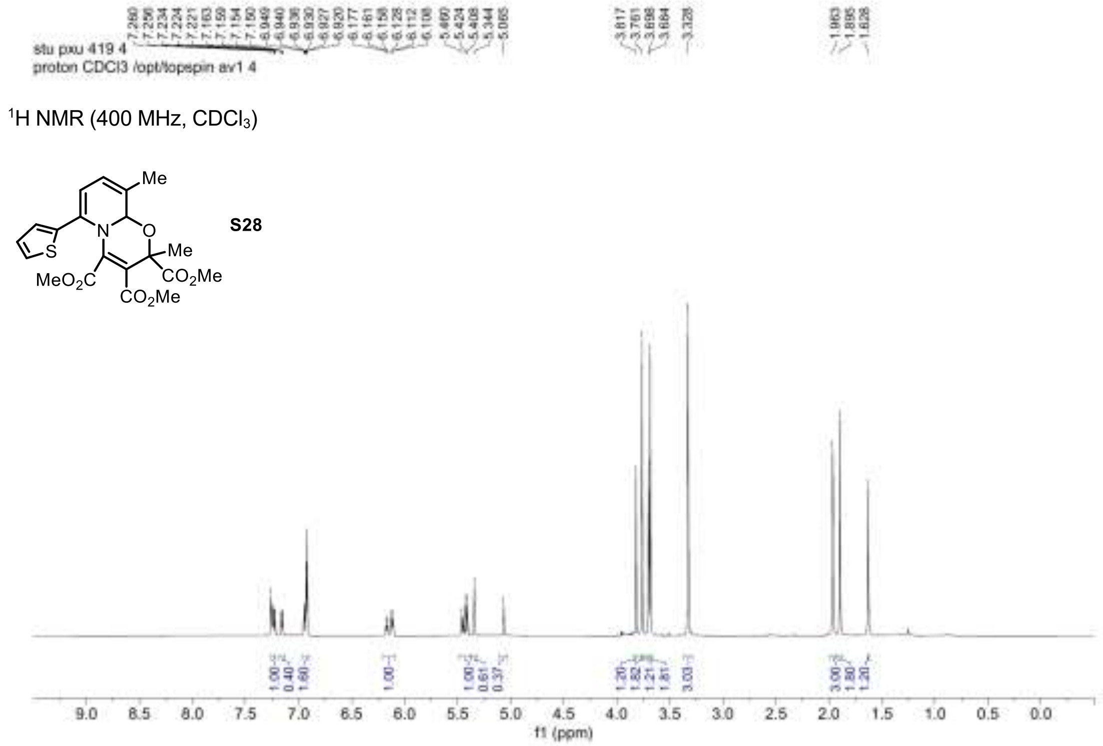

Supplementary Figure 33. ^{1}H NMR (400 MHz, $CDCl_3$) spectrum of compound **S28**

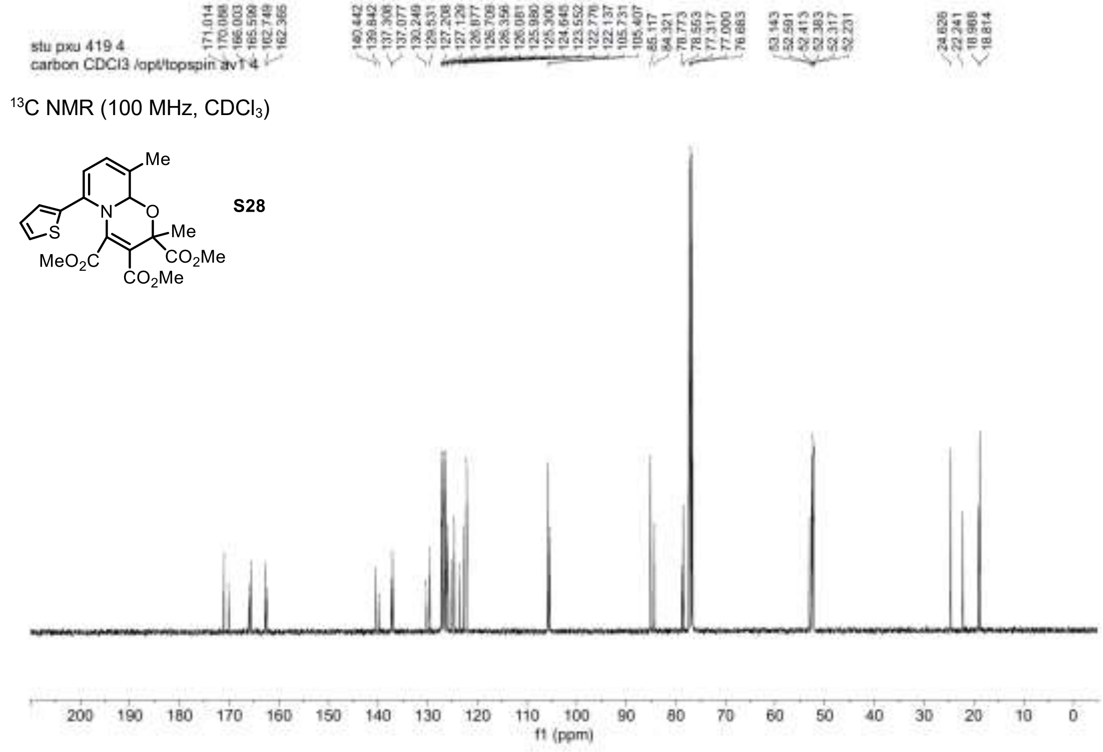

Supplementary Figure 34. ^{13}C NMR (100 MHz, $CDCl_3$) spectrum of compound **S28**

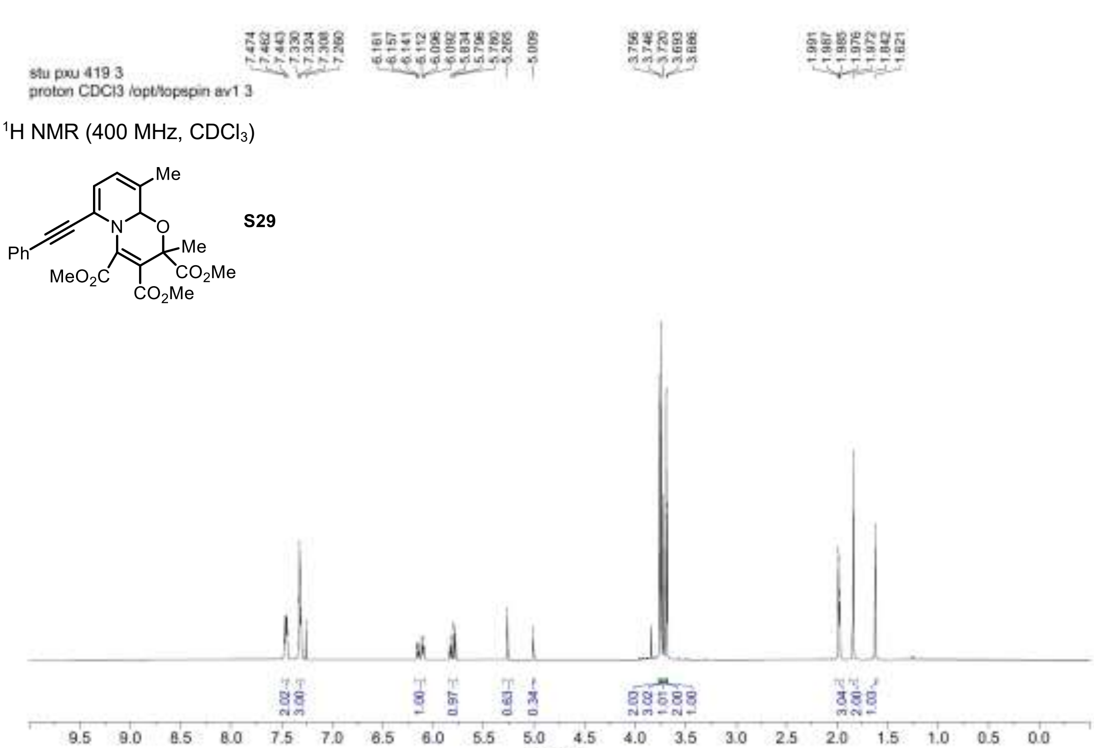

Supplementary Figure 35. ^{1}H NMR (400 MHz, $CDCl_3$) spectrum of compound **S29**

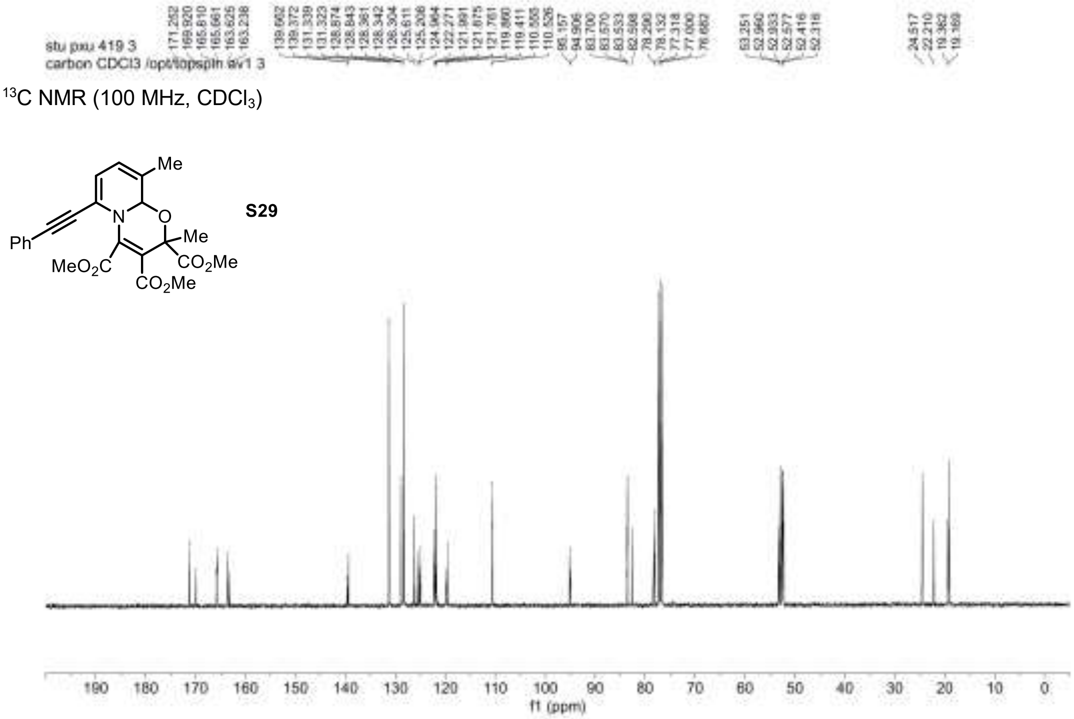

Supplementary Figure 36. ^{13}C NMR (100 MHz, $CDCl_3$) spectrum of compound **S29**

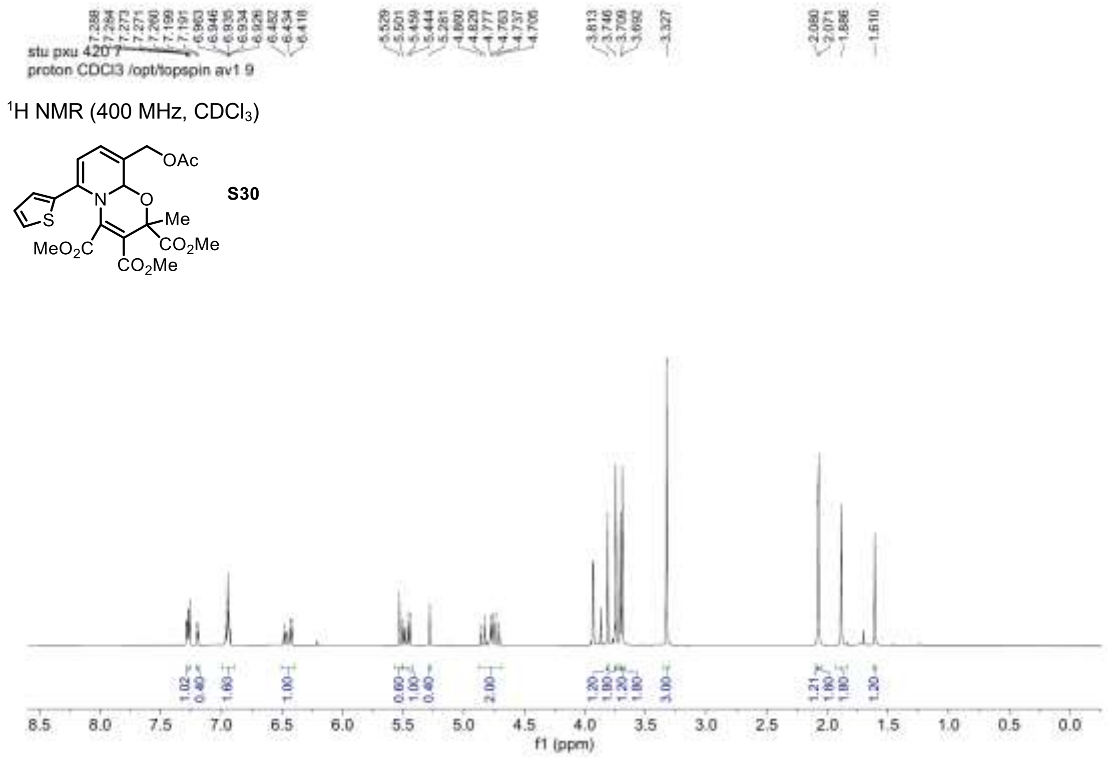

Supplementary Figure 37. ^{1}H NMR (400 MHz, $CDCl_3$) spectrum of compound **S30**

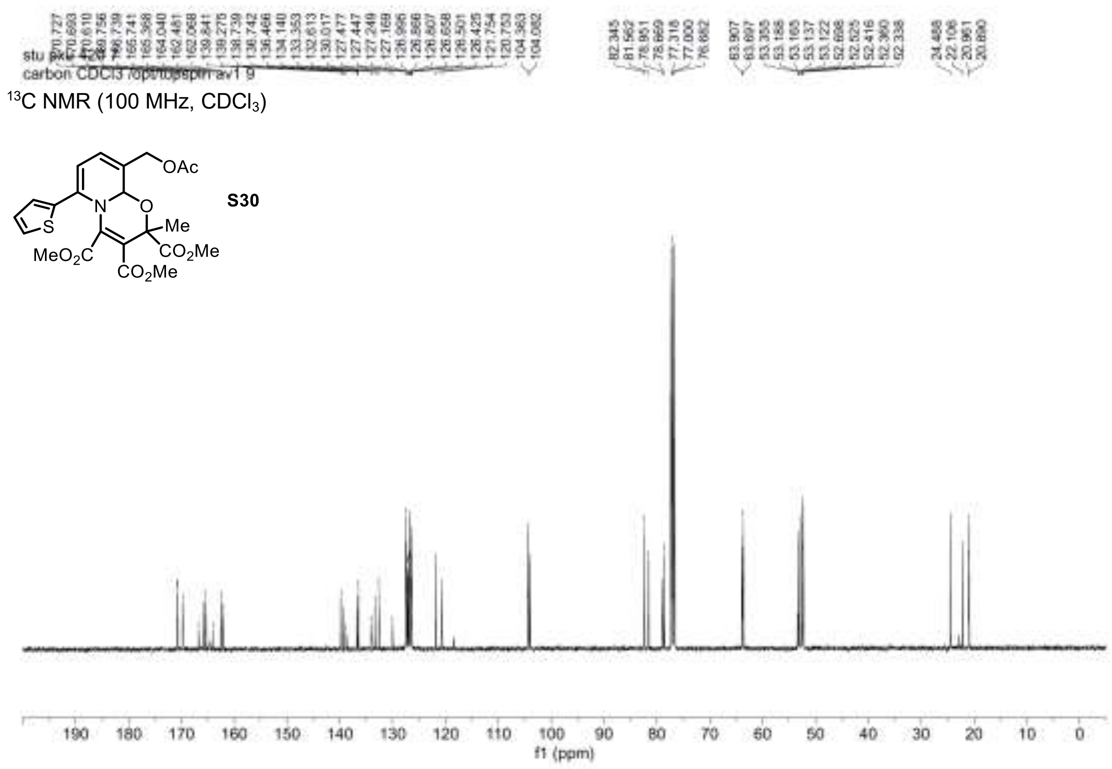

Supplementary Figure 38. ^{13}C NMR (100 MHz, $CDCl_3$) spectrum of compound **S30**

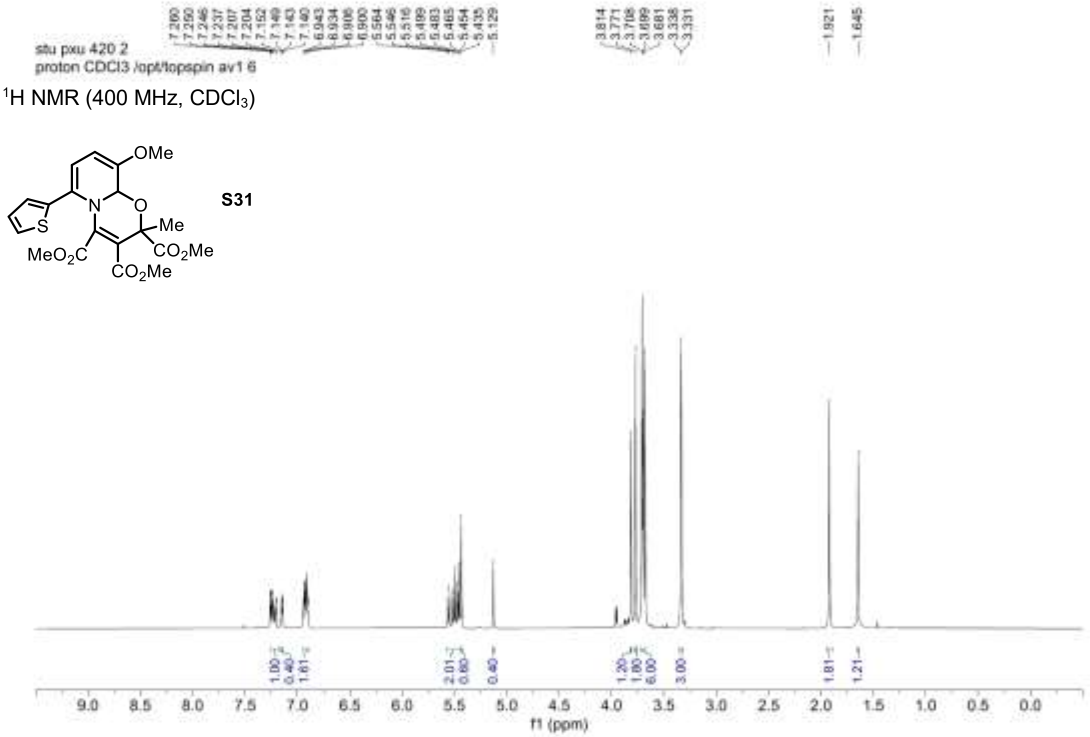

Supplementary Figure 39. ^{1}H NMR (400 MHz, $CDCl_3$) spectrum of compound **S31**

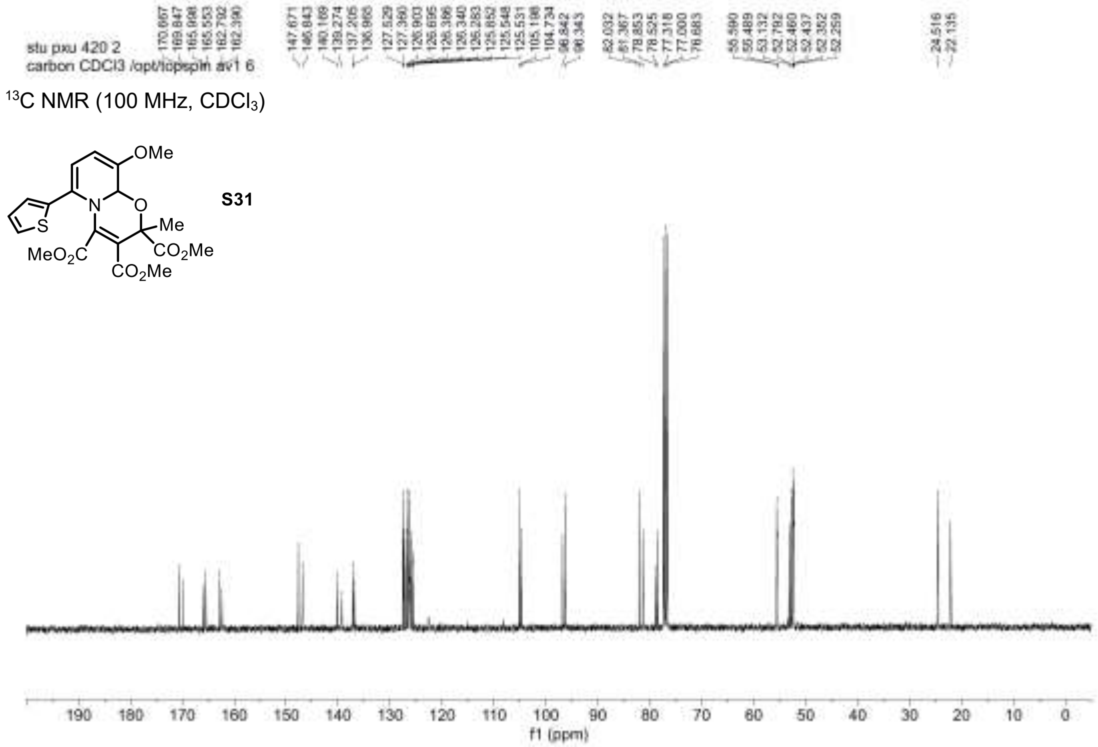

Supplementary Figure 40. ^{13}C NMR (100 MHz, $CDCl_3$) spectrum of compound **S31**

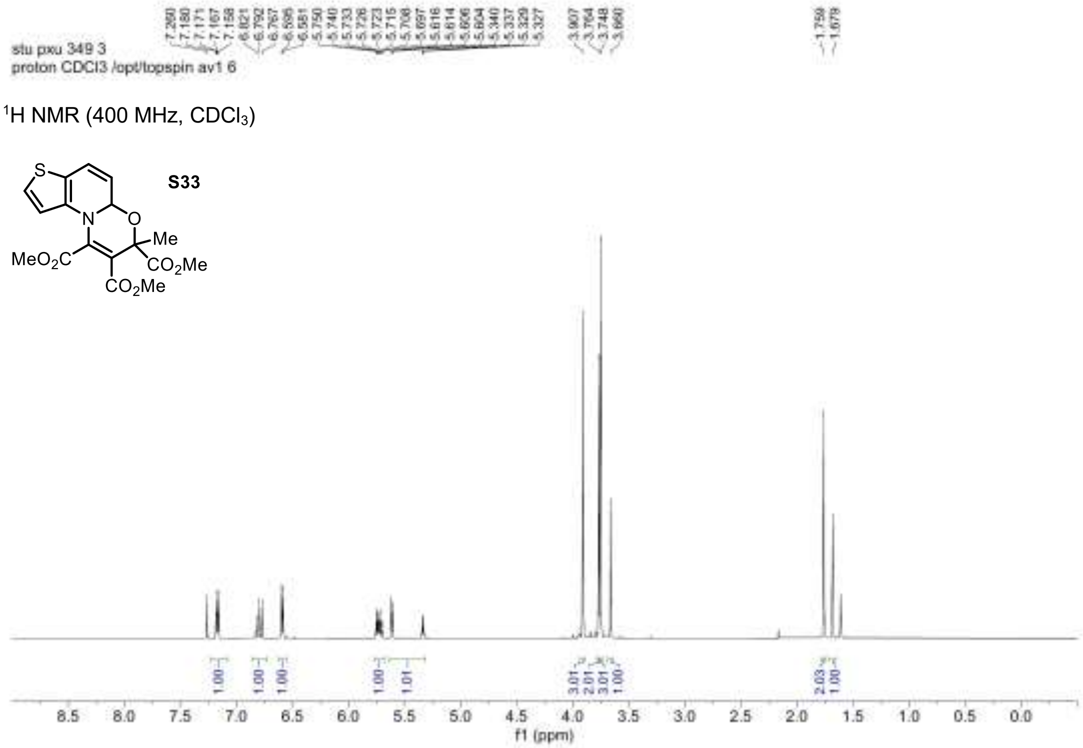

Supplementary Figure 41. ^{1}H NMR (400 MHz, $CDCl_3$) spectrum of compound **S33**

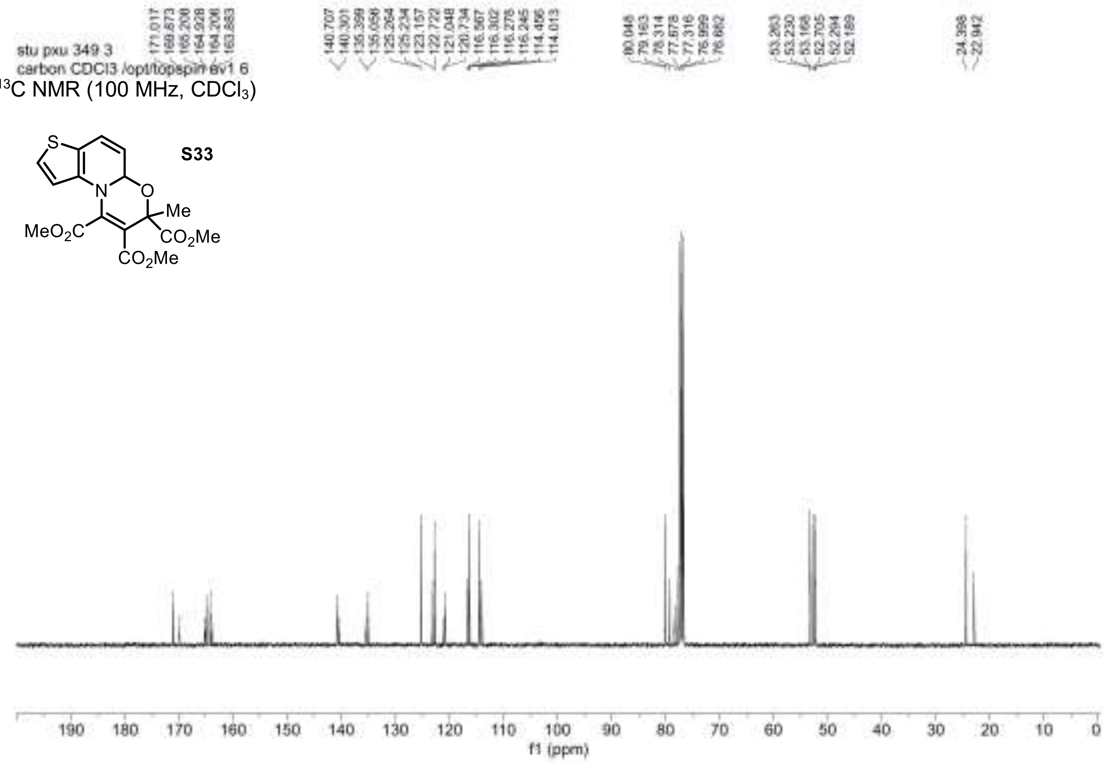

Supplementary Figure 42. ^{13}C NMR (100 MHz, $CDCl_3$) spectrum of compound **S33**

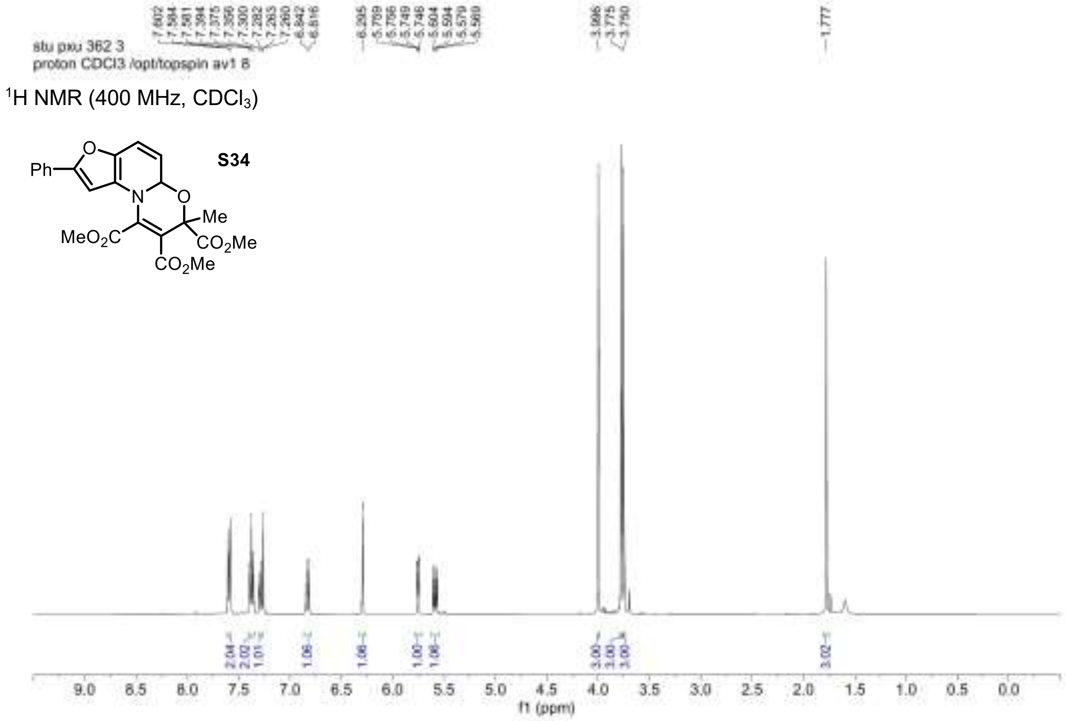

Supplementary Figure 43. ^{1}H NMR (400 MHz, $CDCl_3$) spectrum of compound **S34**

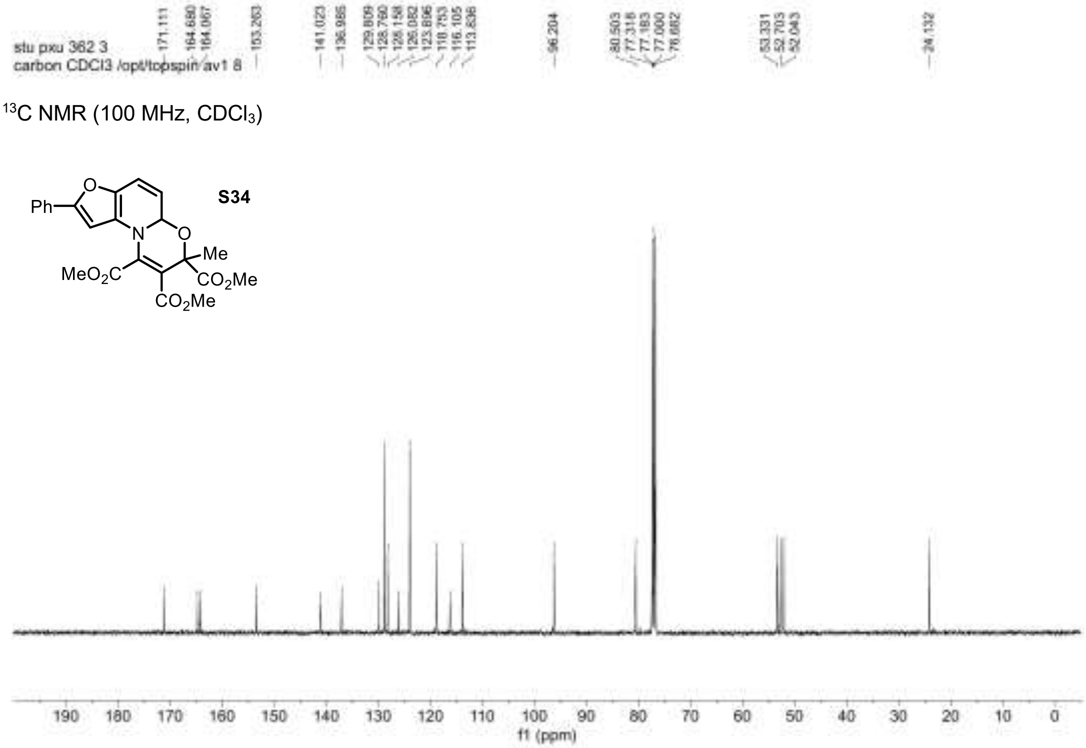

Supplementary Figure 44. ^{13}C NMR (100 MHz, $CDCl_3$) spectrum of compound **S34**

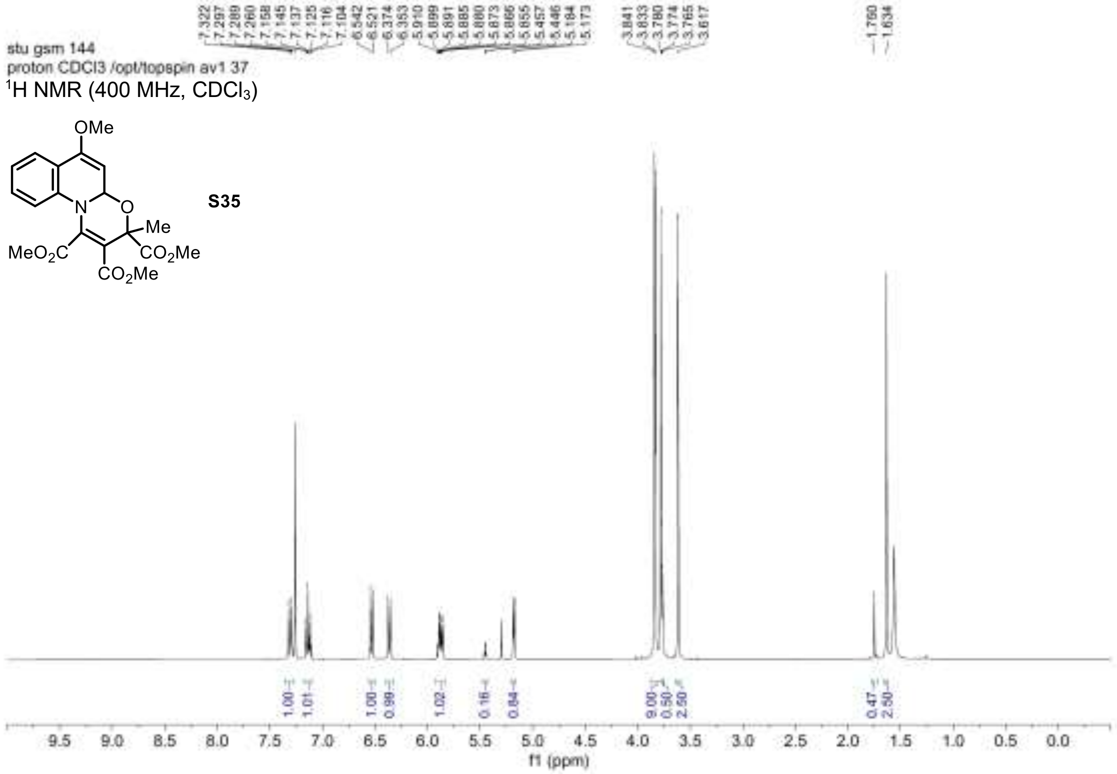

Supplementary Figure 45. ^{1}H NMR (400 MHz, $CDCl_3$) spectrum of compound **S35**

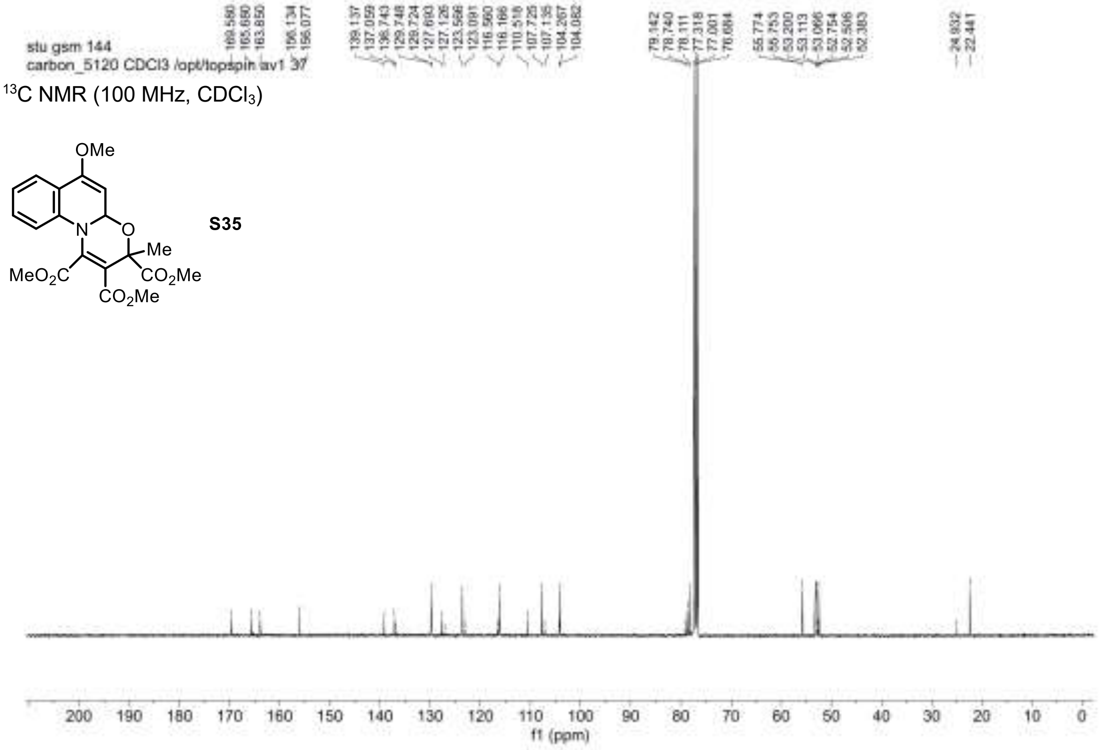

Supplementary Figure 46. ^{13}C NMR (100 MHz, $CDCl_3$) spectrum of compound **S35**

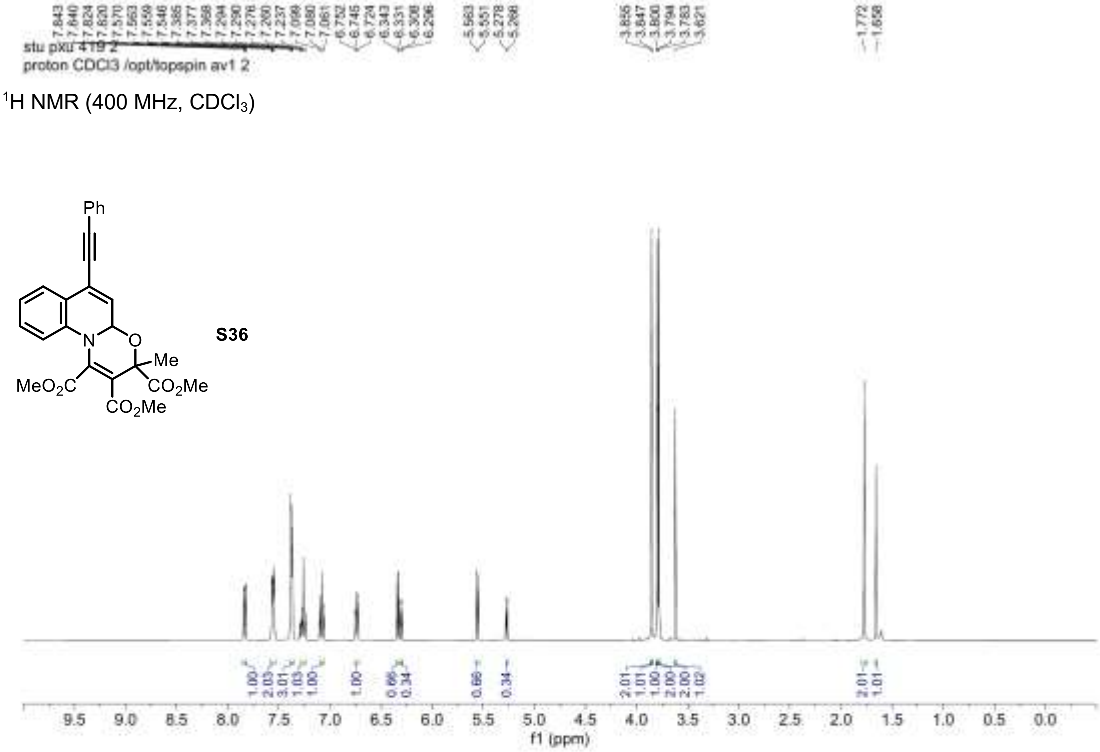

Supplementary Figure 47. ^{1}H NMR (400 MHz, $CDCl_3$) spectrum of compound **S36**

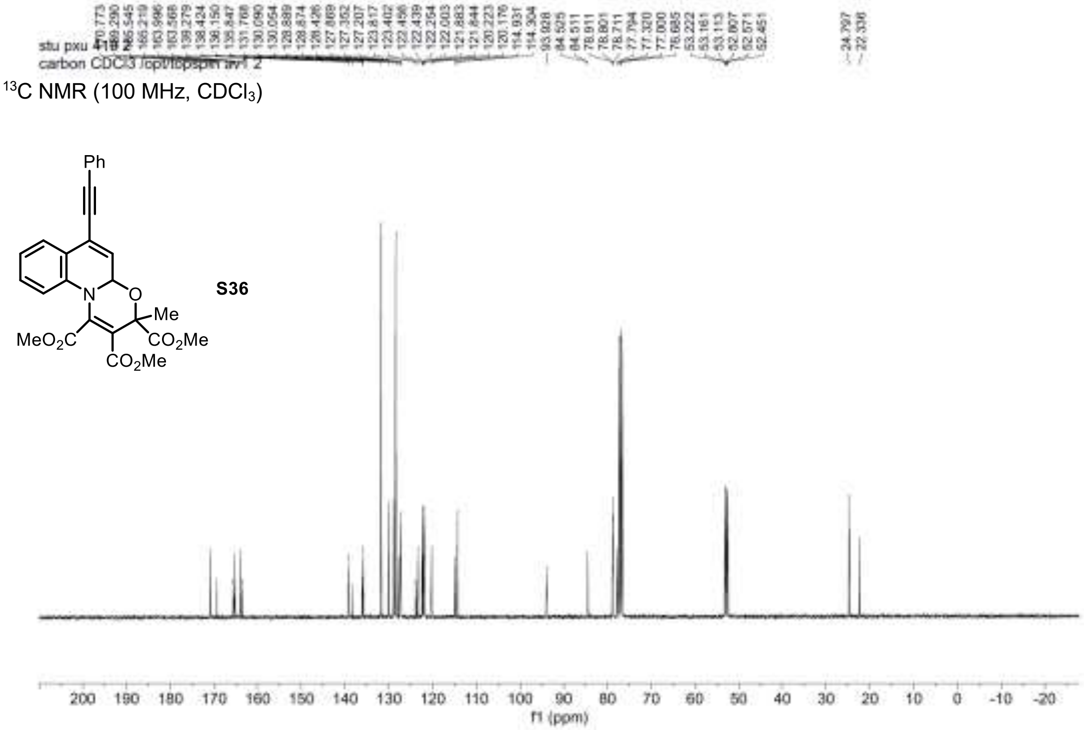

Supplementary Figure 48. ^{13}C NMR (100 MHz, $CDCl_3$) spectrum of compound **S36**

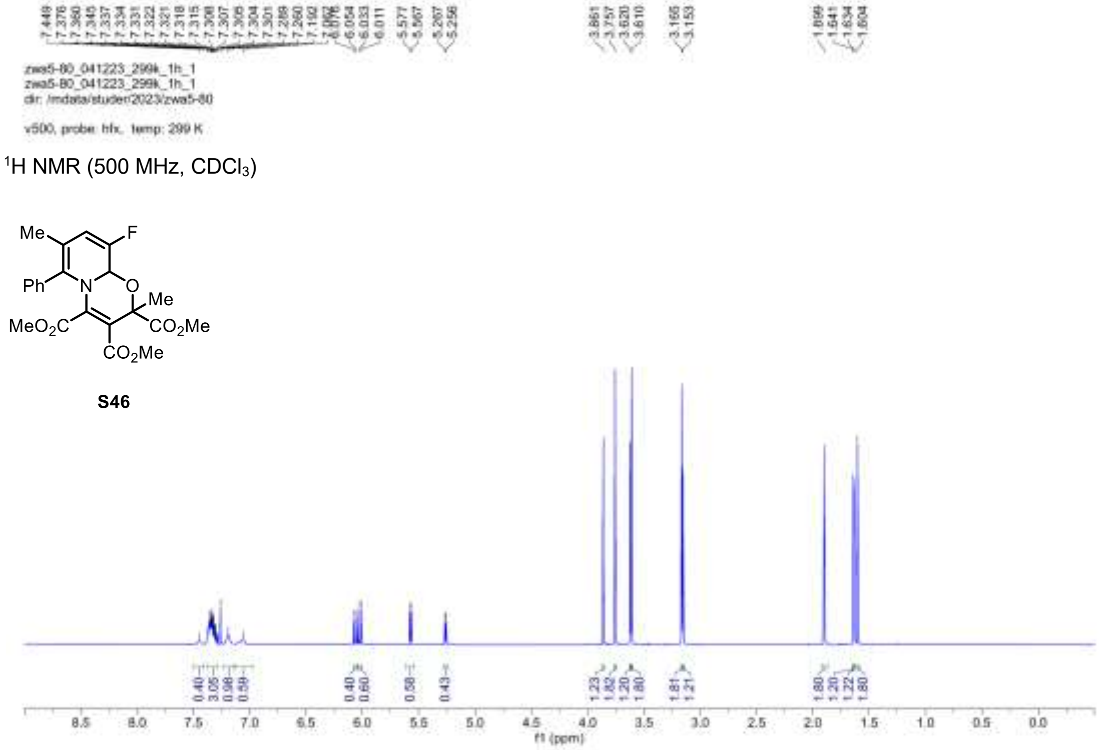

Supplementary Figure 49. ^{1}H NMR (500 MHz, $CDCl_3$) spectrum of compound **S46**

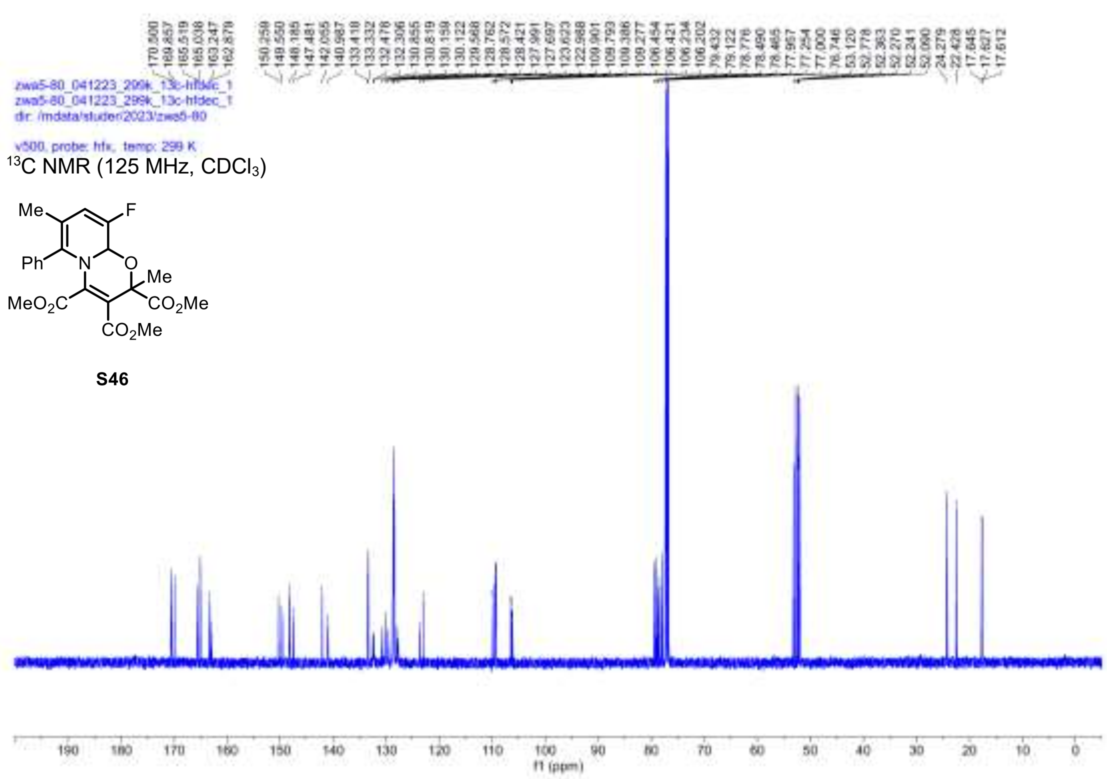

Supplementary Figure 50. ^{13}C NMR (125 MHz, $CDCl_3$) spectrum of compound **S46**

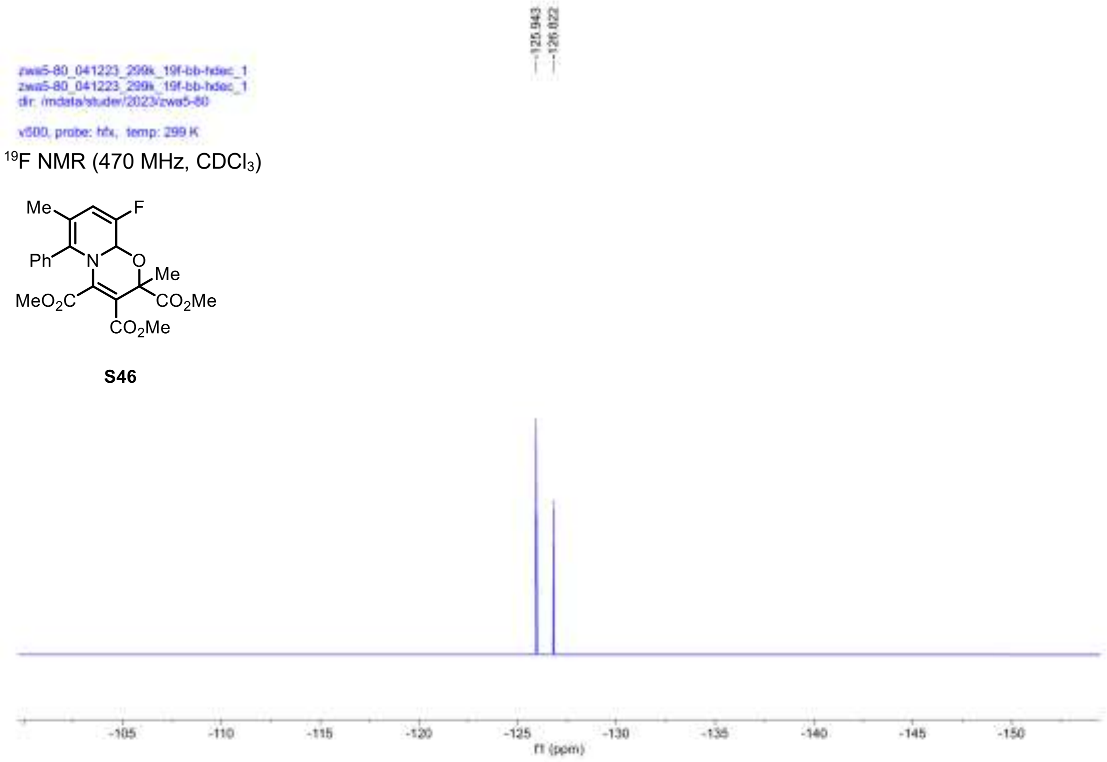

Supplementary Figure 51. ^{19}F NMR (470 MHz, $CDCl_3$) spectrum of compound **S46**

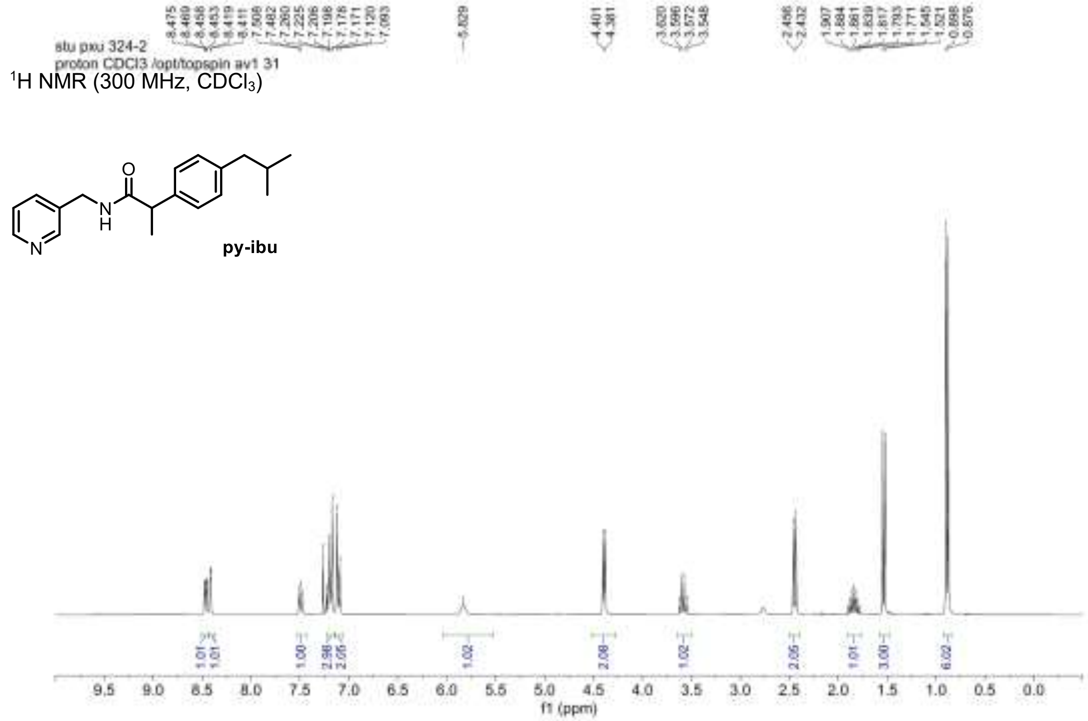

Supplementary Figure 52. ^{1}H NMR (300 MHz, $CDCl_3$) spectrum of compound **py-ibu**

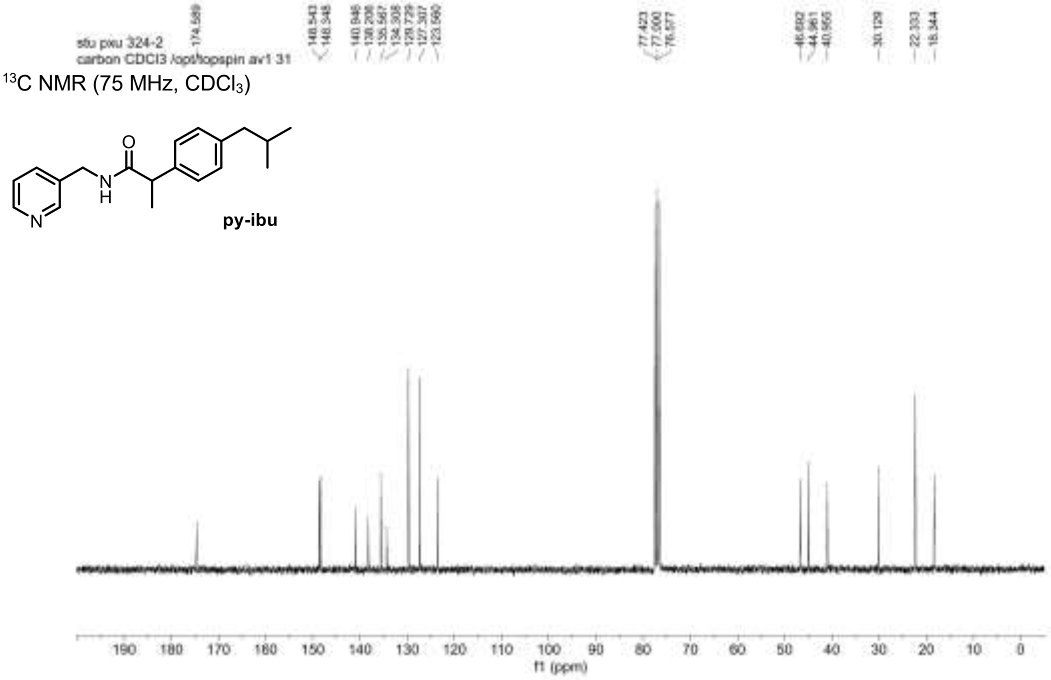

Supplementary Figure 53. ^{13}C NMR (75 MHz, $CDCl_3$) spectrum of compound **py-ibu**

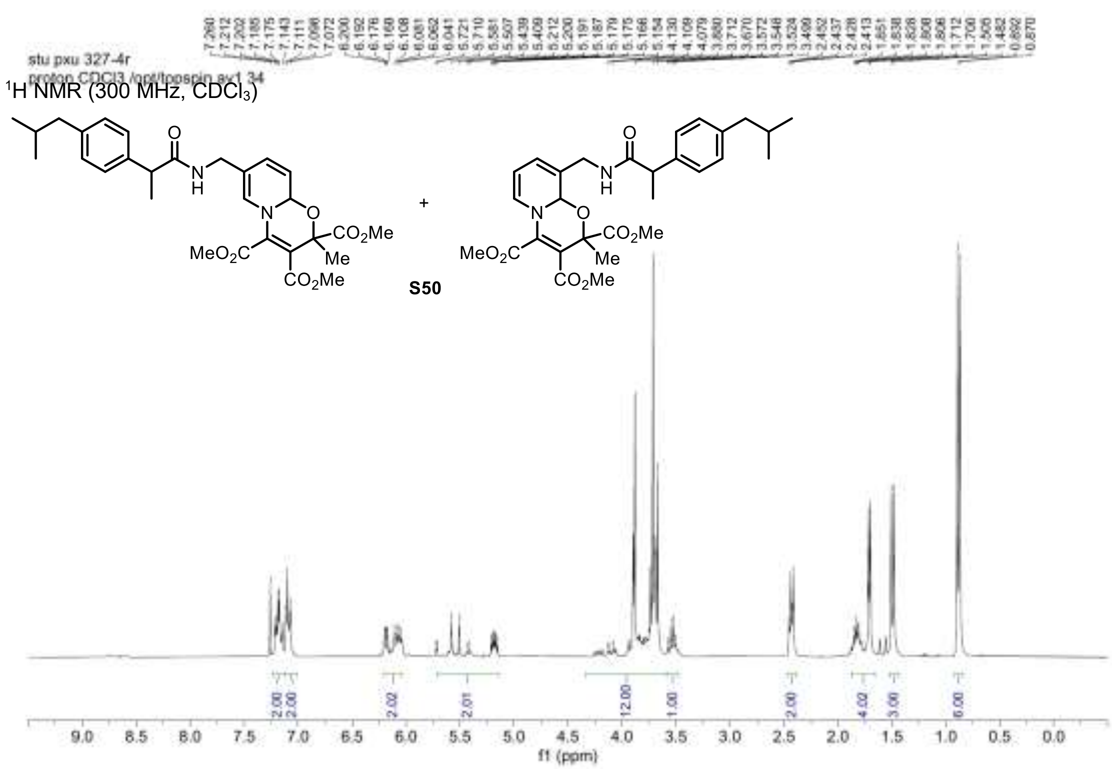

Supplementary Figure 54. ^{1}H NMR (300 MHz, $CDCl_3$) spectrum of compound **S50**

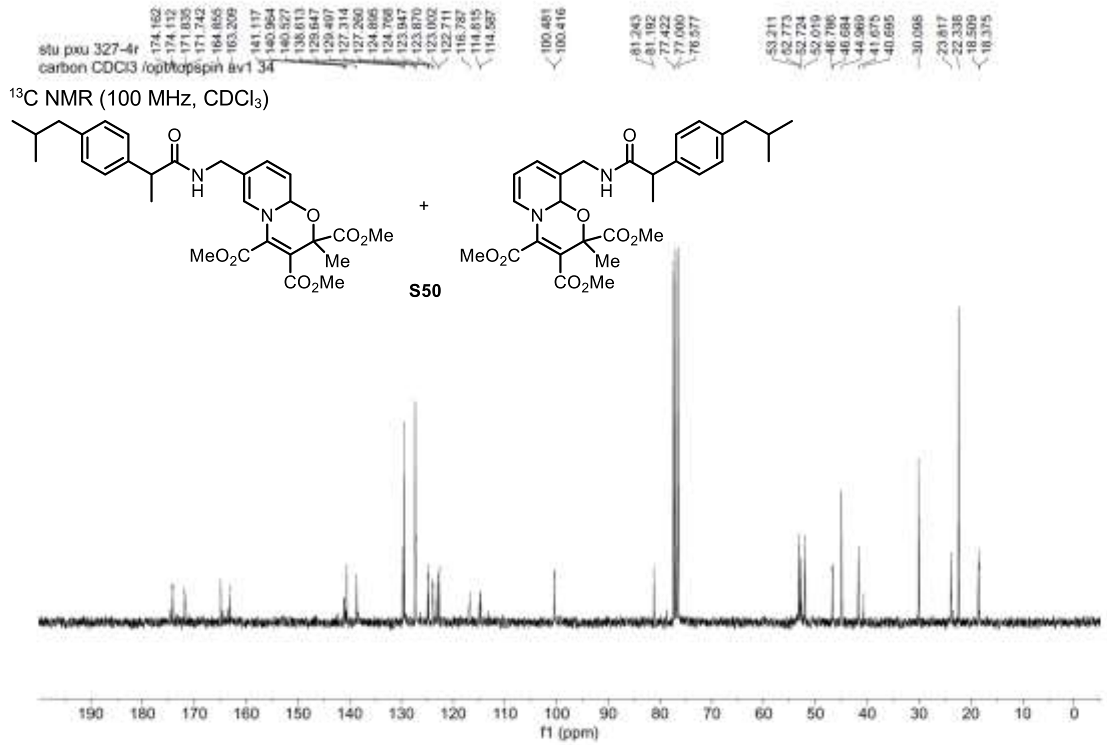

Supplementary Figure 55. ^{13}C NMR (100 MHz, $CDCl_3$) spectrum of compound **S50**

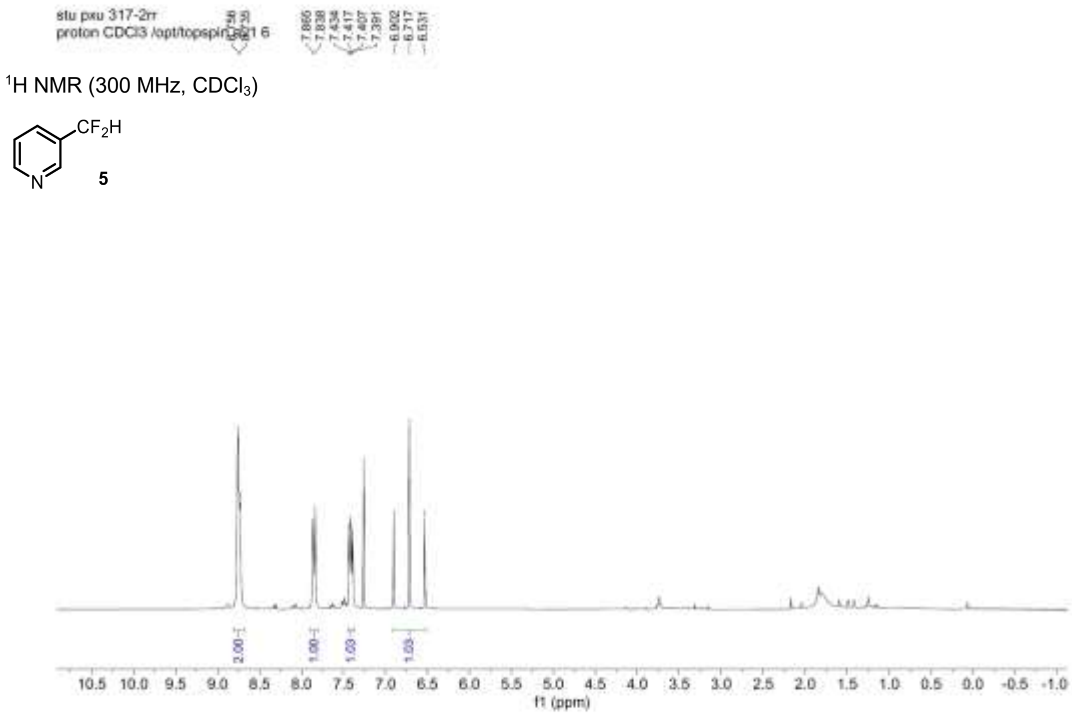

Supplementary Figure 56. ^{1}H NMR (300 MHz, $CDCl_3$) spectrum of compound **5**

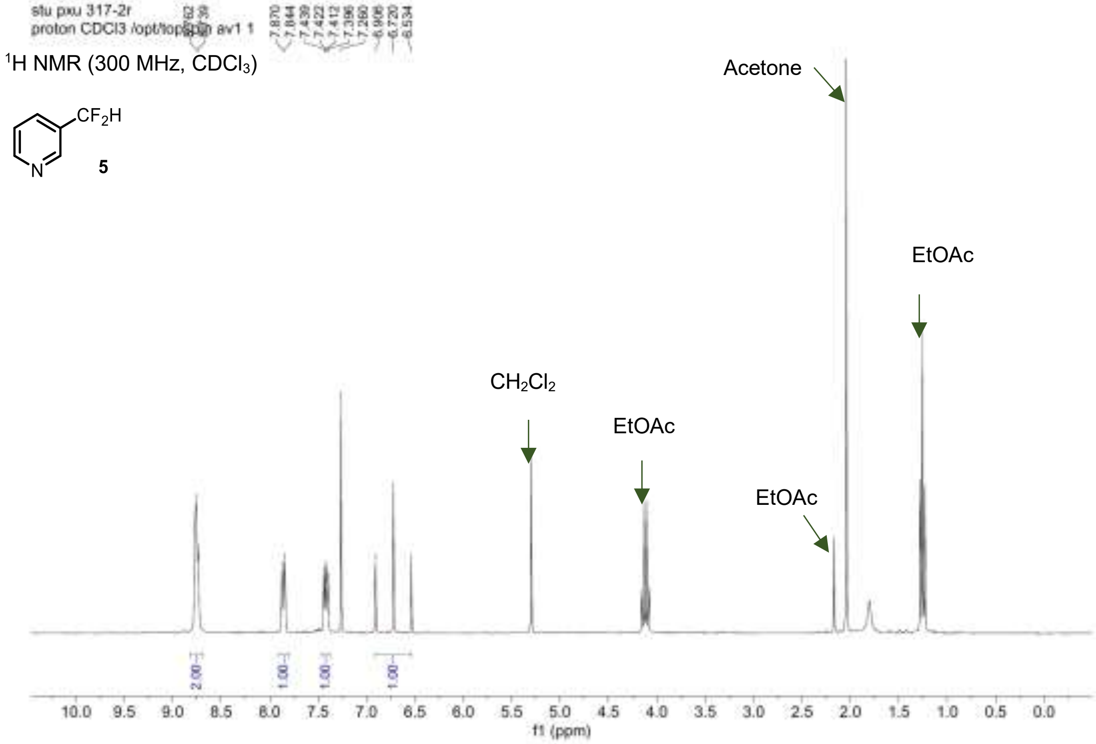

Supplementary Figure 57. ^{1}H NMR (300 MHz, $CDCl_3$) spectrum of compound **5**

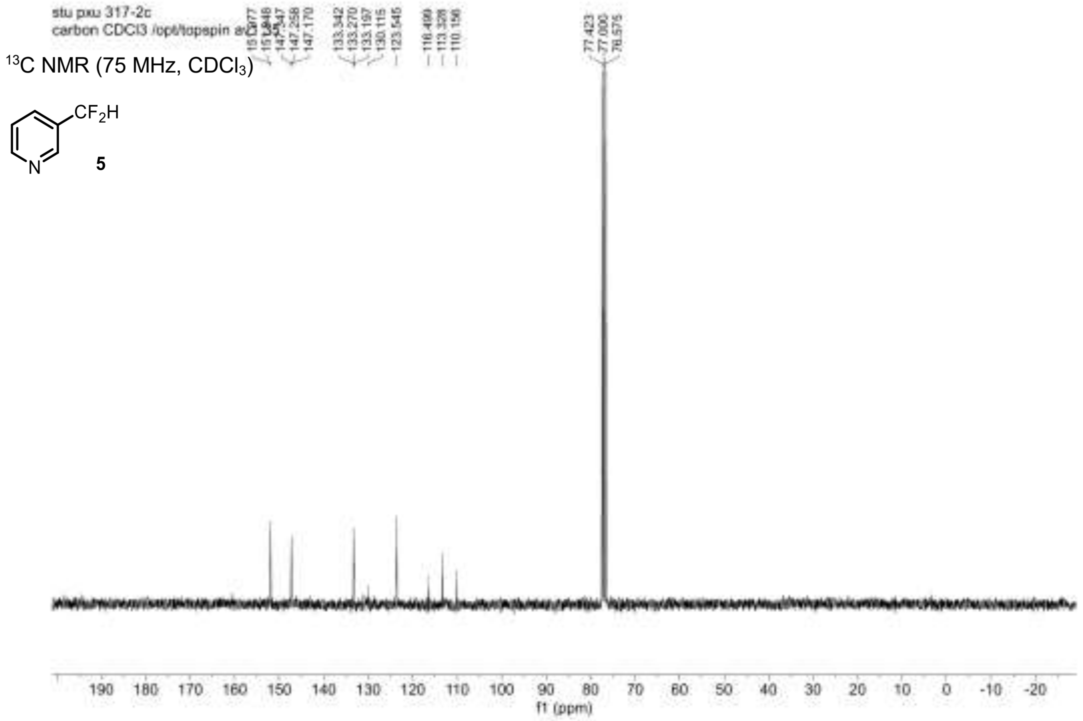

Supplementary Figure 58. ^{13}C NMR (75 MHz, $CDCl_3$) spectrum of compound **5**

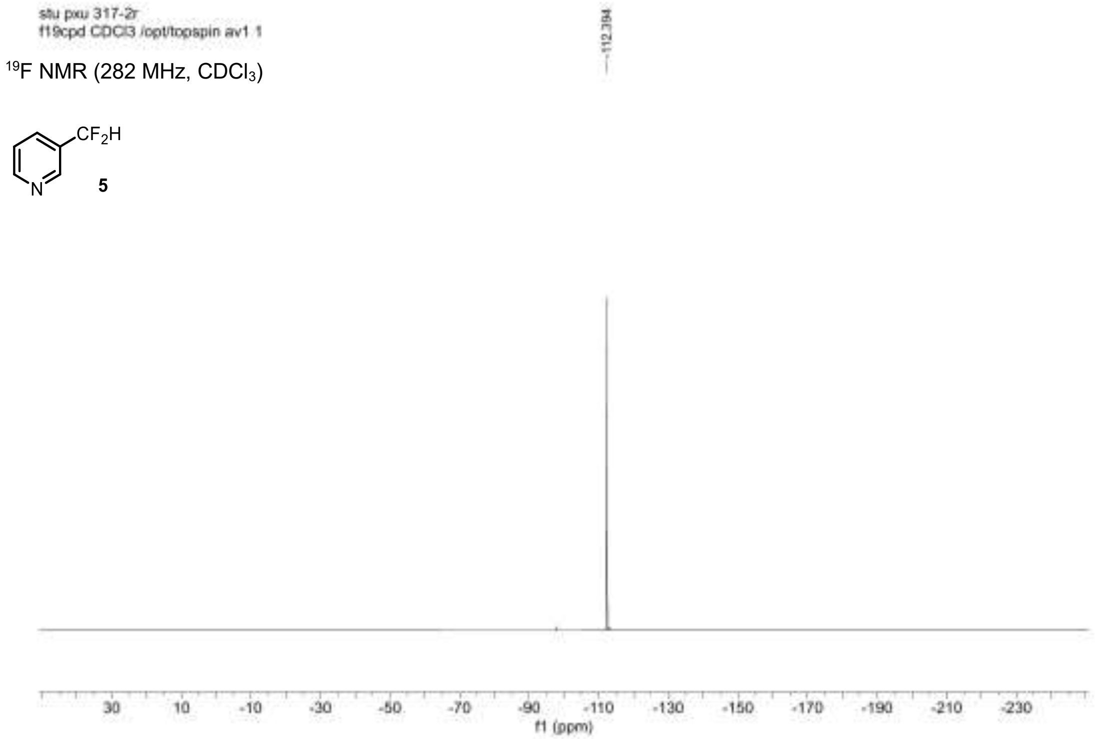

Supplementary Figure 59. ^{19}F NMR (282 MHz, $CDCl_3$) spectrum of compound **5**

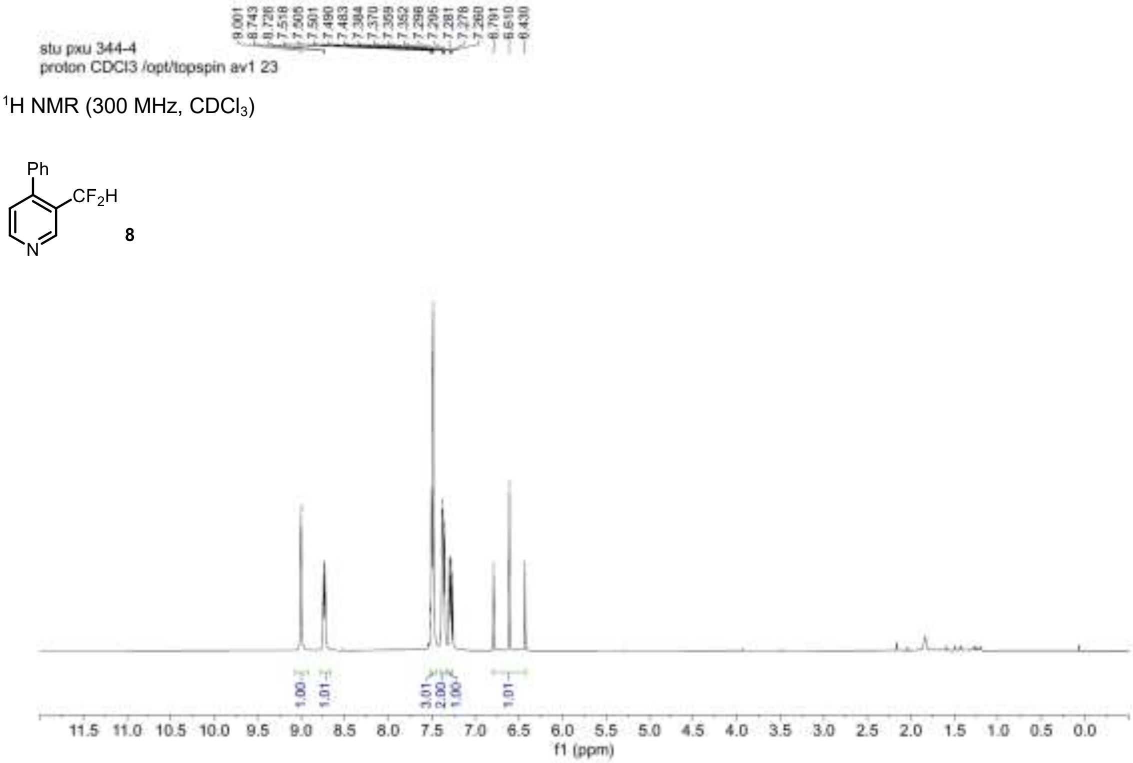

Supplementary Figure 60. ^{1}H NMR (300 MHz, $CDCl_3$) spectrum of compound **8**

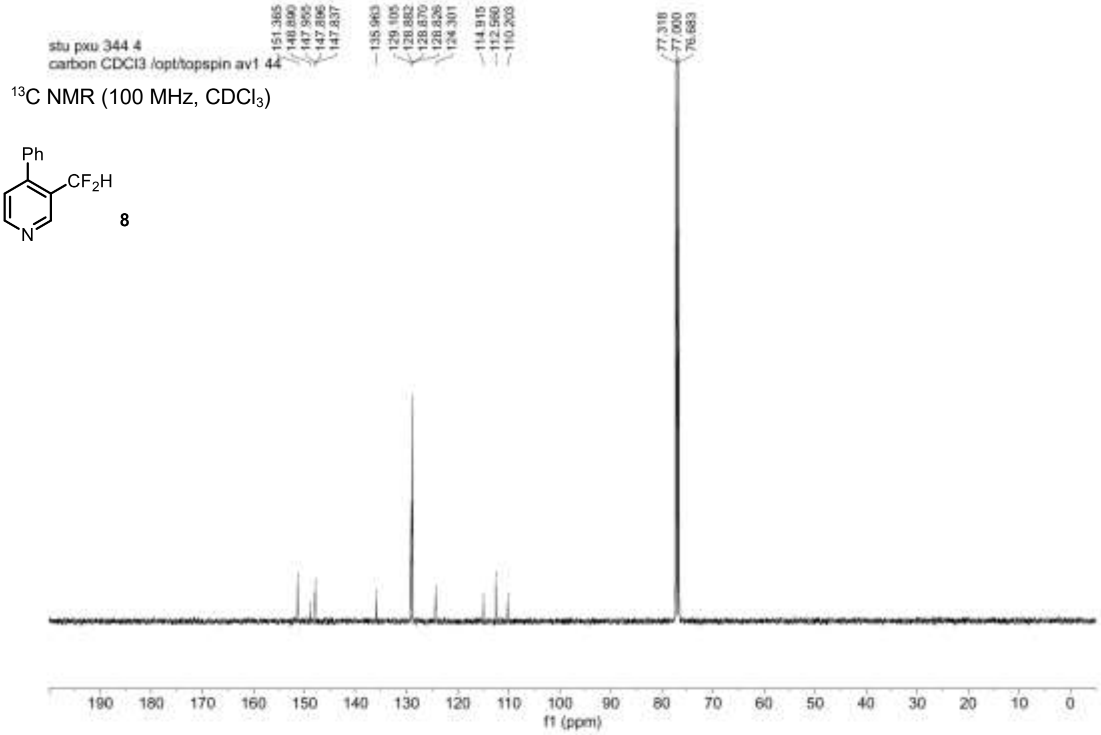

Supplementary Figure 61. ^{13}C NMR (100 MHz, $CDCl_3$) spectrum of compound **8**

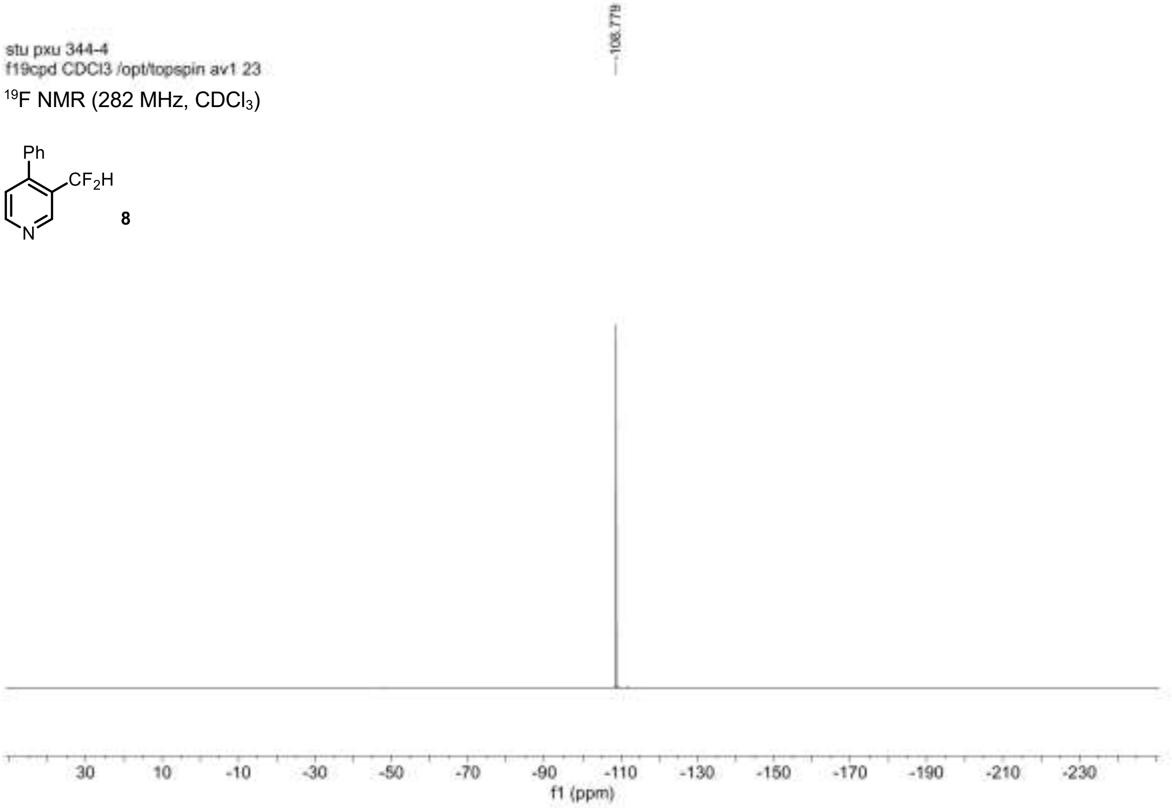

Supplementary Figure 62. ^{19}F NMR (282 MHz, $CDCl_3$) spectrum of compound **8**

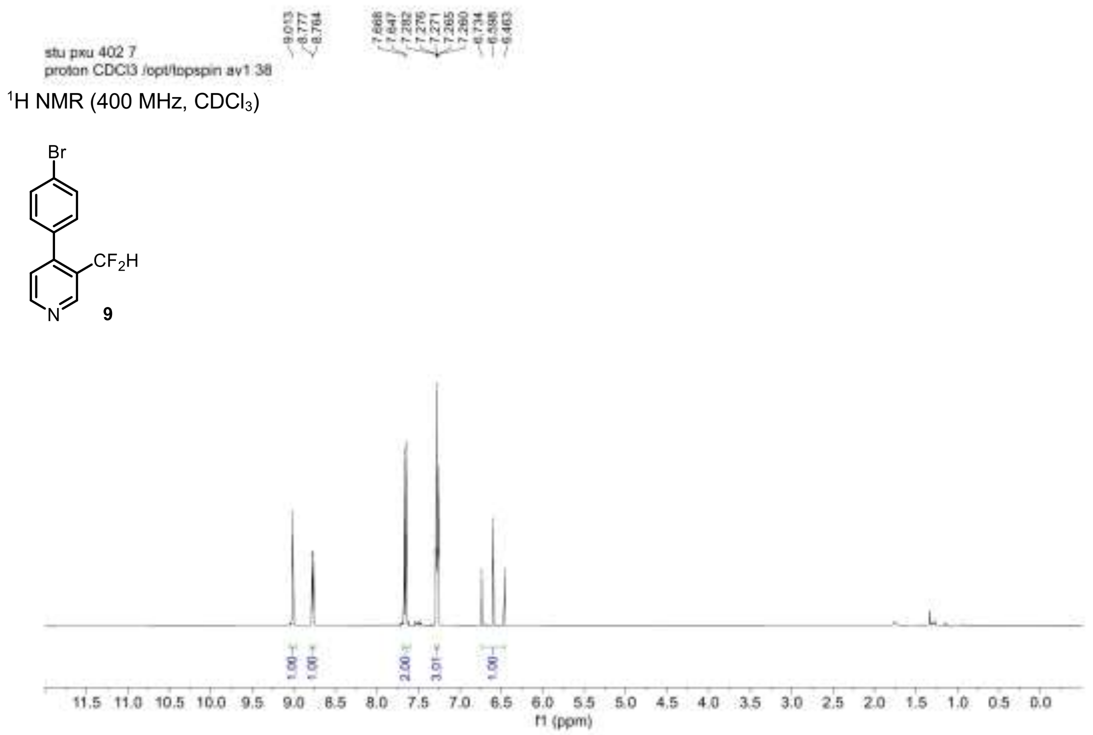

Supplementary Figure 63. ^{1}H NMR (400 MHz, $CDCl_3$) spectrum of compound **9**

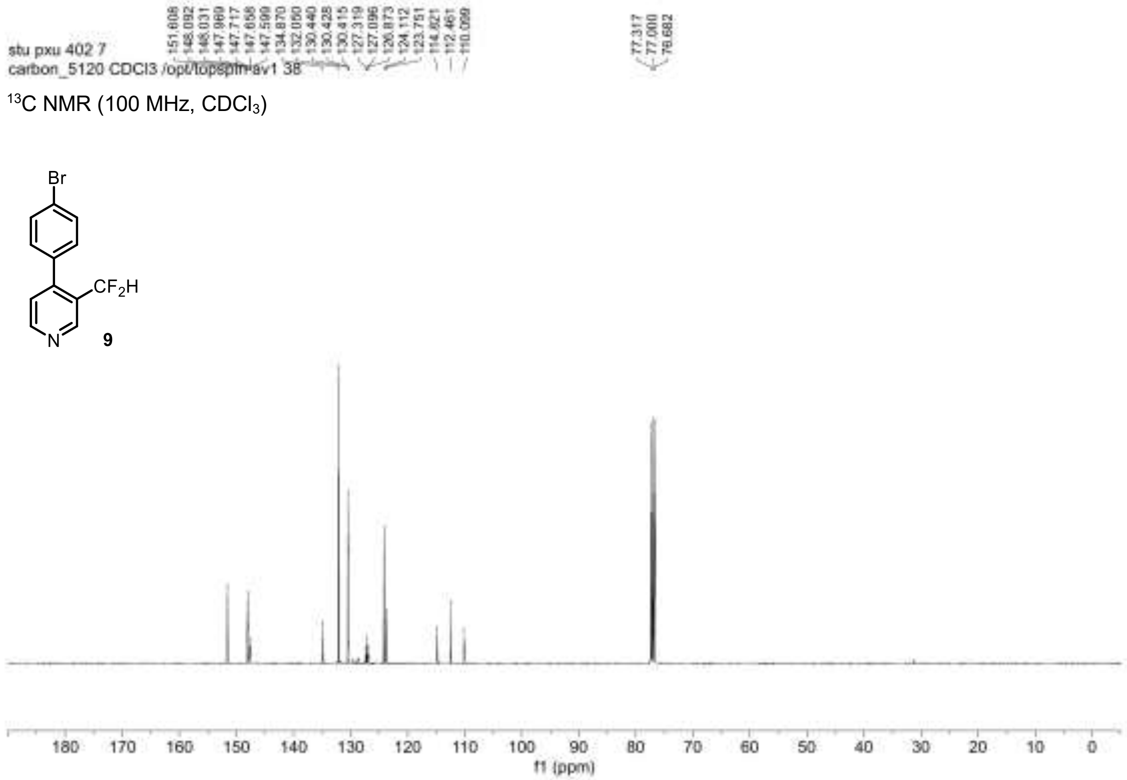

Supplementary Figure 64. ^{13}C NMR (100 MHz, $CDCl_3$) spectrum of compound **9**

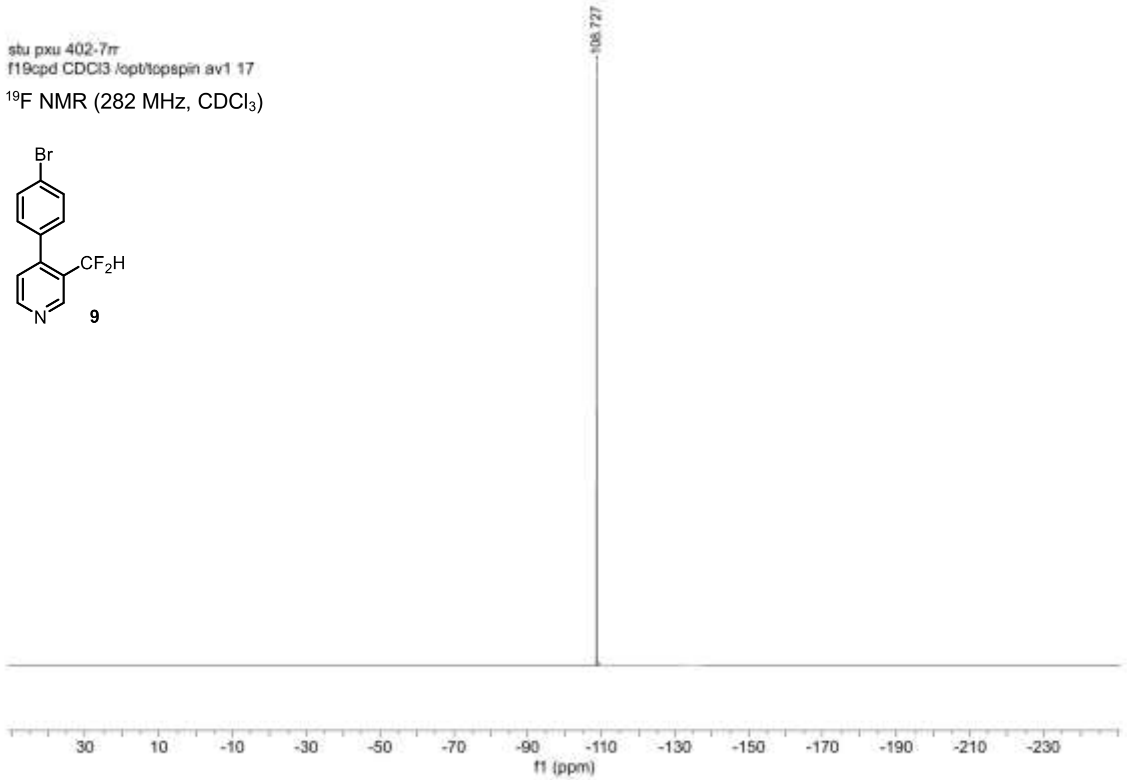

Supplementary Figure 65. ^{19}F NMR (282 MHz, $CDCl_3$) spectrum of compound **9**

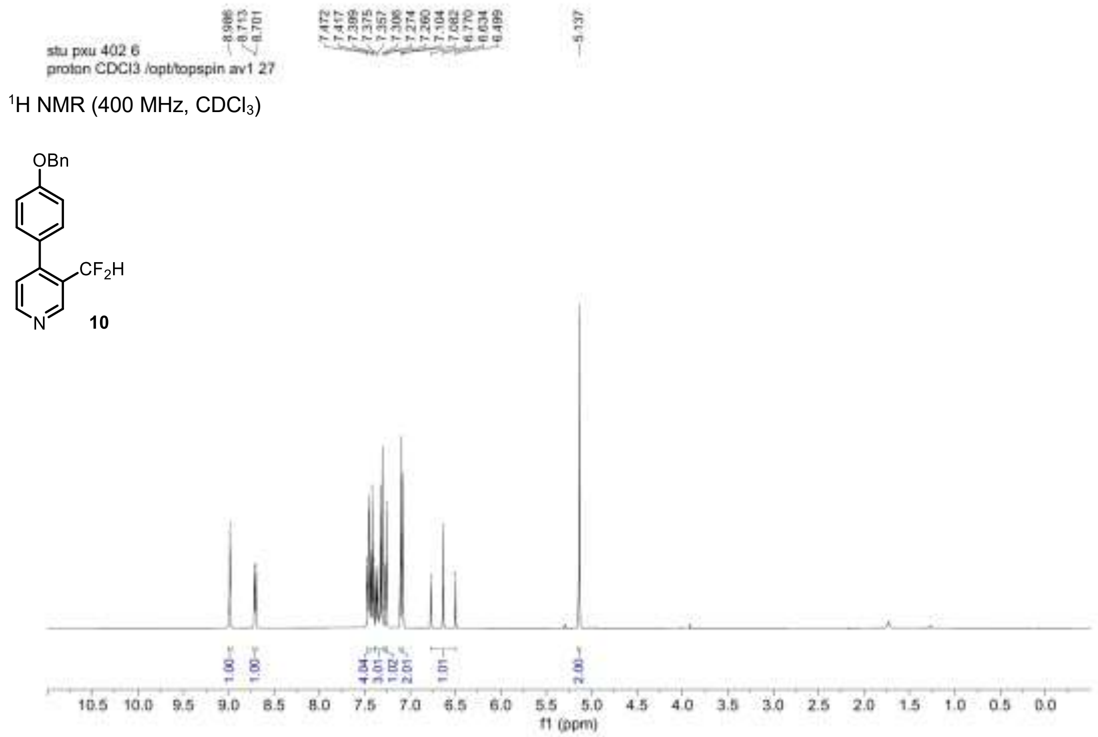

Supplementary Figure 66. ^{1}H NMR (400 MHz, $CDCl_3$) spectrum of compound **10**

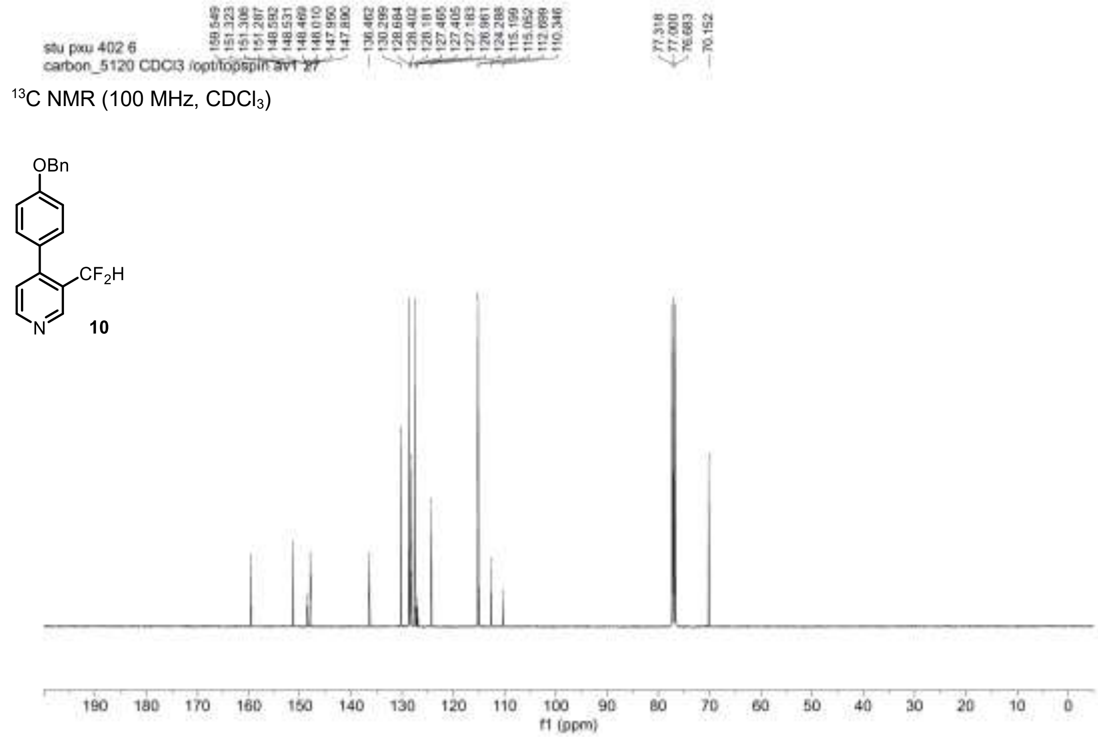

Supplementary Figure 67. ^{13}C NMR (100 MHz, $CDCl_3$) spectrum of compound **10**

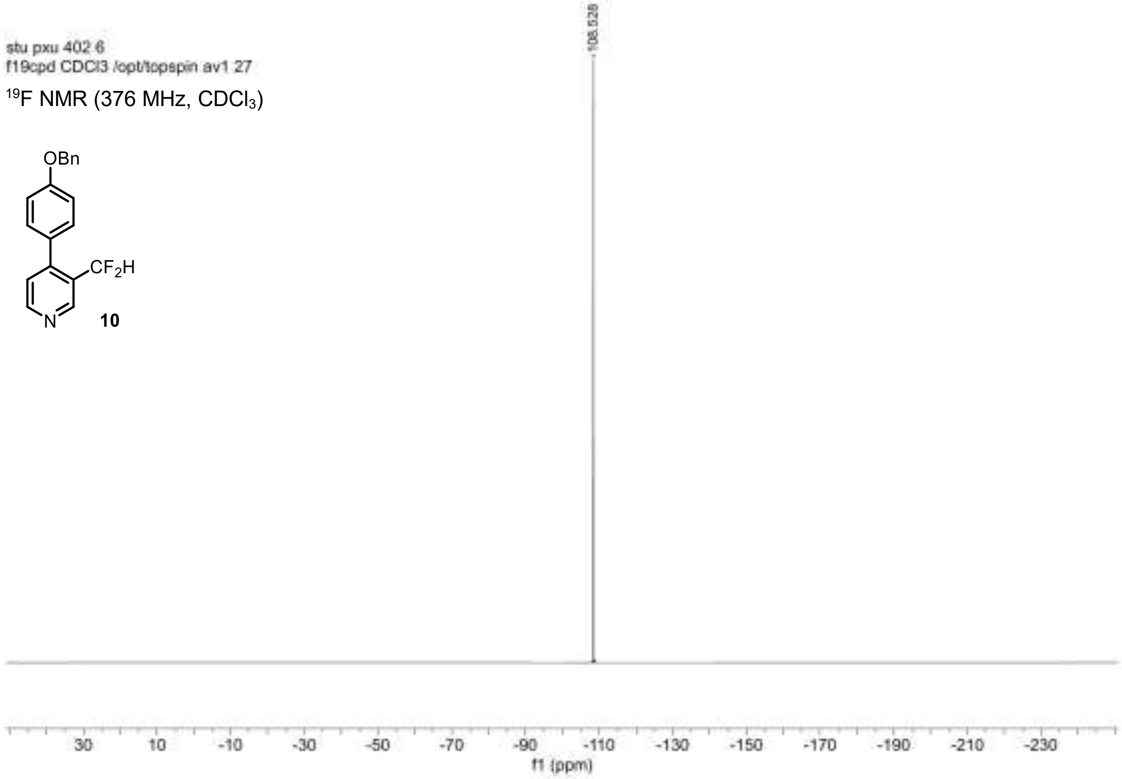

Supplementary Figure 68. ^{19}F NMR (376 MHz, $CDCl_3$) spectrum of compound **10**

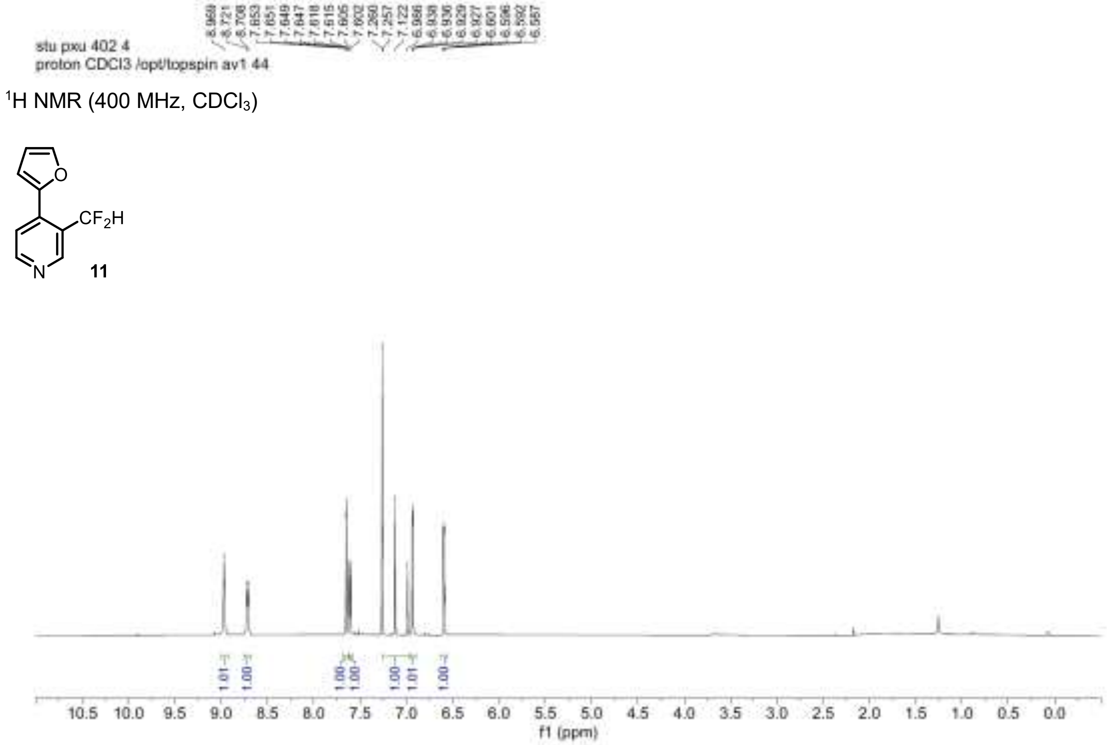

Supplementary Figure 69. ^{1}H NMR (400 MHz, $CDCl_3$) spectrum of compound **11**

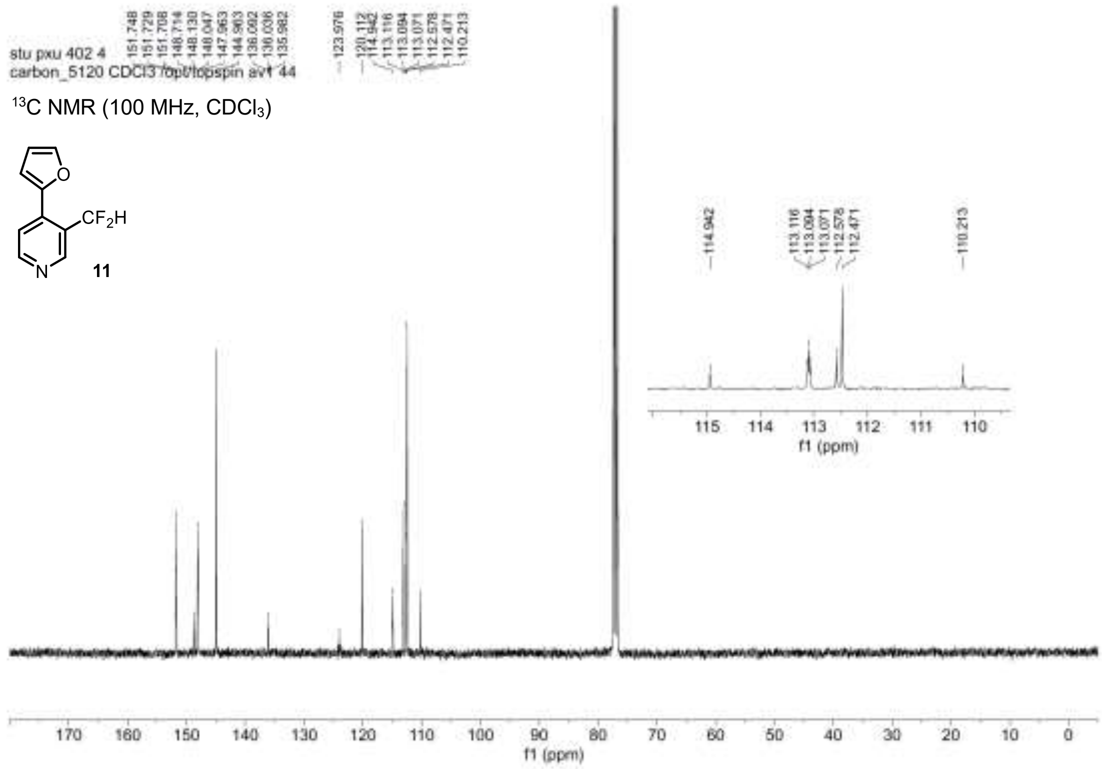

Supplementary Figure 70. ^{13}C NMR (100 MHz, $CDCl_3$) spectrum of compound **11**

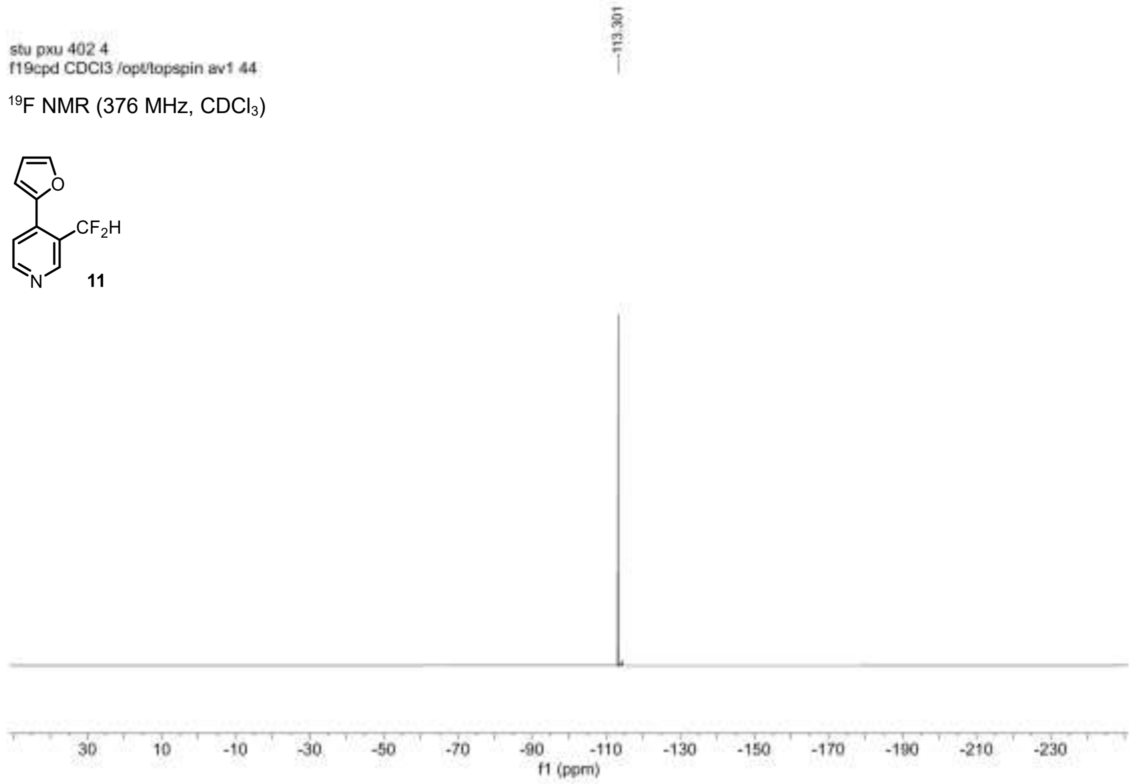

Supplementary Figure 71. ^{19}F NMR (376 MHz, $CDCl_3$) spectrum of compound **11**

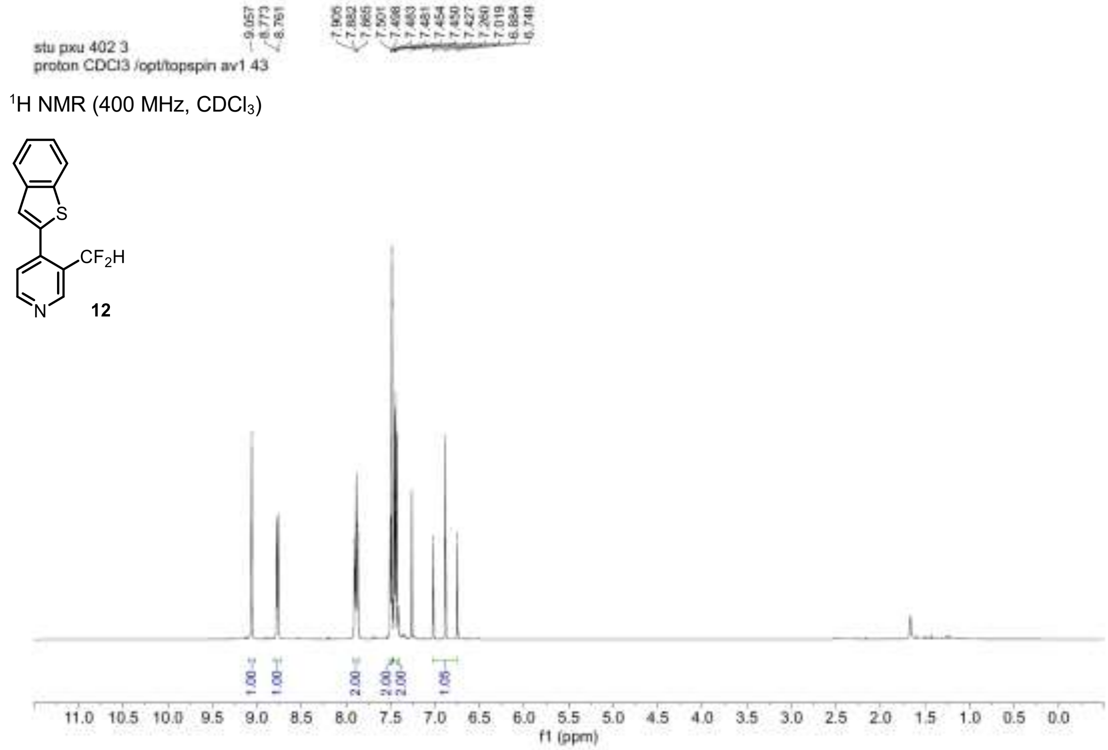

Supplementary Figure 72. ^{1}H NMR (400 MHz, $CDCl_3$) spectrum of compound **12**

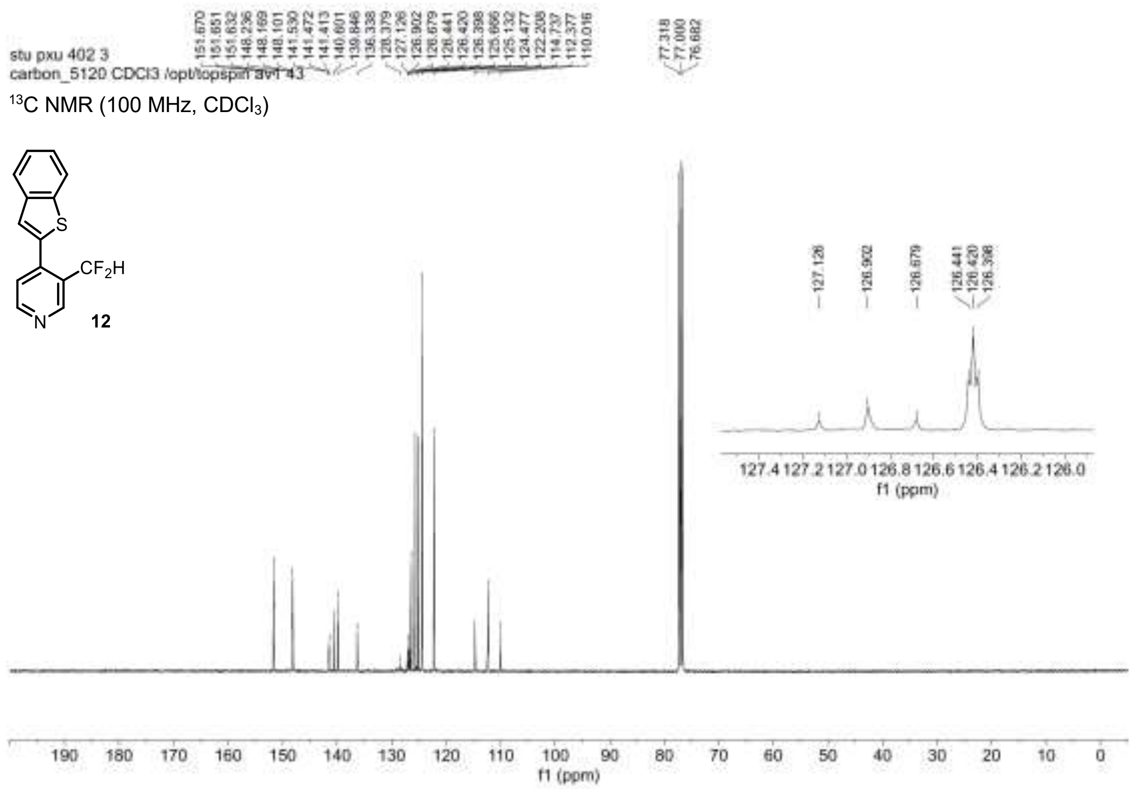

Supplementary Figure 73. ^{13}C NMR (100 MHz, $CDCl_3$) spectrum of compound **12**

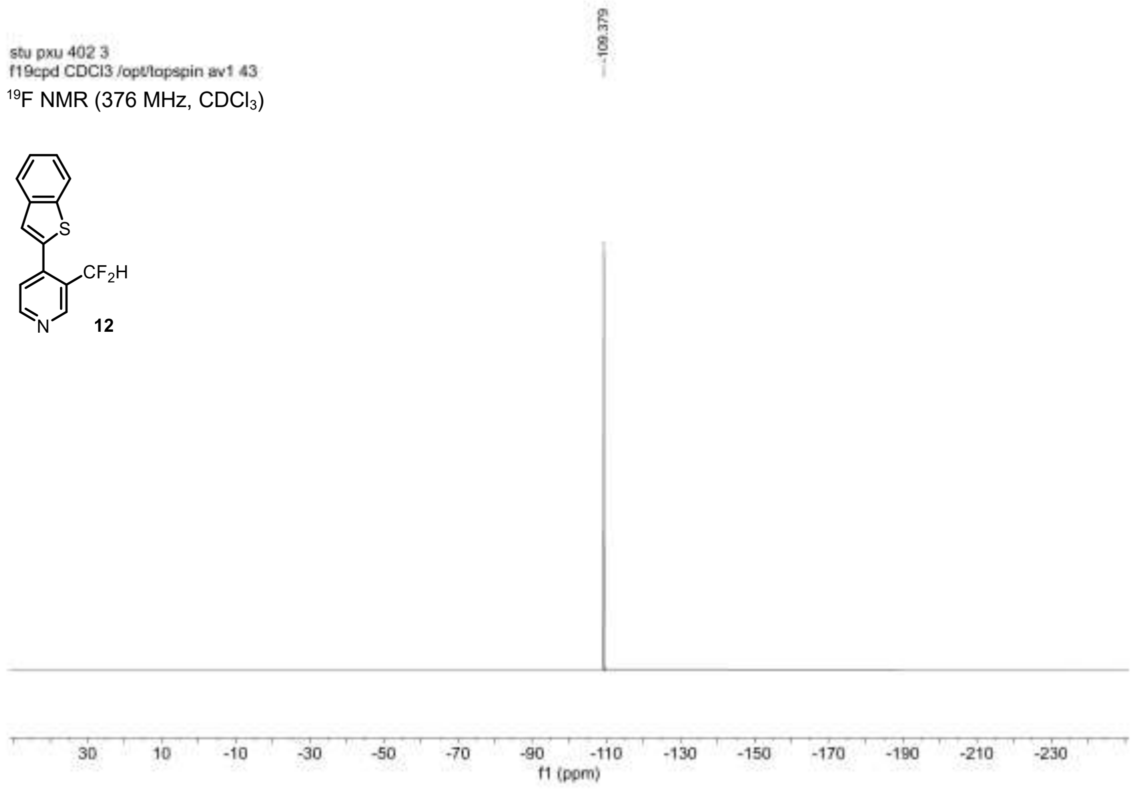

Supplementary Figure 74. ^{19}F NMR (376 MHz, $CDCl_3$) spectrum of compound **12**

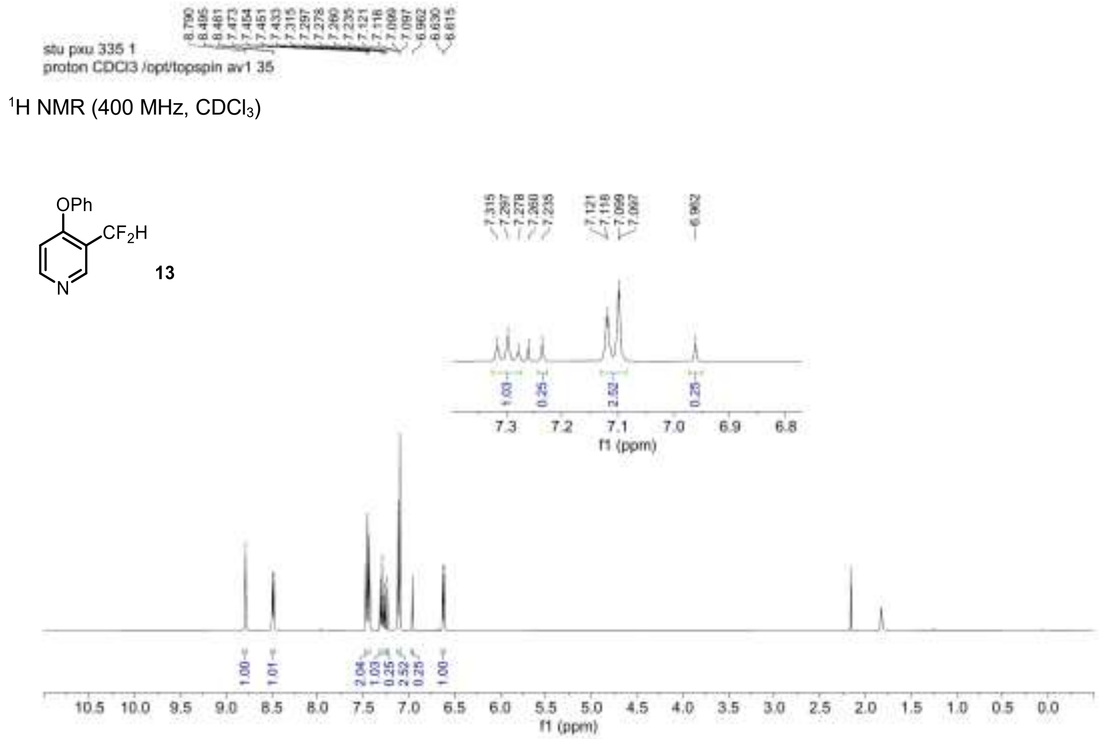

Supplementary Figure 75. ^{1}H NMR (400 MHz, $CDCl_3$) spectrum of compound **13**

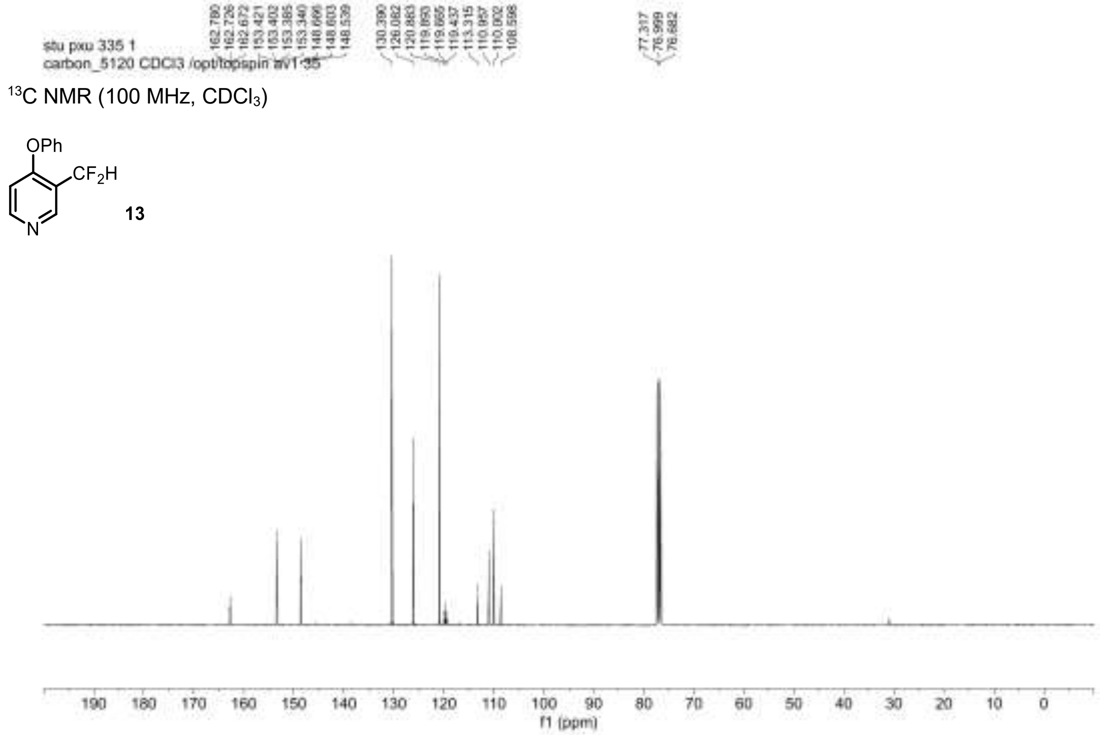

Supplementary Figure 76. ^{13}C NMR (100 MHz, $CDCl_3$) spectrum of compound **13**

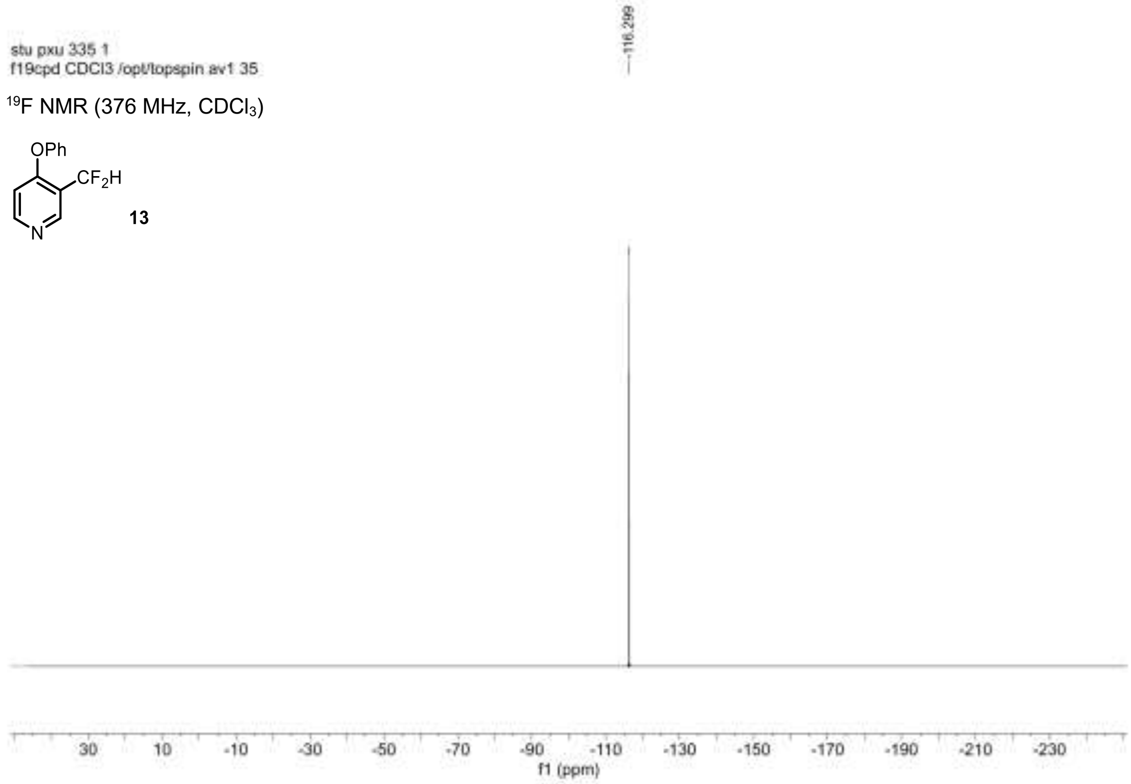

Supplementary Figure 77. ^{19}F NMR (376 MHz, $CDCl_3$) spectrum of compound **13**

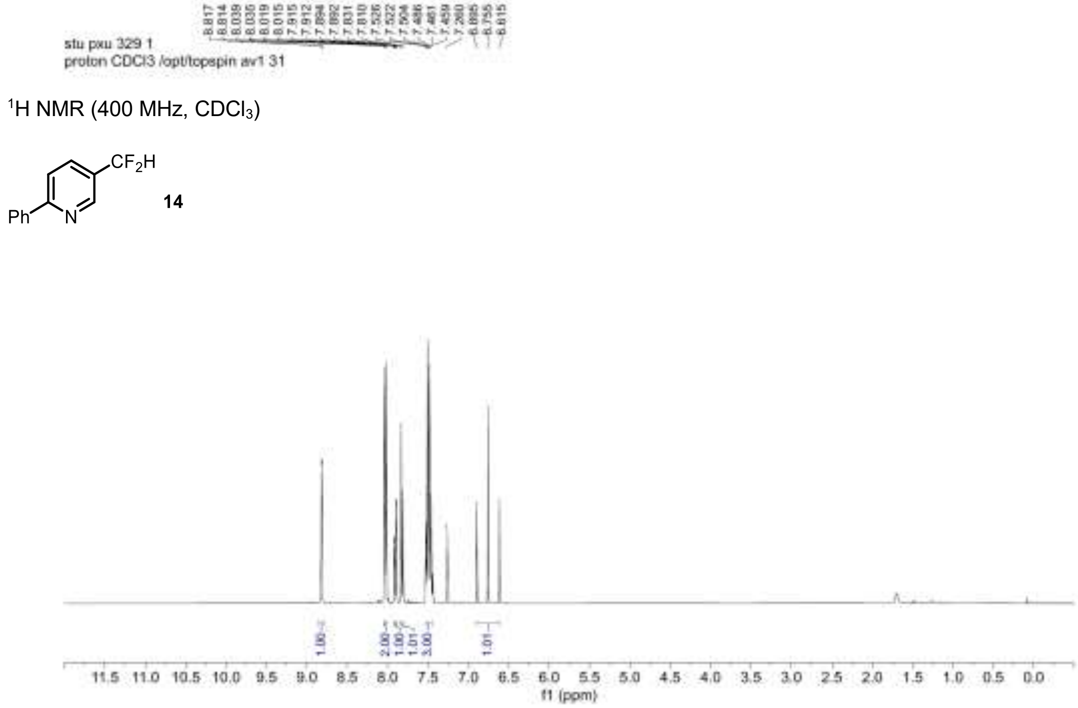

Supplementary Figure 78. ^{1}H NMR (400 MHz, $CDCl_3$) spectrum of compound **14**

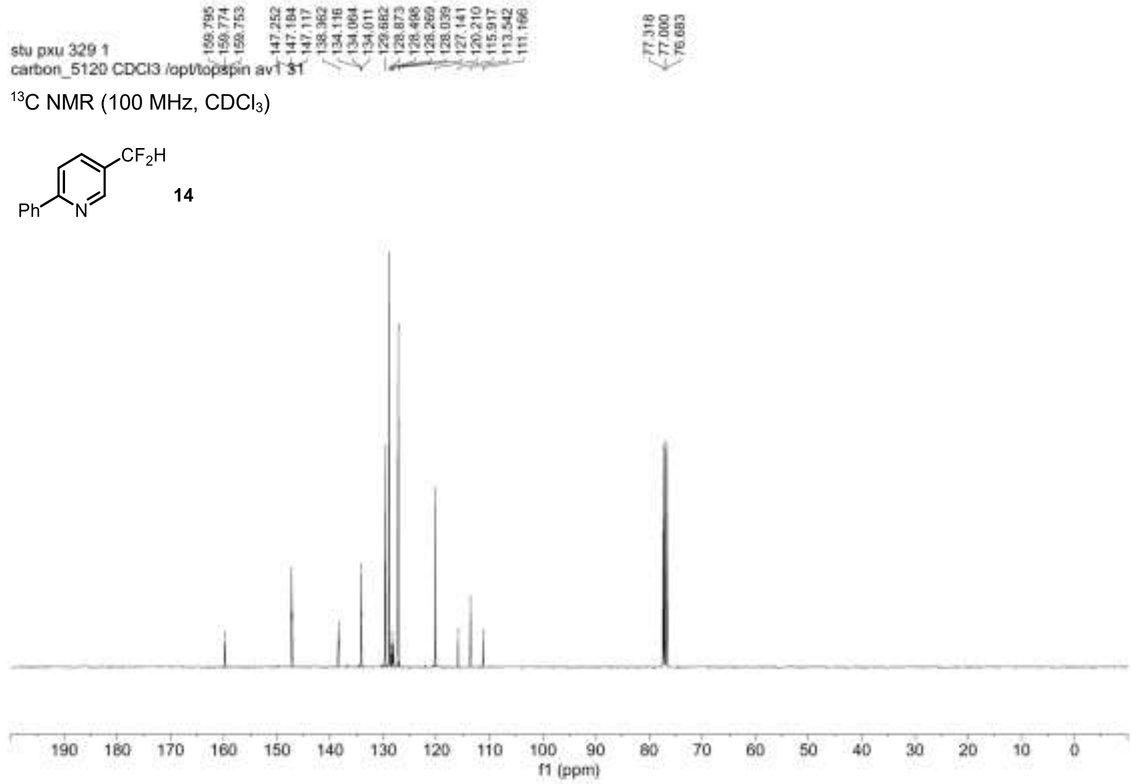

Supplementary Figure 79. ^{13}C NMR (100 MHz, $CDCl_3$) spectrum of compound **14**

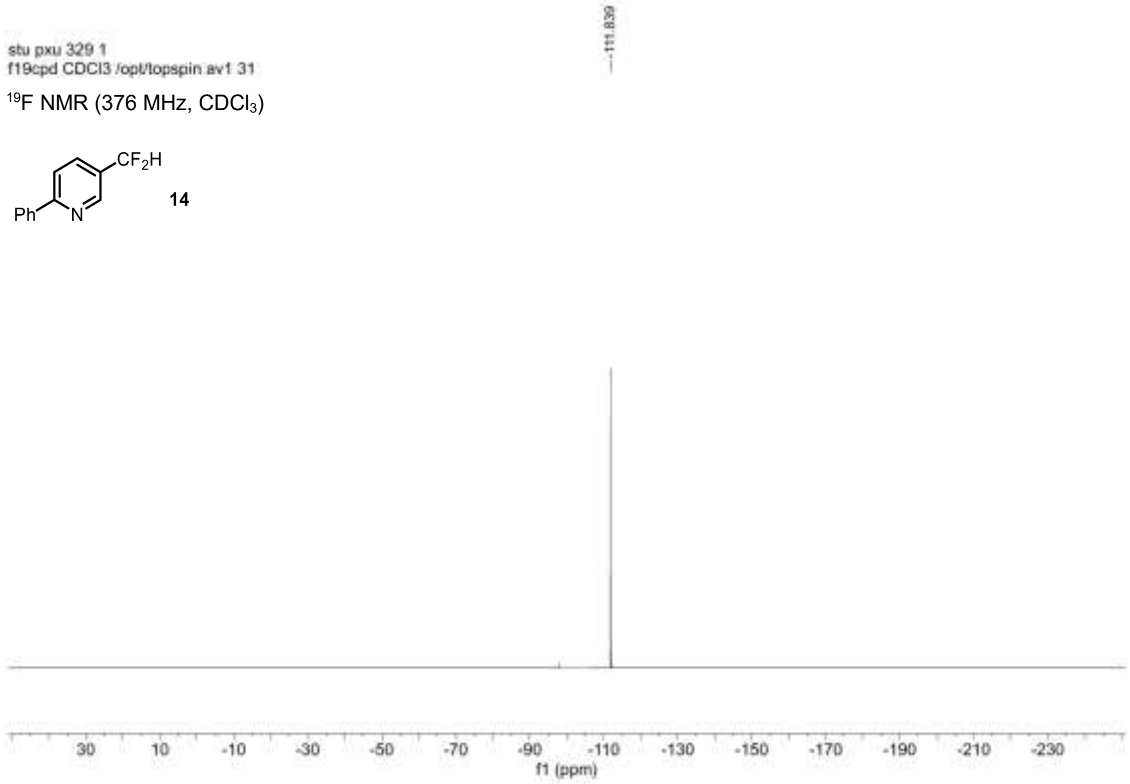

Supplementary Figure 80. ^{19}F NMR (376 MHz, $CDCl_3$) spectrum of compound **14**

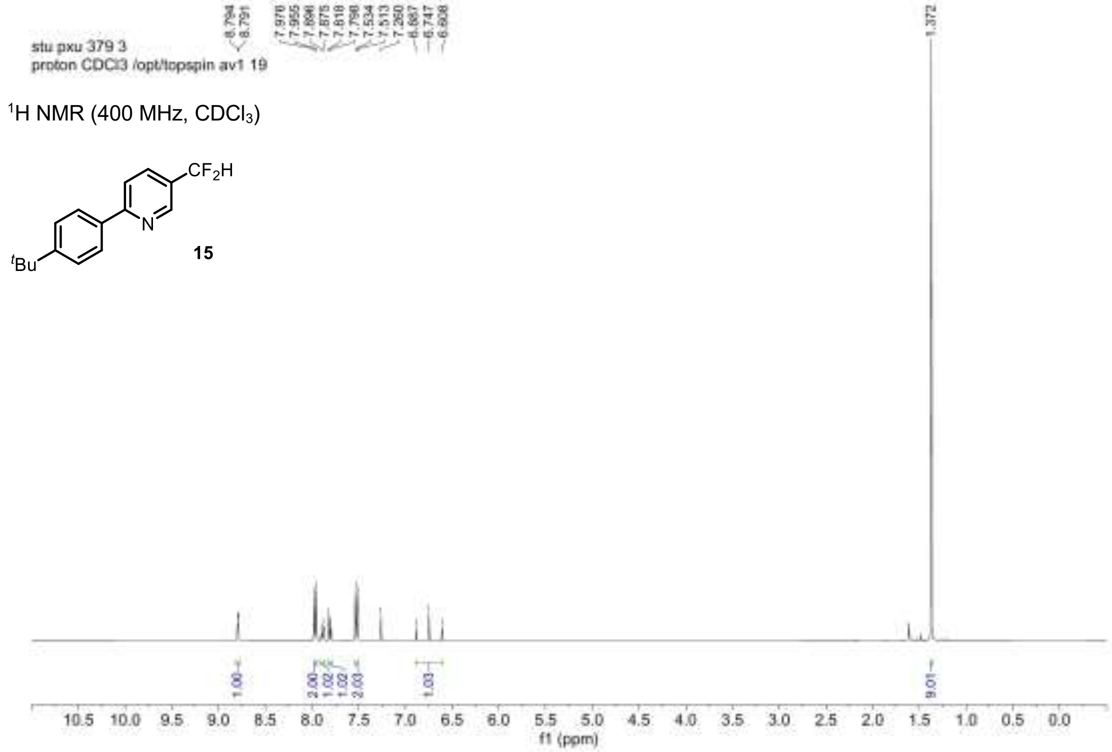

Supplementary Figure 81. ^{1}H NMR (400 MHz, $CDCl_3$) spectrum of compound **15**

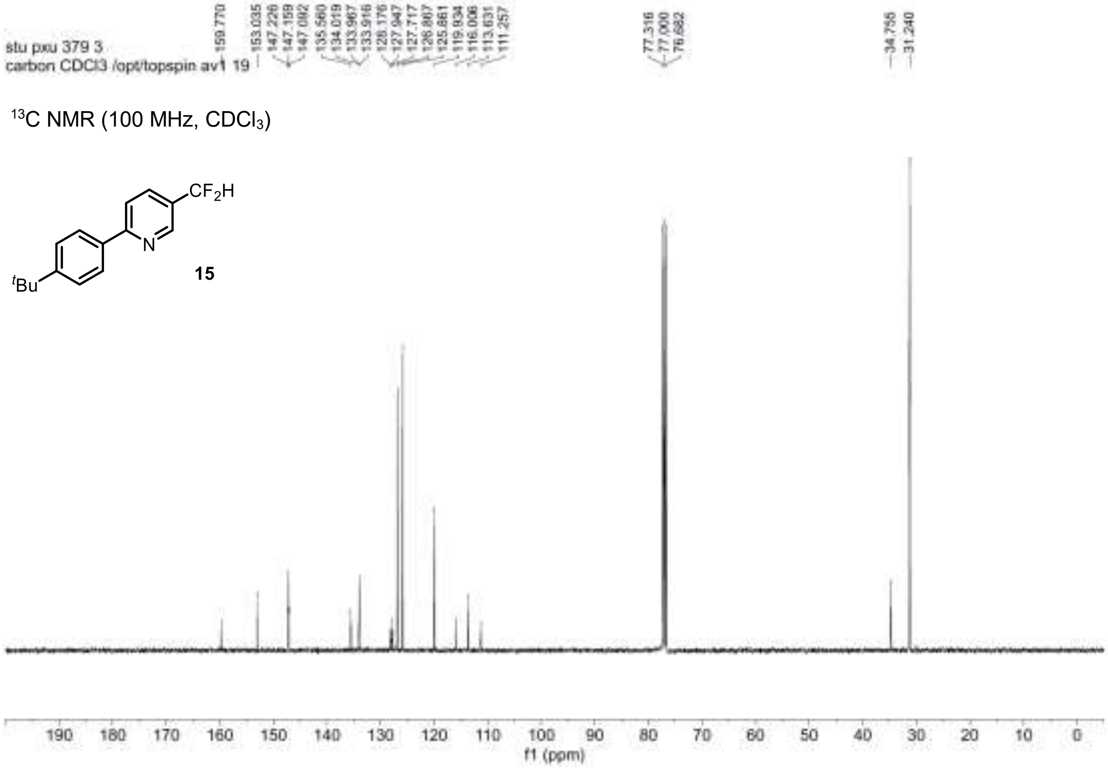

Supplementary Figure 82. ^{13}C NMR (100 MHz, $CDCl_3$) spectrum of compound **15**

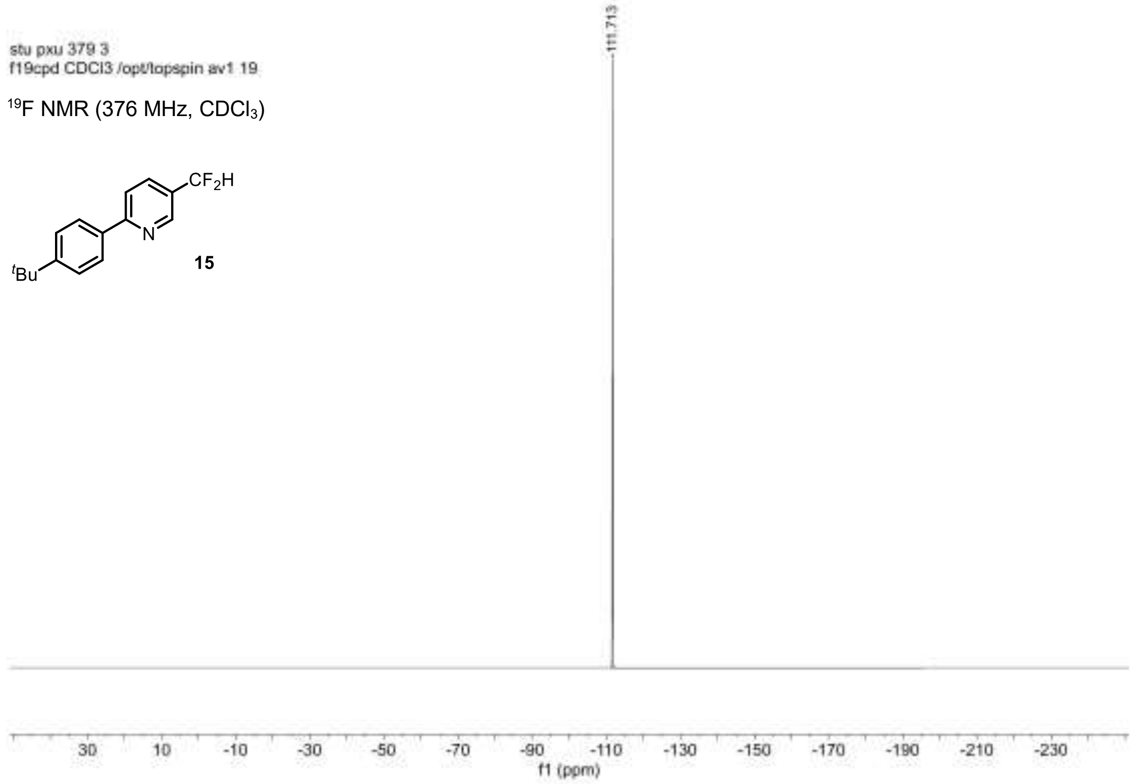

Supplementary Figure 83. ^{19}F NMR (376 MHz, $CDCl_3$) spectrum of compound **15**

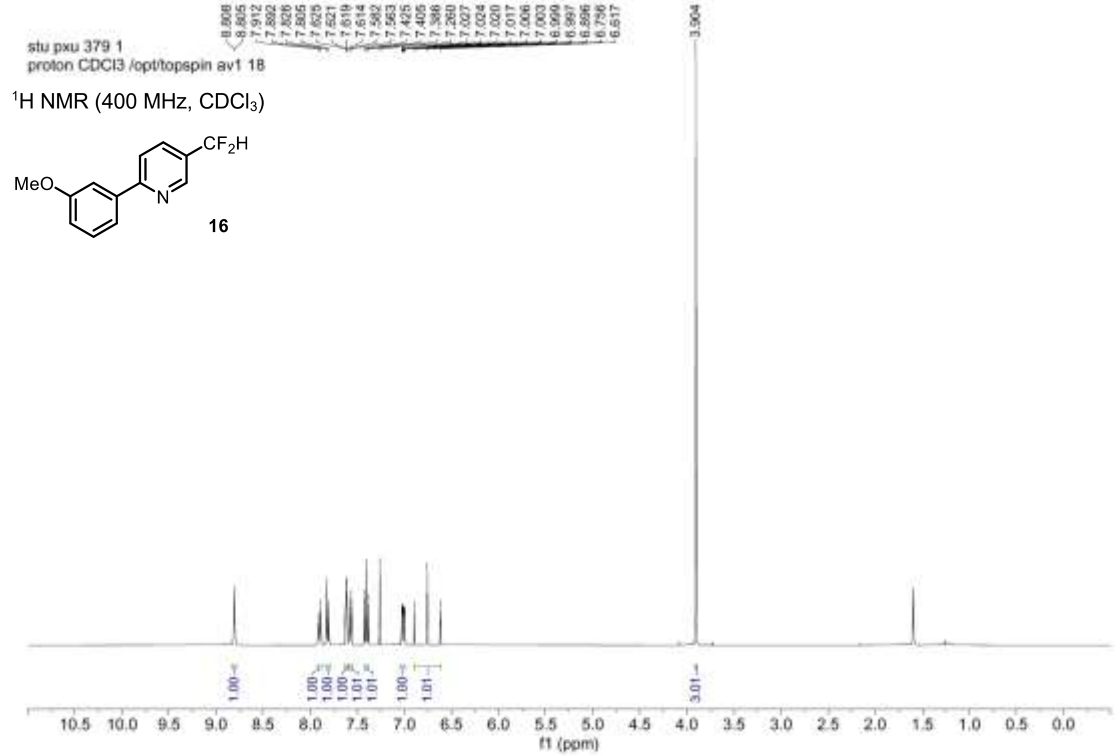

Supplementary Figure 84. ^{1}H NMR (400 MHz, $CDCl_3$) spectrum of compound **16**

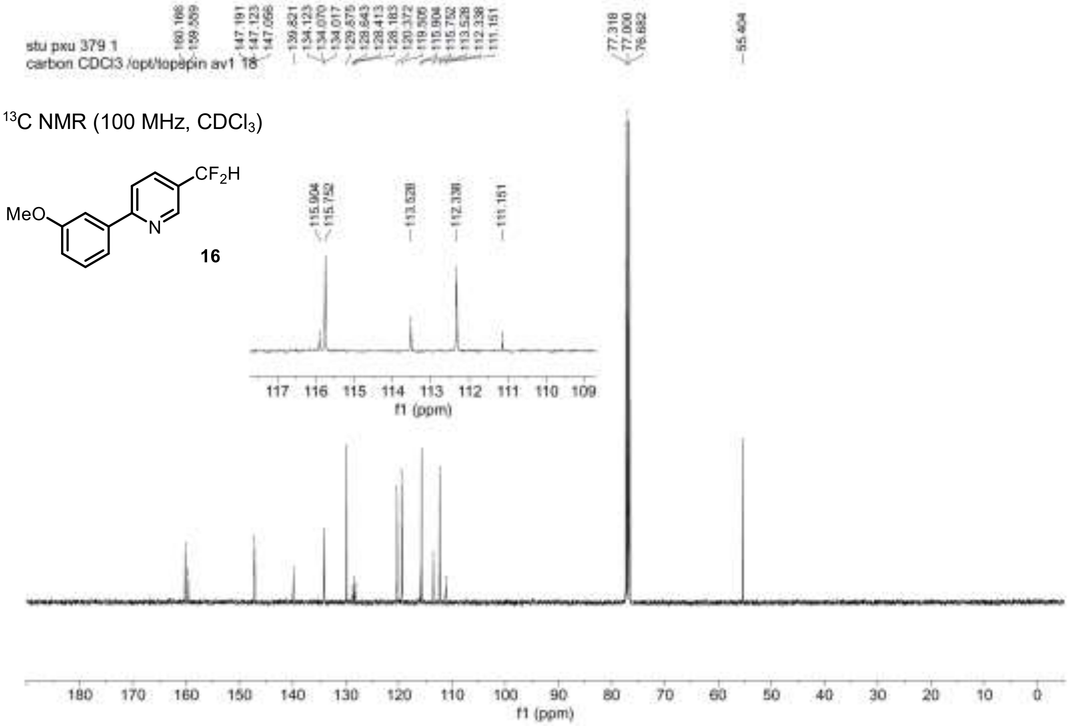

Supplementary Figure 85. ^{13}C NMR (100 MHz, $CDCl_3$) spectrum of compound **16**

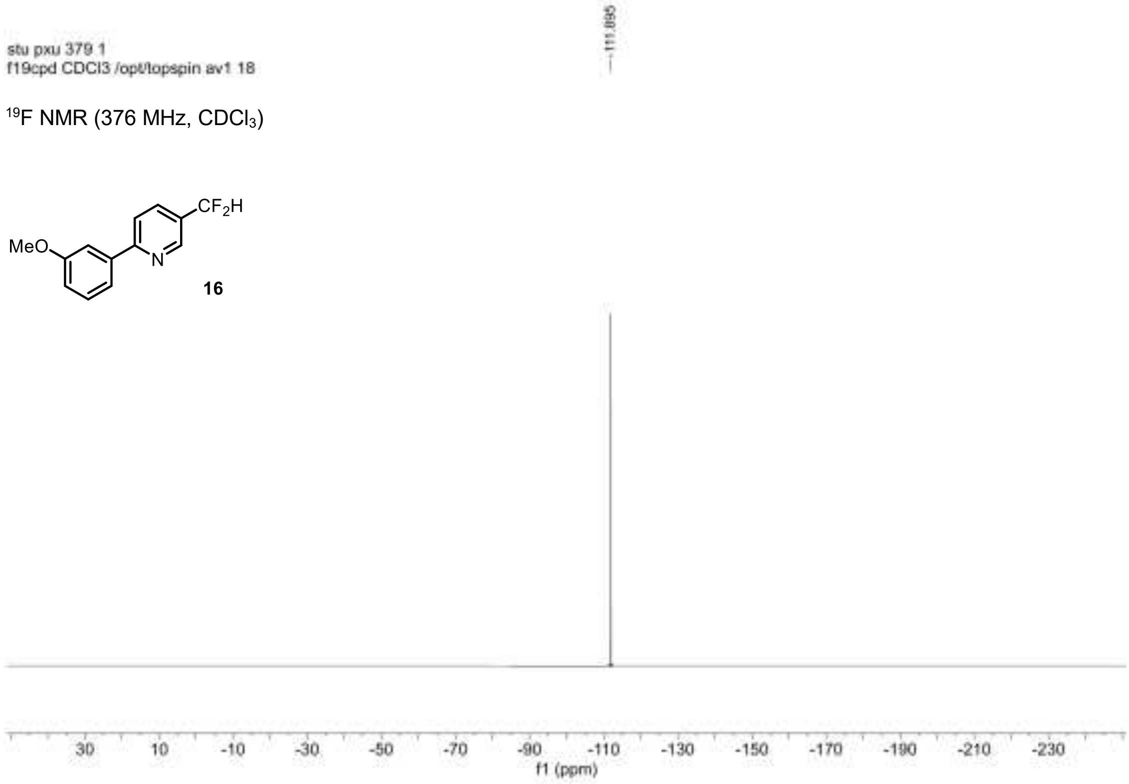

Supplementary Figure 86. ^{19}F NMR (376 MHz, $CDCl_3$) spectrum of compound **16**

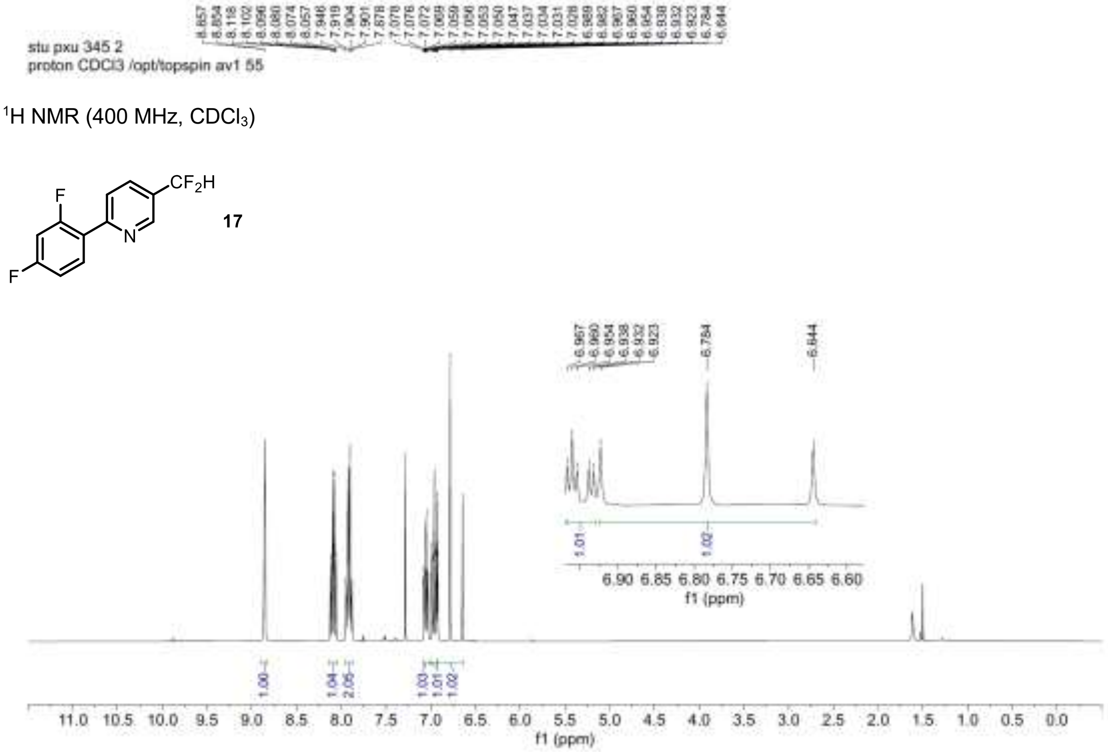

Supplementary Figure 87. ^{1}H NMR (400 MHz, $CDCl_3$) spectrum of compound **17**

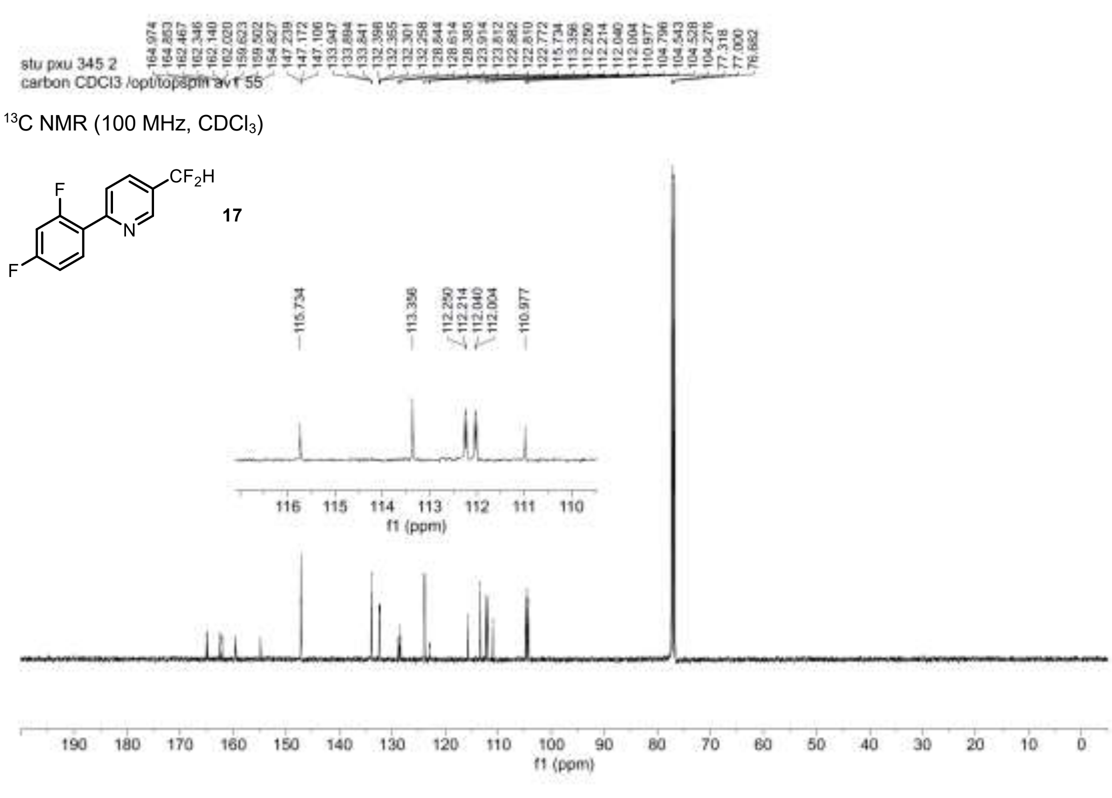

Supplementary Figure 88. ^{13}C NMR (100 MHz, $CDCl_3$) spectrum of compound **17**

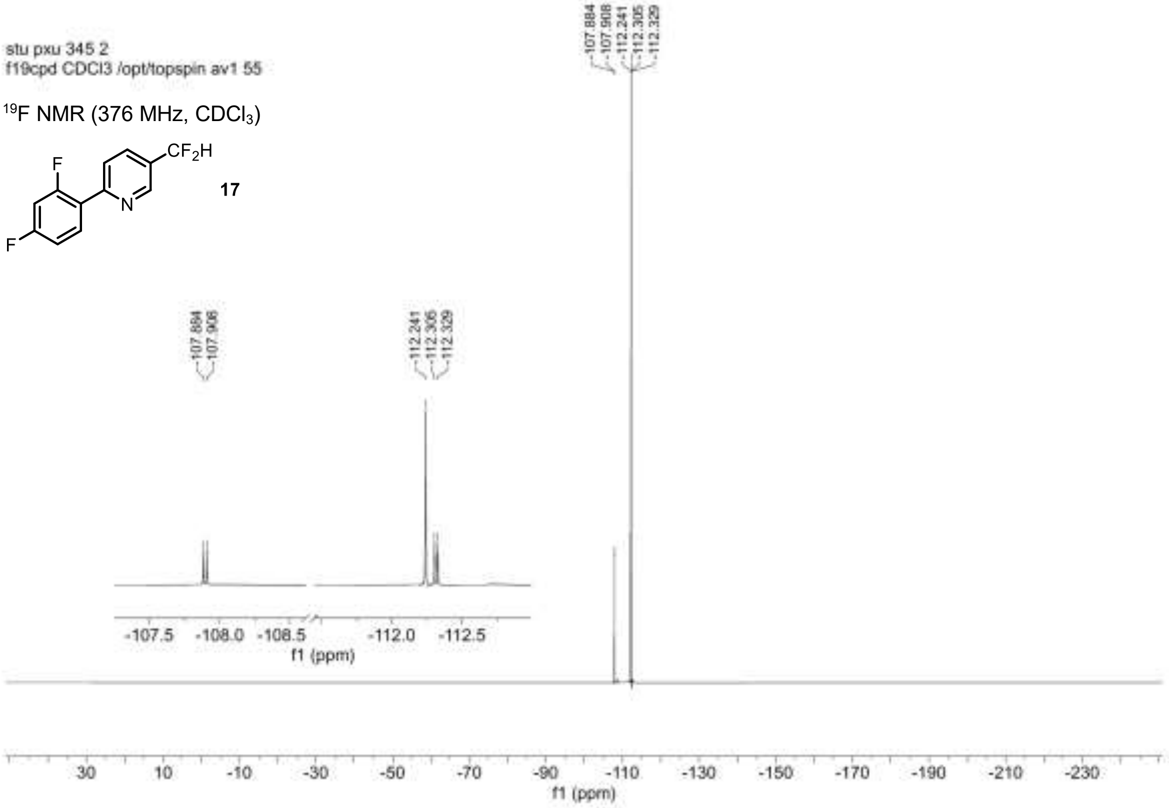

Supplementary Figure 89. ^{19}F NMR (376 MHz, $CDCl_3$) spectrum of compound **17**

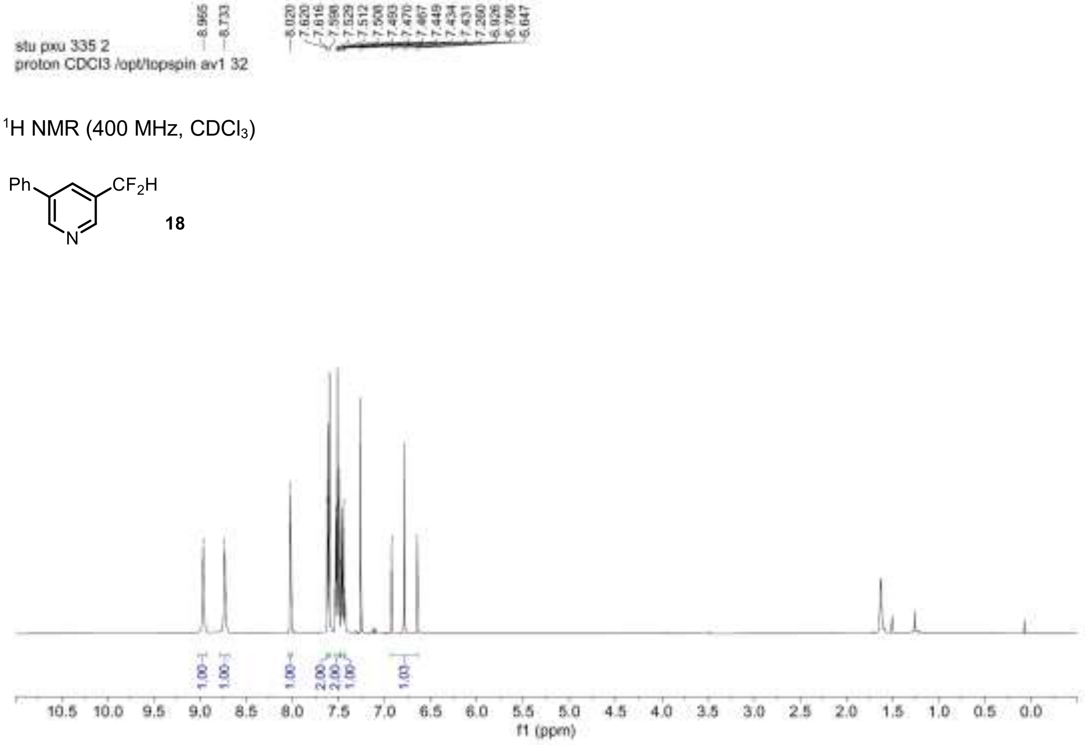

Supplementary Figure 90. ^{1}H NMR (400 MHz, $CDCl_3$) spectrum of compound **18**

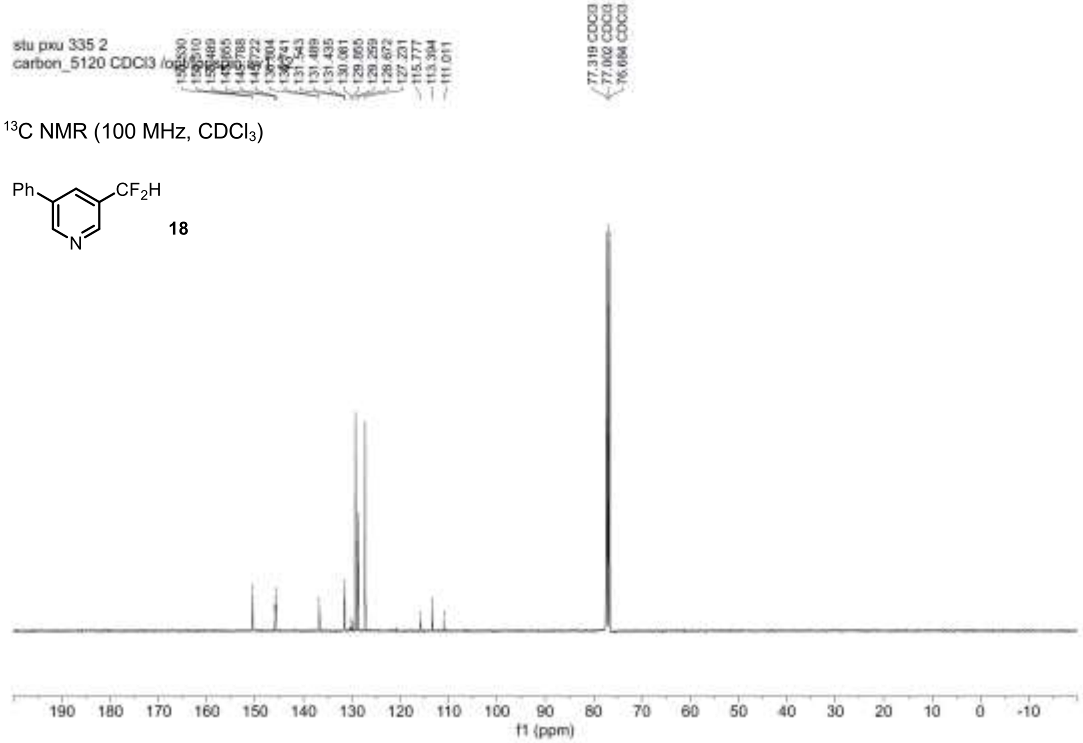

Supplementary Figure 91. ^{13}C NMR (100 MHz, $CDCl_3$) spectrum of compound **18**

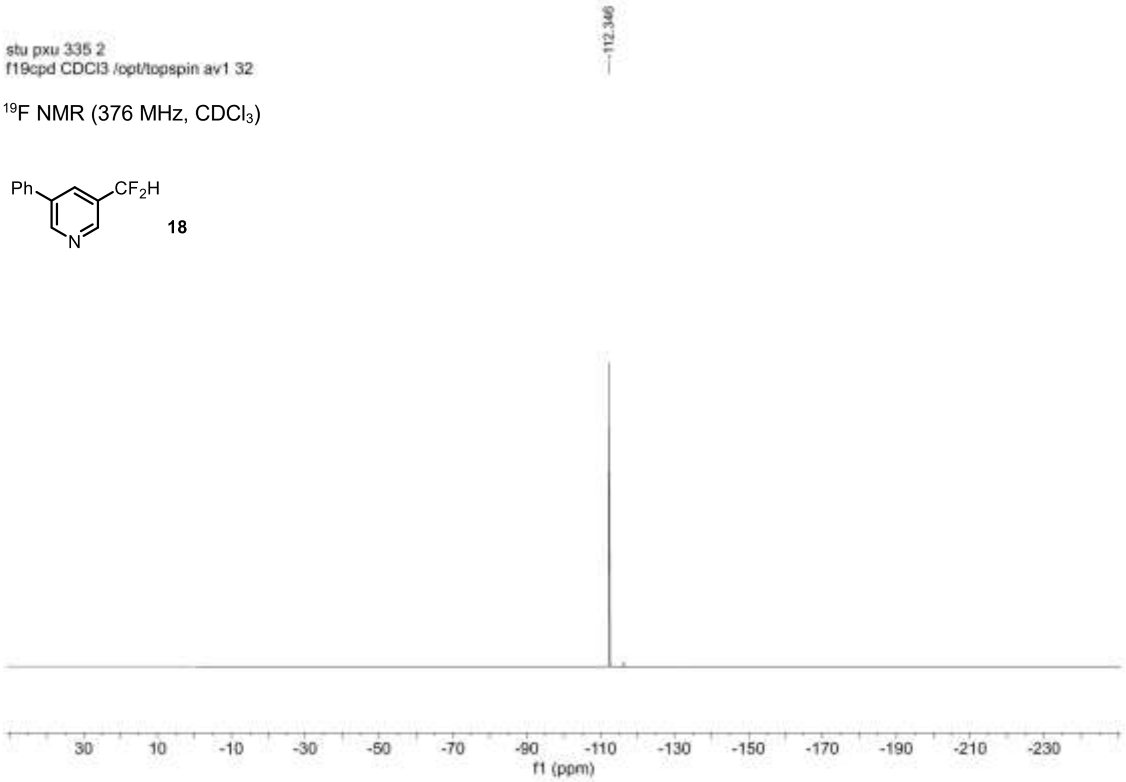

Supplementary Figure 92. ^{19}F NMR (376 MHz, $CDCl_3$) spectrum of compound **18**

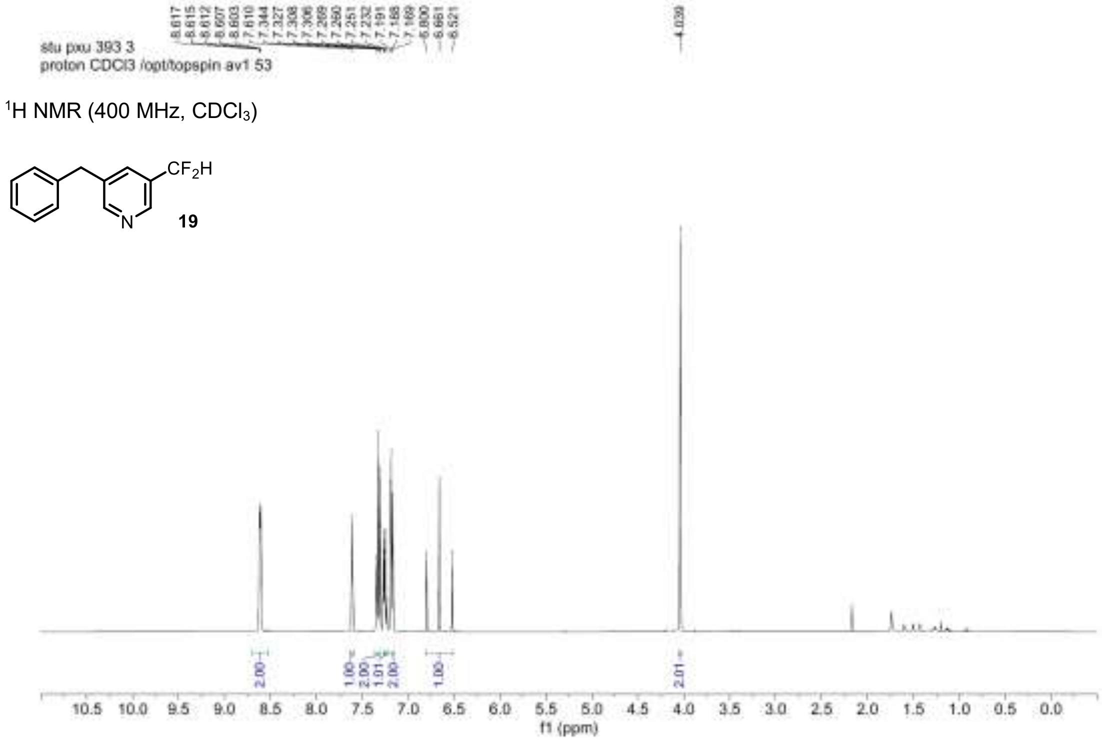

Supplementary Figure 93. ^{1}H NMR (400 MHz, $CDCl_3$) spectrum of compound **19**

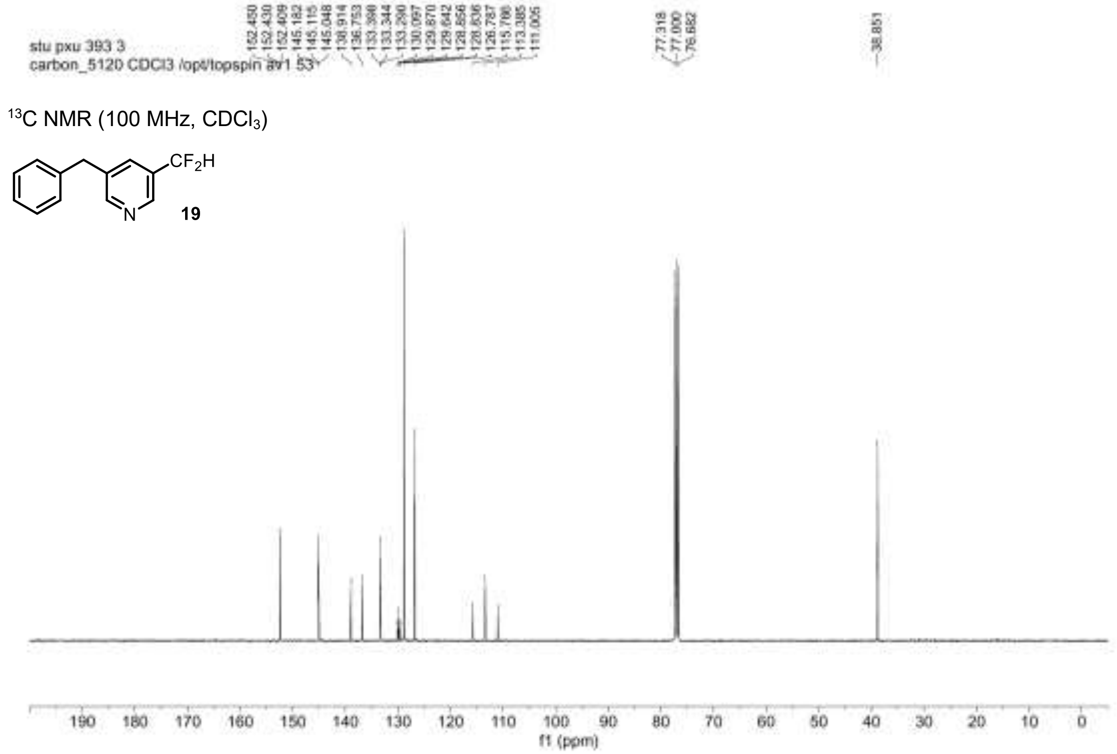

Supplementary Figure 94. ^{13}C NMR (100 MHz, $CDCl_3$) spectrum of compound **19**

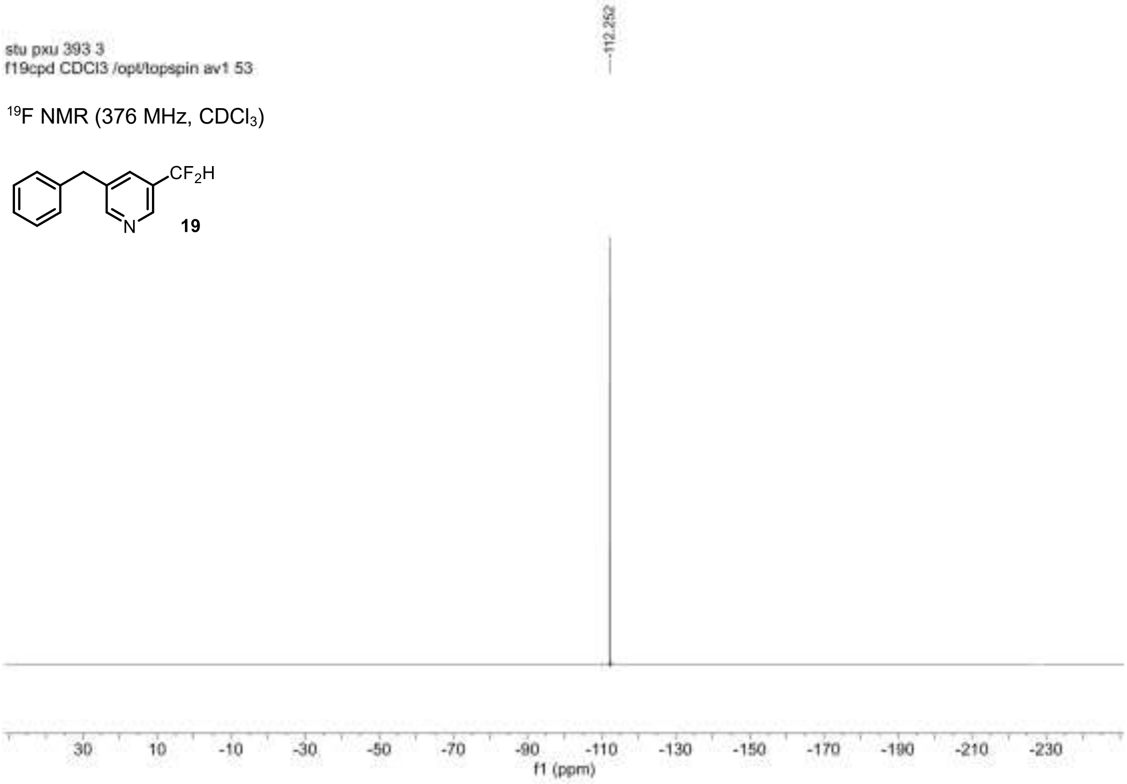

Supplementary Figure 95. ^{19}F NMR (376 MHz, $CDCl_3$) spectrum of compound **19**

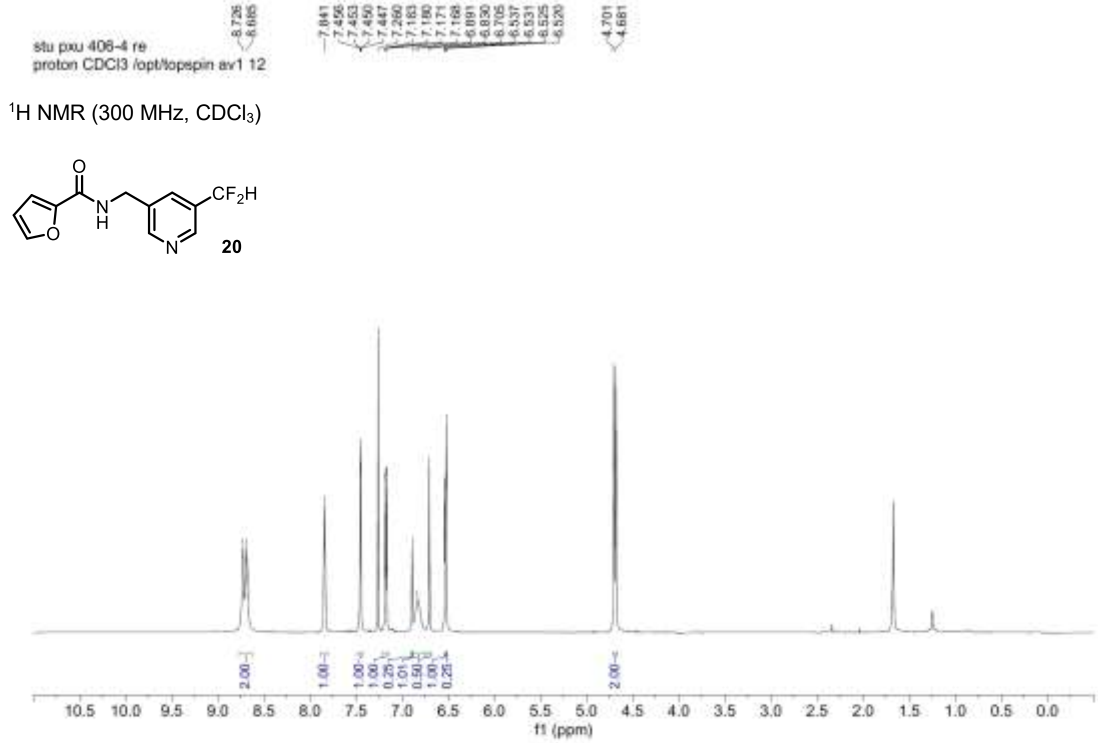

Supplementary Figure 96. ^{1}H NMR (400 MHz, $CDCl_3$) spectrum of compound **20**

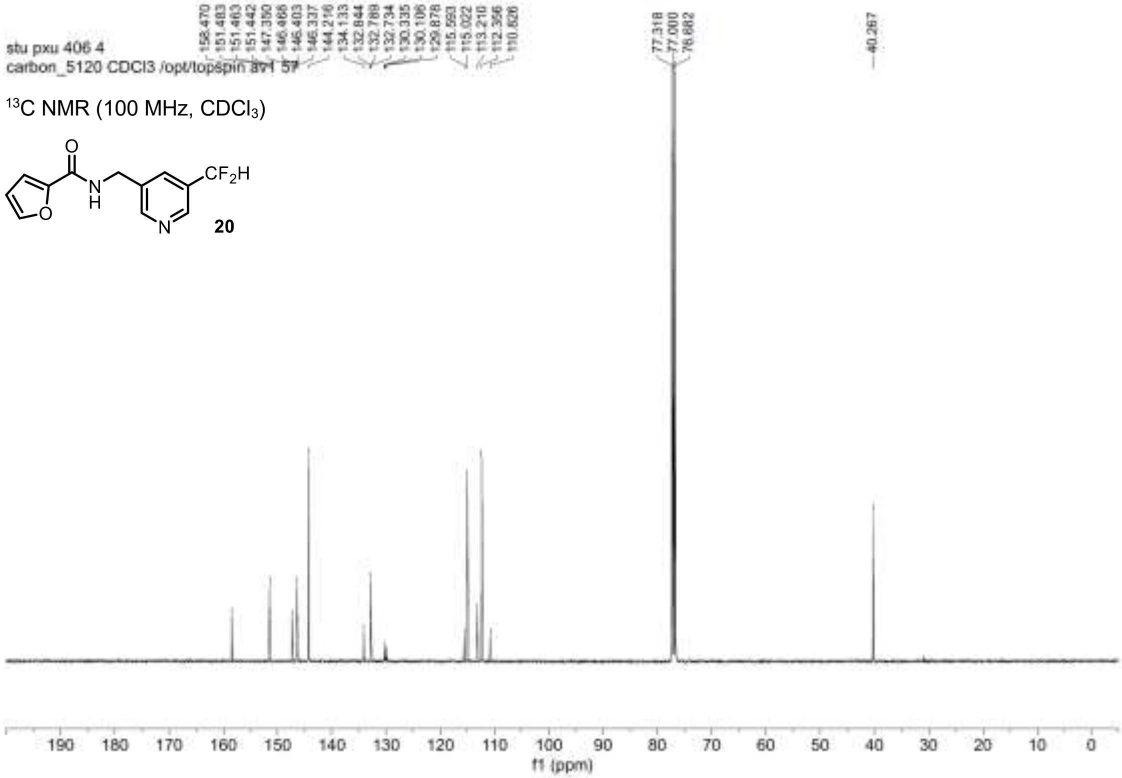

Supplementary Figure 97. ^{13}C NMR (100 MHz, $CDCl_3$) spectrum of compound **20**

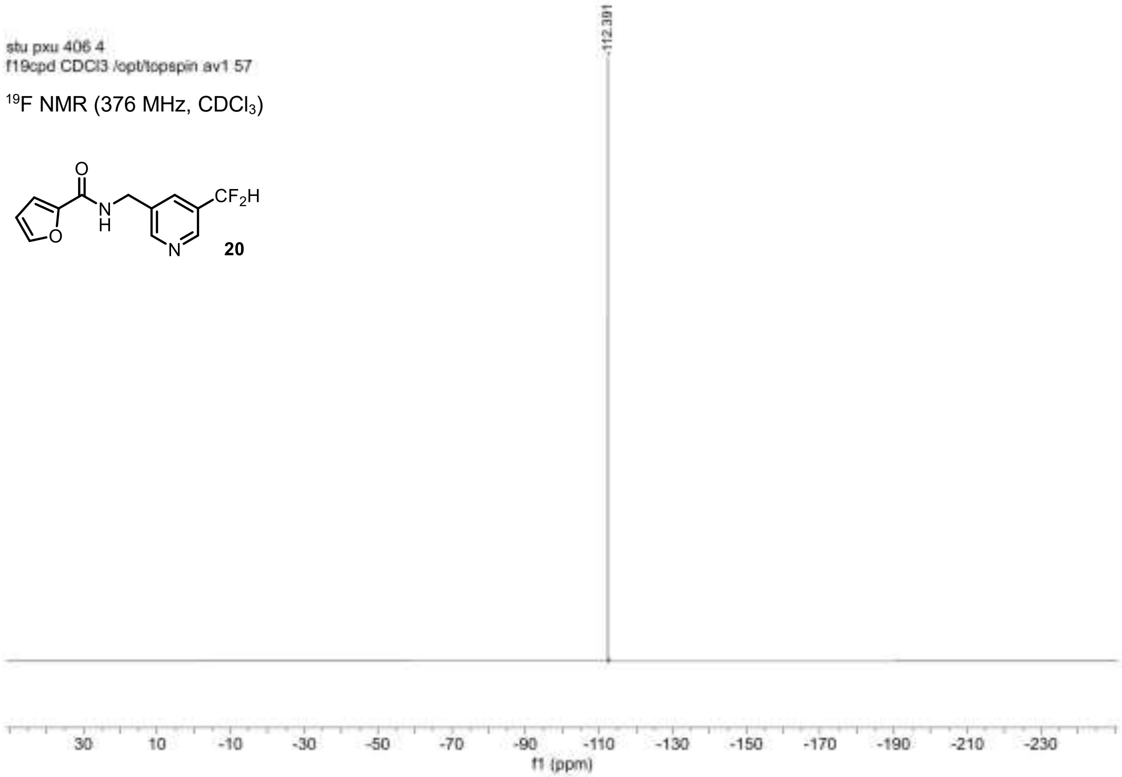

Supplementary Figure 98. ^{19}F NMR (376 MHz, $CDCl_3$) spectrum of compound **20**

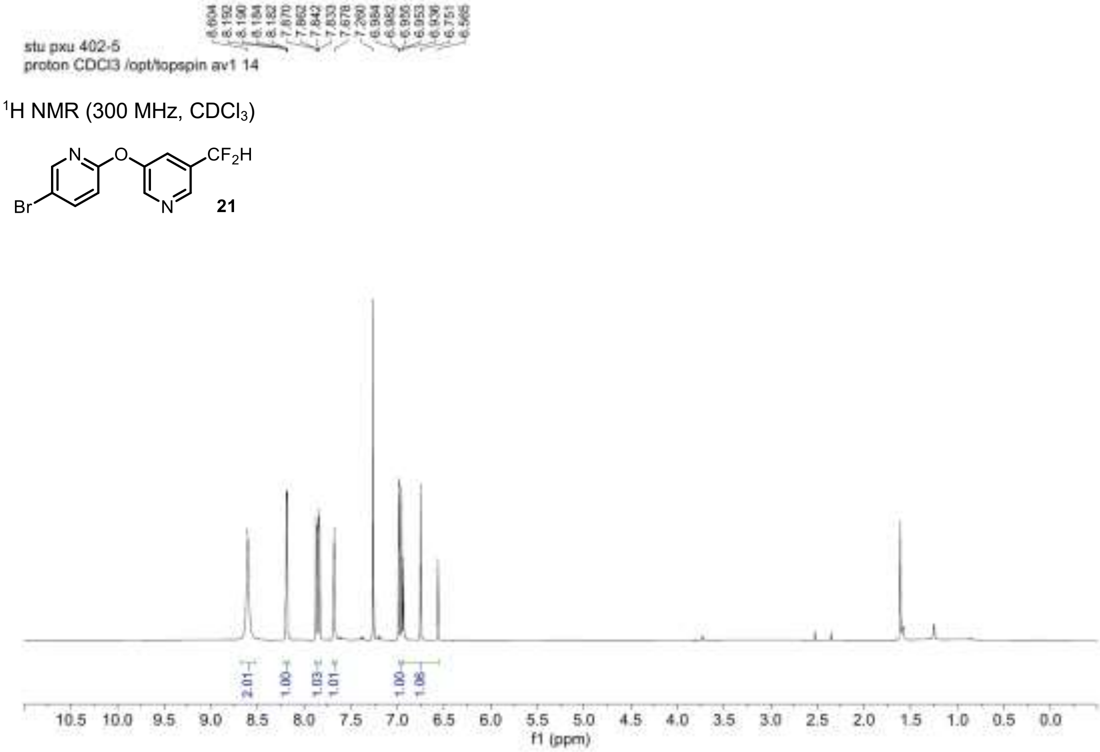

Supplementary Figure 99. ^{1}H NMR (300 MHz, $CDCl_3$) spectrum of compound **21**

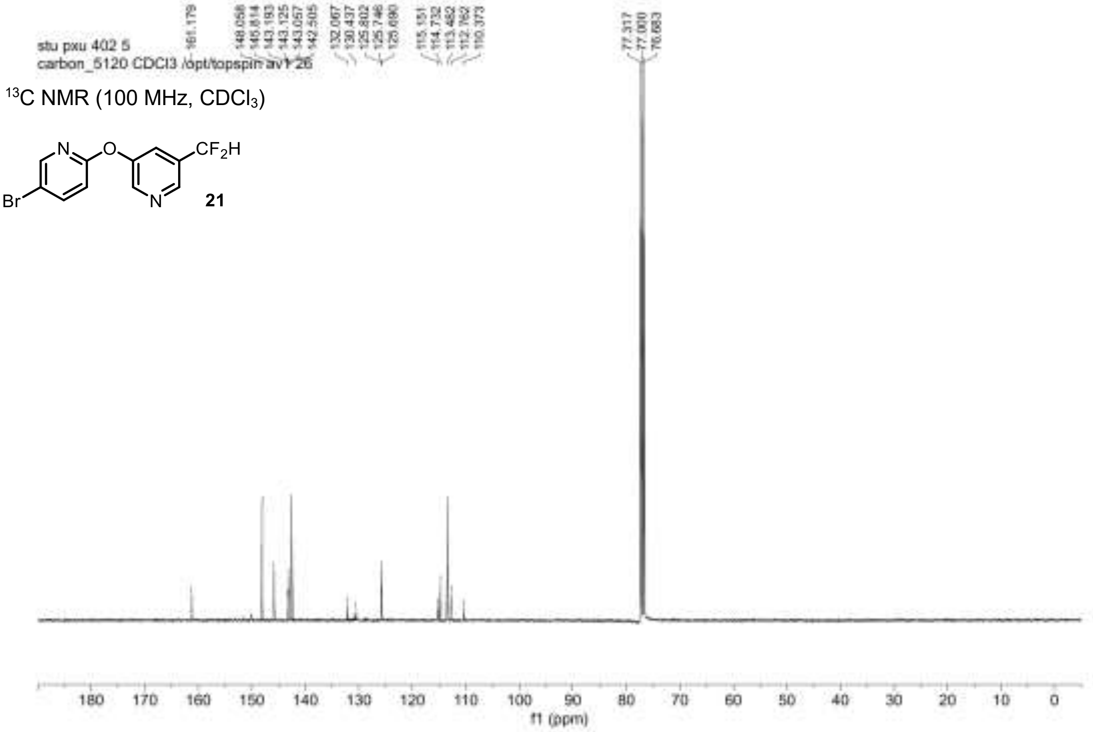

Supplementary Figure 100. ^{13}C NMR (100 MHz, $CDCl_3$) spectrum of compound **21**

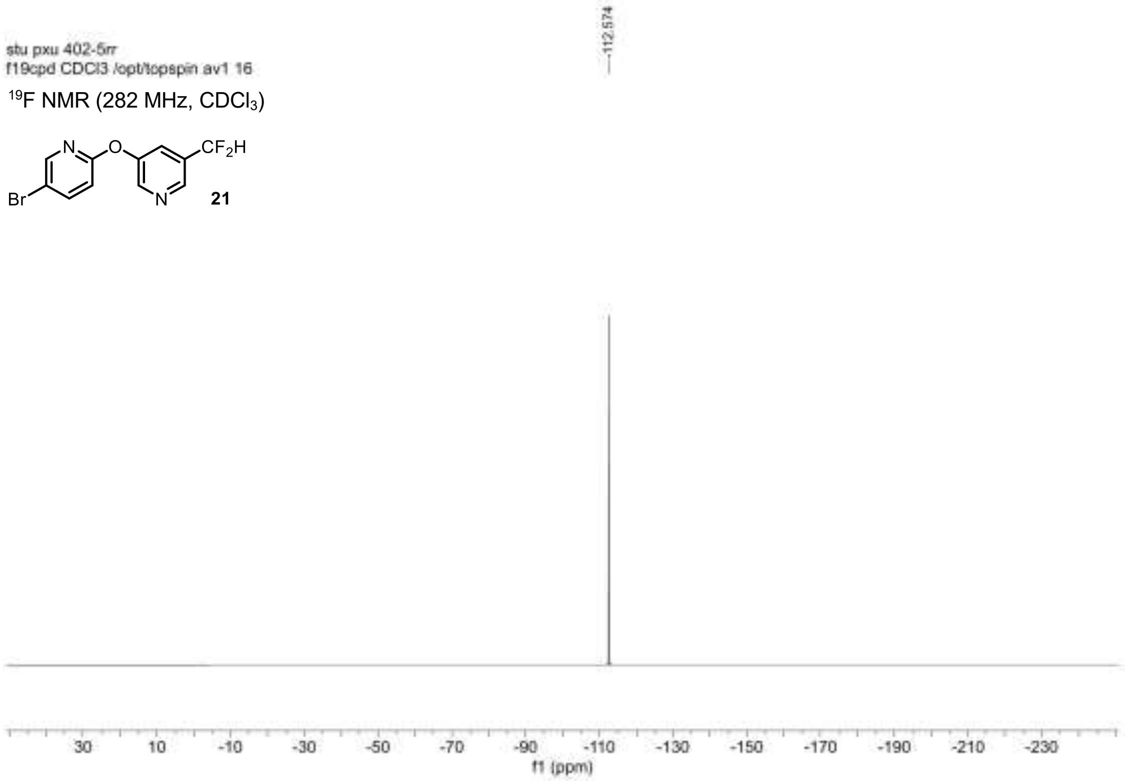

Supplementary Figure 101. ^{19}F NMR (282 MHz, $CDCl_3$) spectrum of compound **21**

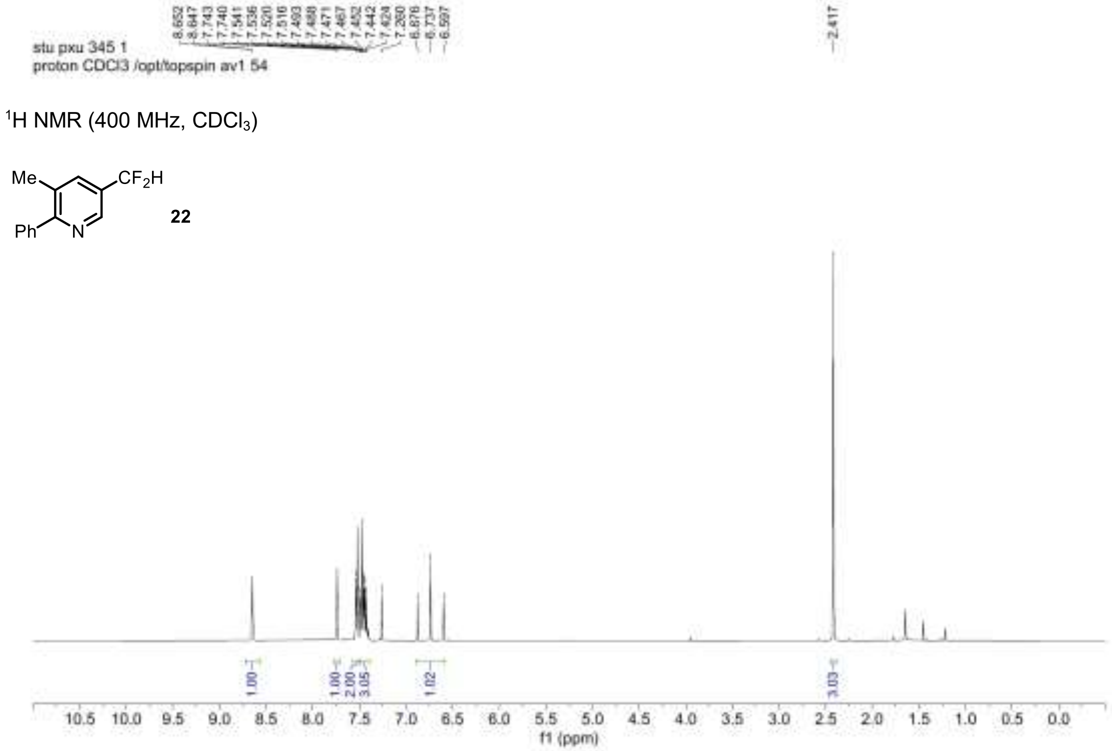

Supplementary Figure 102. ^{1}H NMR (400 MHz, $CDCl_3$) spectrum of compound **22**

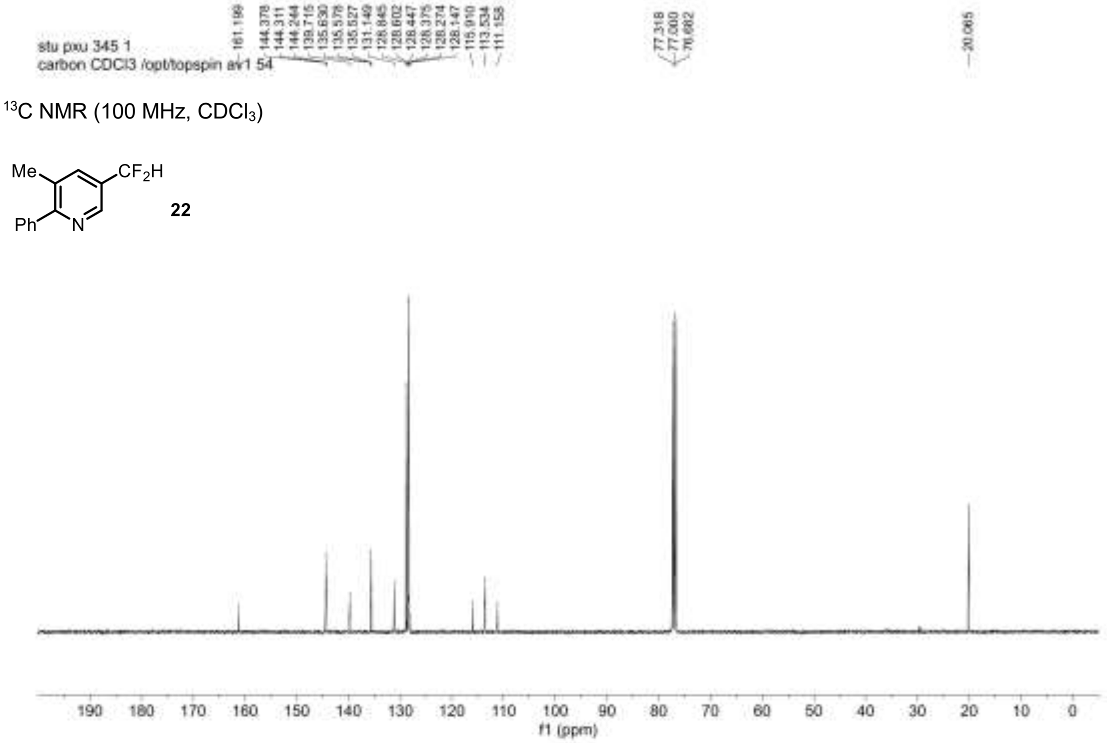

Supplementary Figure 103. ^{13}C NMR (100 MHz, $CDCl_3$) spectrum of compound **22**

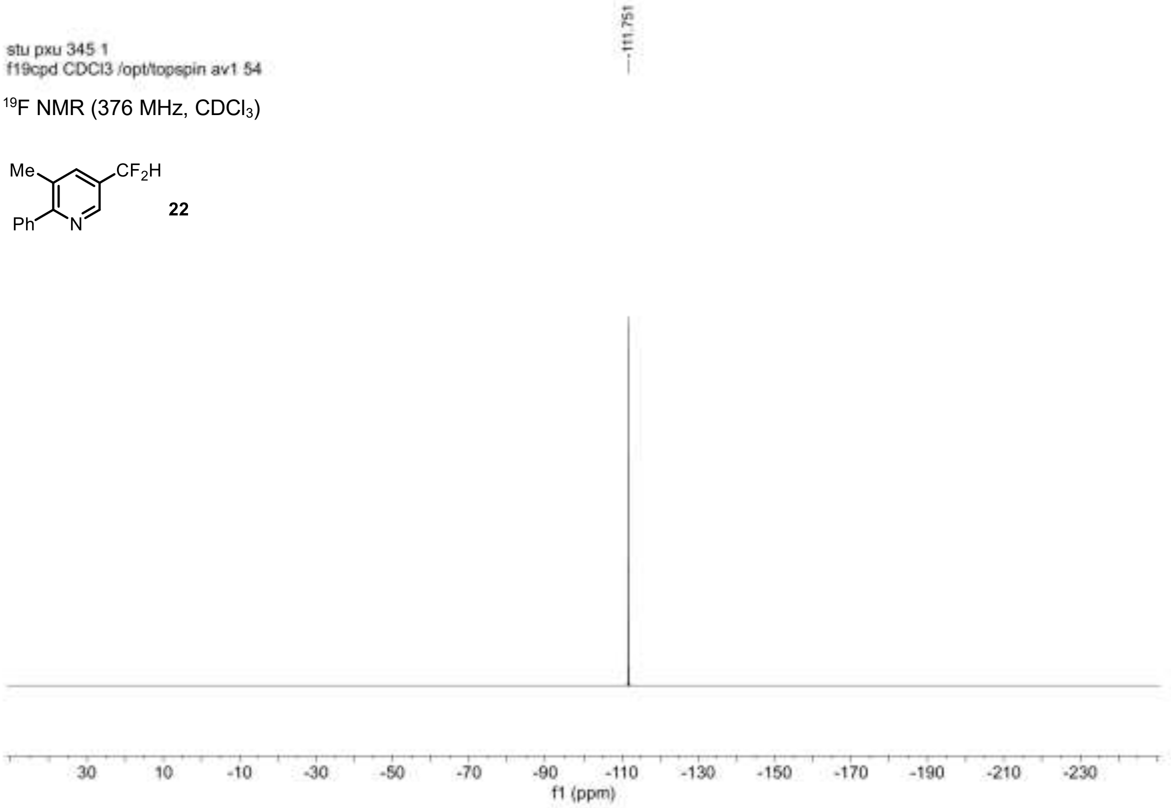

Supplementary Figure 104. ^{19}F NMR (376 MHz, $CDCl_3$) spectrum of compound **22**

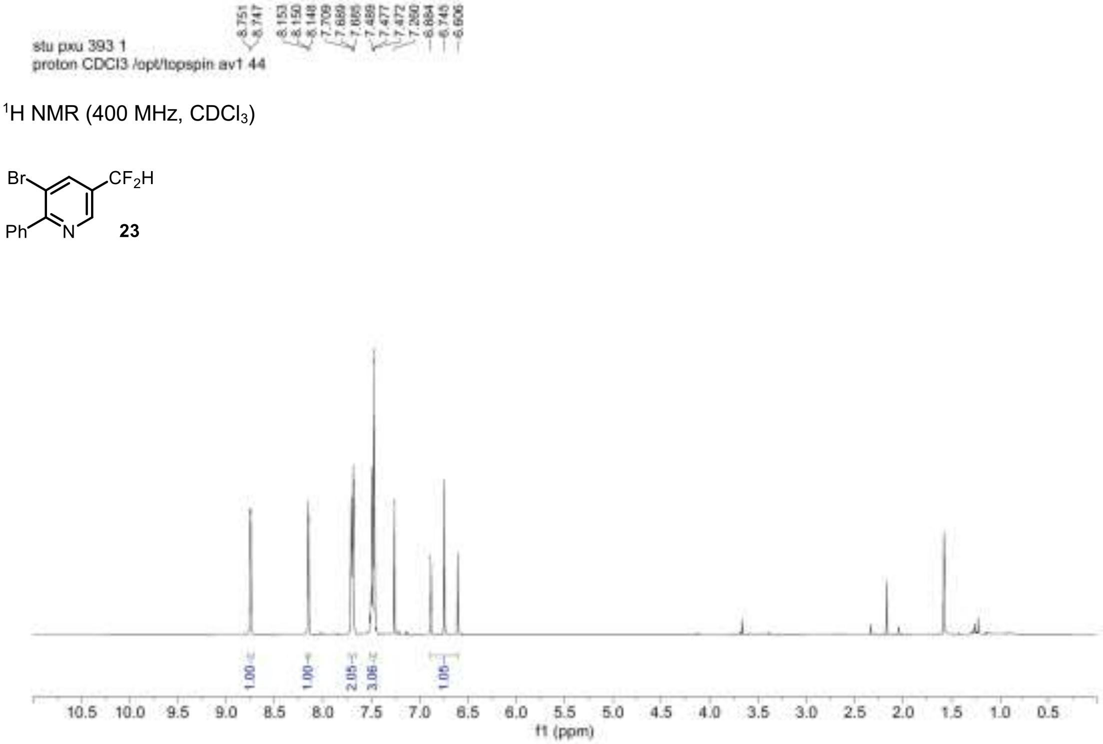

Supplementary Figure 105. ^{1}H NMR (400 MHz, $CDCl_3$) spectrum of compound **23**

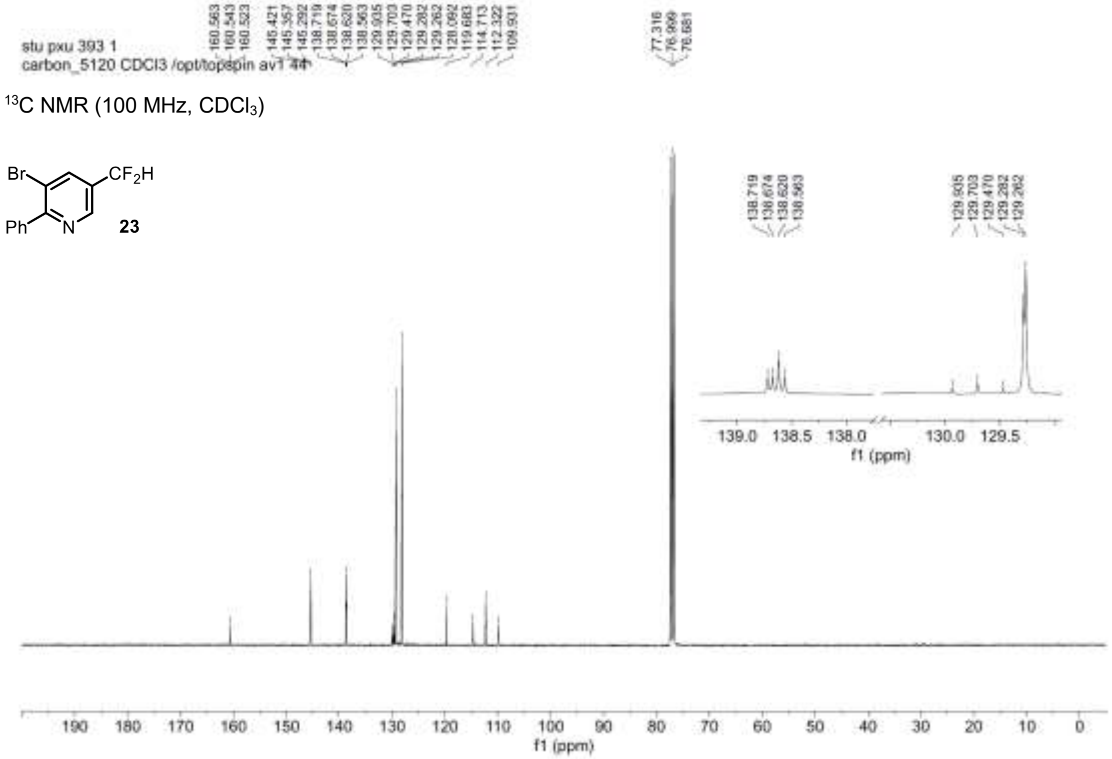

Supplementary Figure 106. ^{13}C NMR (100 MHz, $CDCl_3$) spectrum of compound **23**

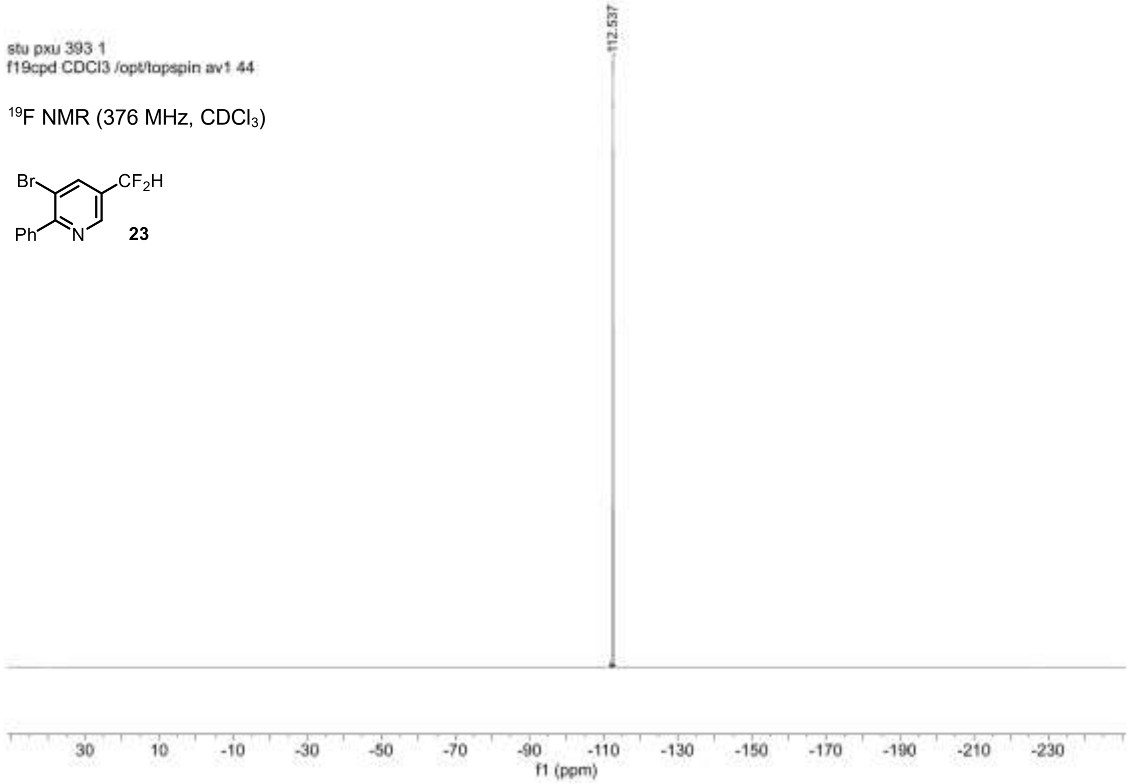

Supplementary Figure 107. ^{19}F NMR (376 MHz, $CDCl_3$) spectrum of compound **23**

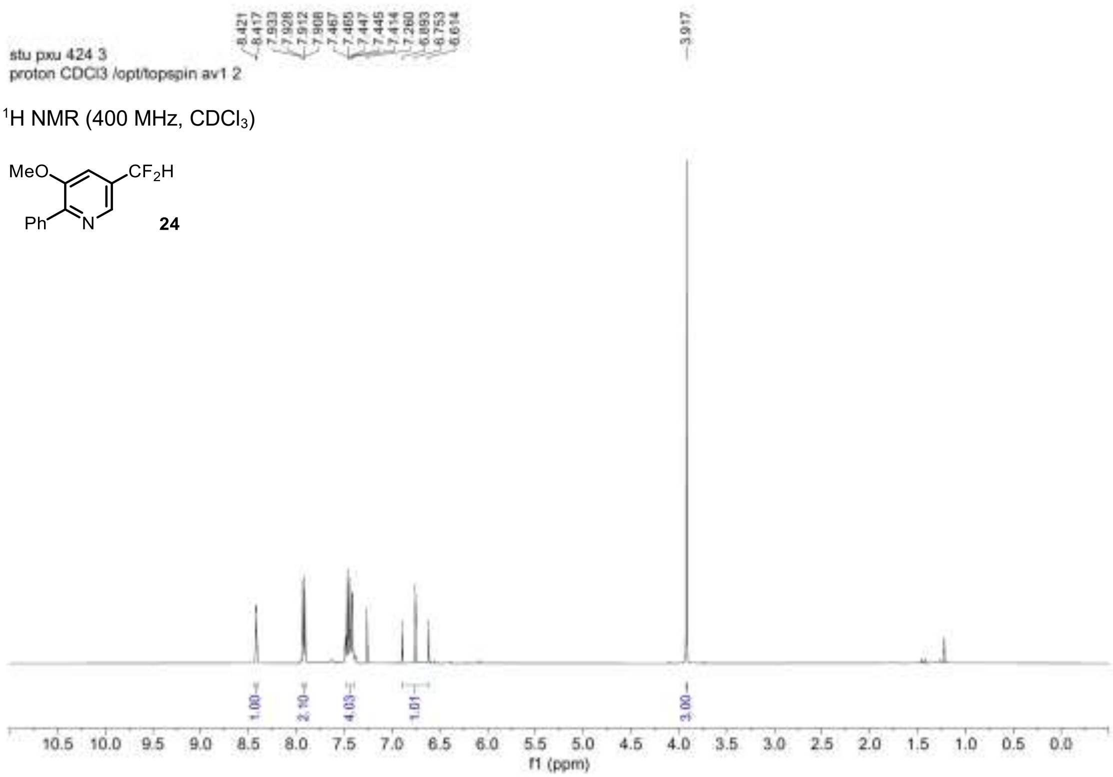

Supplementary Figure 108. ^{1}H NMR (400 MHz, $CDCl_3$) spectrum of compound **24**

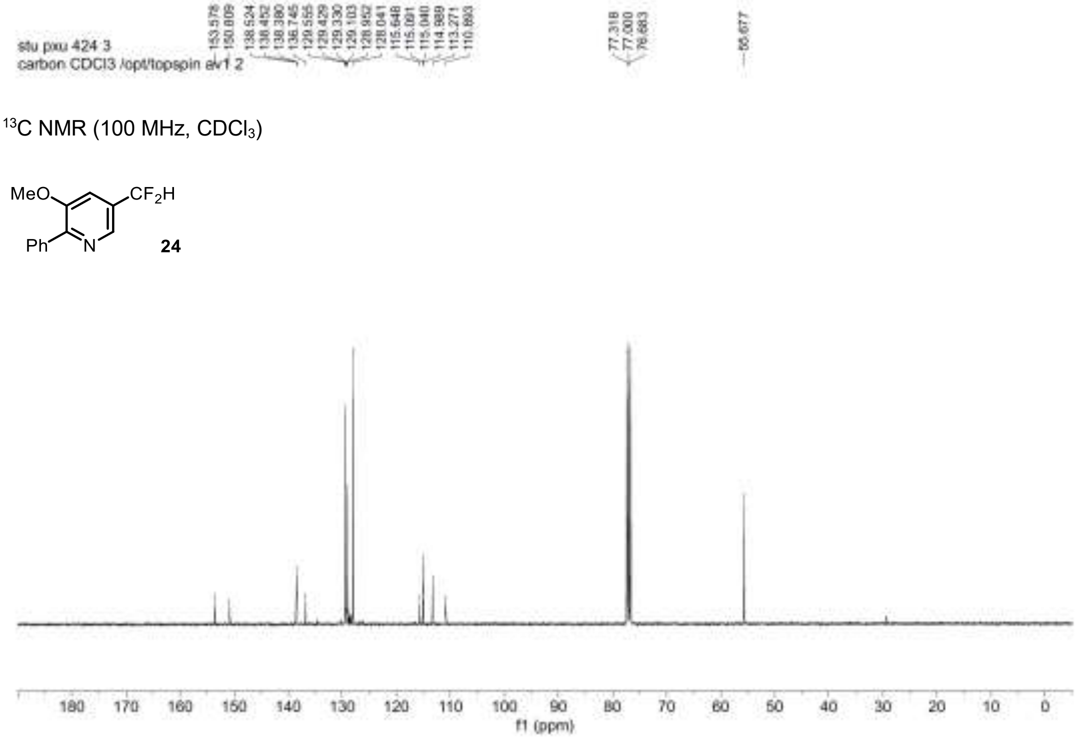

Supplementary Figure 109. ^{13}C NMR (100 MHz, $CDCl_3$) spectrum of compound **24**

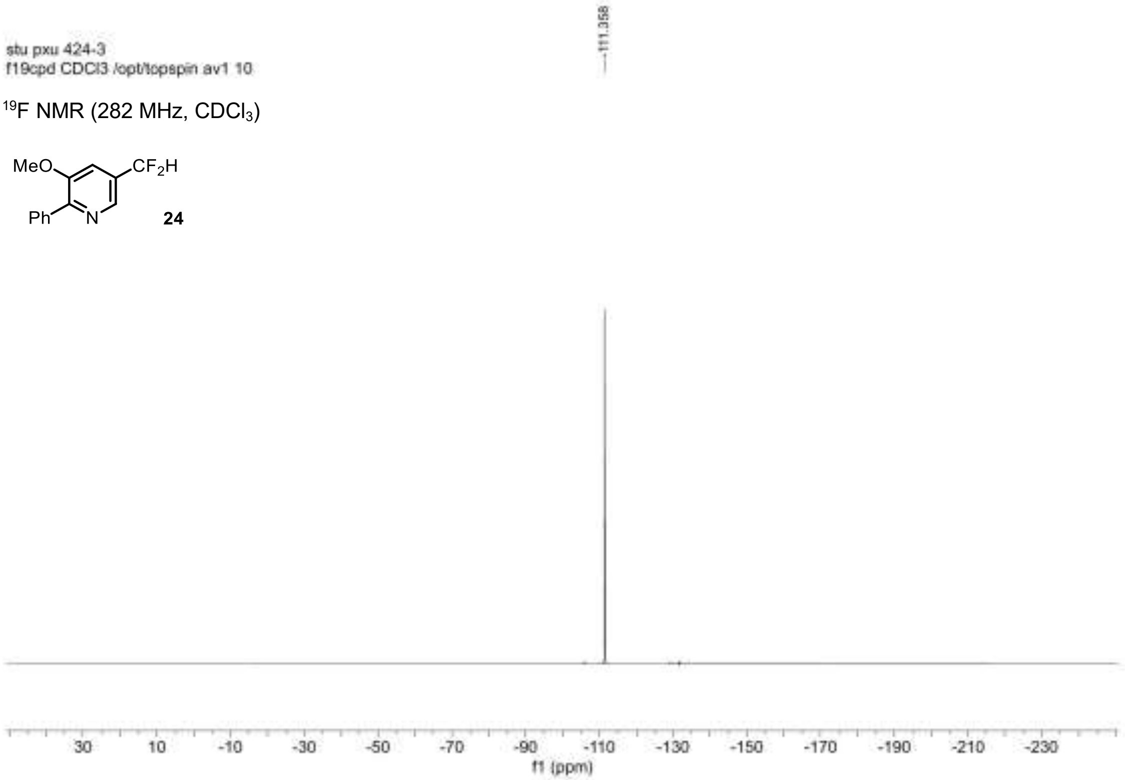

Supplementary Figure 110. ^{19}F NMR (282 MHz, $CDCl_3$) spectrum of compound **24**

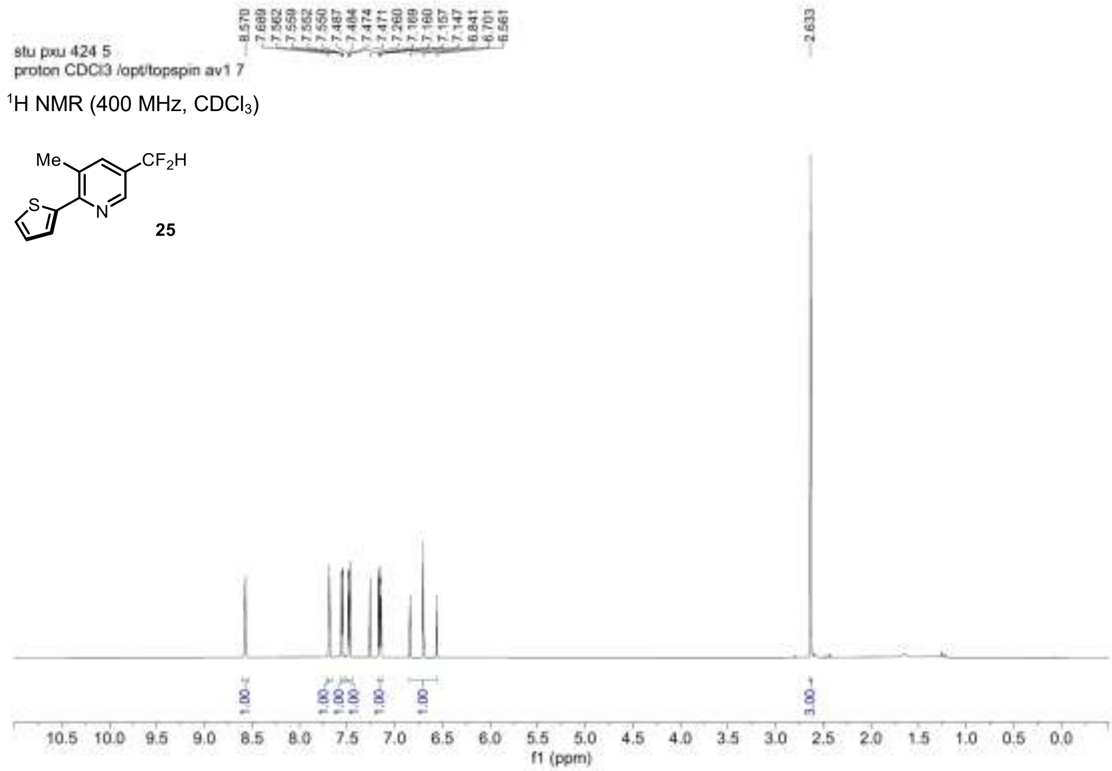

Supplementary Figure 111. ^{1}H NMR (400 MHz, $CDCl_3$) spectrum of compound **25**

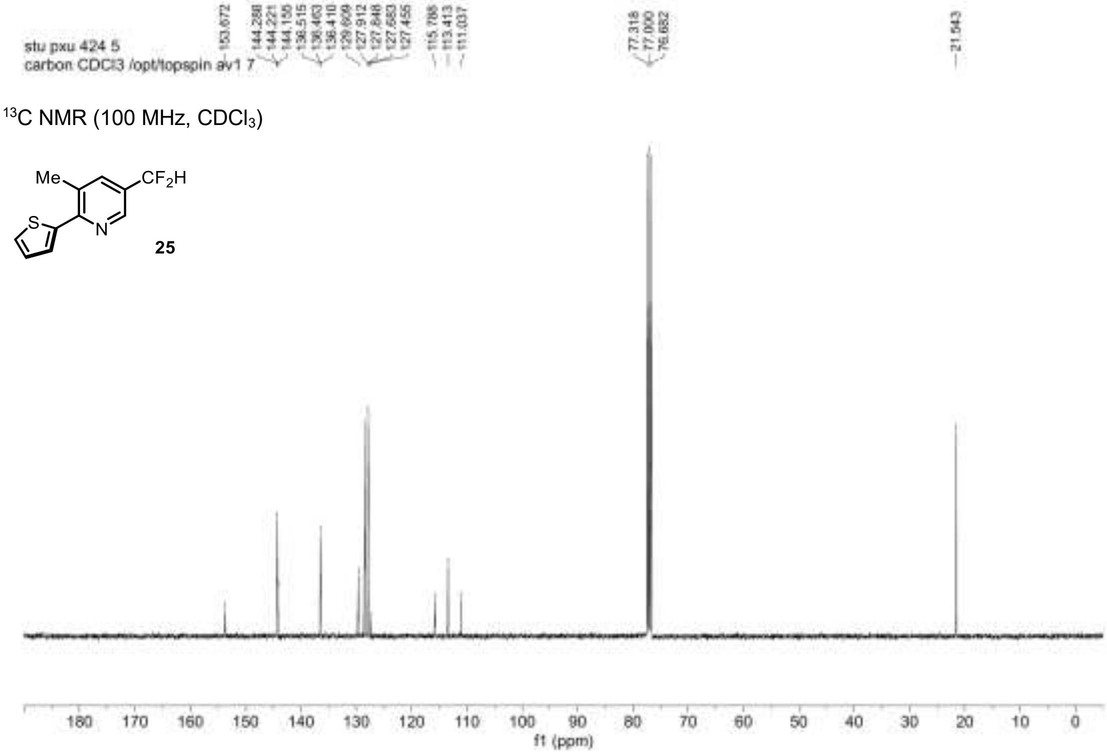

Supplementary Figure 112. ^{13}C NMR (100 MHz, $CDCl_3$) spectrum of compound **25**

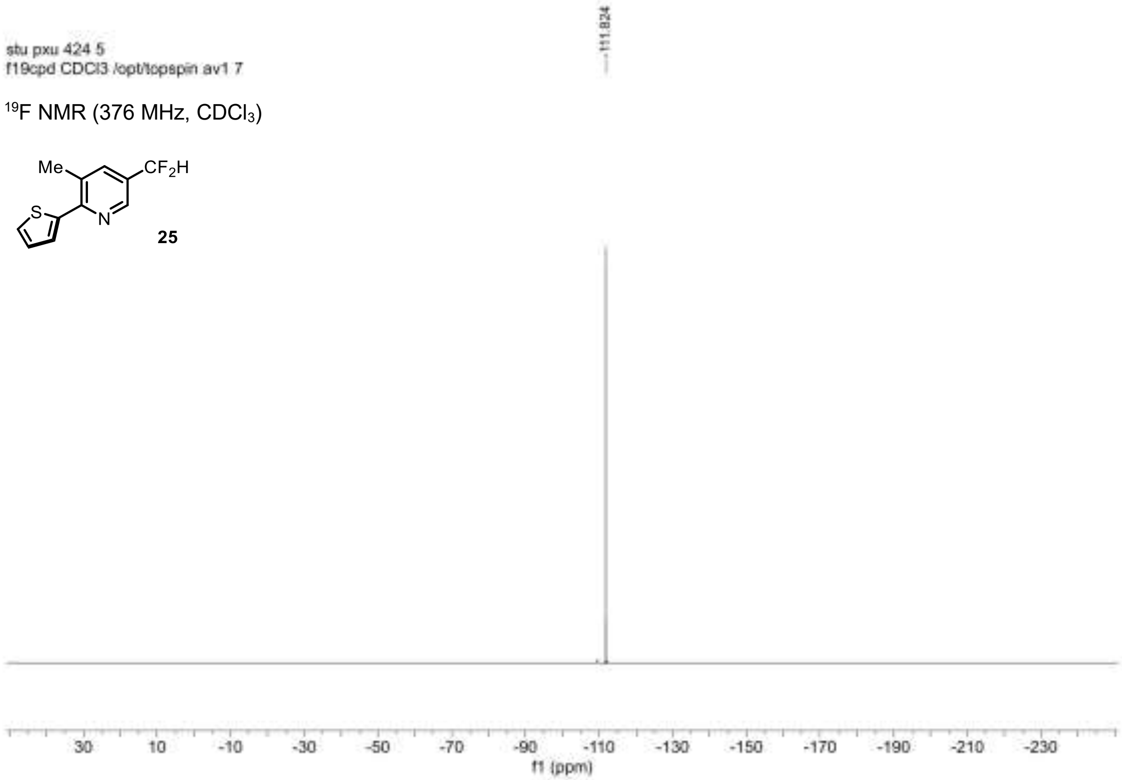

Supplementary Figure 113. ^{19}F NMR (376 MHz, $CDCl_3$) spectrum of compound **25**

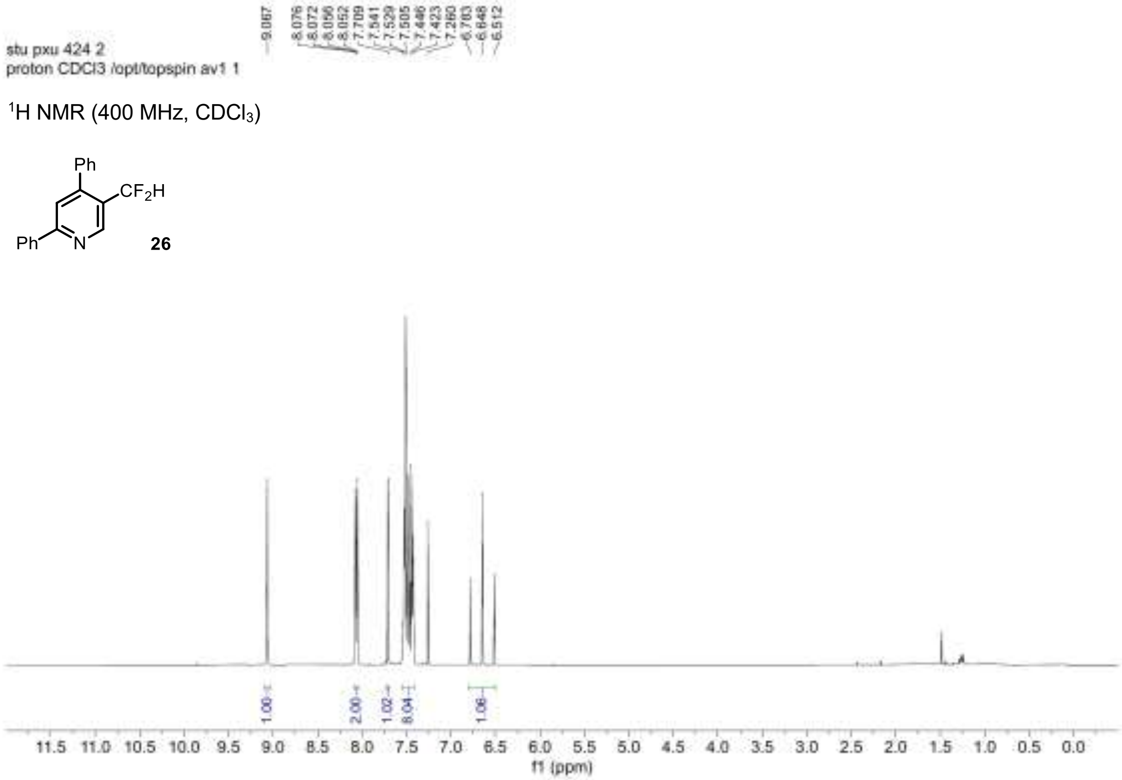

Supplementary Figure 114. ^{1}H NMR (400 MHz, $CDCl_3$) spectrum of compound **26**

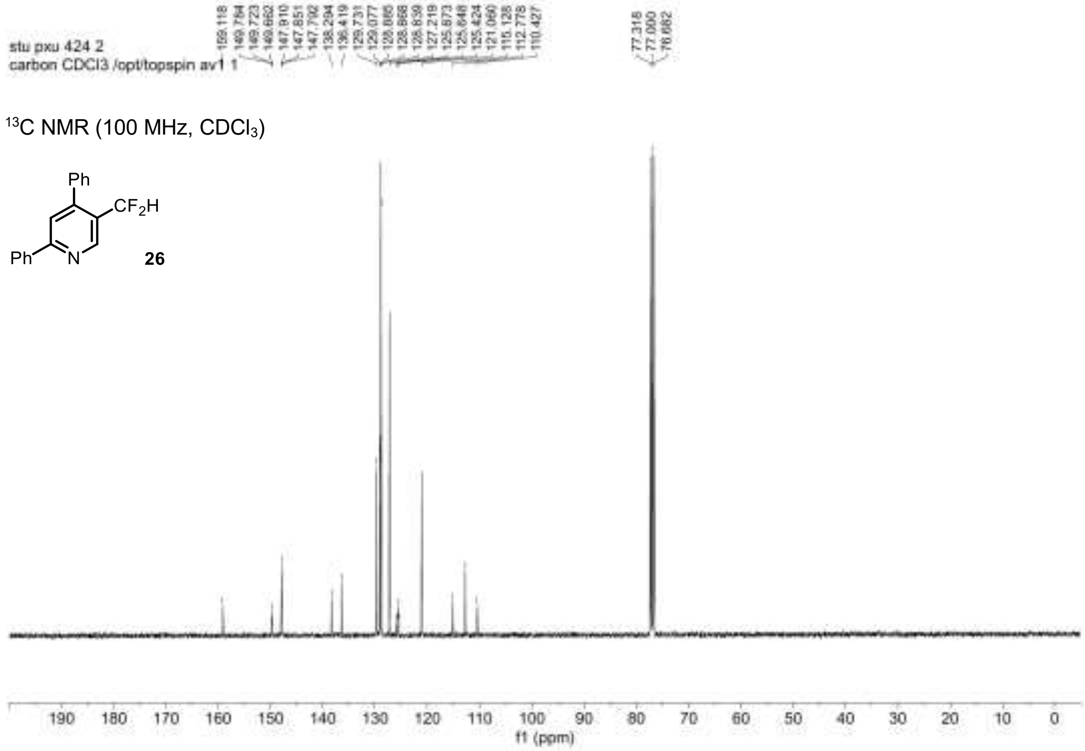

Supplementary Figure 115. ^{13}C NMR (100 MHz, $CDCl_3$) spectrum of compound **26**

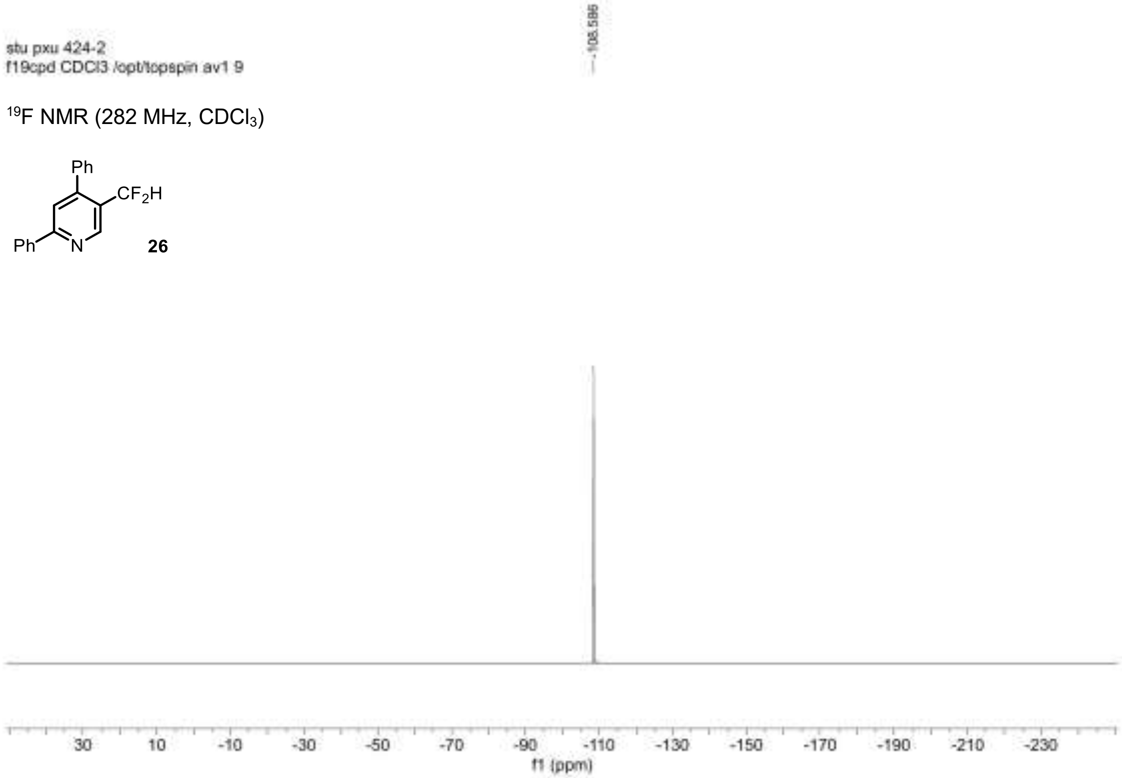

Supplementary Figure 116. ^{19}F NMR (282 MHz, $CDCl_3$) spectrum of compound **26**

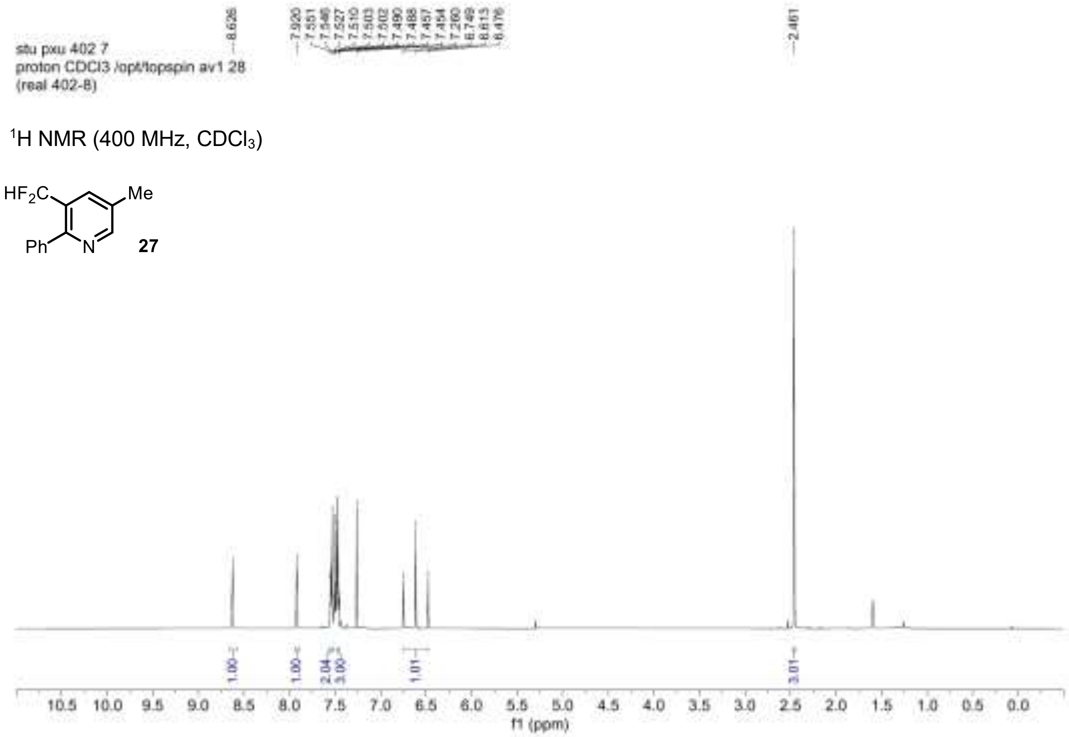

Supplementary Figure 117. ^{1}H NMR (400 MHz, $CDCl_3$) spectrum of compound **27**

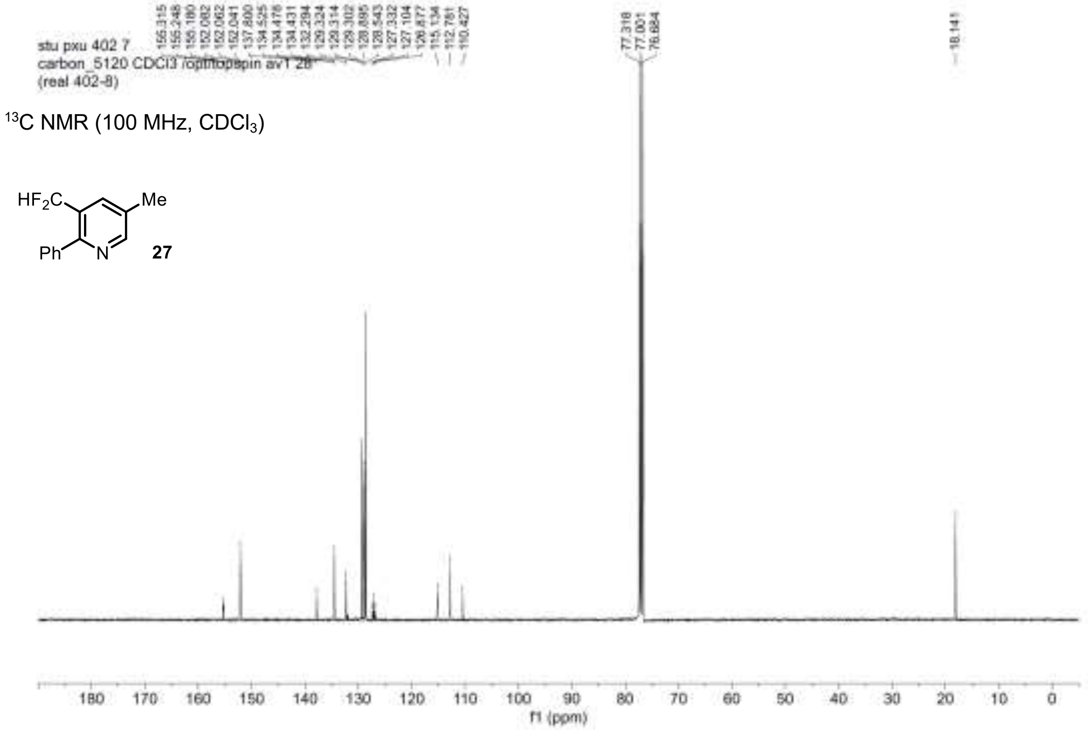

Supplementary Figure 118. ^{13}C NMR (100 MHz, $CDCl_3$) spectrum of compound **27**

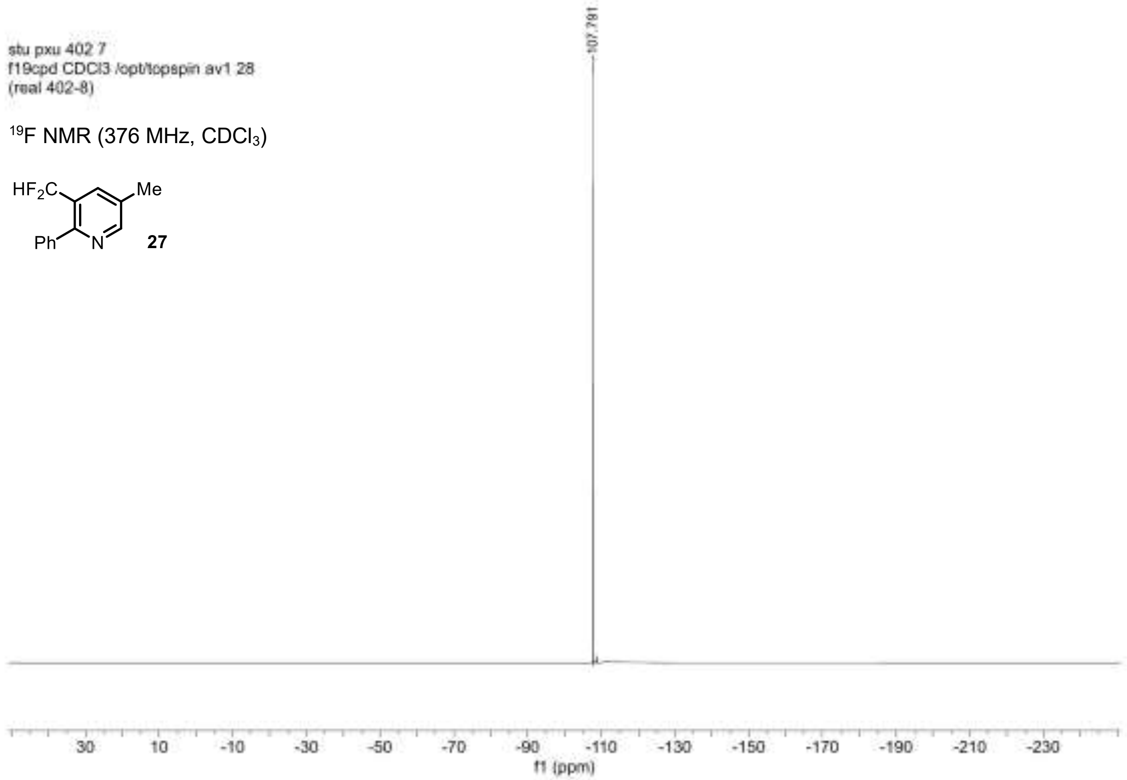

Supplementary Figure 119. ^{19}F NMR (376 MHz, $CDCl_3$) spectrum of compound **27**

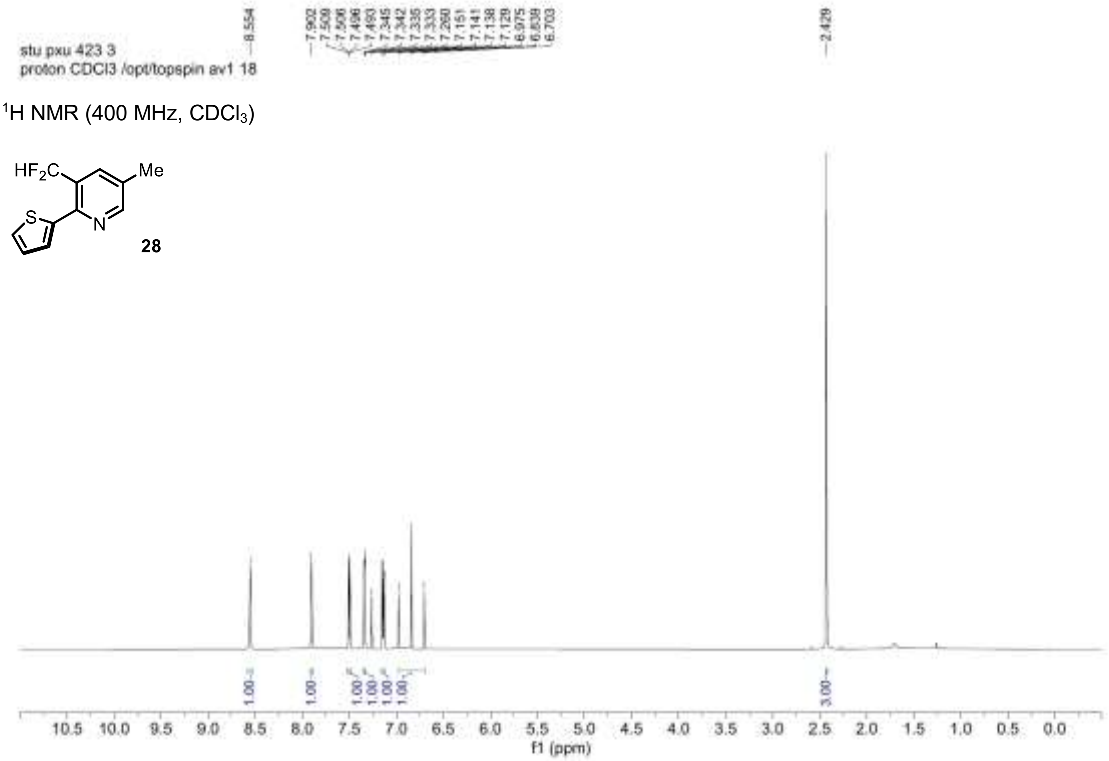

Supplementary Figure 120. ^{1}H NMR (400 MHz, CDCl$_3$) spectrum of compound **28**

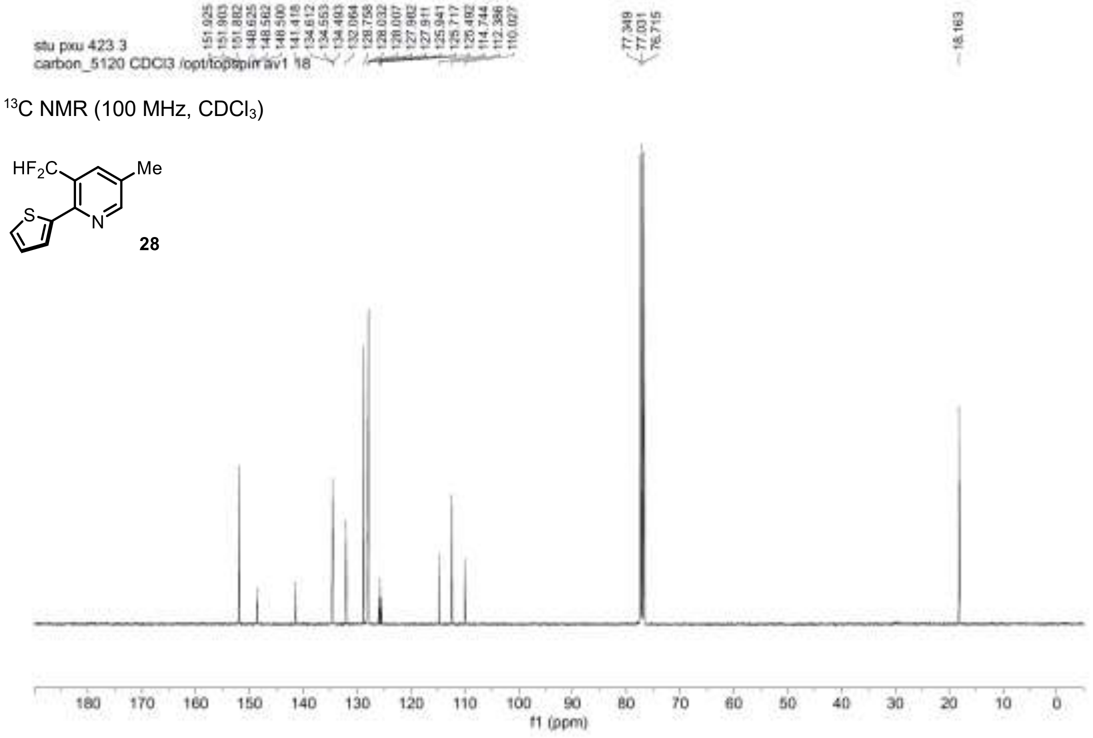

Supplementary Figure 121. ^{13}C NMR (100 MHz, CDCl$_3$) spectrum of compound **28**

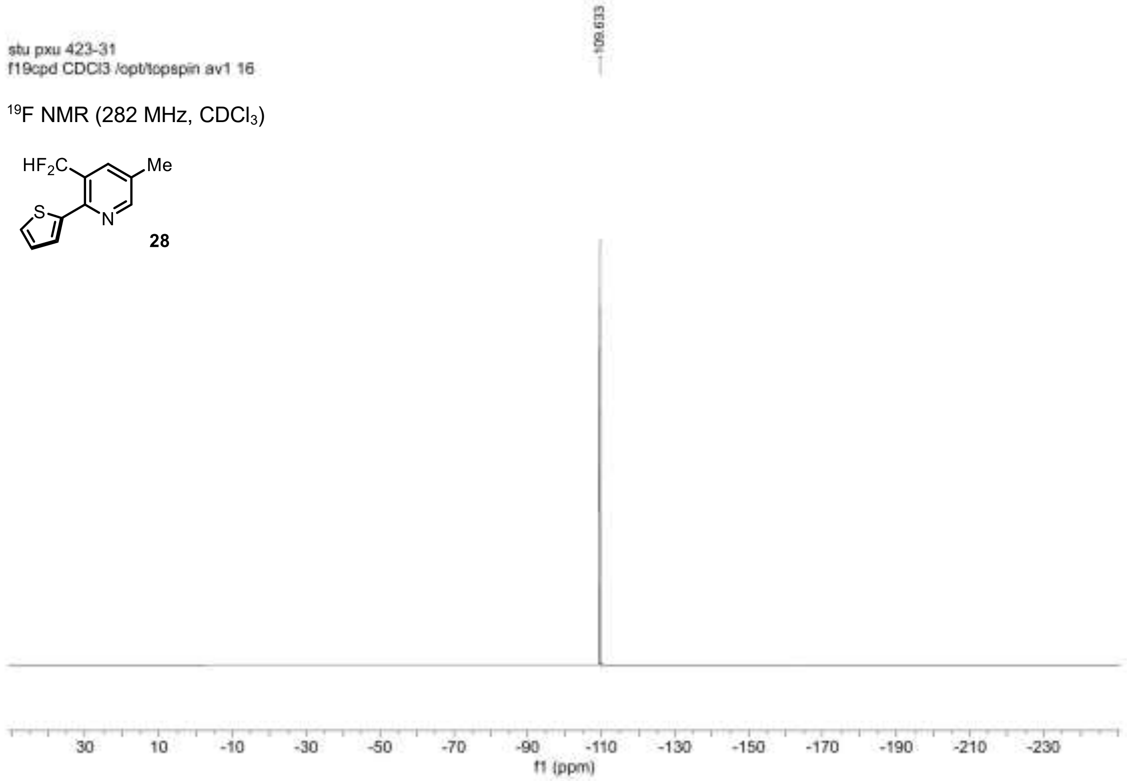

Supplementary Figure 122. ^{19}F NMR (282 MHz, $CDCl_3$) spectrum of compound **28**

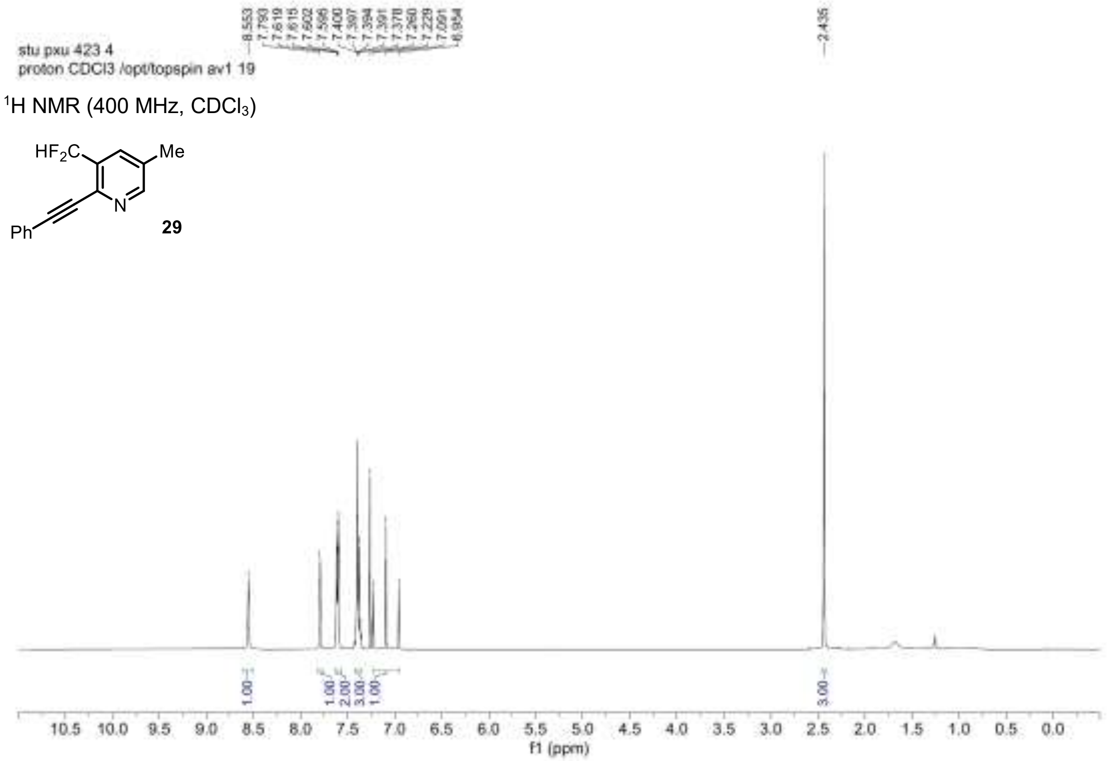

Supplementary Figure 123. ^{1}H NMR (400 MHz, $CDCl_3$) spectrum of compound **29**

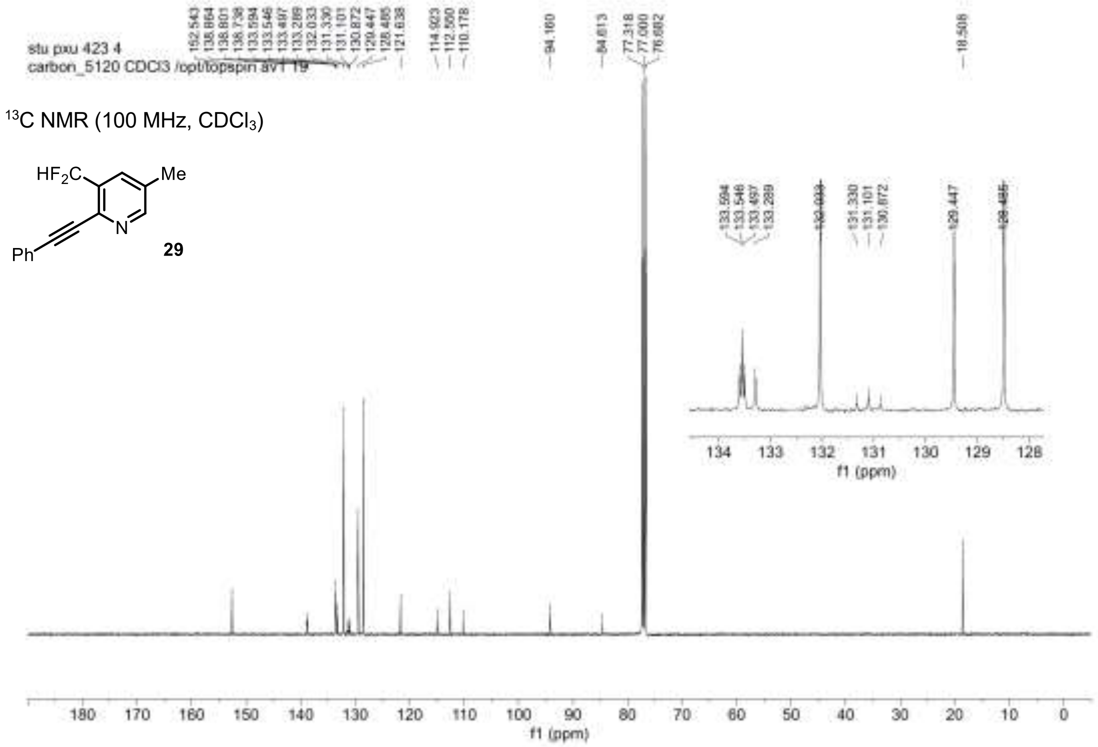

Supplementary Figure 124. ^{13}C NMR (100 MHz, $CDCl_3$) spectrum of compound **29**

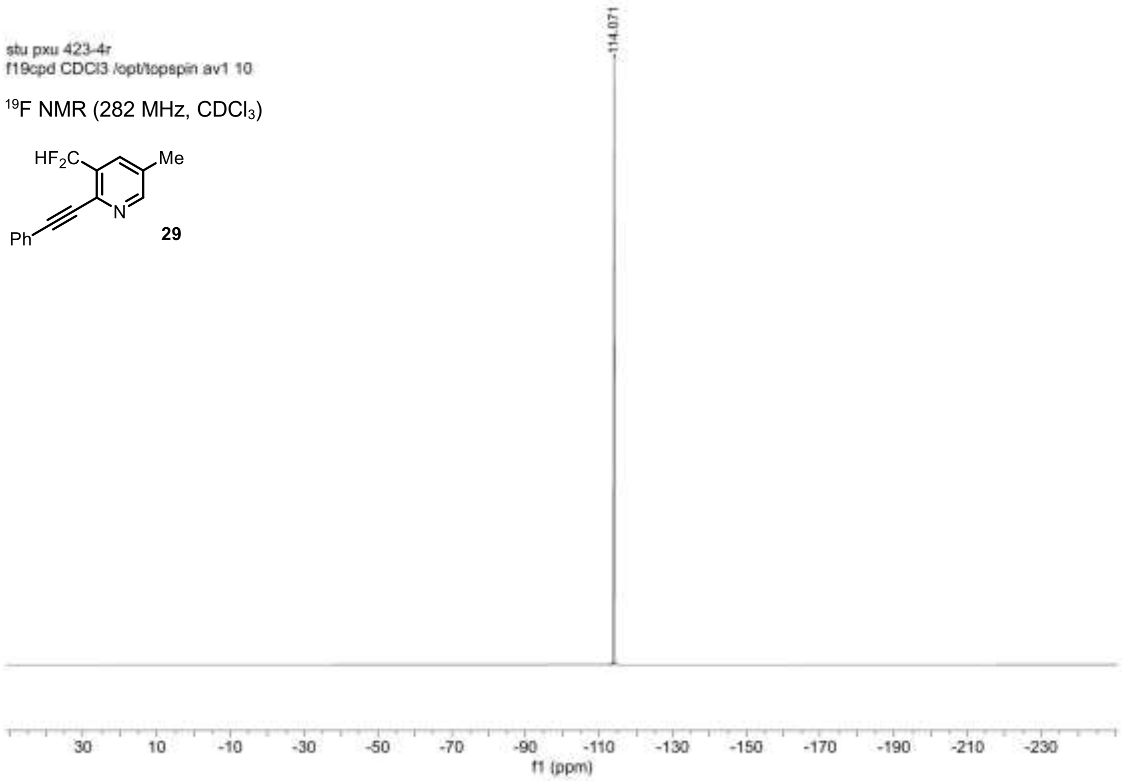

Supplementary Figure 125. ^{19}F NMR (282 MHz, $CDCl_3$) spectrum of compound **29**

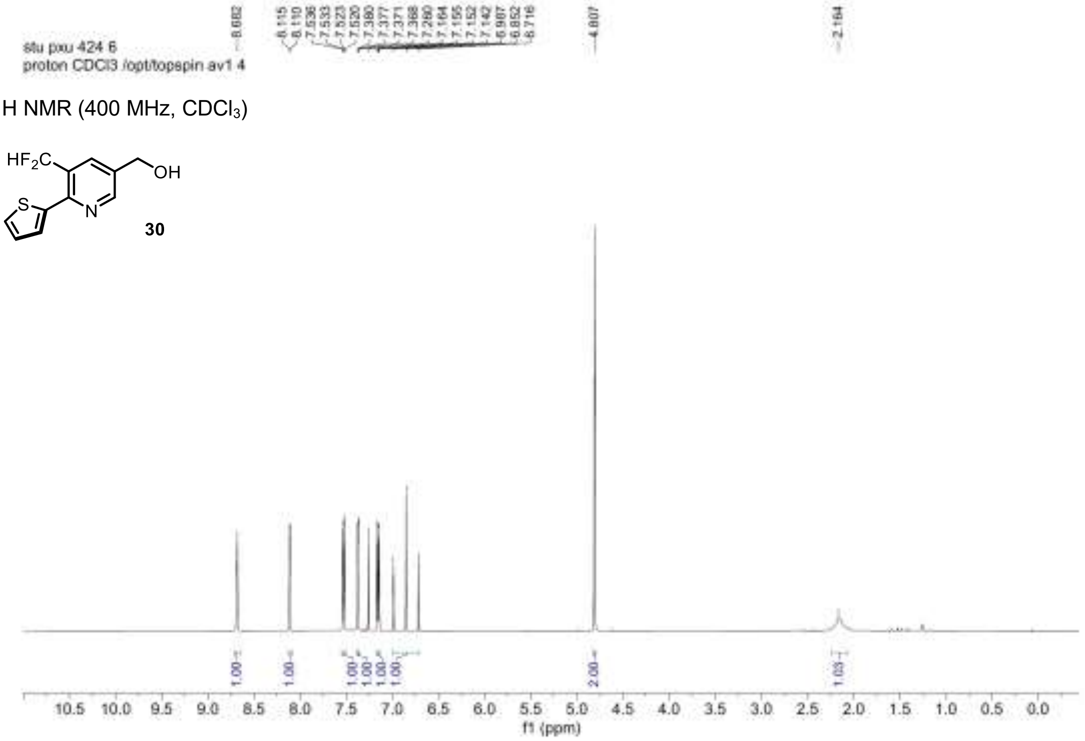

Supplementary Figure 126. ^{1}H NMR (400 MHz, $CDCl_3$) spectrum of compound **30**

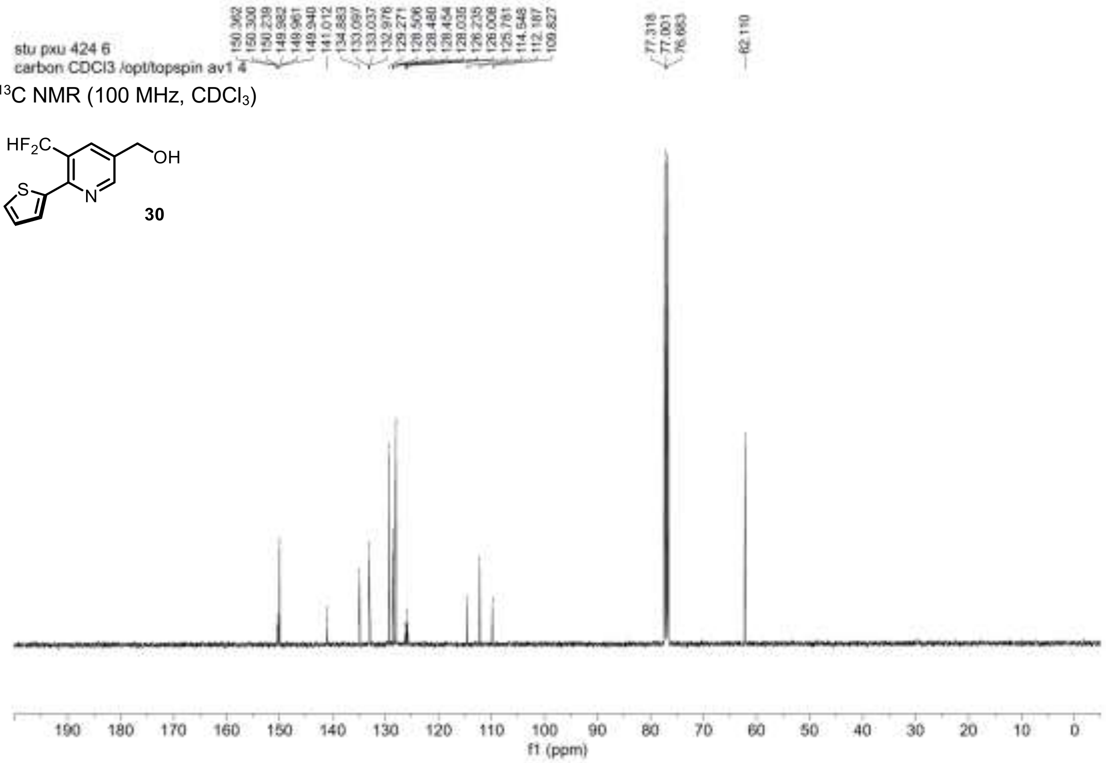

Supplementary Figure 127. ^{13}C NMR (100 MHz, $CDCl_3$) spectrum of compound **30**

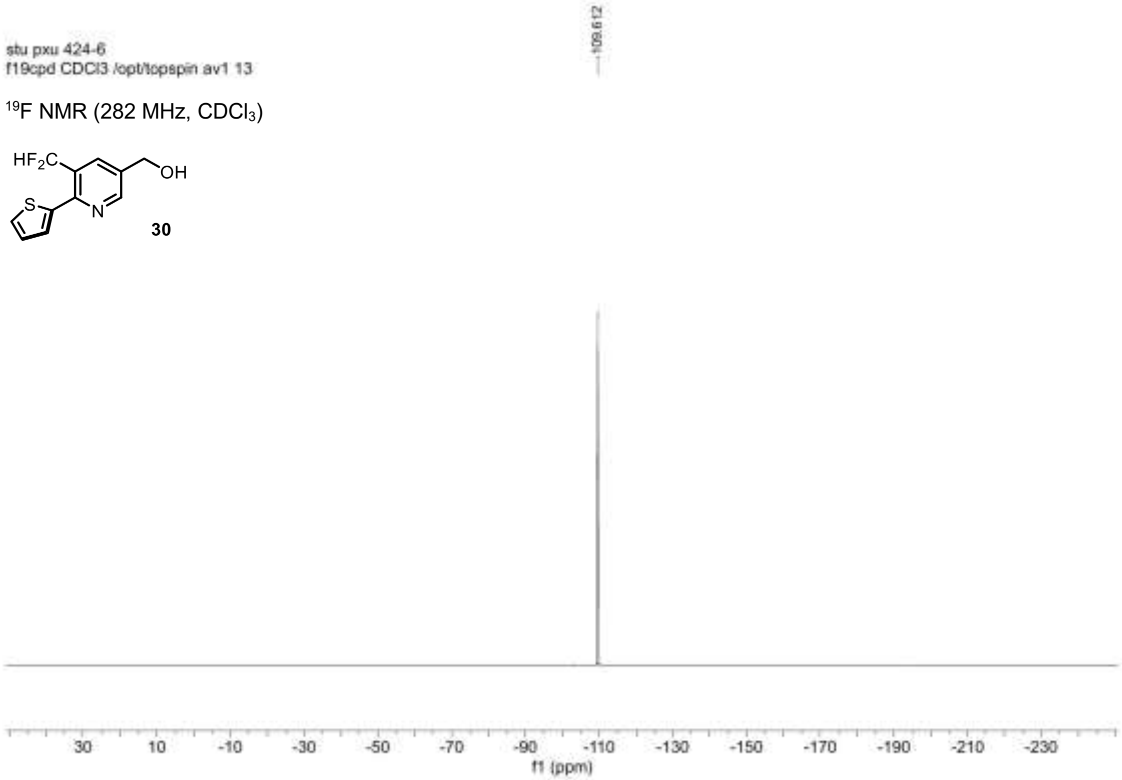

Supplementary Figure 128. ^{19}F NMR (282 MHz, $CDCl_3$) spectrum of compound **30**

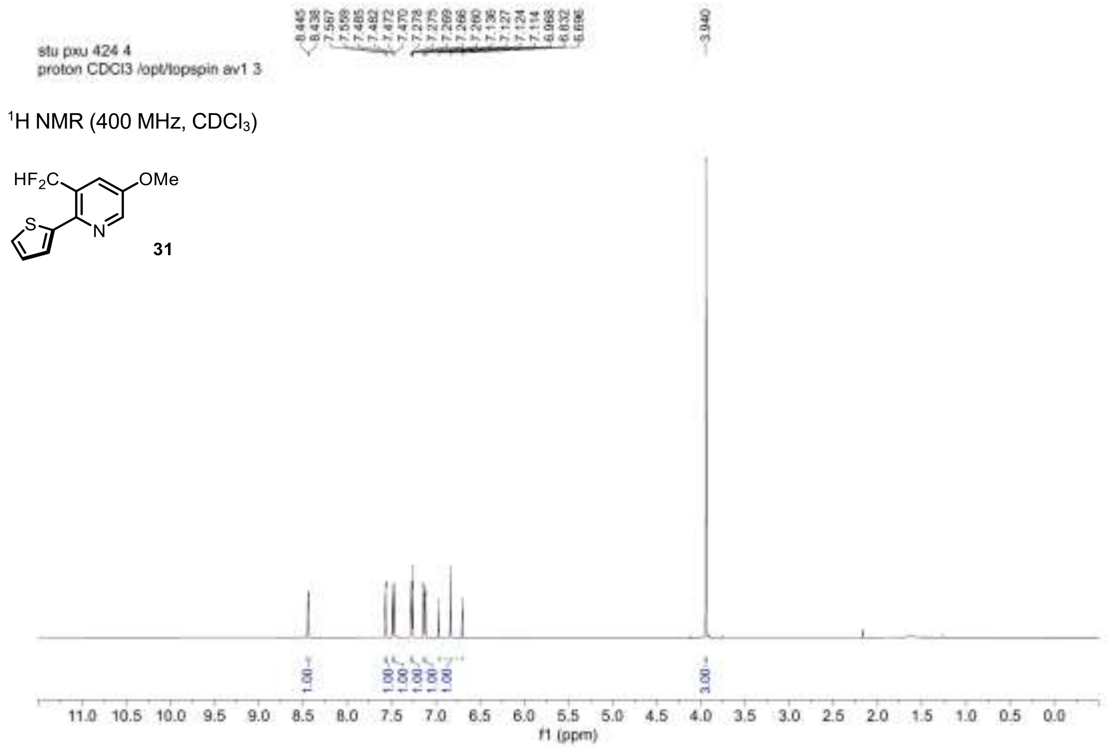

Supplementary Figure 129 ^{1}H NMR (400 MHz, $CDCl_3$) spectrum of compound **31**

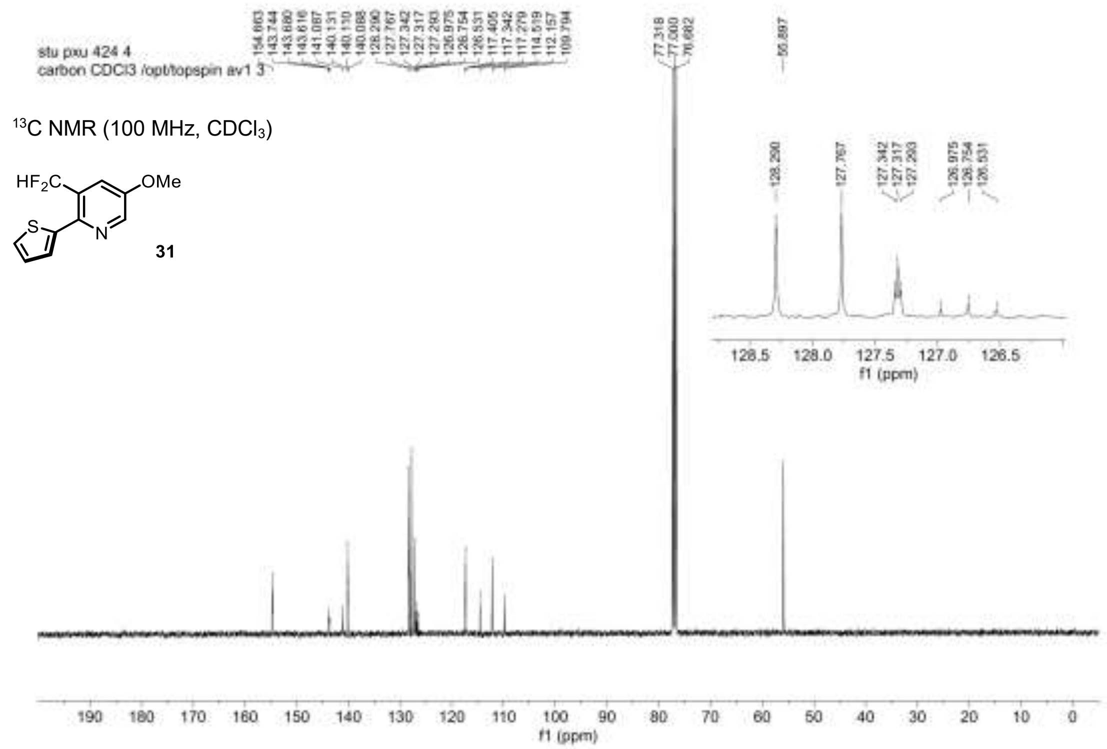

Supplementary Figure 130. ^{13}C NMR (100 MHz, $CDCl_3$) spectrum of compound **31**

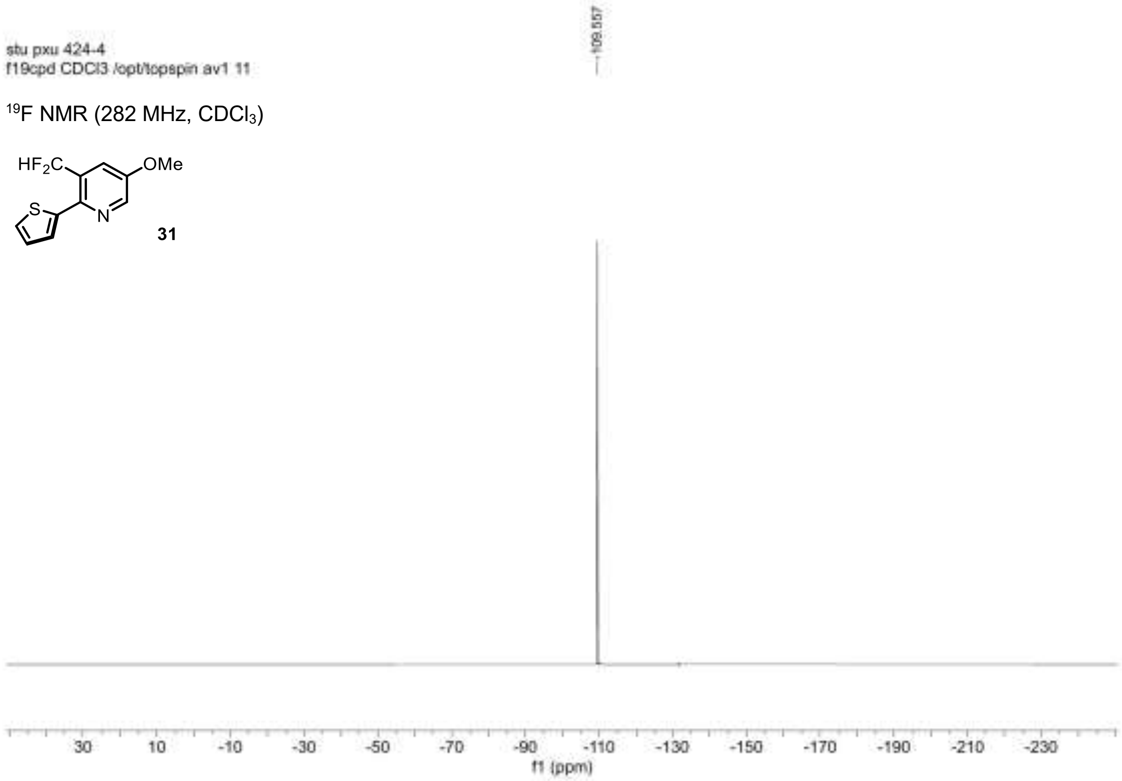

Supplementary Figure 131. ^{19}F NMR (282 MHz, $CDCl_3$) spectrum of compound **31**

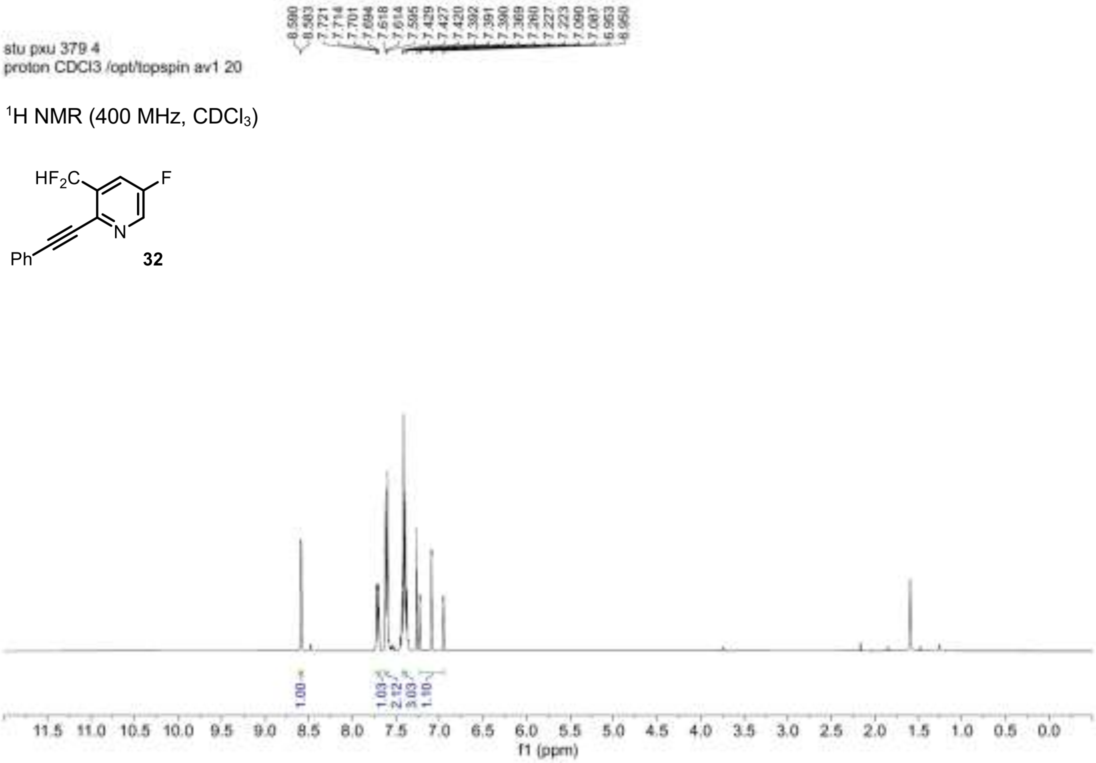

Supplementary Figure 132 ^{1}H NMR (400 MHz, $CDCl_3$) spectrum of compound **32**

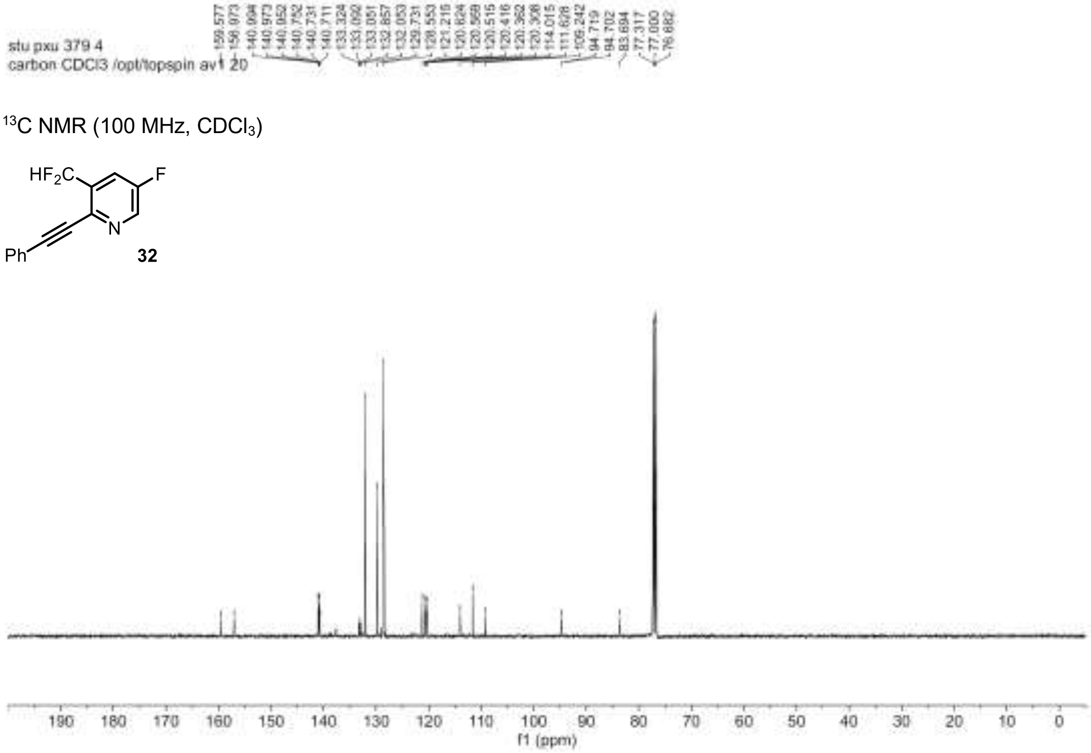

Supplementary Figure 133. ^{13}C NMR (100 MHz, $CDCl_3$) spectrum of compound **32**

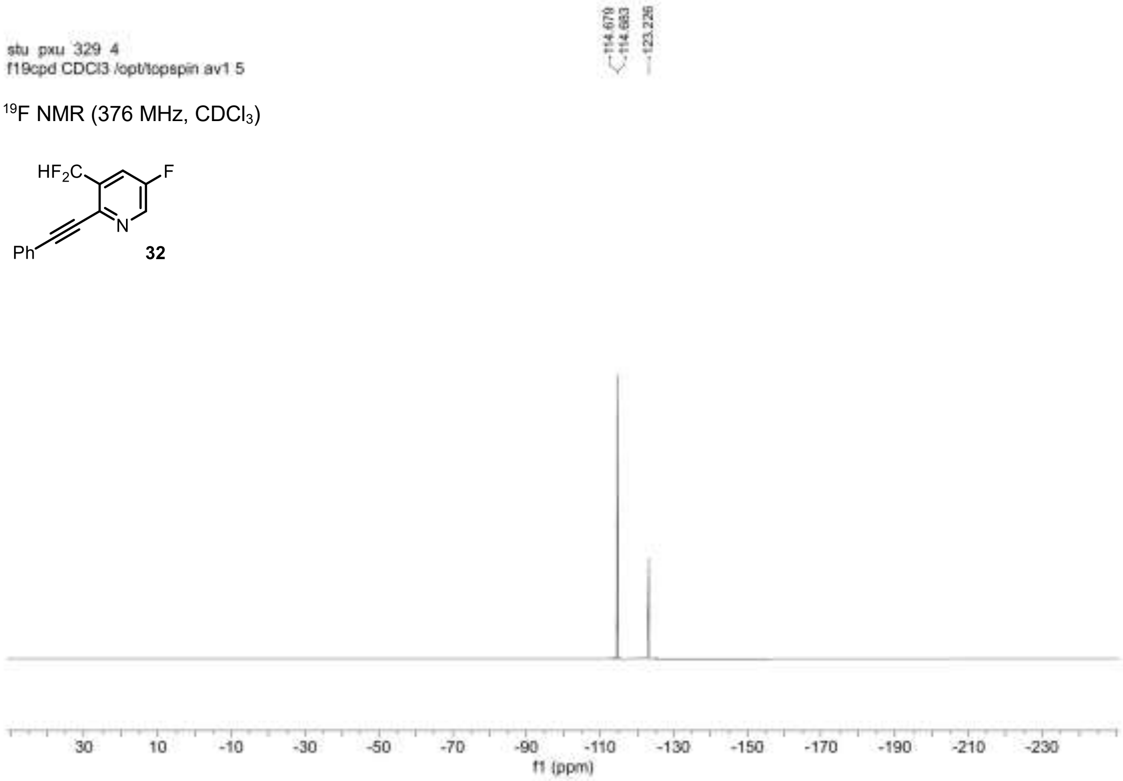

Supplementary Figure 134. ^{19}F NMR (376 MHz, $CDCl_3$) spectrum of compound **32**

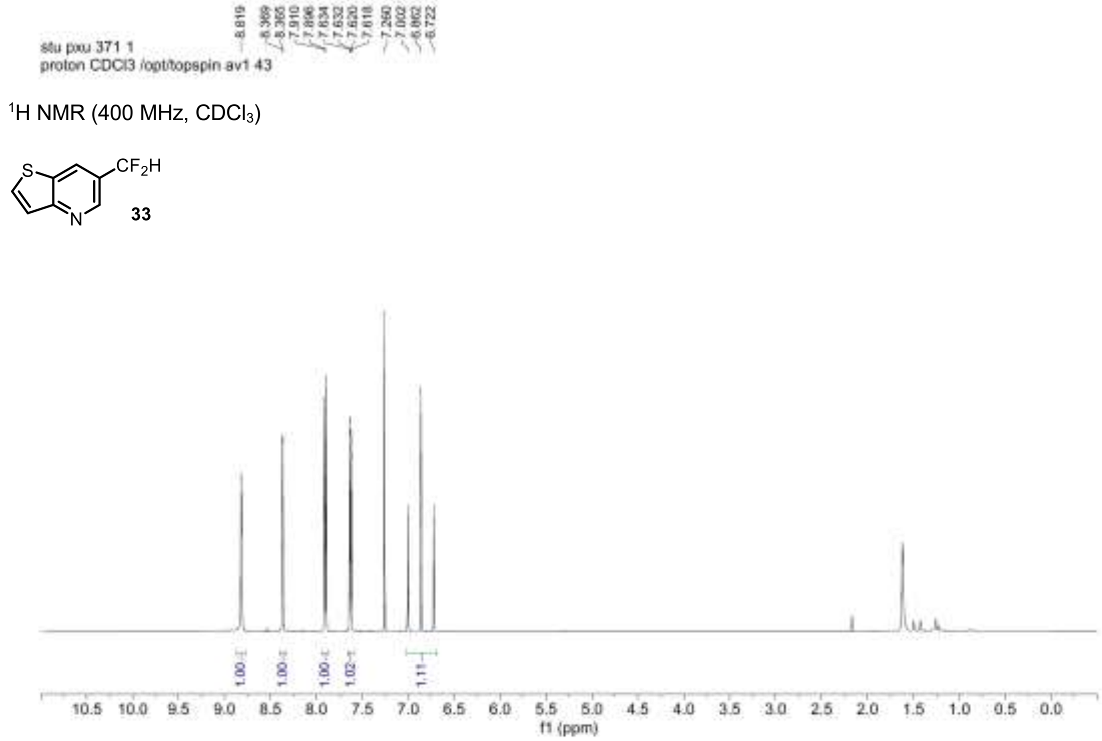

Supplementary Figure 135. ^{1}H NMR (400 MHz, $CDCl_3$) spectrum of compound **33**

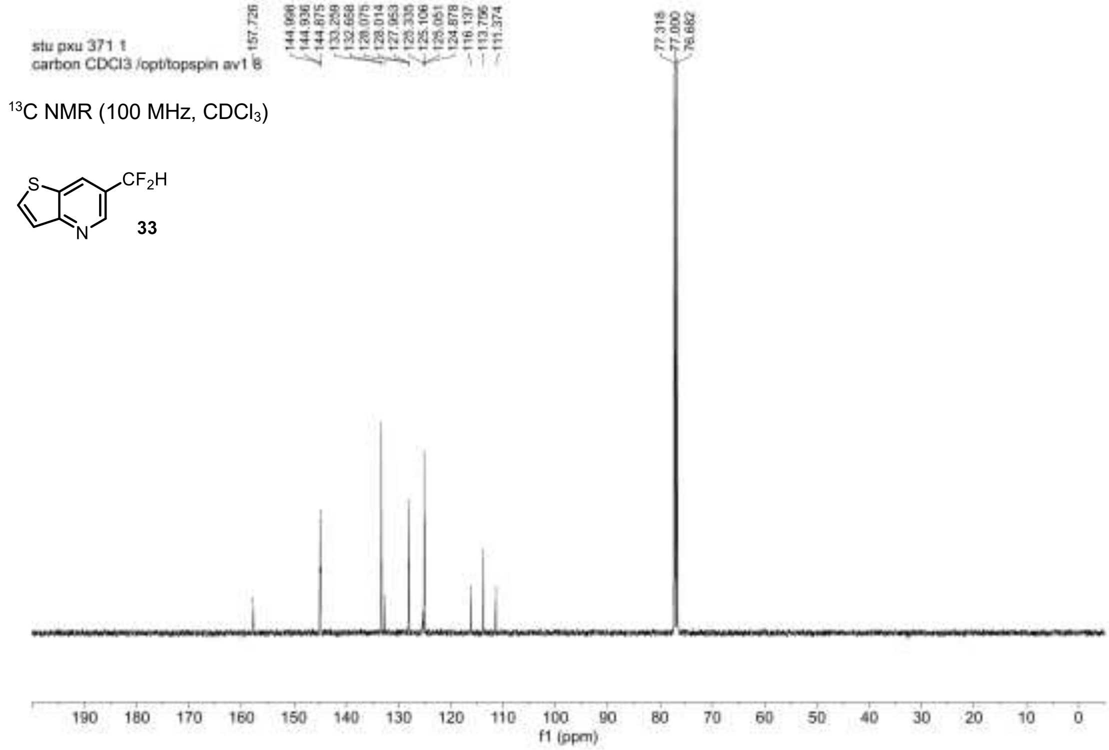

Supplementary Figure 136. ^{13}C NMR (100 MHz, $CDCl_3$) spectrum of compound **33**

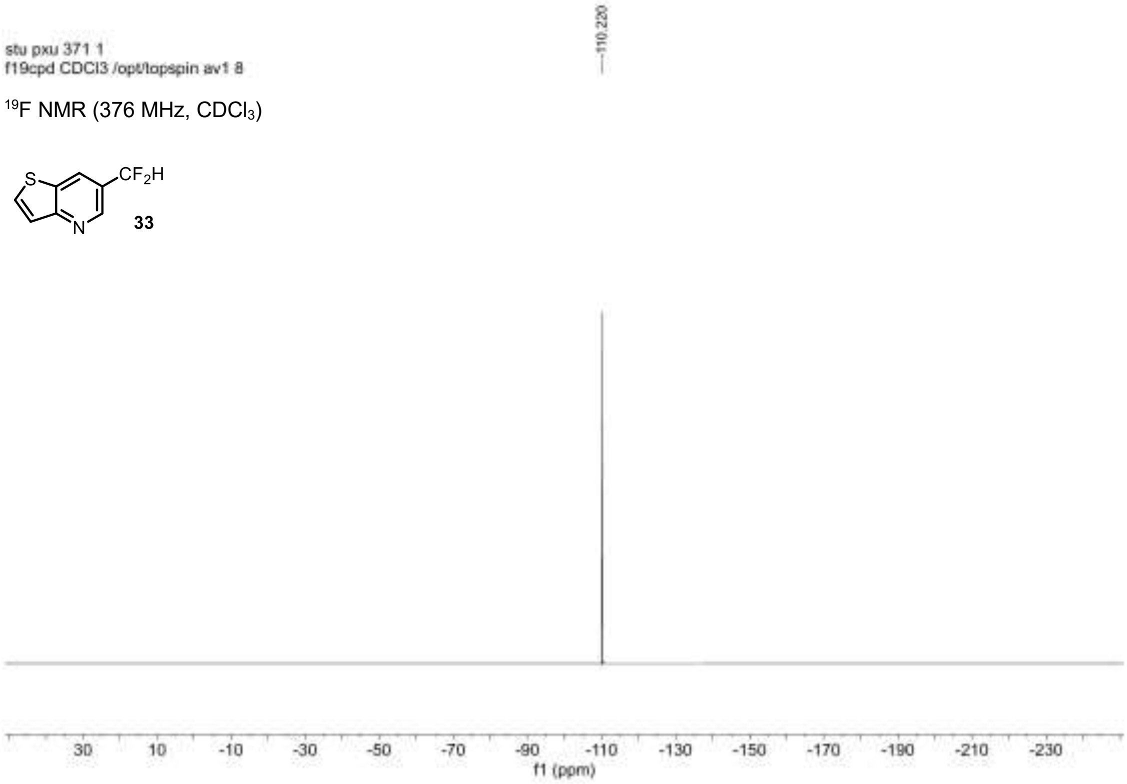

Supplementary Figure 137. ^{19}F NMR (376 MHz, $CDCl_3$) spectrum of compound **33**

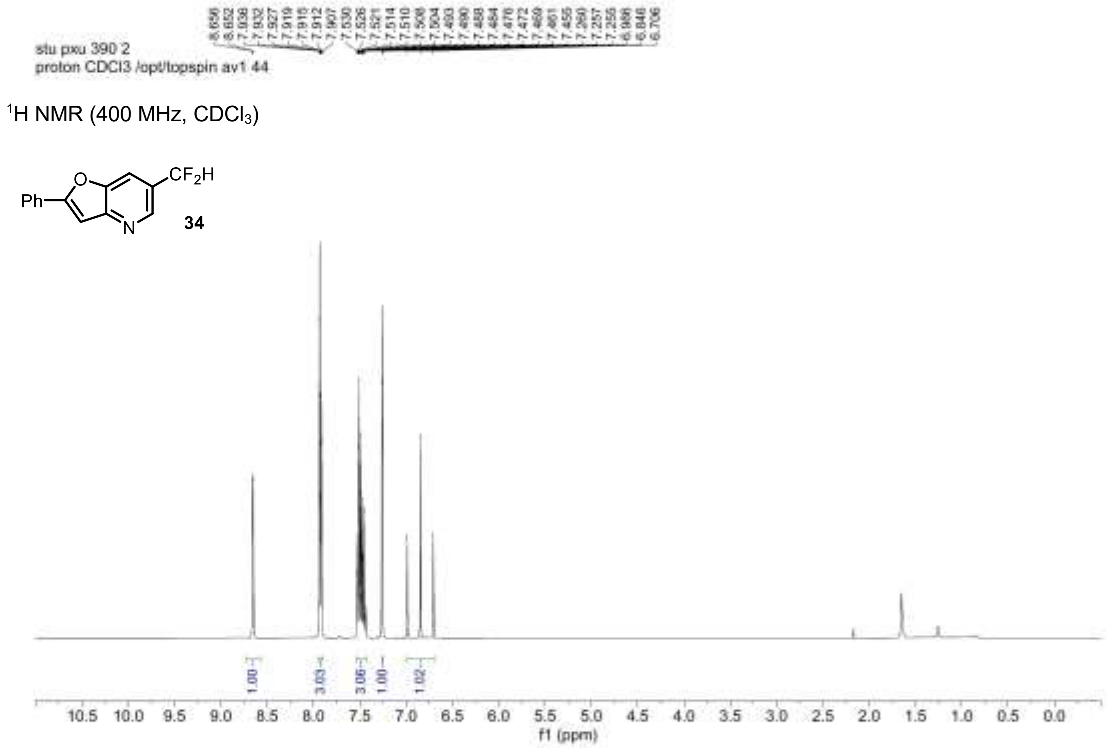

Supplementary Figure 138. ^{1}H NMR (400 MHz, $CDCl_3$) spectrum of compound **34**

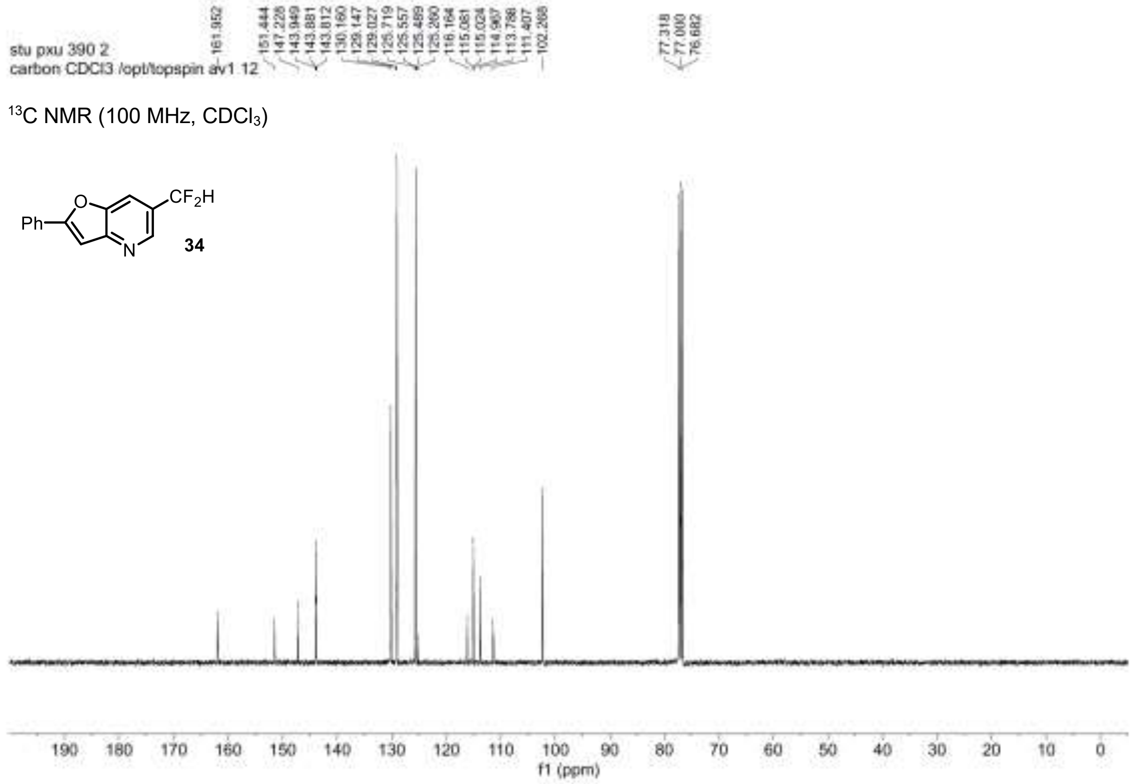

Supplementary Figure 139. ^{13}C NMR (100 MHz, $CDCl_3$) spectrum of compound **34**

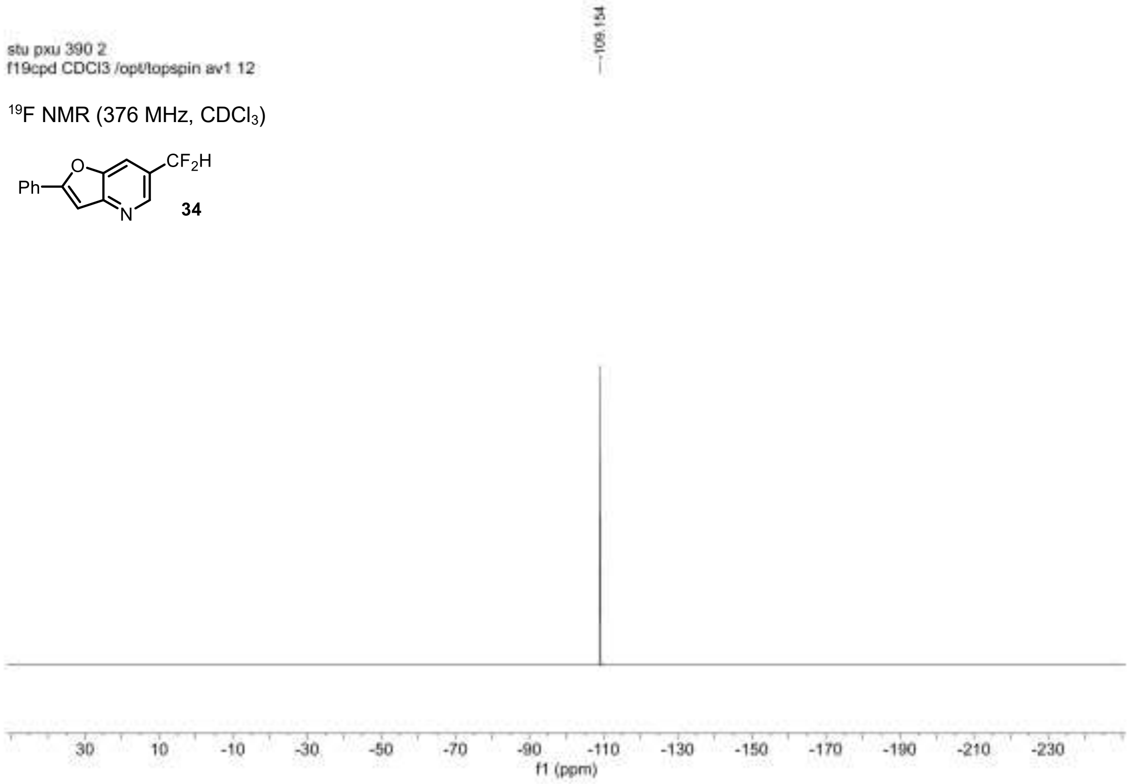

Supplementary Figure 140. ^{19}F NMR (376 MHz, CDCl$_3$) spectrum of compound **34**

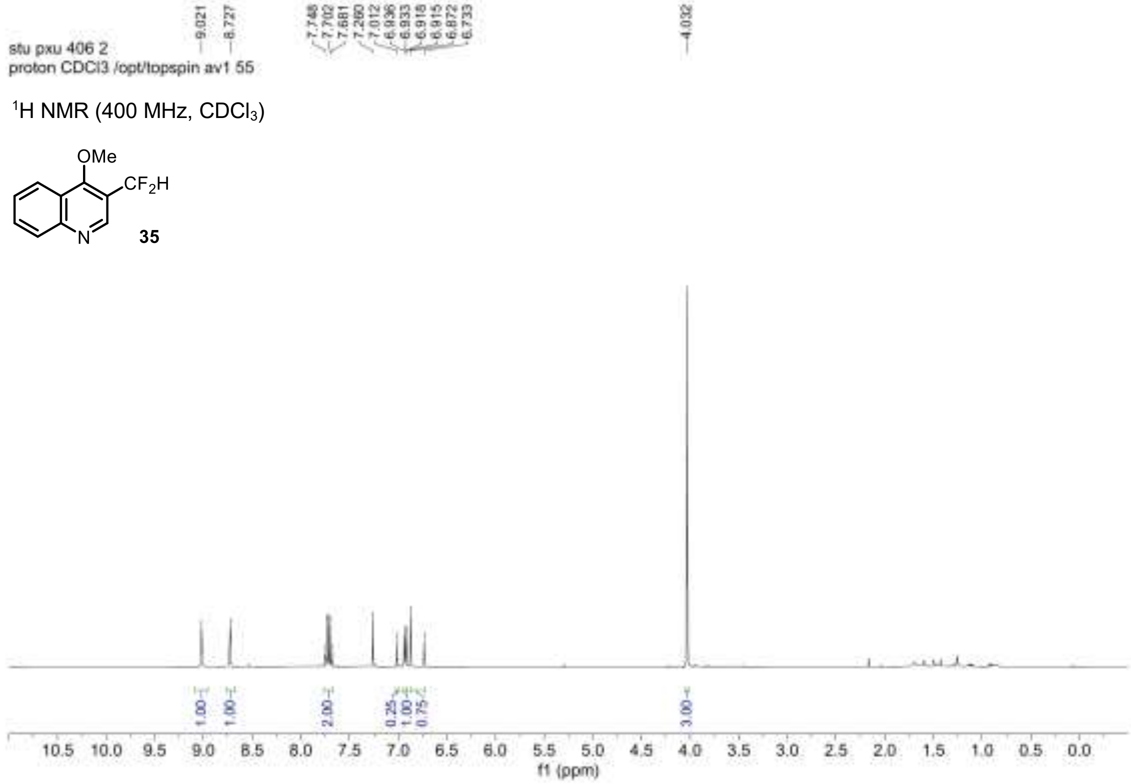

Supplementary Figure 141. ^{1}H NMR (400 MHz, $CDCl_3$) spectrum of compound **35**

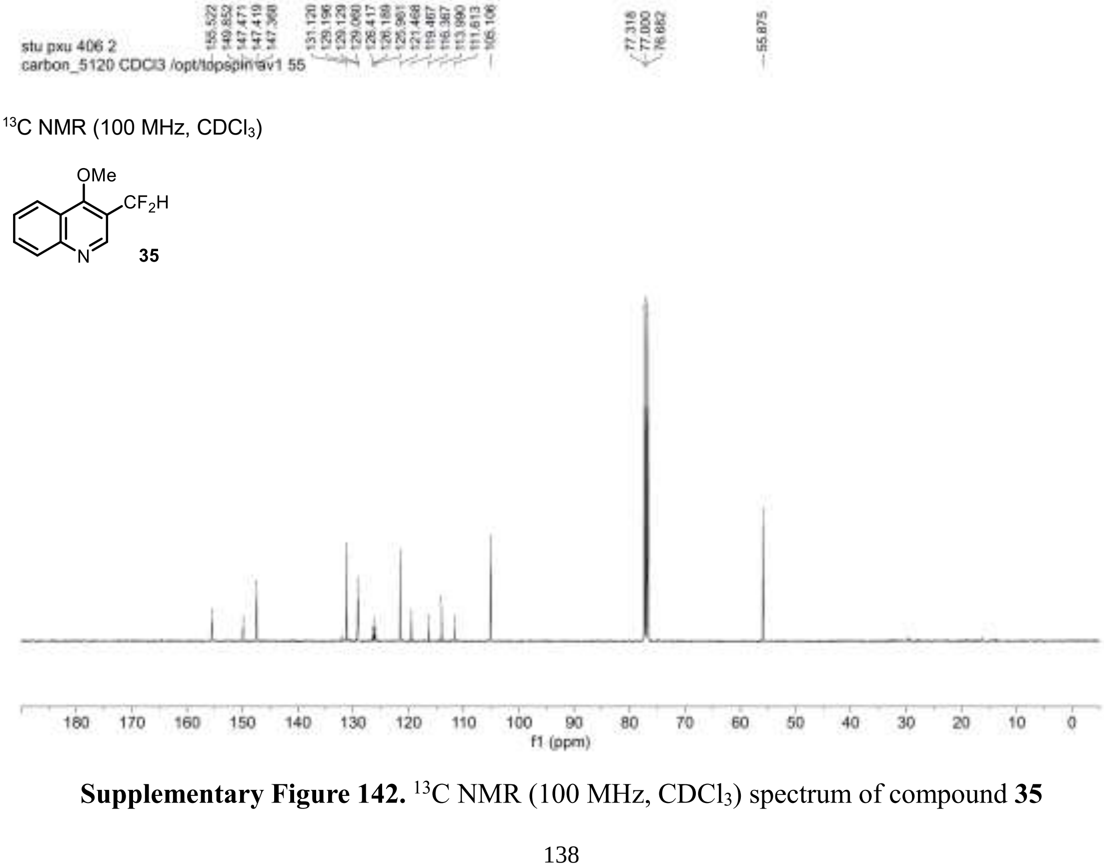

Supplementary Figure 142. ^{13}C NMR (100 MHz, $CDCl_3$) spectrum of compound **35**

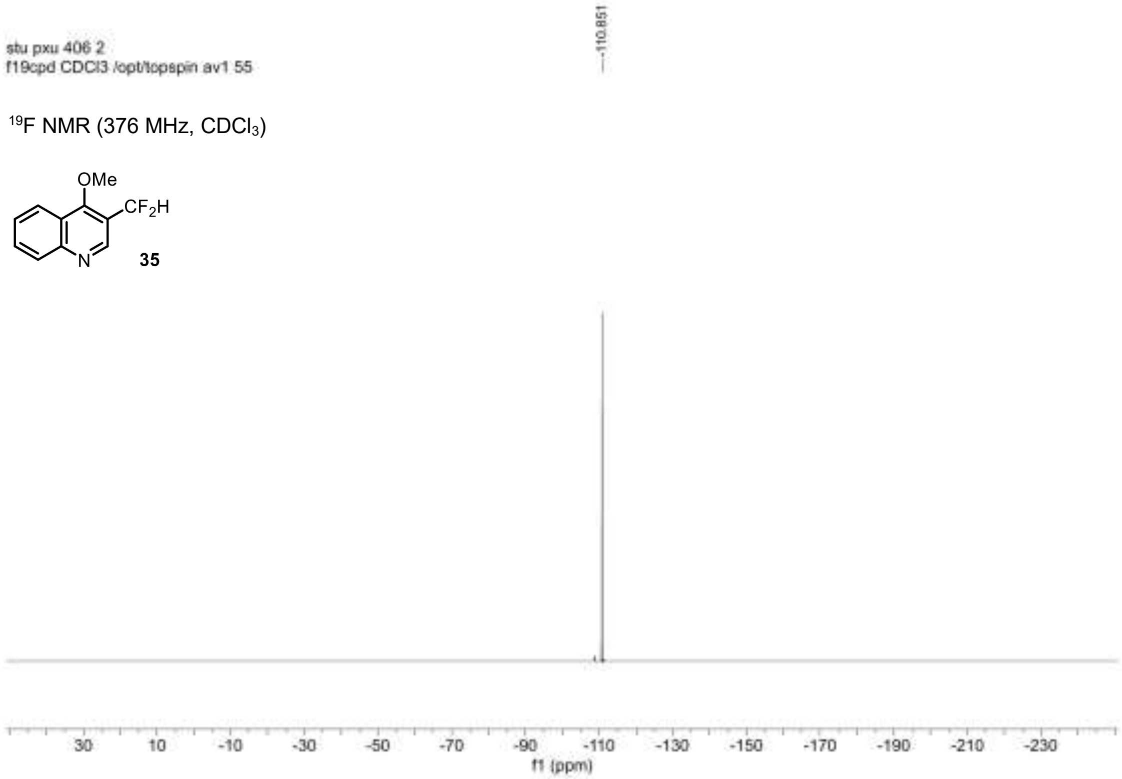

Supplementary Figure 143. ^{19}F NMR (376 MHz, $CDCl_3$) spectrum of compound **35**

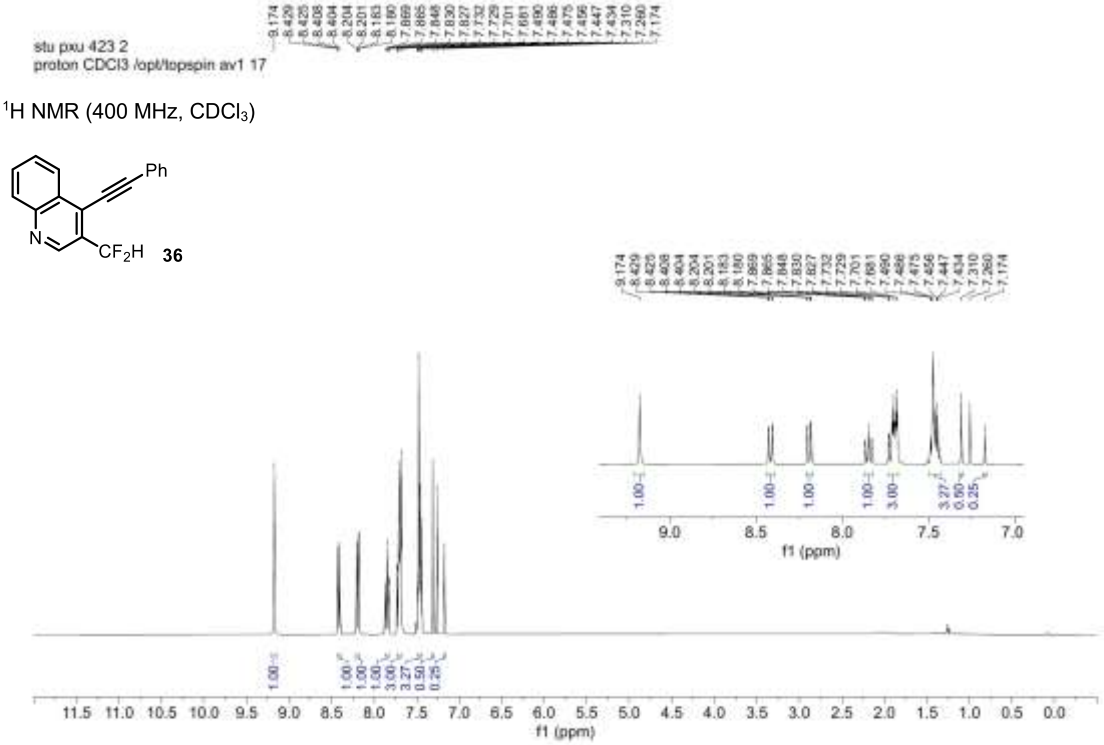

Supplementary Figure 144. ^{1}H NMR (400 MHz, $CDCl_3$) spectrum of compound **36**

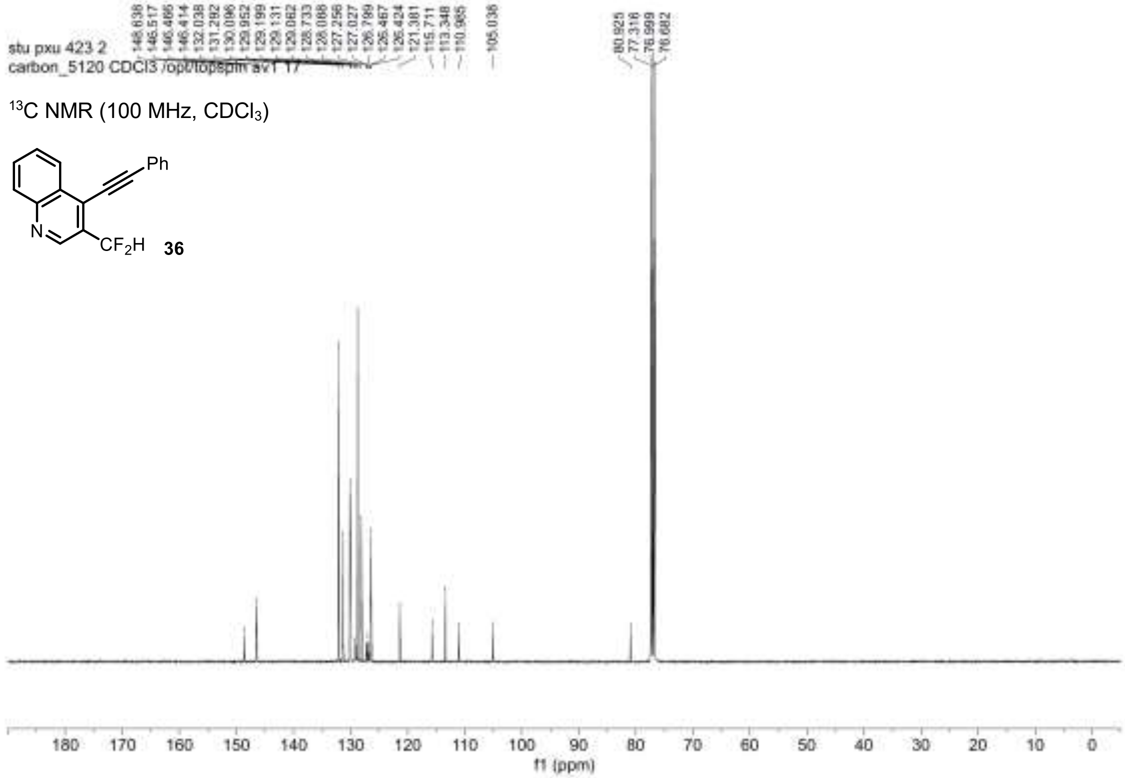

Supplementary Figure 145. ^{13}C NMR (100 MHz, $CDCl_3$) spectrum of compound **36**

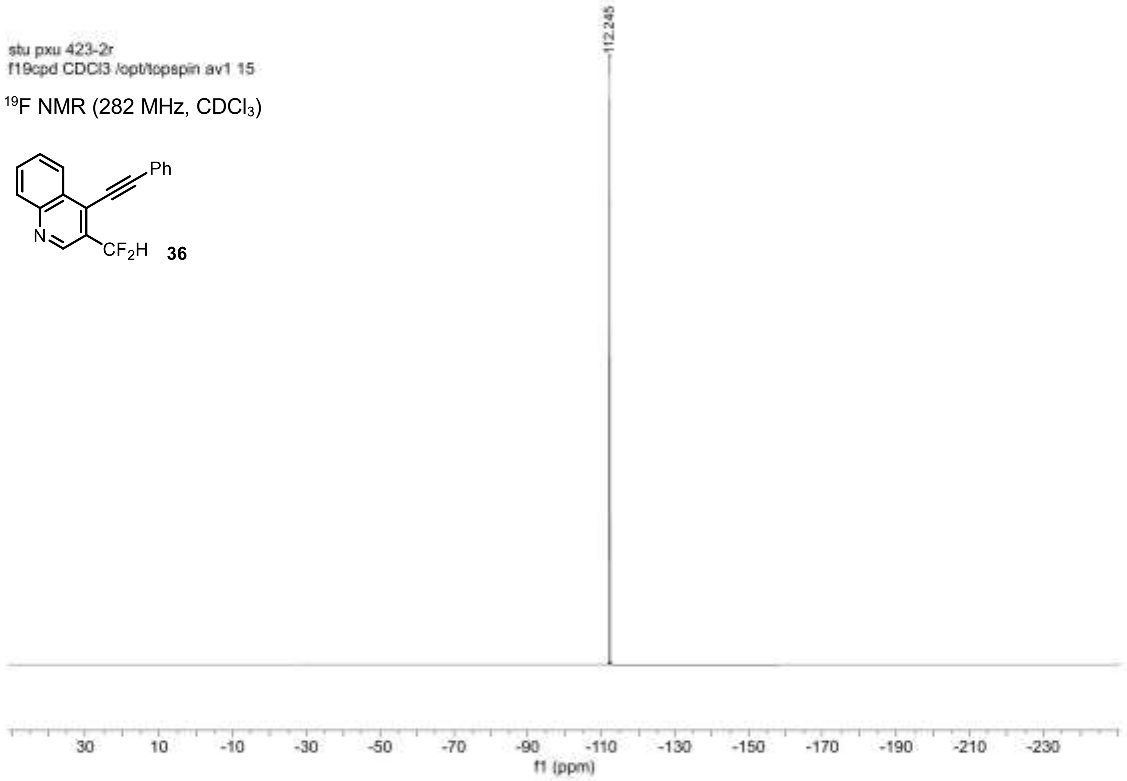

Supplementary Figure 146. ^{19}F NMR (282 MHz, $CDCl_3$) spectrum of compound **36**

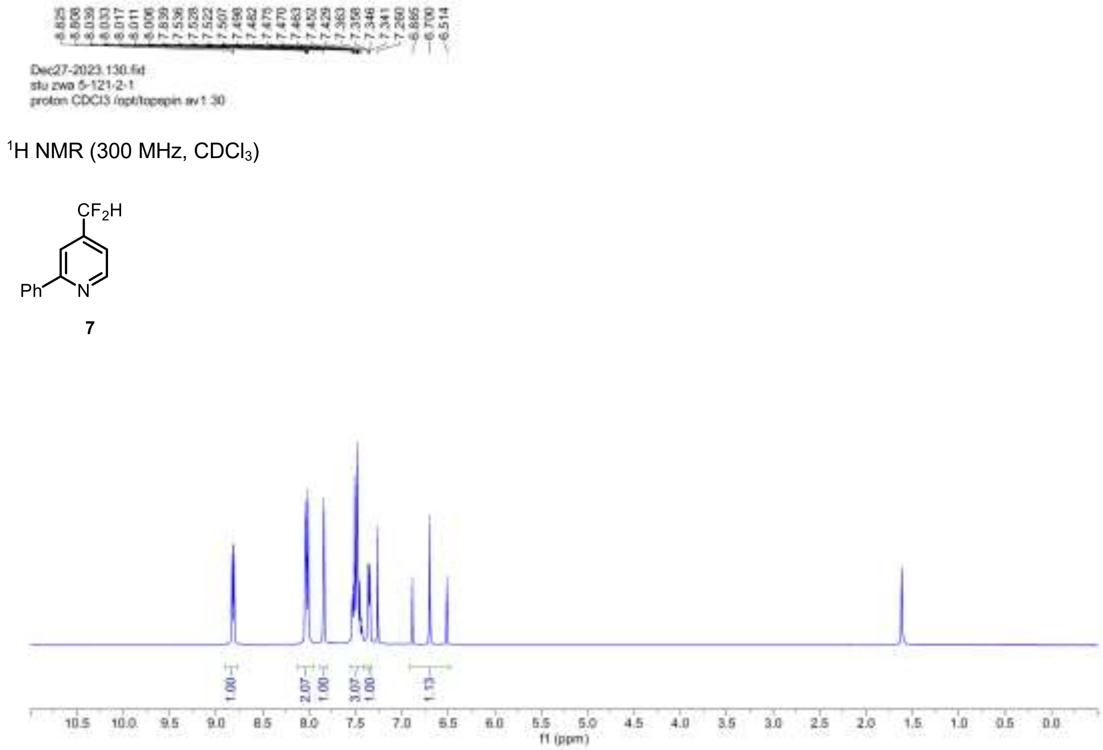

Supplementary Figure 147. ^{1}H NMR (300 MHz, $CDCl_3$) spectrum of compound **7**

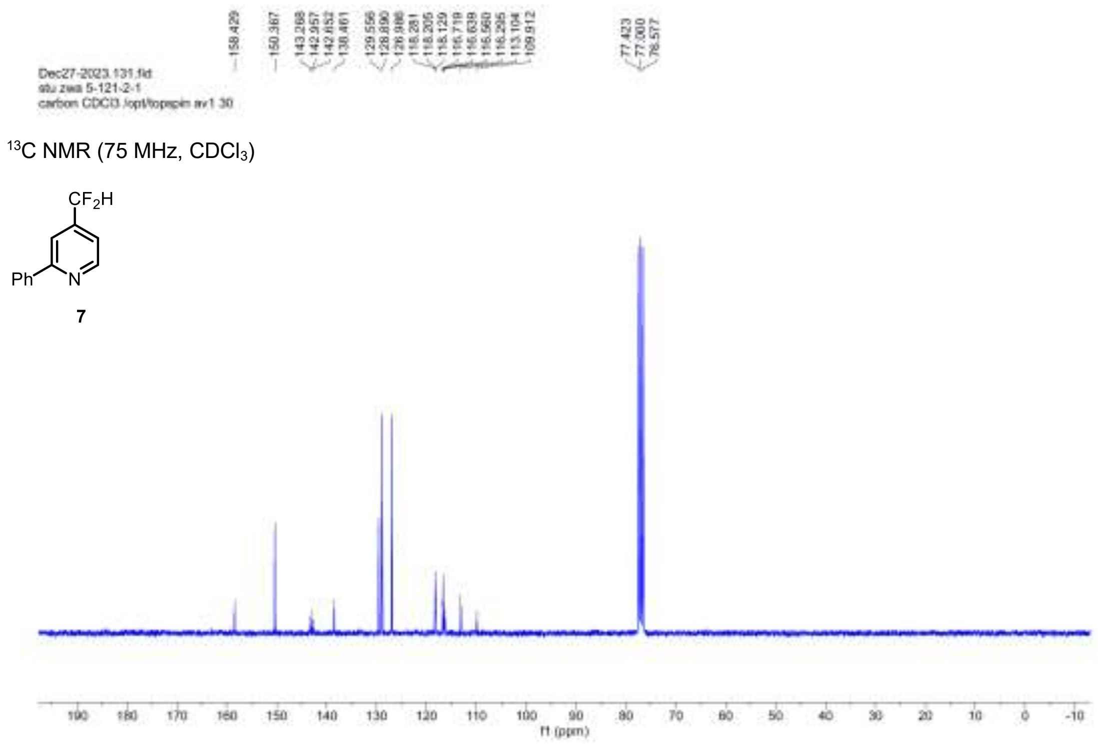

Supplementary Figure 148. ^{13}C NMR (75 MHz, $CDCl_3$) spectrum of compound **7**

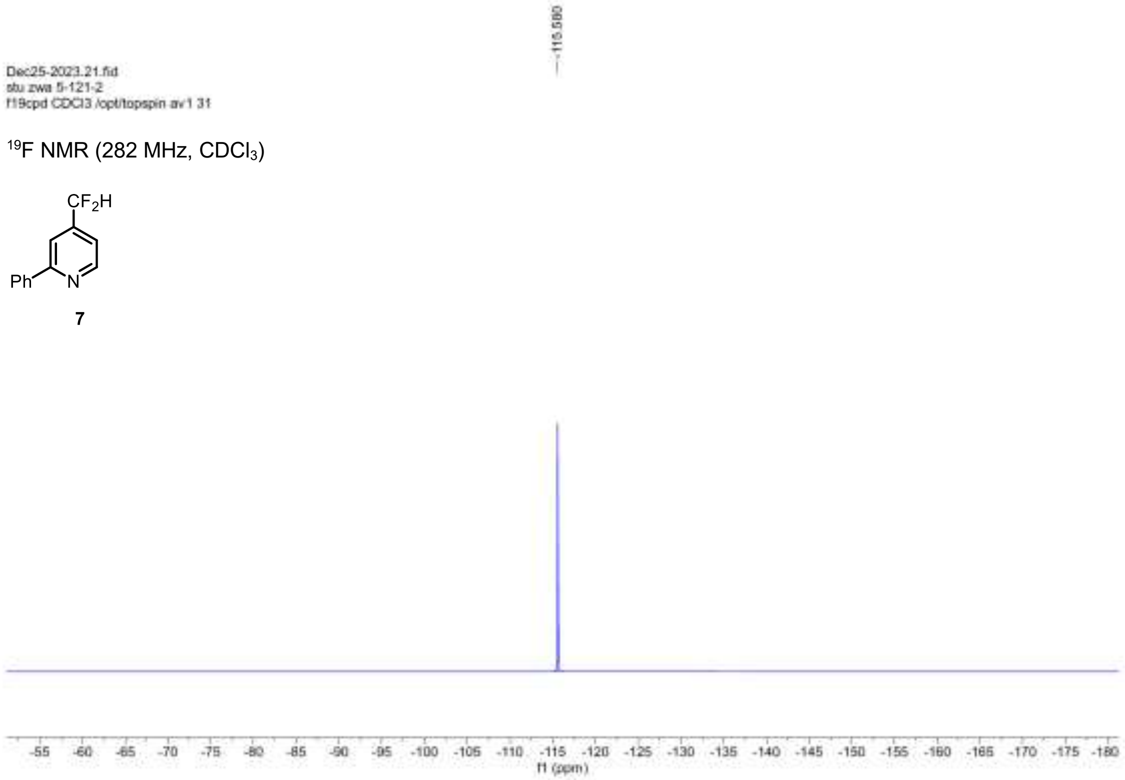

Supplementary Figure 149. ^{19}F NMR (282 MHz, $CDCl_3$) spectrum of compound **7**

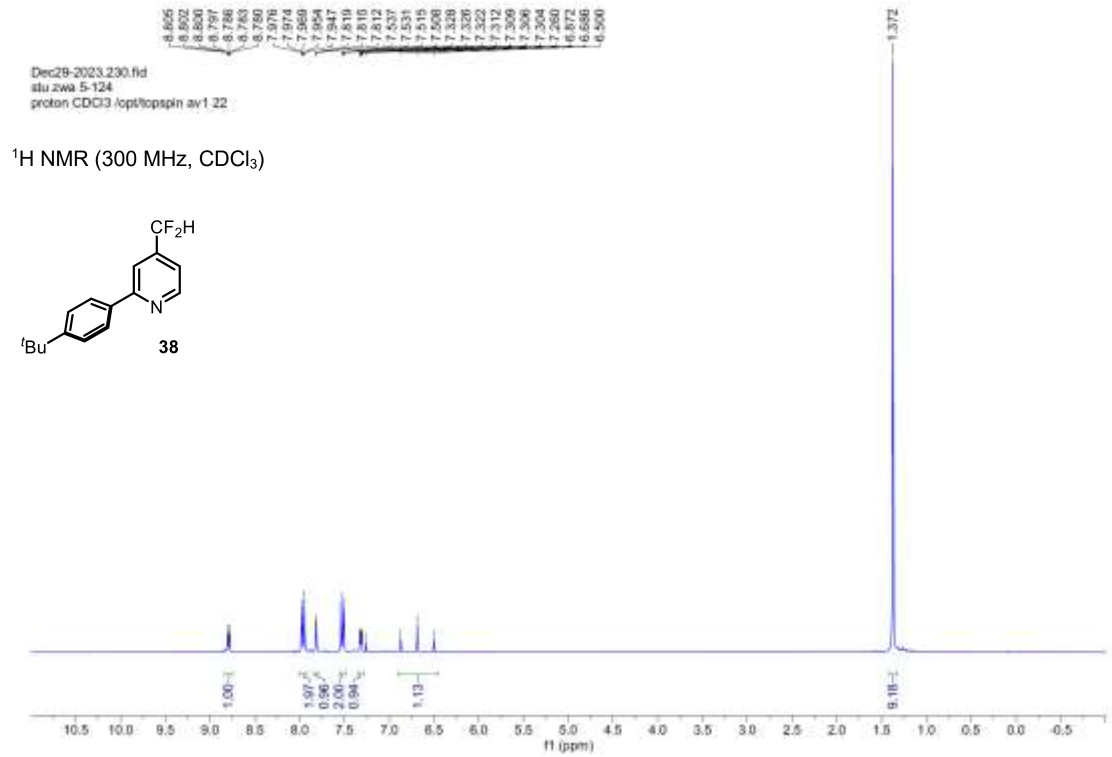

Supplementary Figure 150. ^{1}H NMR (300 MHz, $CDCl_3$) spectrum of compound **38**

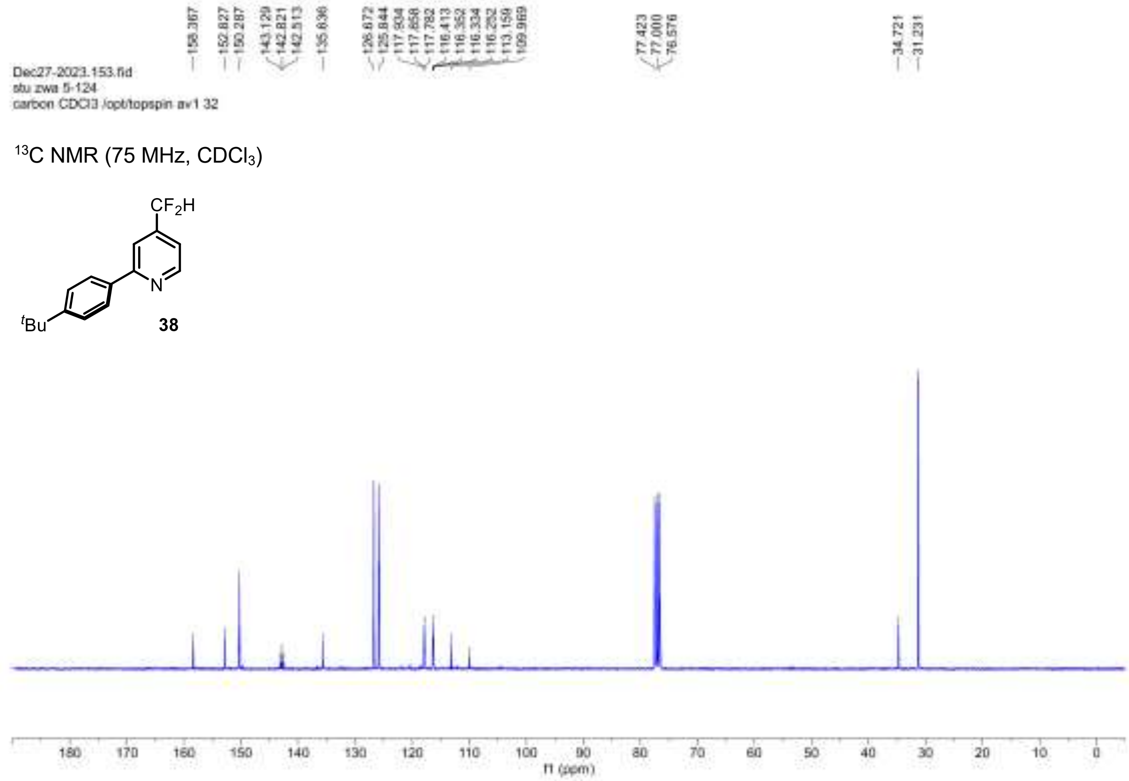

Supplementary Figure 151. ^{13}C NMR (75 MHz, $CDCl_3$) spectrum of compound **38**

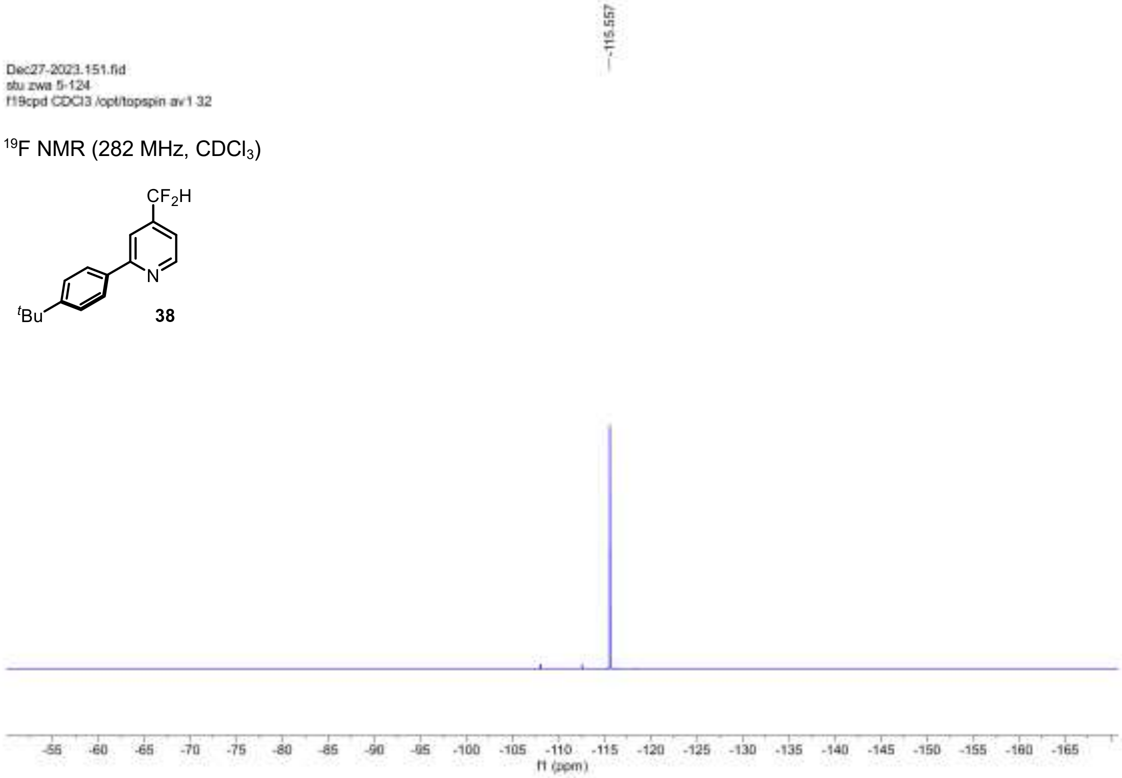

Supplementary Figure 152. ^{19}F NMR (282 MHz, $CDCl_3$) spectrum of compound **38**

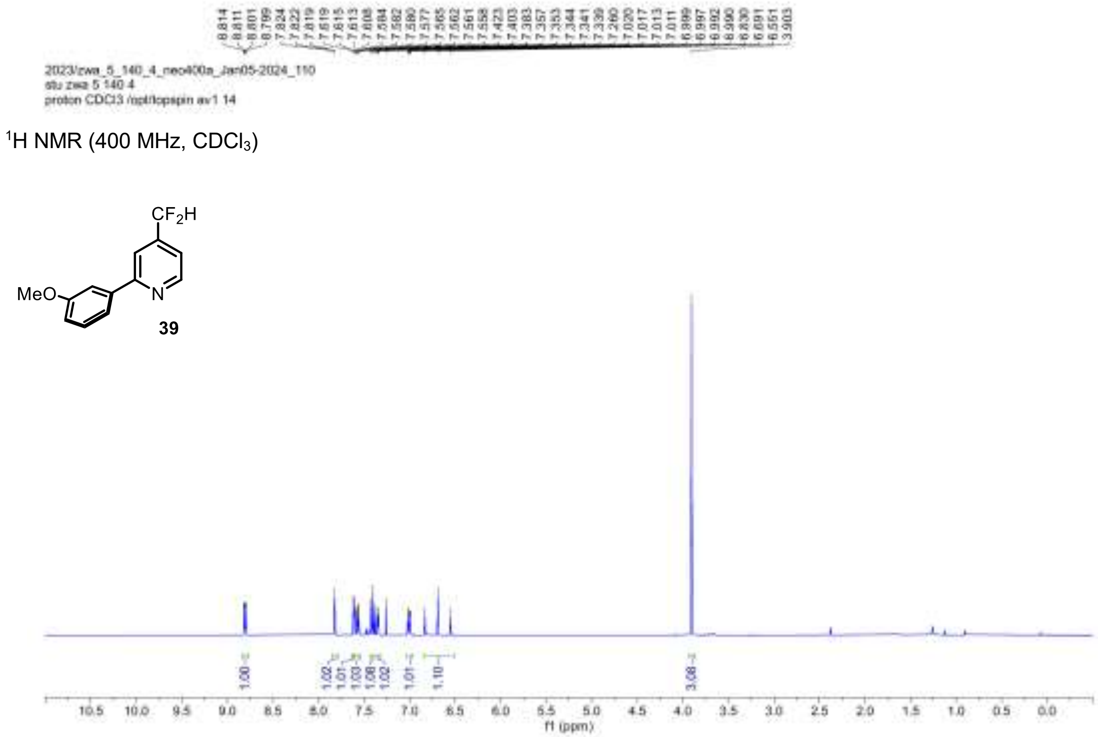

Supplementary Figure 153. ^{1}H NMR (400 MHz, $CDCl_3$) spectrum of compound **39**

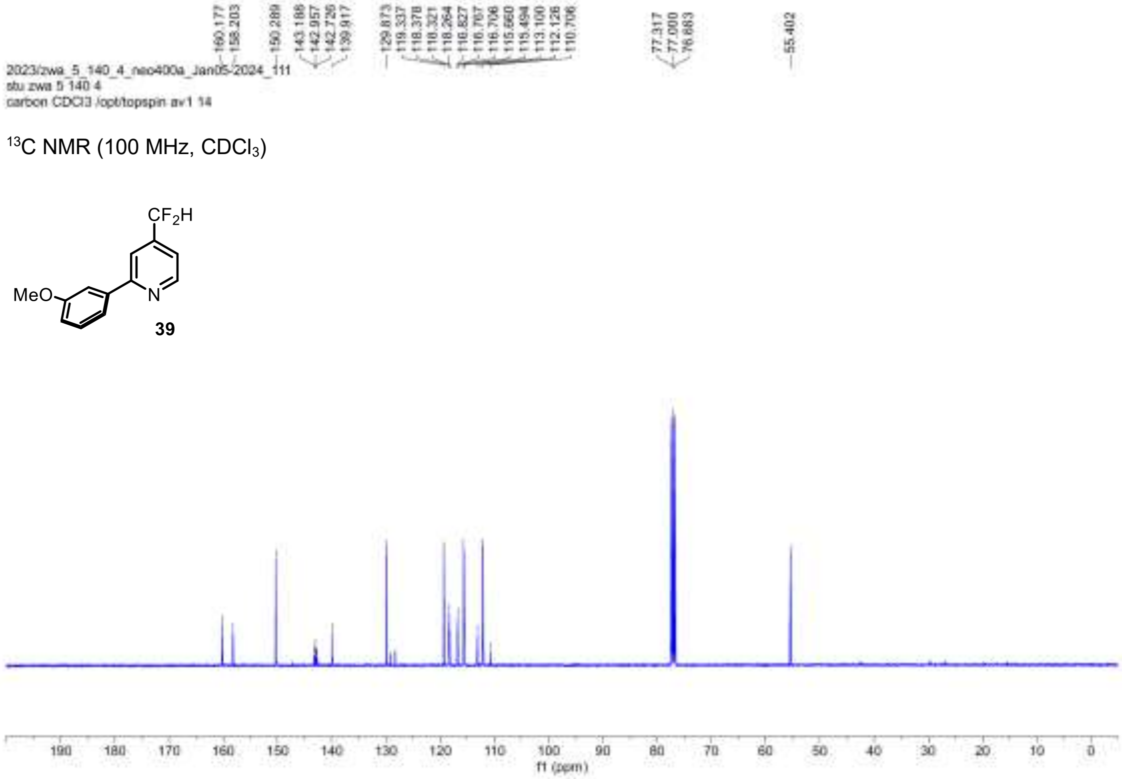

Supplementary Figure 154. ^{13}C NMR (100 MHz, $CDCl_3$) spectrum of compound **39**

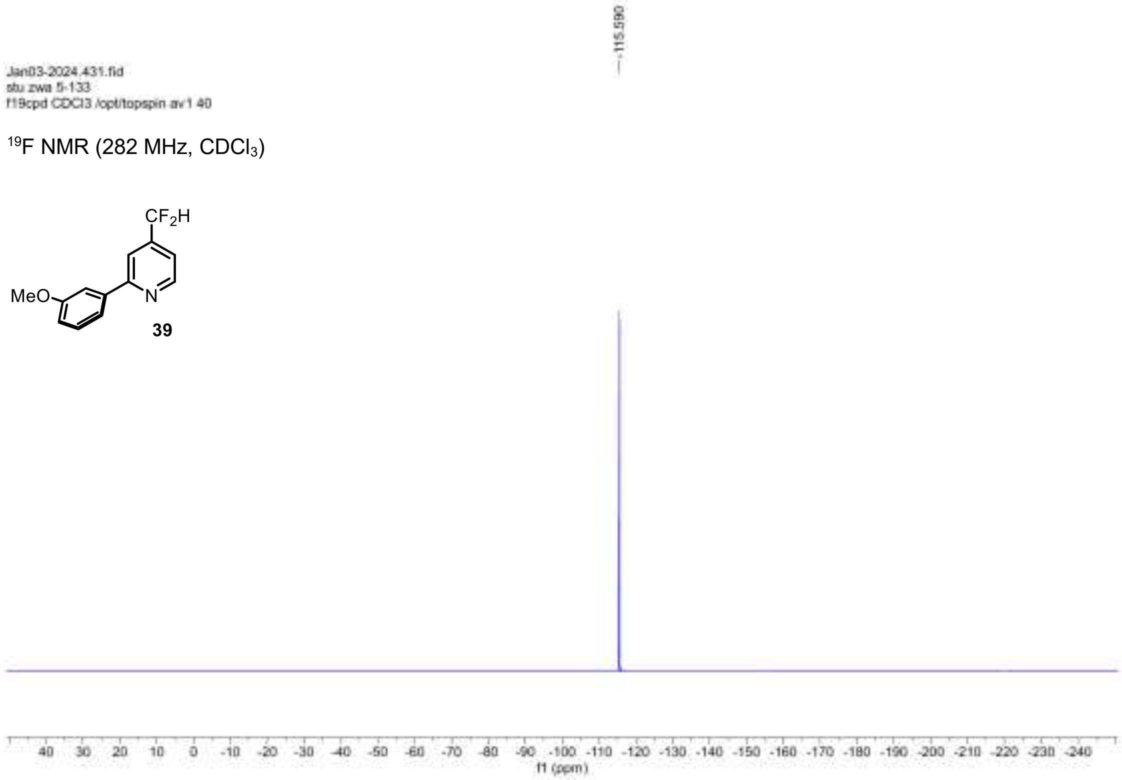

Supplementary Figure 155. ^{19}F NMR (282 MHz, $CDCl_3$) spectrum of compound **39**

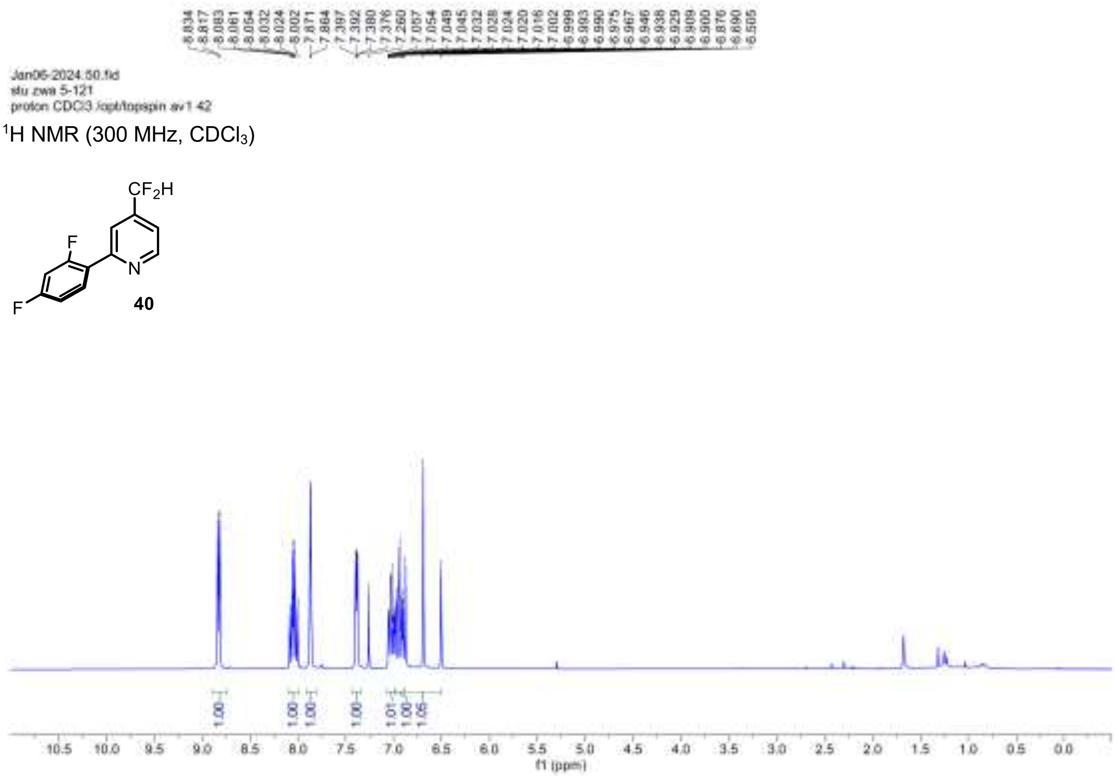

Supplementary Figure 156. ^{1}H NMR (300 MHz, $CDCl_3$) spectrum of compound **40**

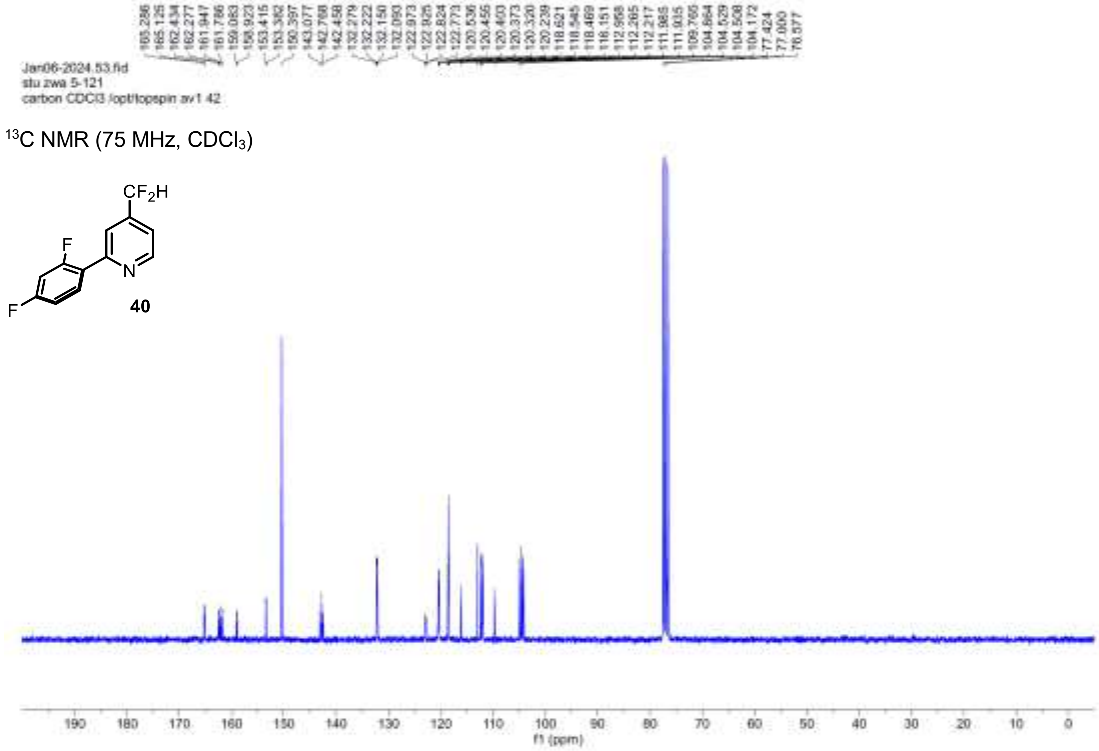

Supplementary Figure 157. ^{13}C NMR (75 MHz, $CDCl_3$) spectrum of compound **40**

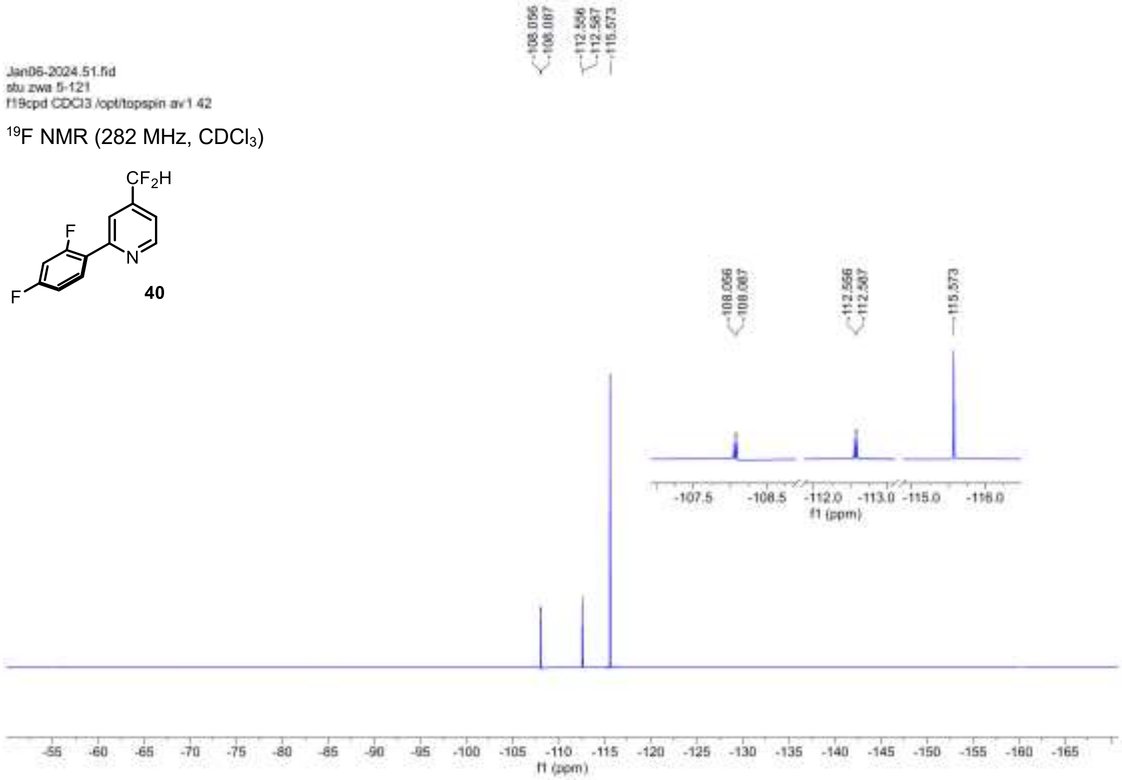

Supplementary Figure 158. ^{19}F NMR (282 MHz, $CDCl_3$) spectrum of compound **40**

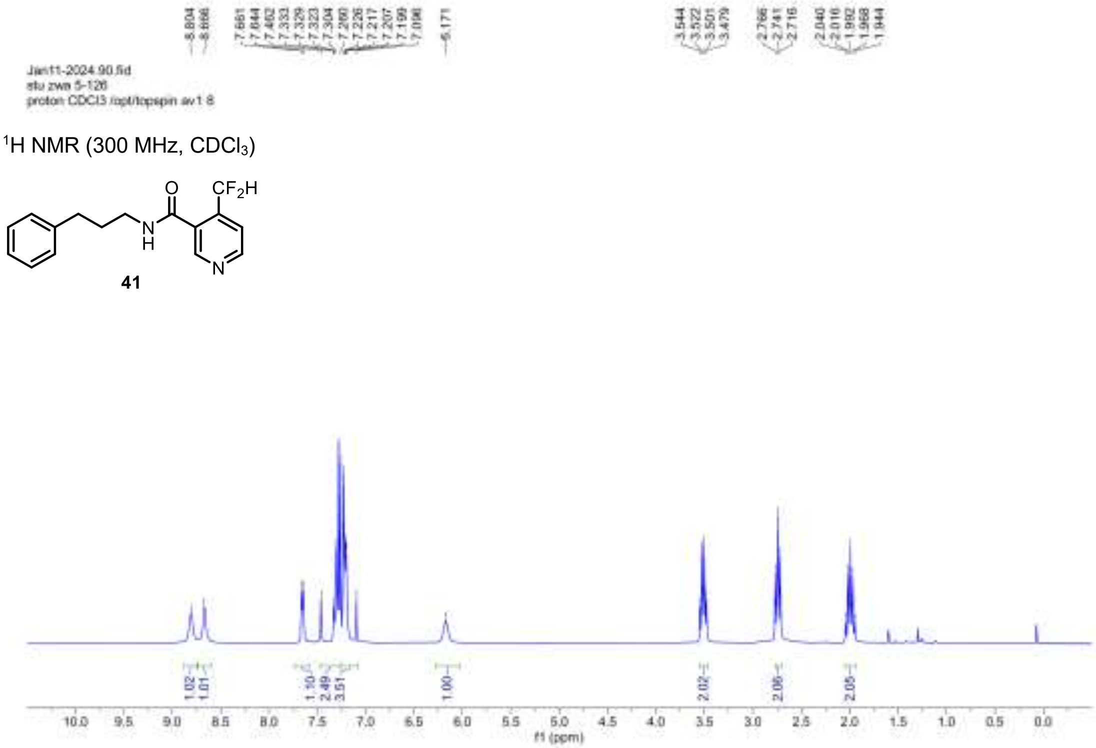

Supplementary Figure 159. ^{1}H NMR (300 MHz, $CDCl_3$) spectrum of compound **41**

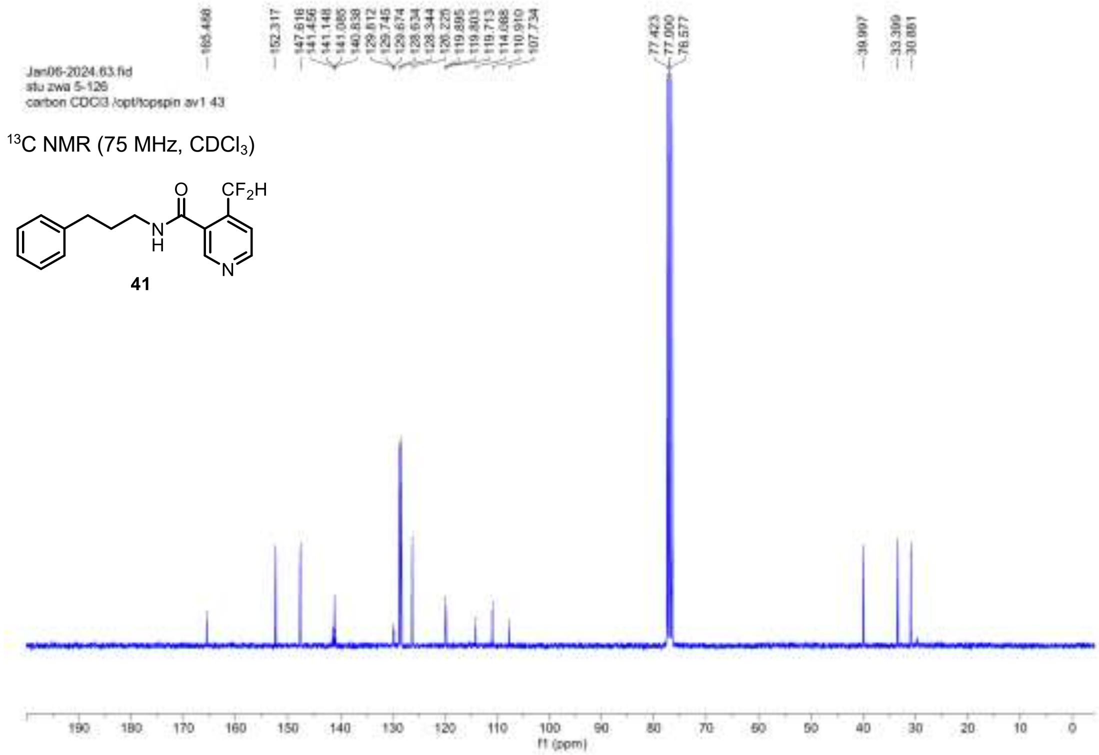

Supplementary Figure 160. ^{13}C NMR (75 MHz, $CDCl_3$) spectrum of compound **41**

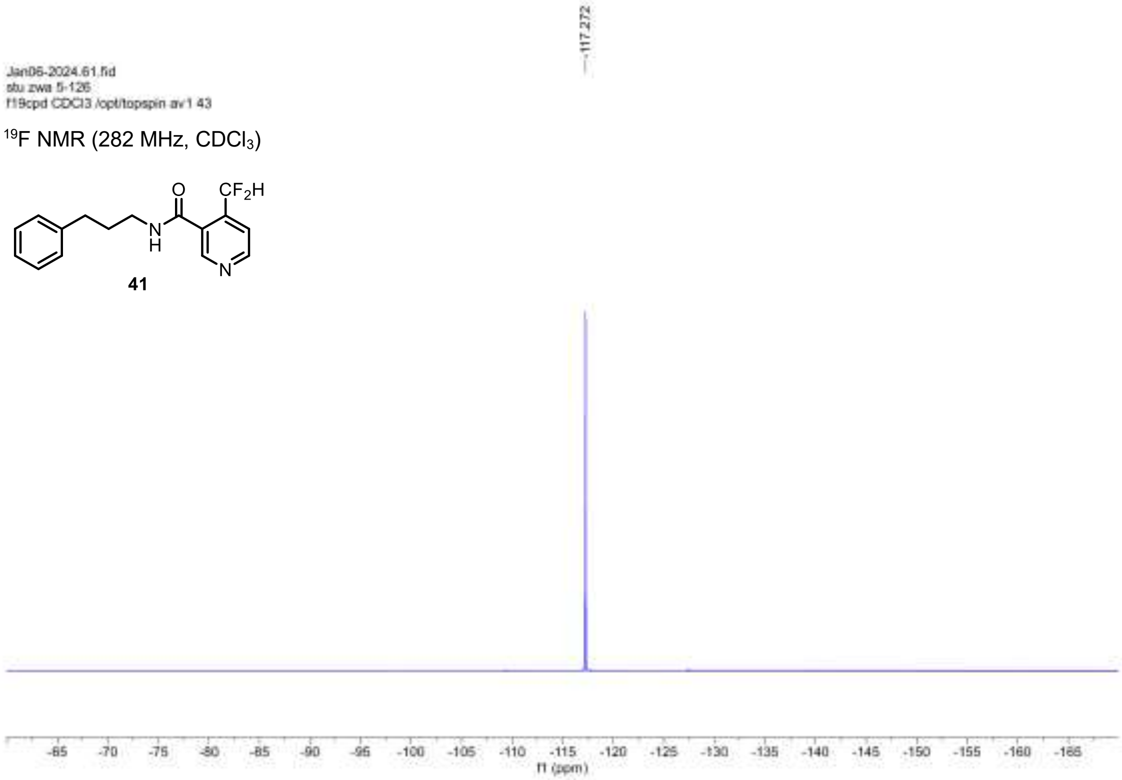

Supplementary Figure 161. ^{19}F NMR (282 MHz, $CDCl_3$) spectrum of compound **41**

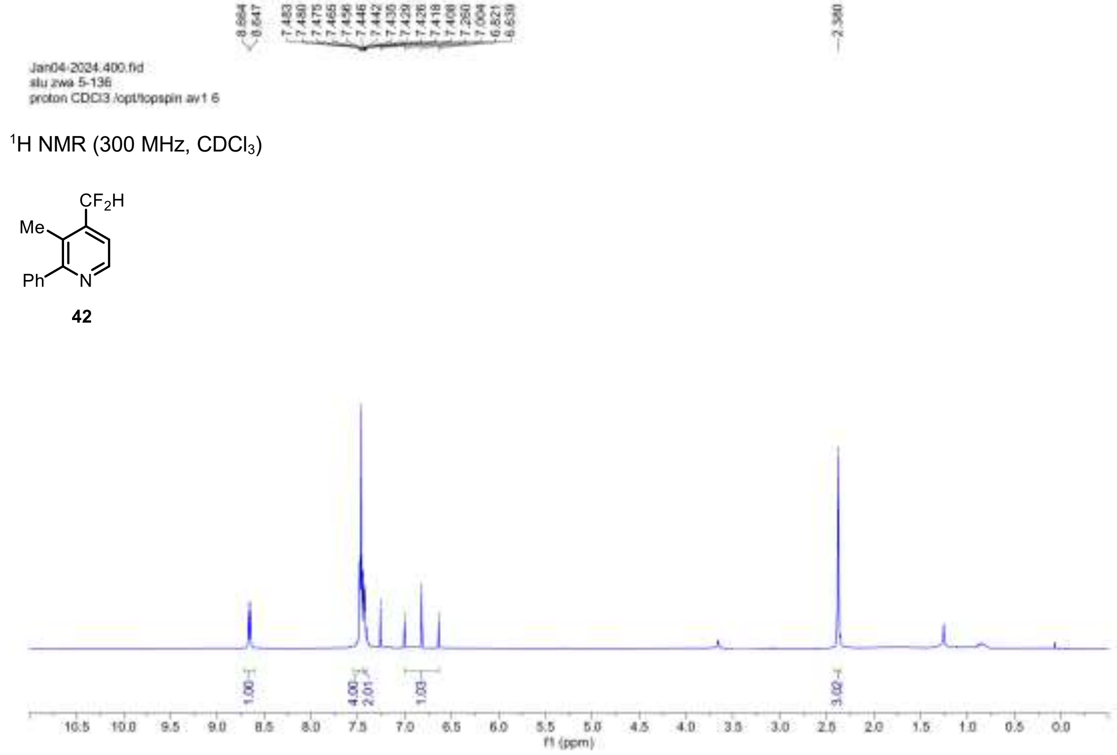

Supplementary Figure 162. ^{1}H NMR (300 MHz, $CDCl_3$) spectrum of compound **42**

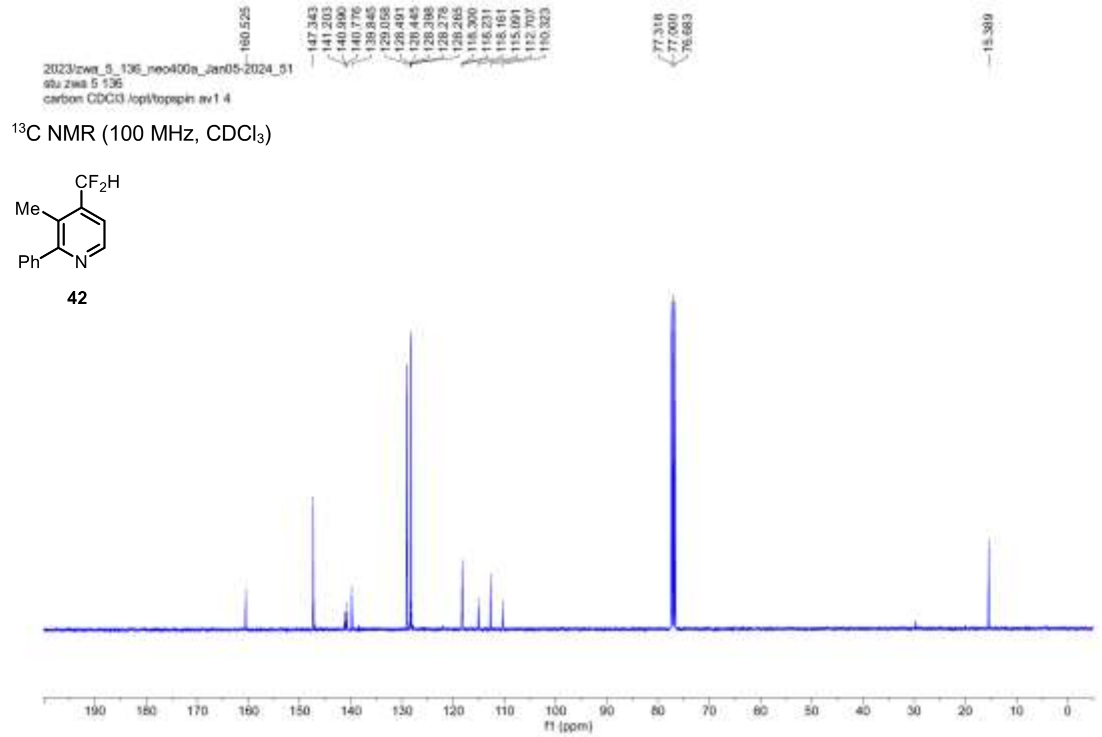

Supplementary Figure 163. ^{13}C NMR (100 MHz, $CDCl_3$) spectrum of compound **42**

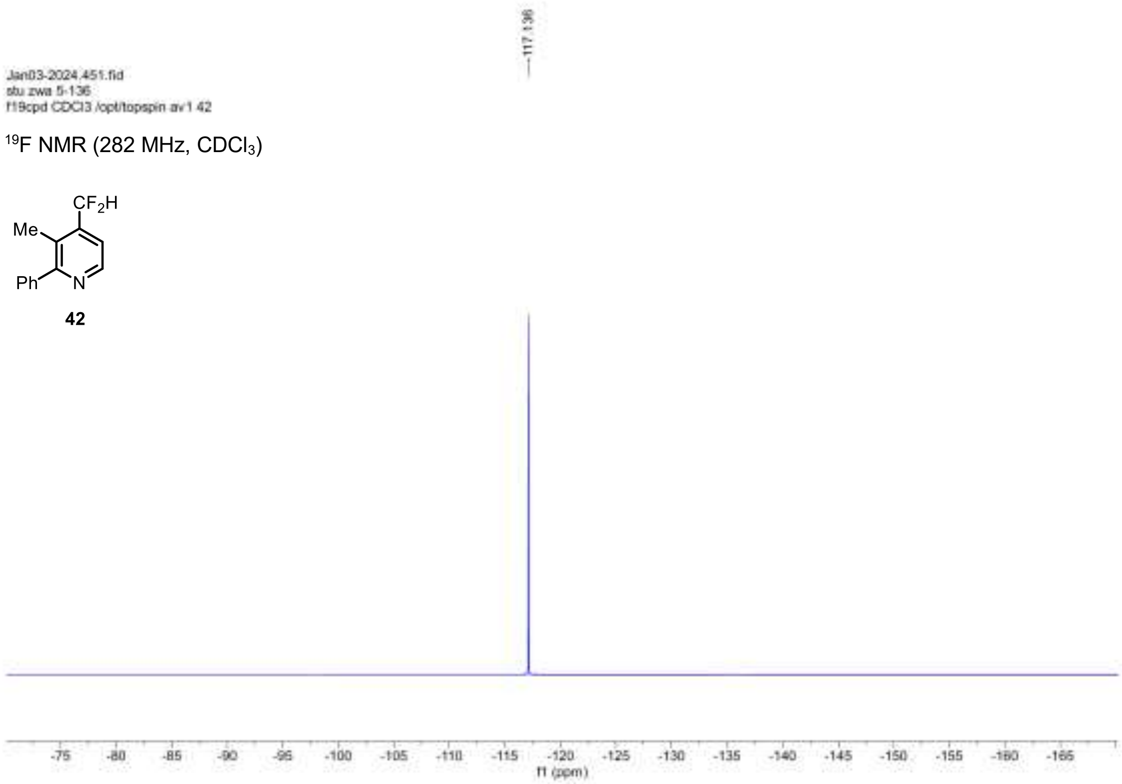

Supplementary Figure 164. ^{19}F NMR (282 MHz, $CDCl_3$) spectrum of compound **42**

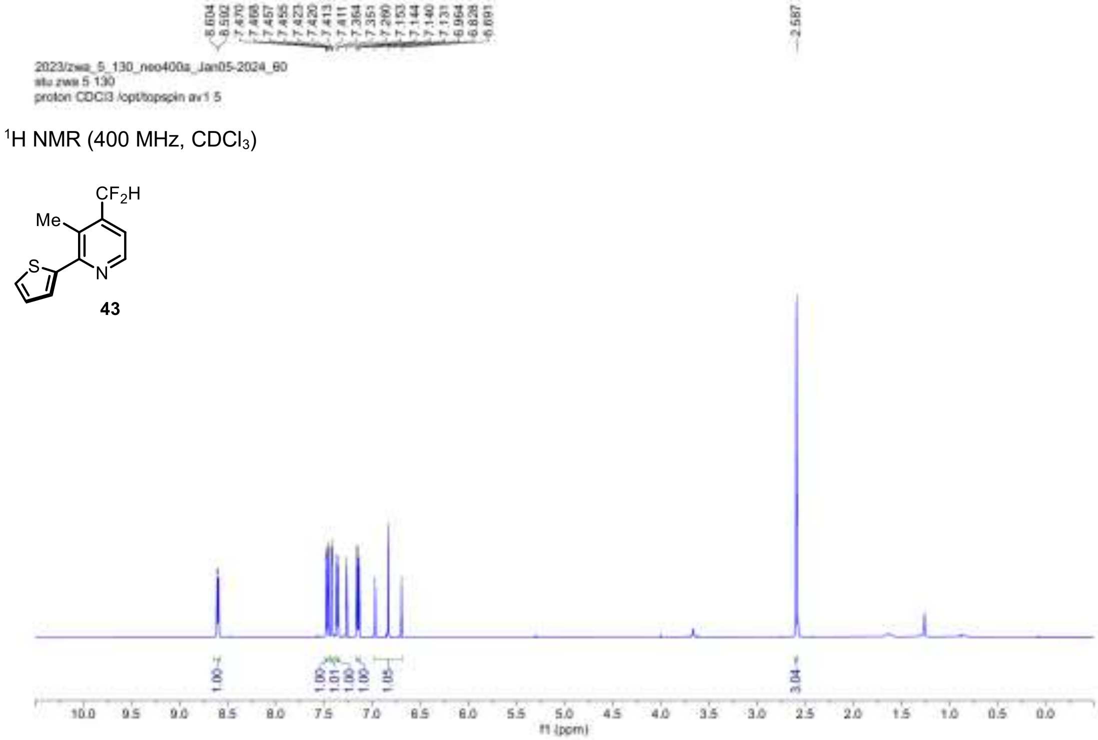

Supplementary Figure 165. ^{1}H NMR (400 MHz, $CDCl_3$) spectrum of compound **43**

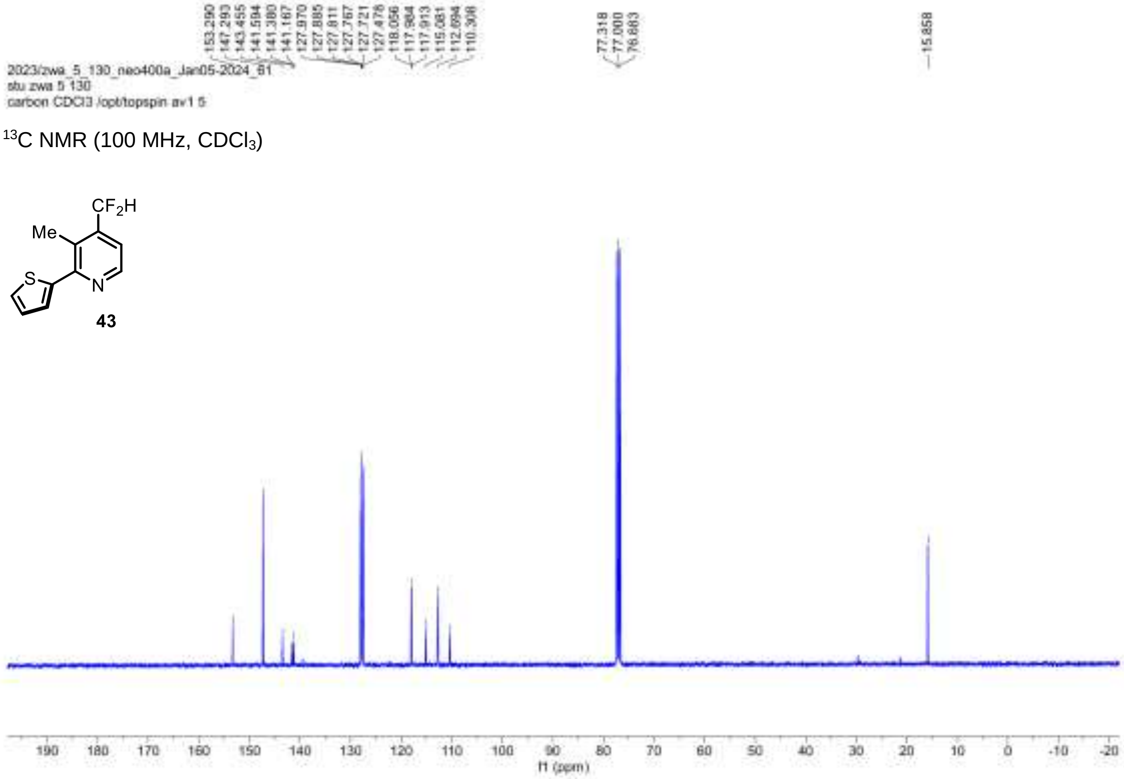

Supplementary Figure 166. ^{13}C NMR (100 MHz, $CDCl_3$) spectrum of compound **43**

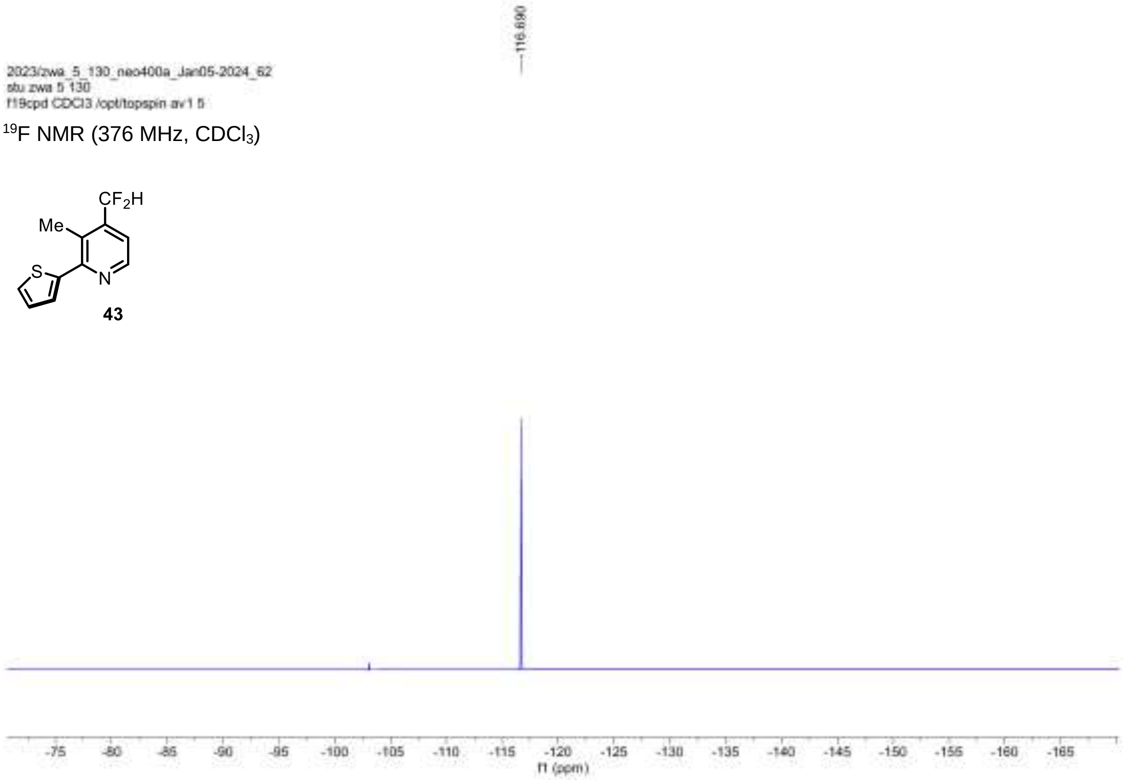

Supplementary Figure 167. ^{19}F NMR (376 MHz, $CDCl_3$) spectrum of compound **43**

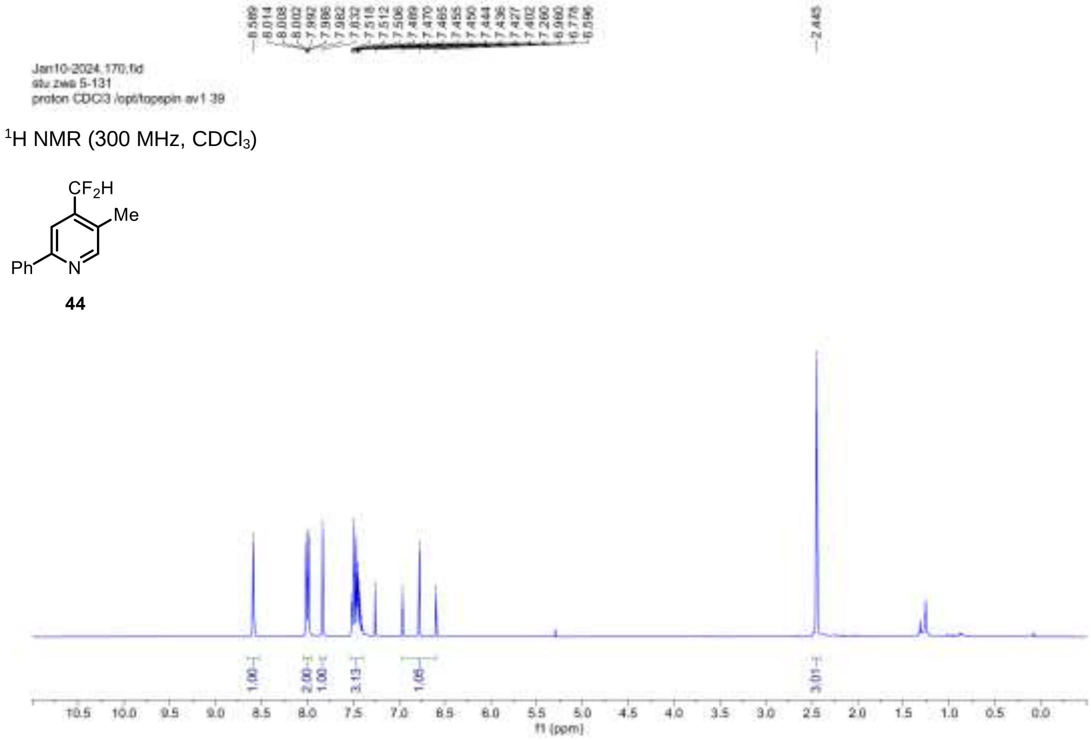

Supplementary Figure 168. ^{1}H NMR (300 MHz, $CDCl_3$) spectrum of compound **44**

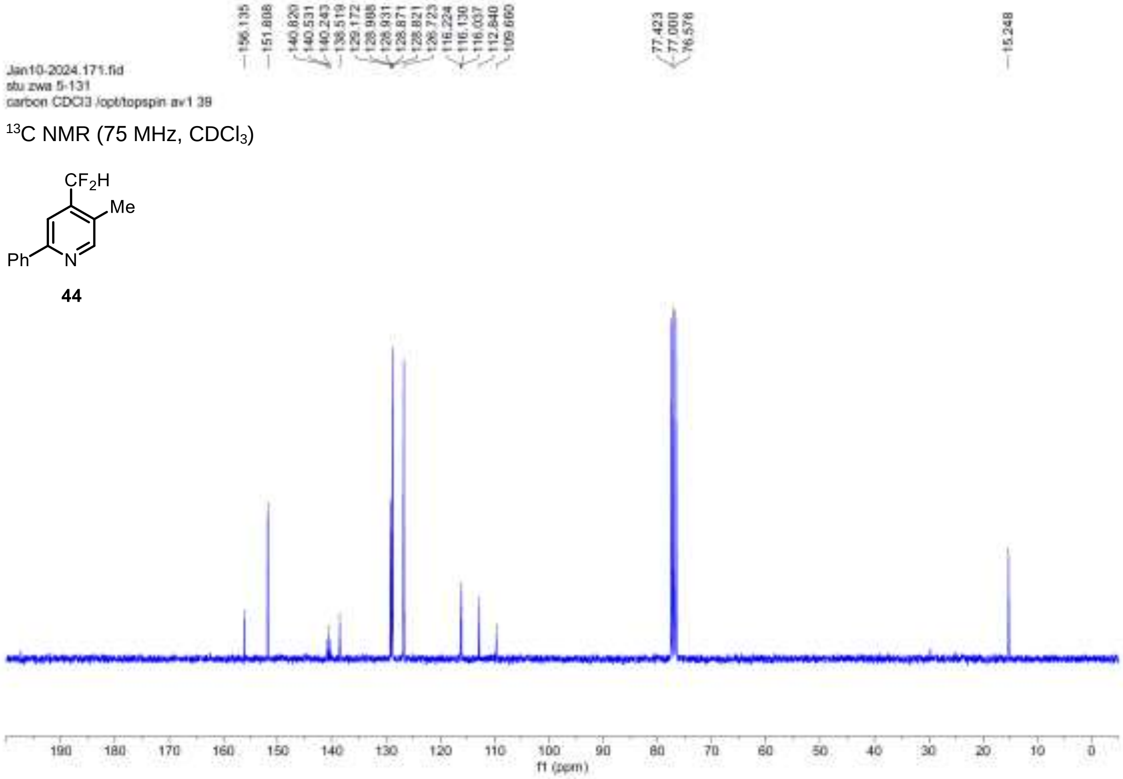

Supplementary Figure 169. ^{13}C NMR (75 MHz, $CDCl_3$) spectrum of compound **44**

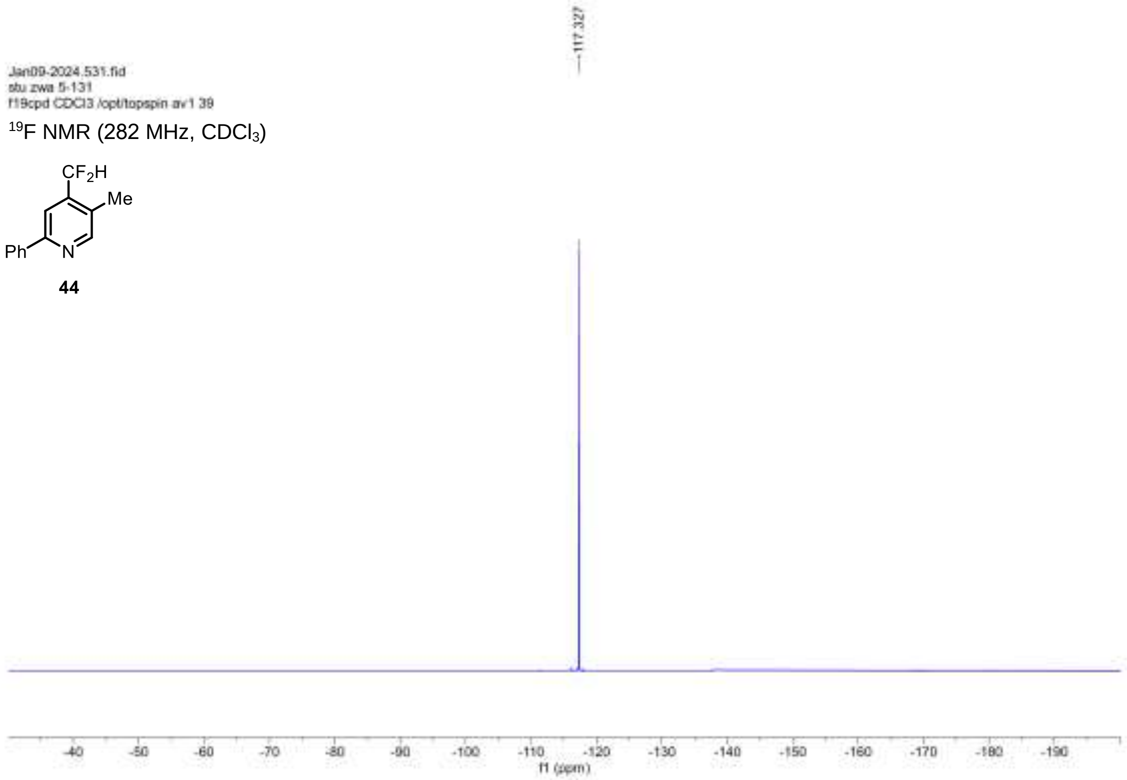

Supplementary Figure 170. ^{19}F NMR (282 MHz, $CDCl_3$) spectrum of compound **44**

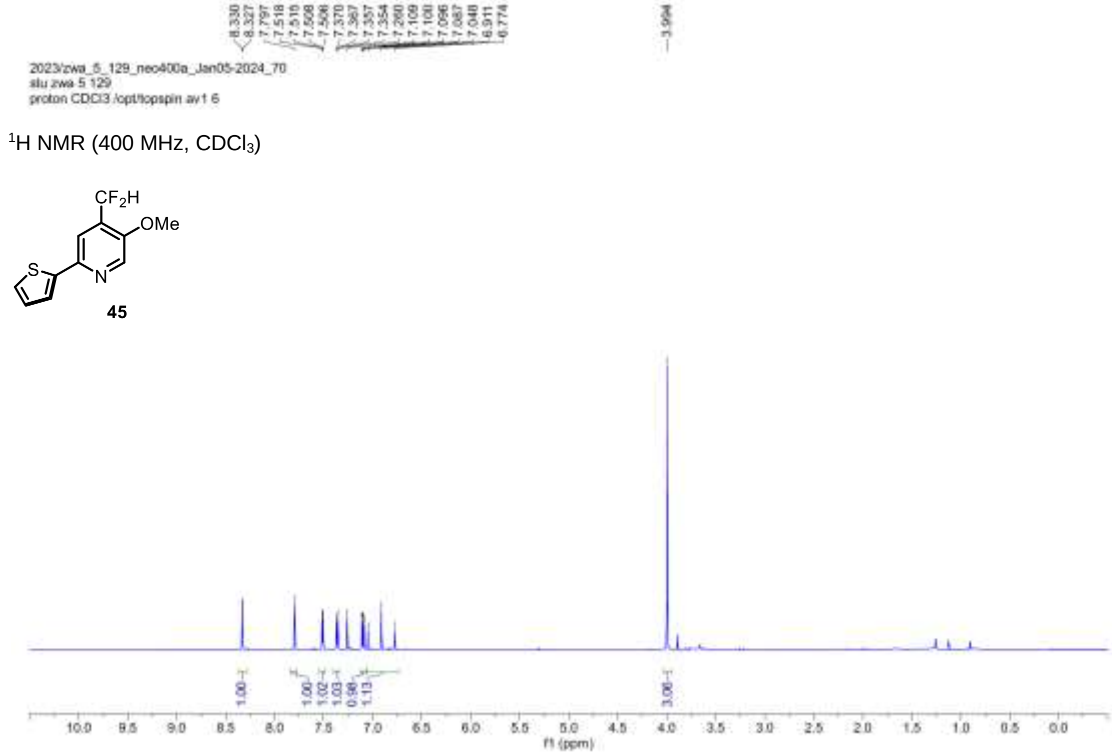

Supplementary Figure 171. ^{1}H NMR (400 MHz, $CDCl_3$) spectrum of compound **45**

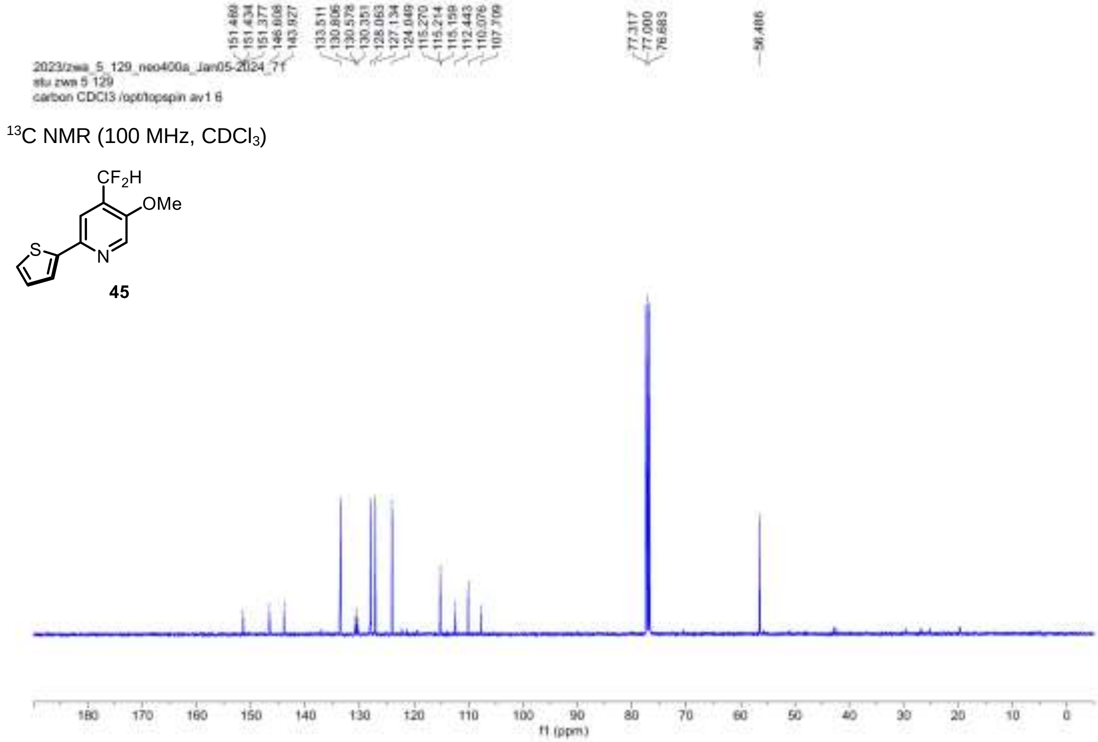

Supplementary Figure 172. ^{13}C NMR (100 MHz, $CDCl_3$) spectrum of compound **45**

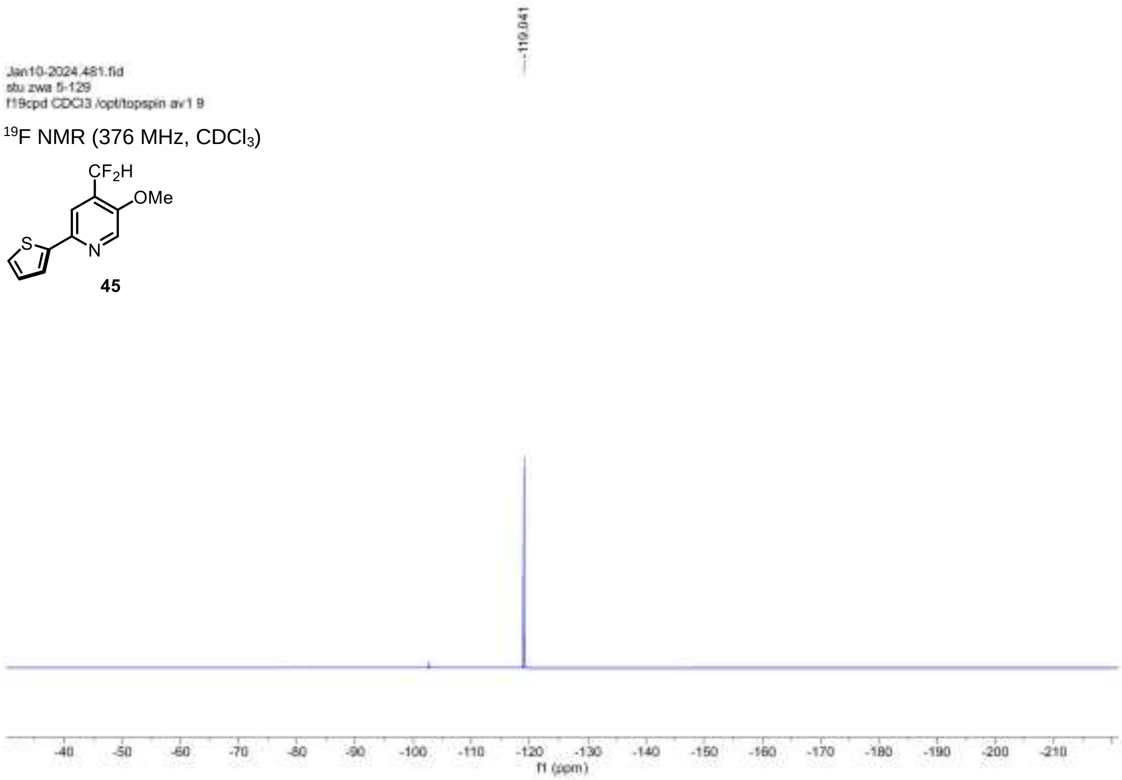

Supplementary Figure 173. ^{19}F NMR (282 MHz, $CDCl_3$) spectrum of compound **45**

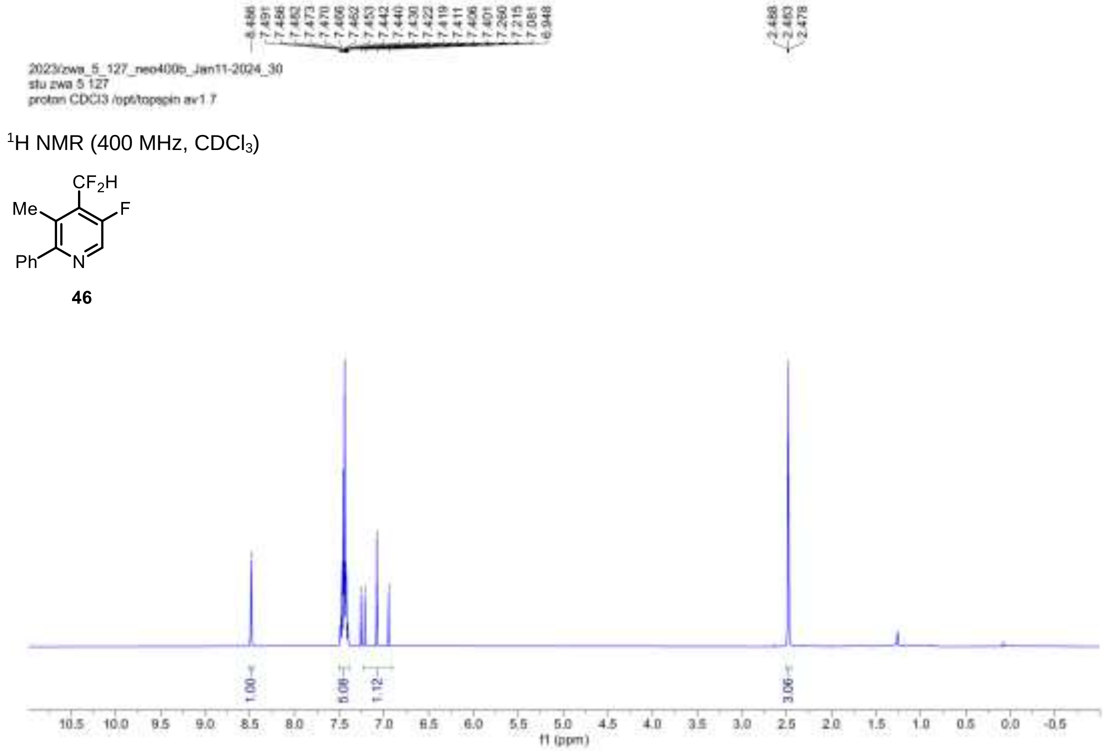

Supplementary Figure 174. ^{1}H NMR (400 MHz, $CDCl_3$) spectrum of compound **46**

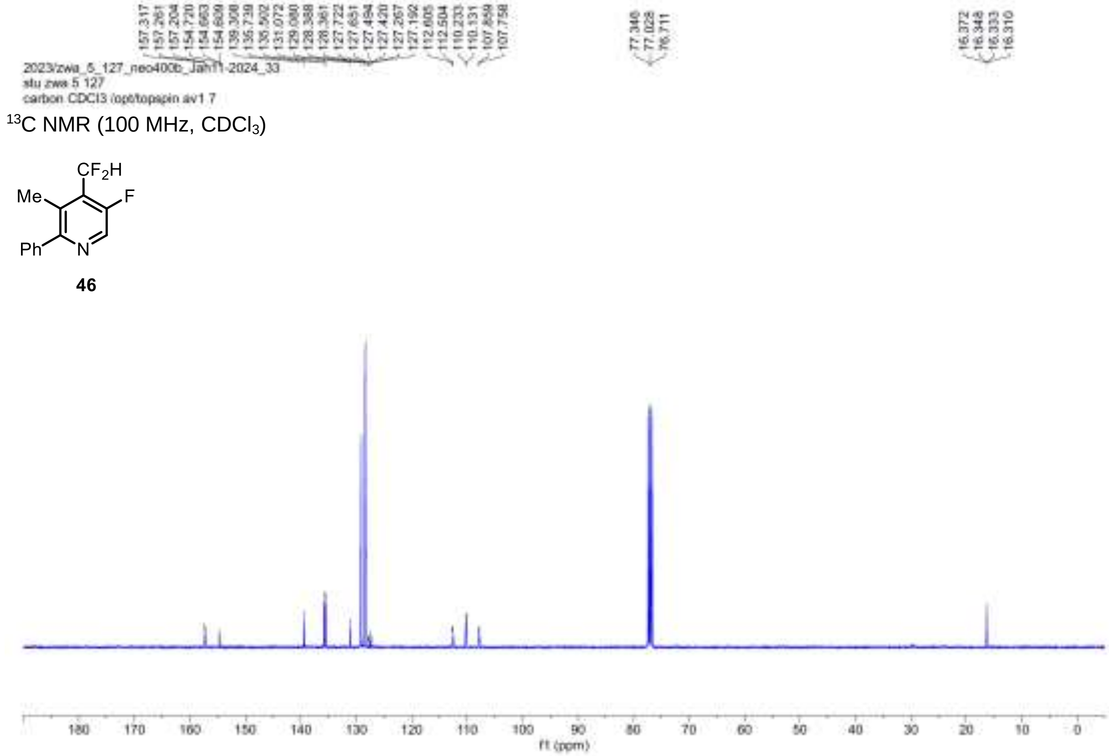

Supplementary Figure 175. ^{13}C NMR (100 MHz, $CDCl_3$) spectrum of compound **46**

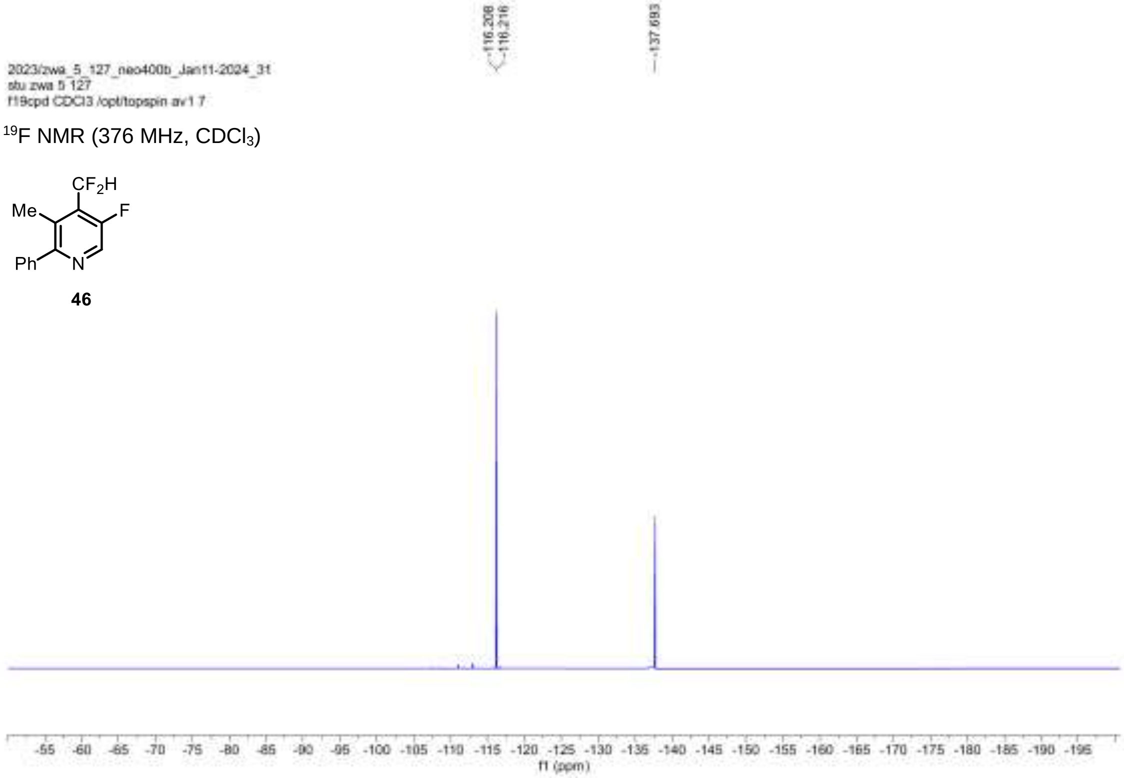

Supplementary Figure 176. ^{19}F NMR (376 MHz, $CDCl_3$) spectrum of compound **46**

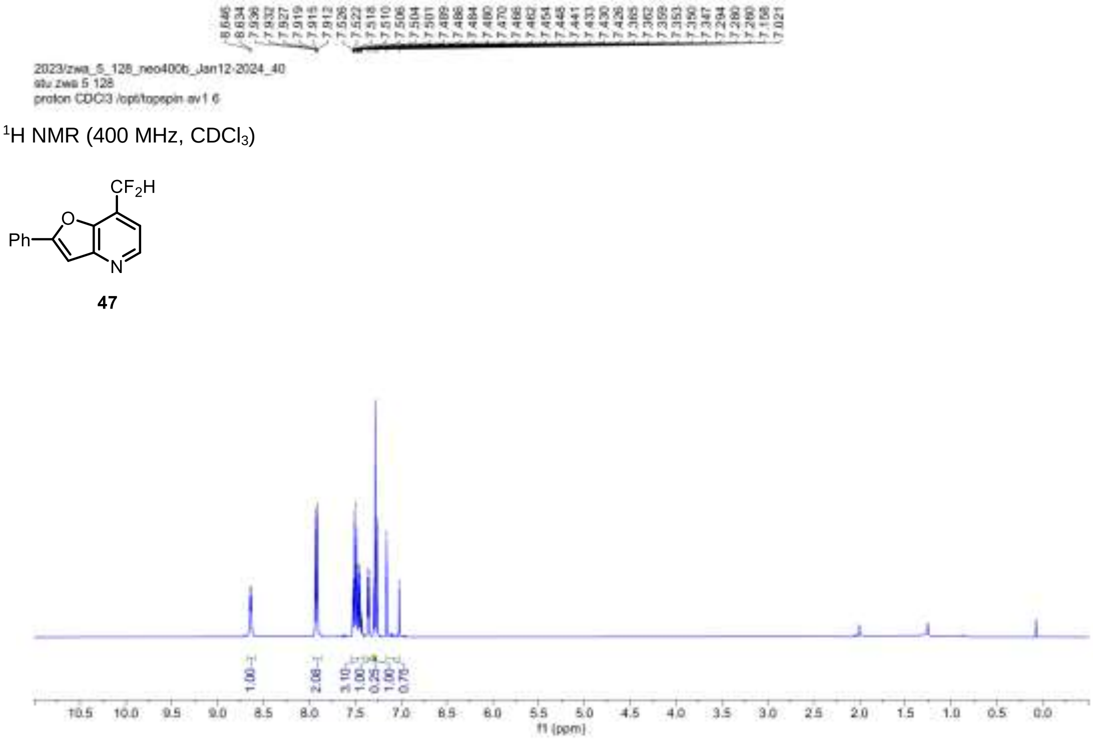

Supplementary Figure 177. ^{1}H NMR (400 MHz, $CDCl_3$) spectrum of compound **47**

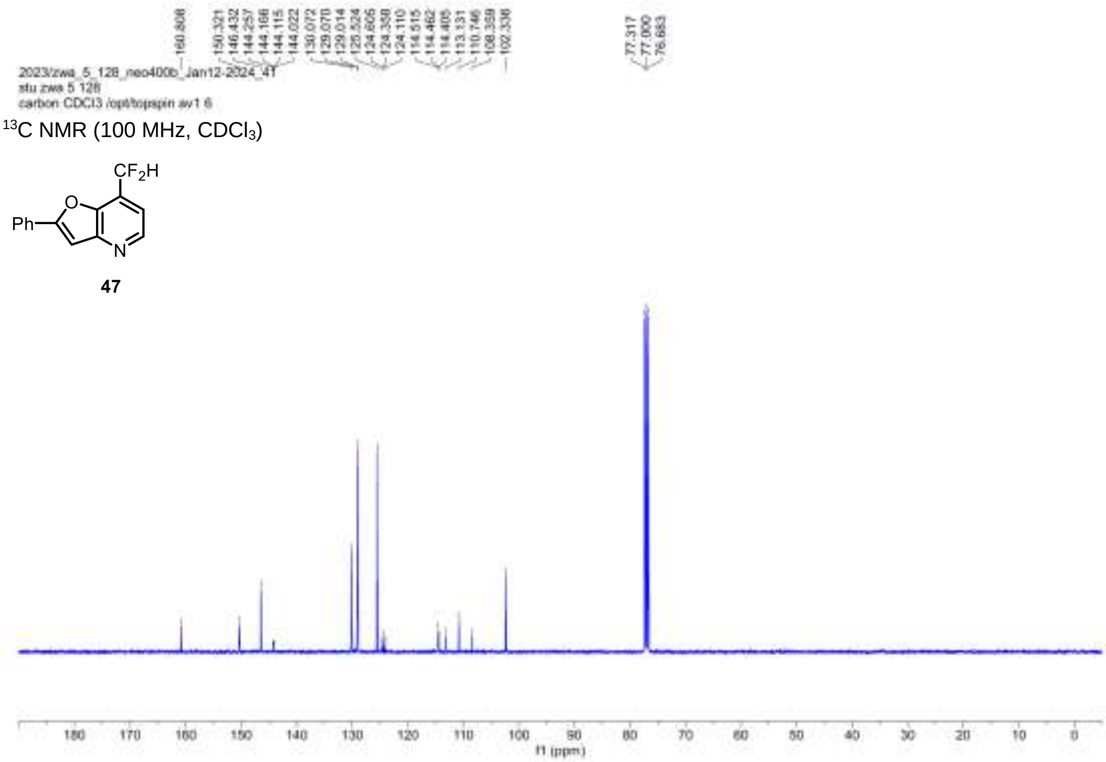

Supplementary Figure 178. ^{13}C NMR (100 MHz, $CDCl_3$) spectrum of compound **47**

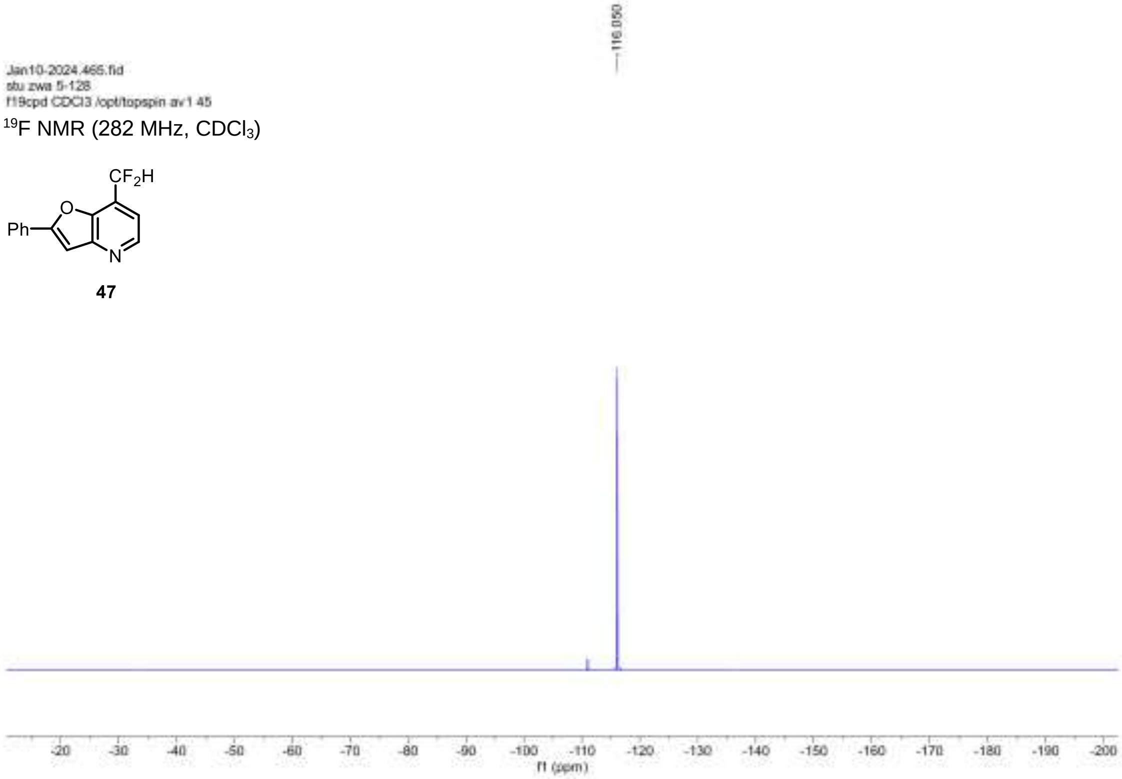

Supplementary Figure 179. ^{19}F NMR (376 MHz, $CDCl_3$) spectrum of compound **47**

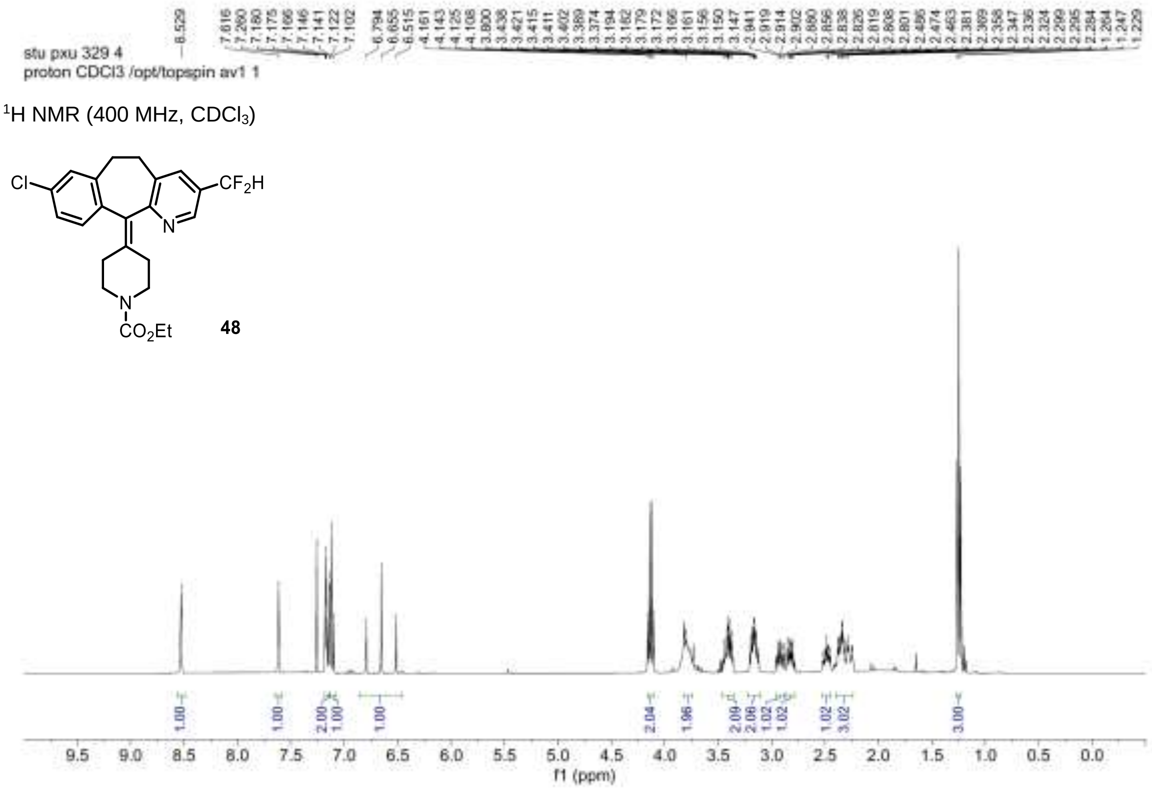

Supplementary Figure 180. ^{1}H NMR (400 MHz, $CDCl_3$) spectrum of compound **48**

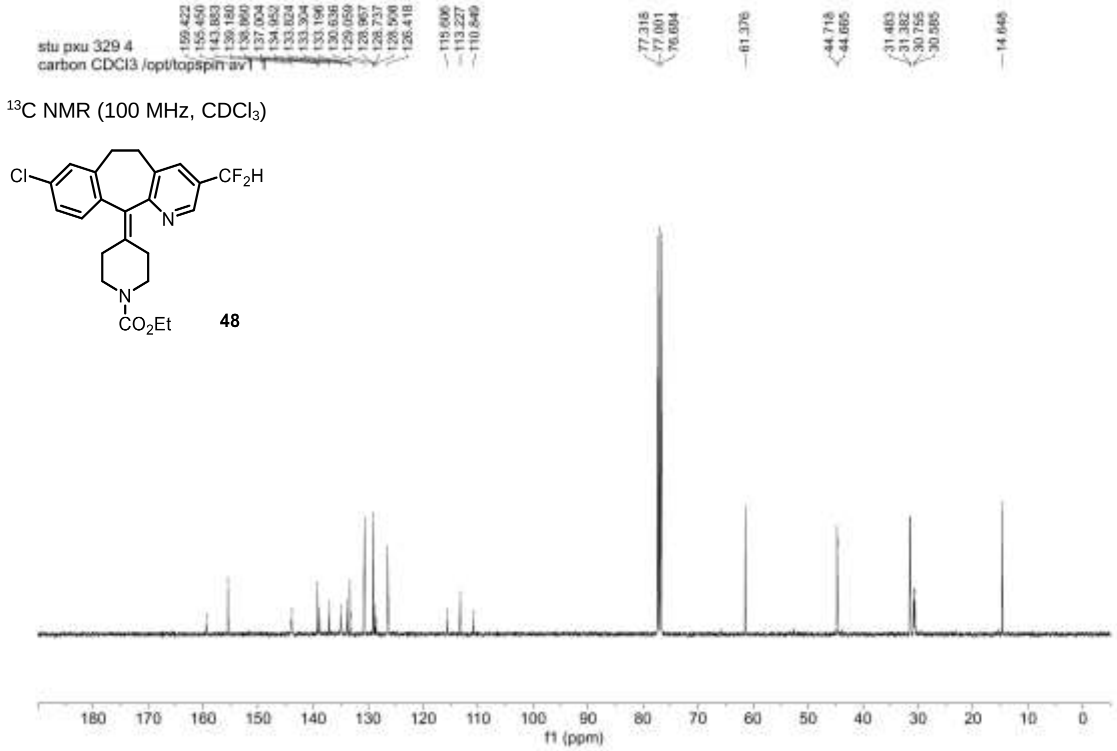

Supplementary Figure 181. ^{13}C NMR (100 MHz, $CDCl_3$) spectrum of compound **48**

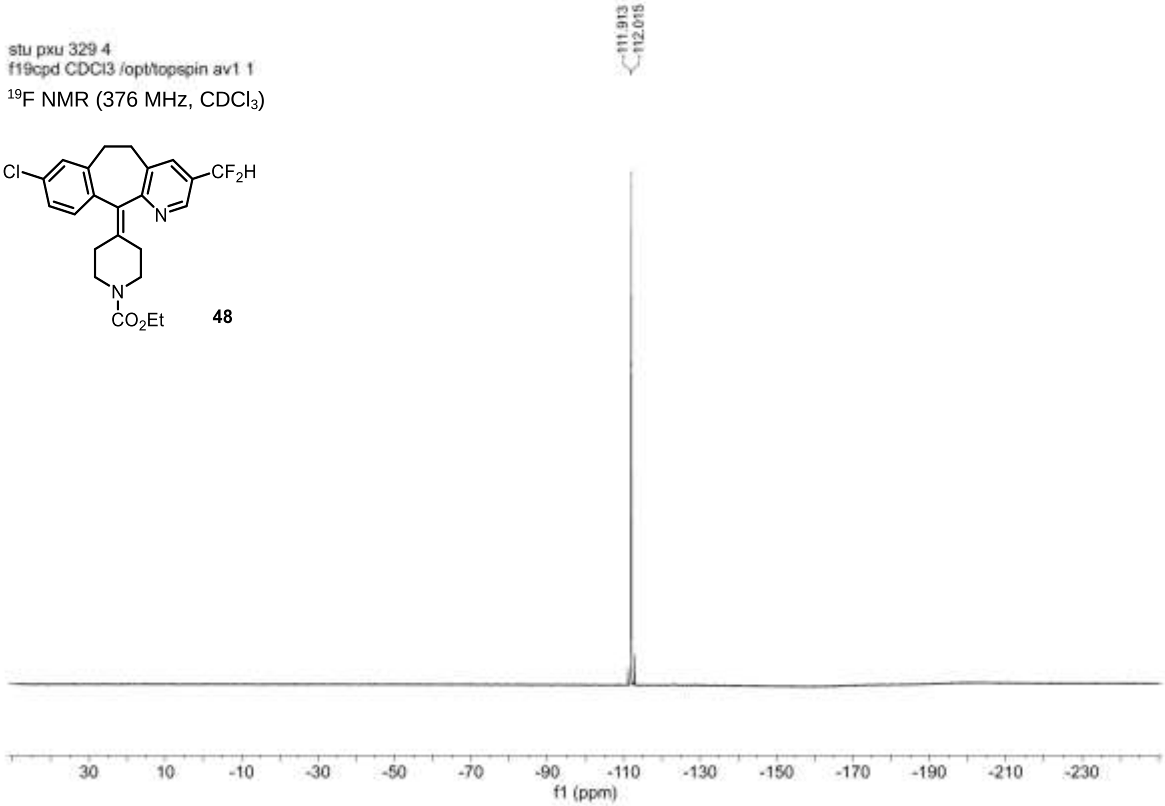

Supplementary Figure 182. ^{19}F NMR (376 MHz, $CDCl_3$) spectrum of compound **48**

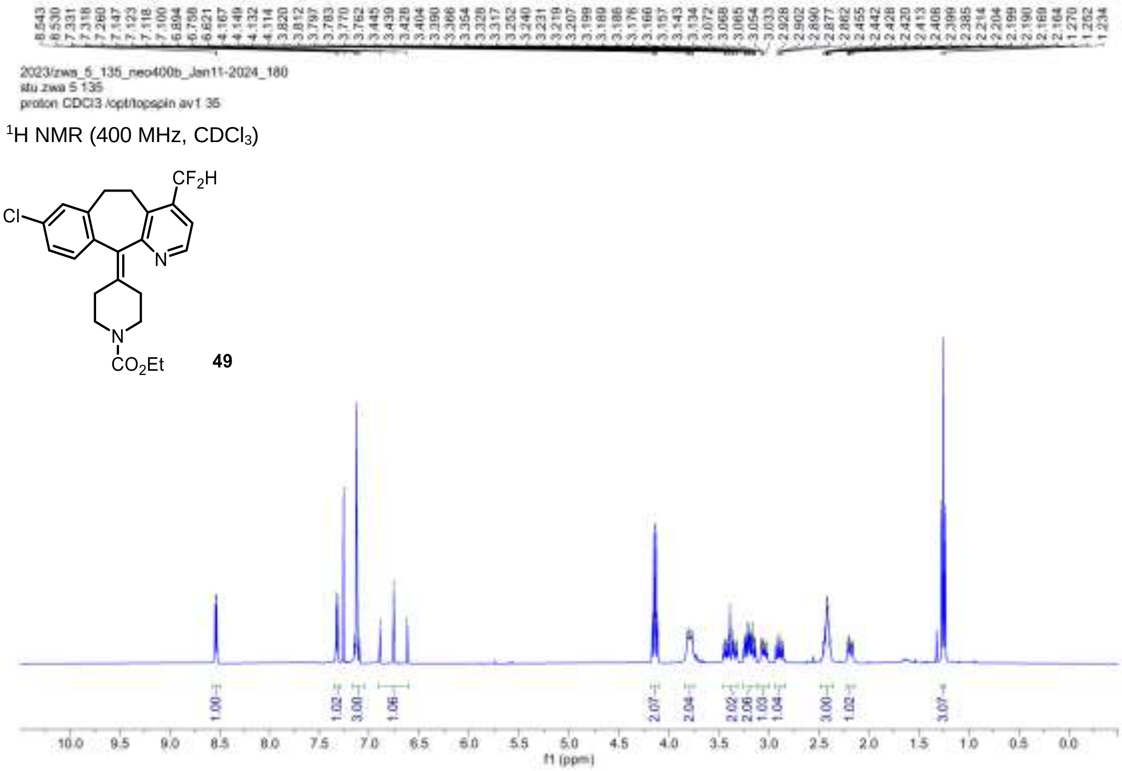

Supplementary Figure 183. ^{1}H NMR (400 MHz, $CDCl_3$) spectrum of compound **49**

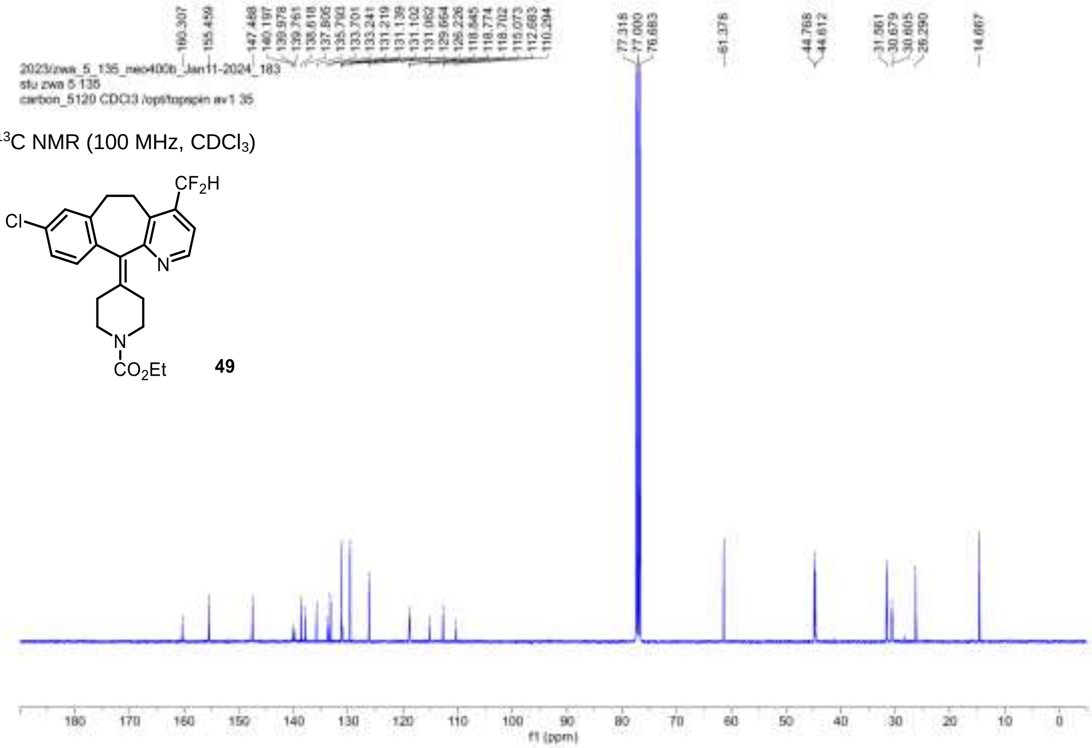

Supplementary Figure 184. ^{13}C NMR (100 MHz, $CDCl_3$) spectrum of compound **49**

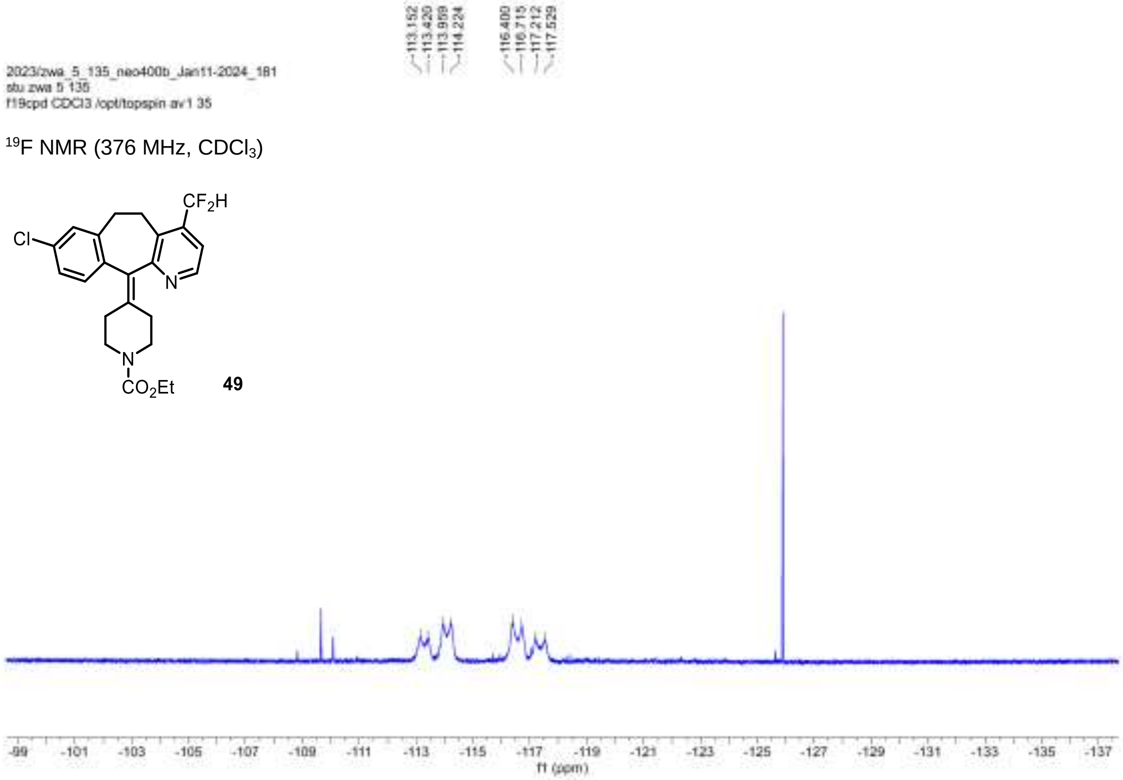

Supplementary Figure 185. ^{19}F NMR (376 MHz, $CDCl_3$) spectrum of compound **49**

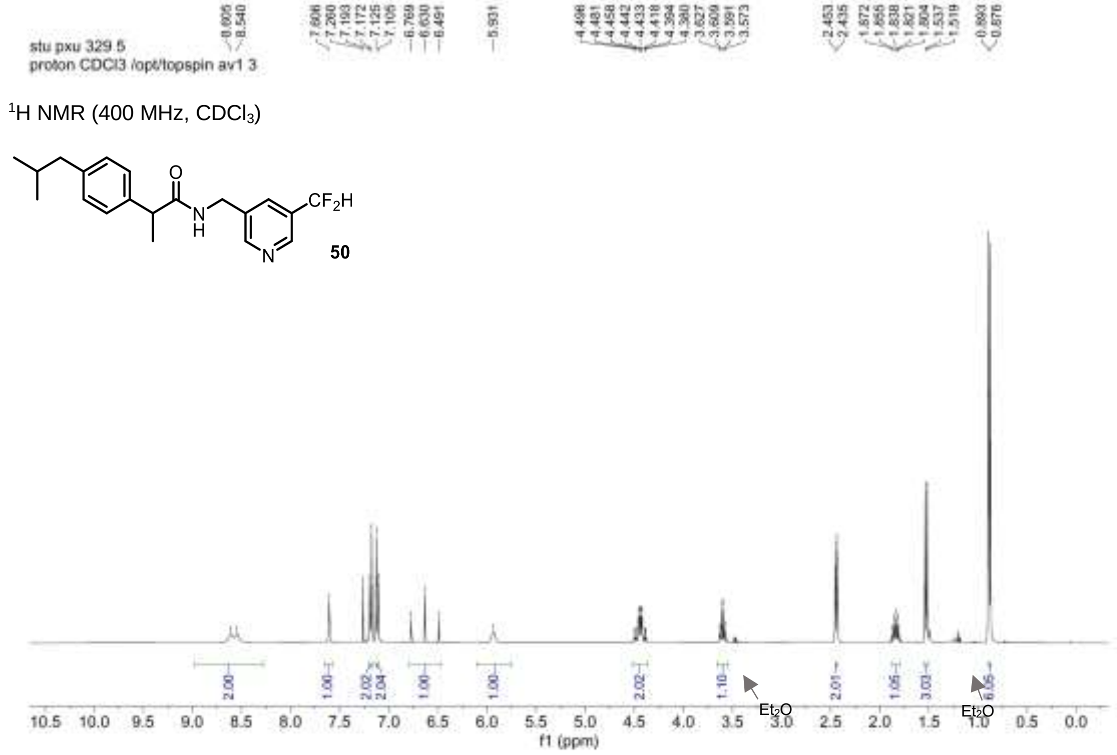

Supplementary Figure 186. ^{1}H NMR (400 MHz, $CDCl_3$) spectrum of compound **50**

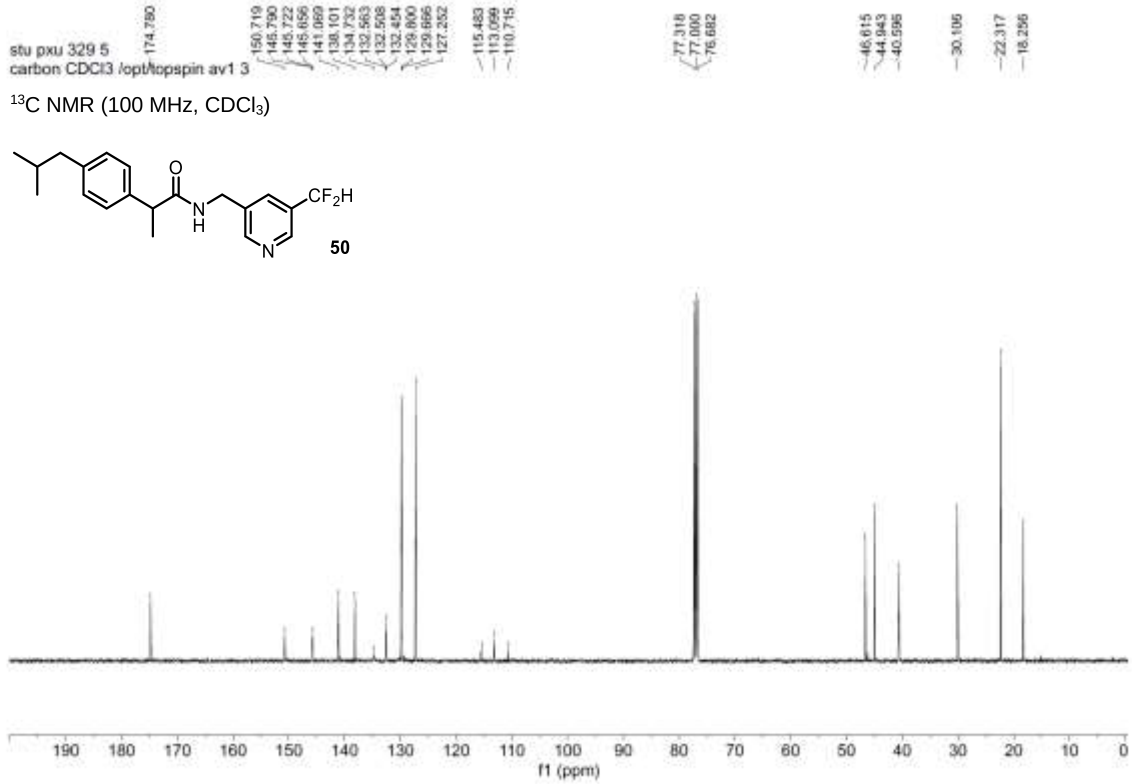

Supplementary Figure 187. ^{13}C NMR (100 MHz, $CDCl_3$) spectrum of compound **50**

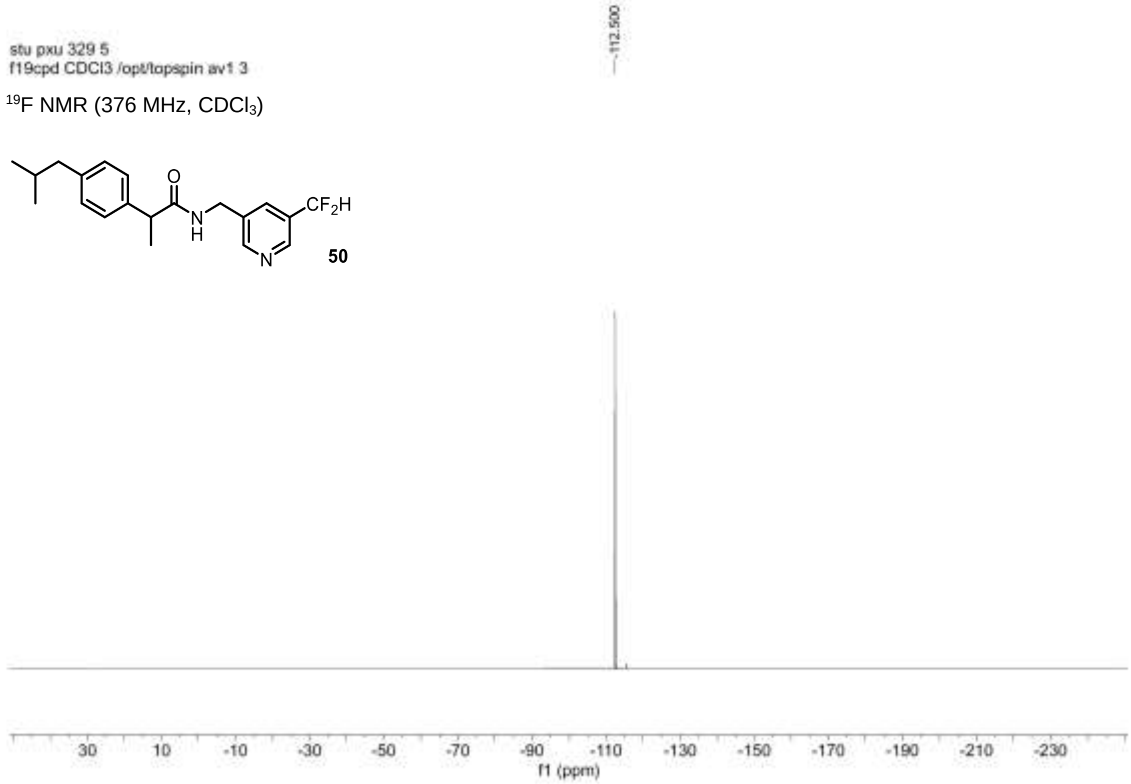

Supplementary Figure 188. ^{19}F NMR (376 MHz, $CDCl_3$) spectrum of compound **50**

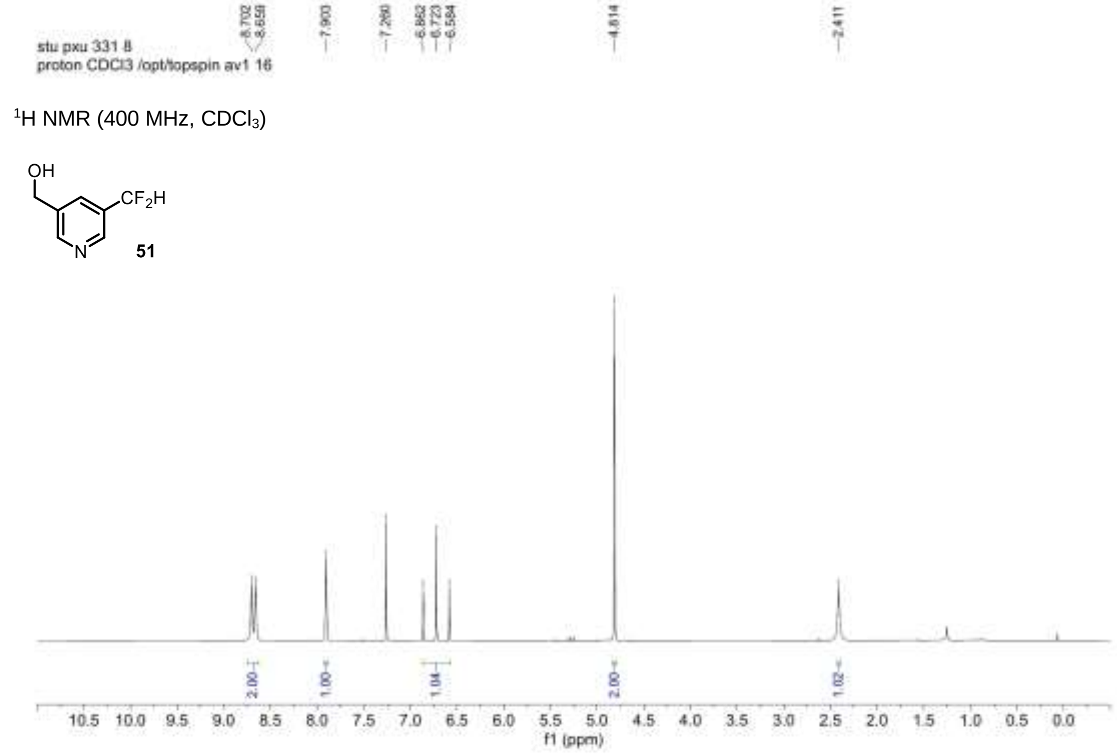

Supplementary Figure 189. ^{1}H NMR (400 MHz, $CDCl_3$) spectrum of compound **51**

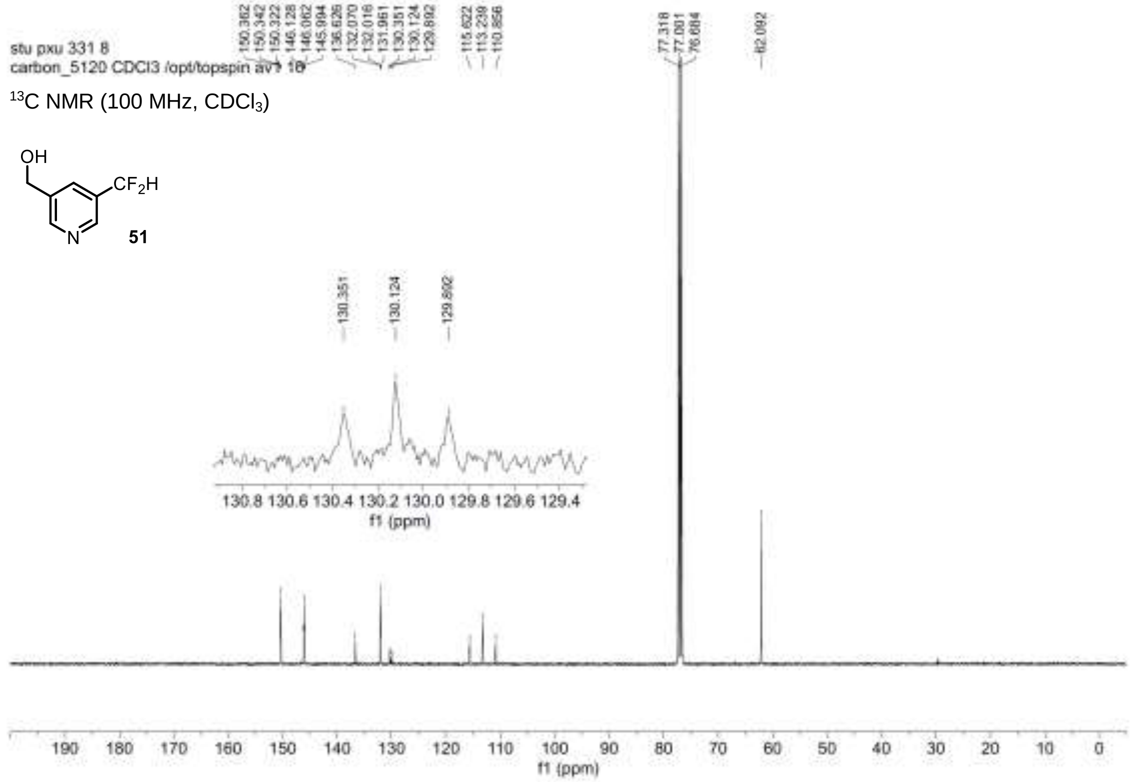

Supplementary Figure 190. ^{13}C NMR (100 MHz, $CDCl_3$) spectrum of compound **51**

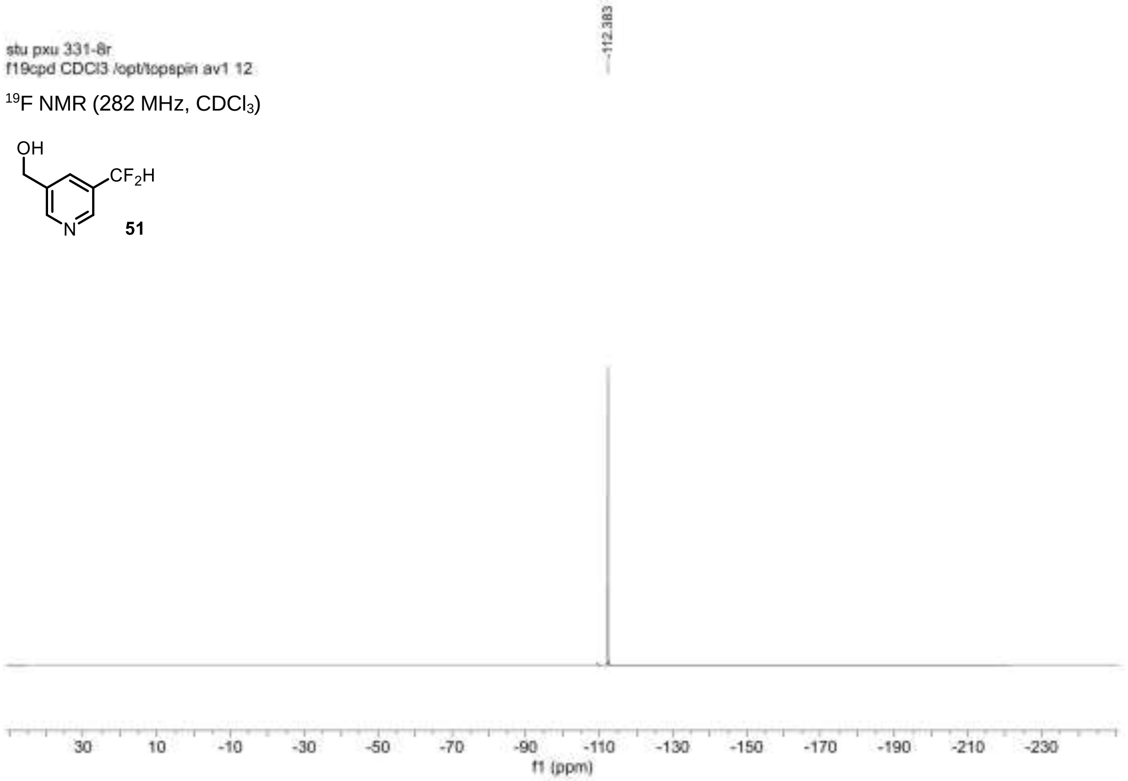

Supplementary Figure 191. ^{19}F NMR (282 MHz, $CDCl_3$) spectrum of compound **51**

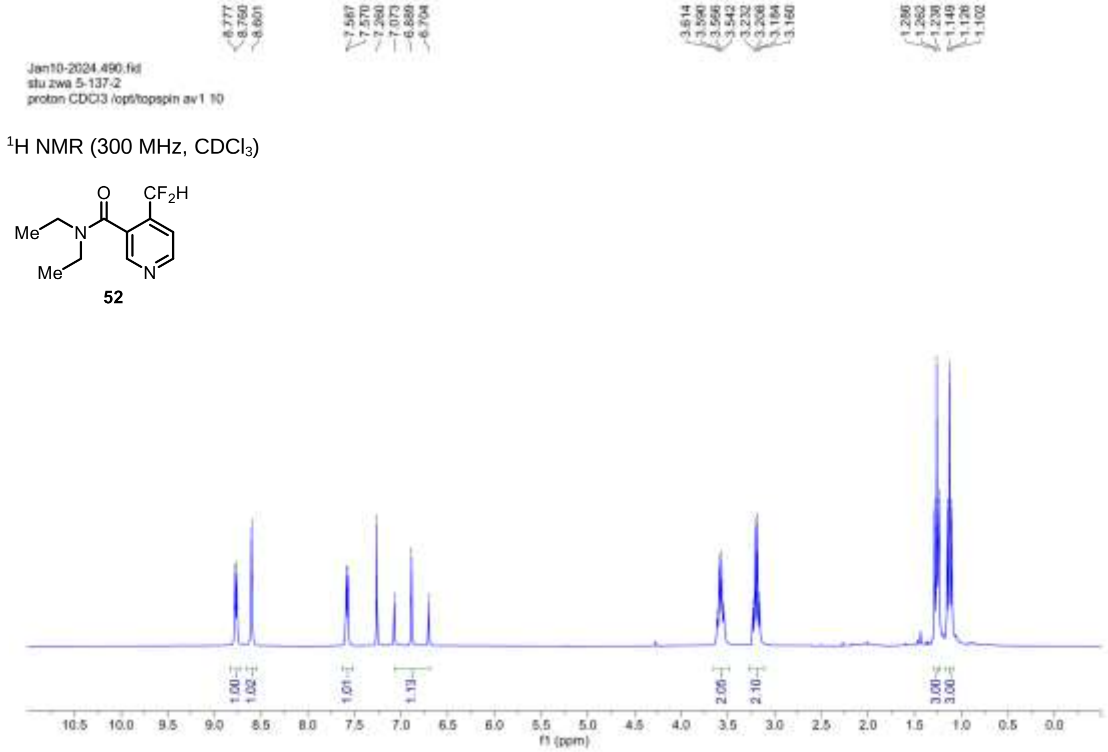

Supplementary Figure 192. ^{1}H NMR (300 MHz, $CDCl_3$) spectrum of compound **52**

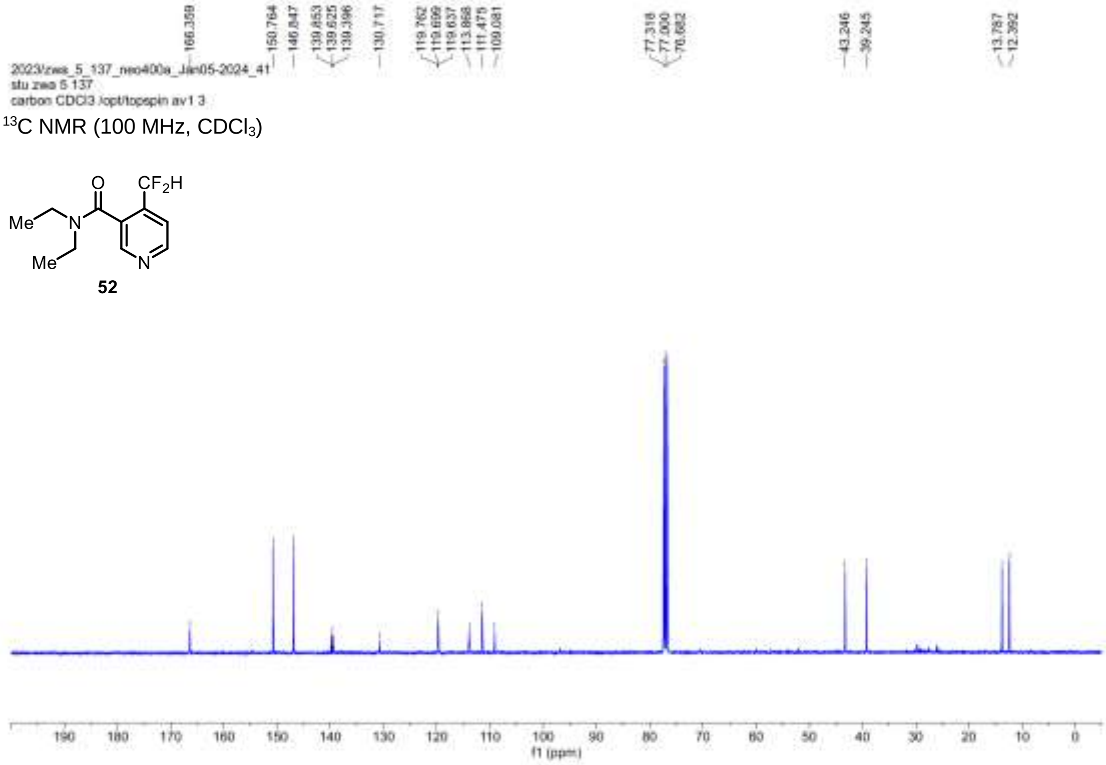

Supplementary Figure 193. ^{13}C NMR (100 MHz, $CDCl_3$) spectrum of compound **52**

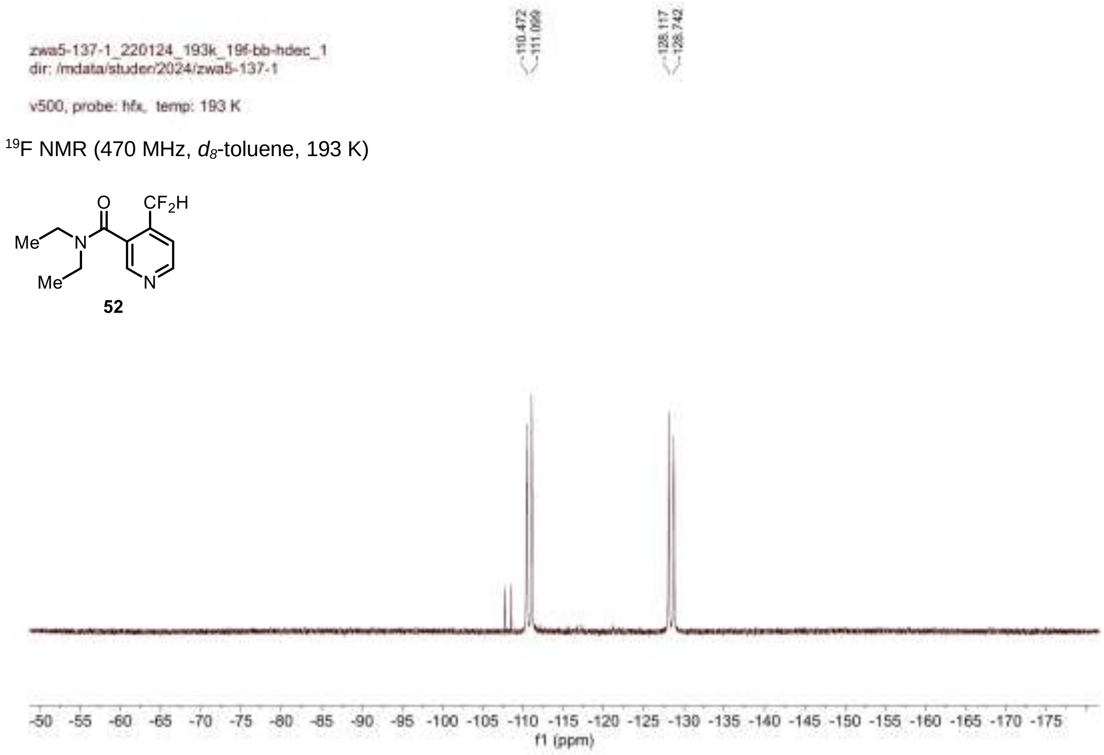

Supplementary Figure 194. ^{19}F NMR (470 MHz, d_8-toluene, 193 K) spectrum of compound **52**

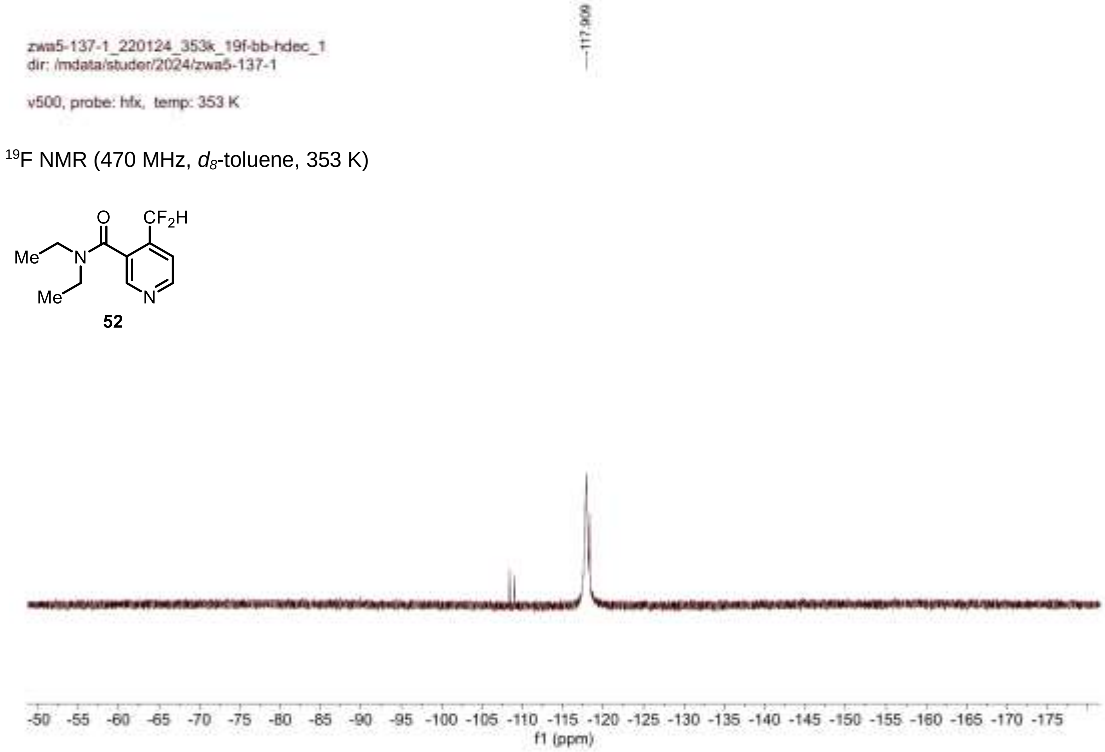

Supplementary Figure 195. ^{19}F NMR (470 MHz, d_8-toluene, 353 K) spectrum of compound **52**

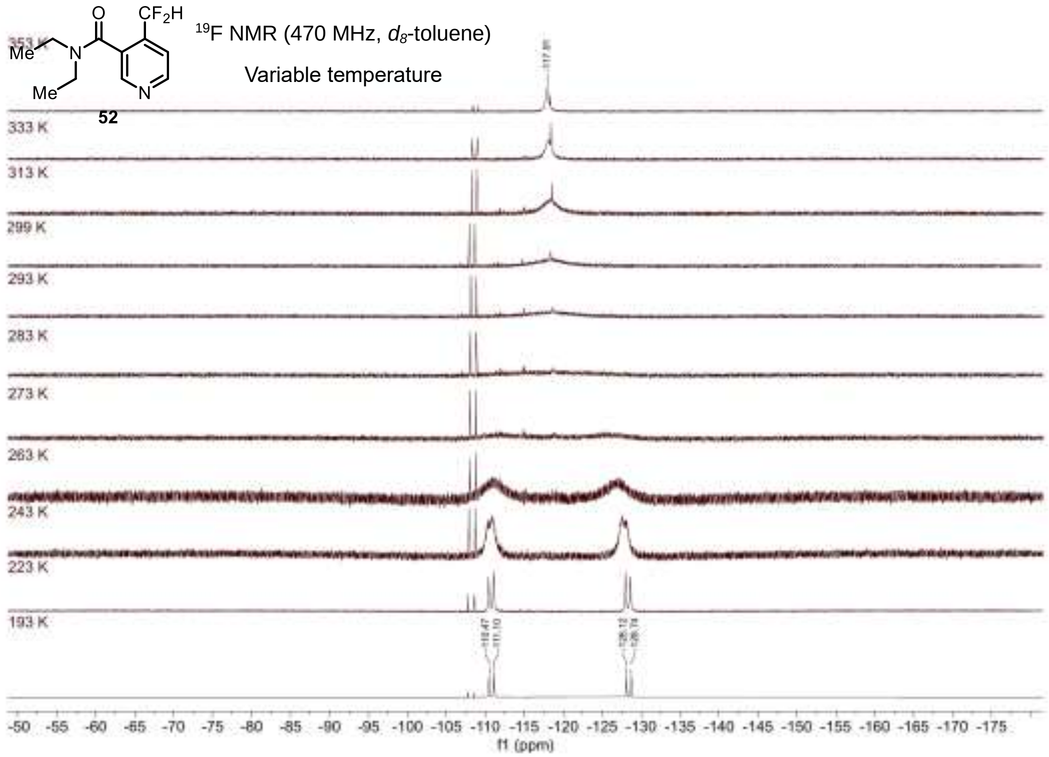

Supplementary Figure 196. ^{19}F NMR (470 MHz, d_8-toluene, 193 K) spectrum of compound **52**

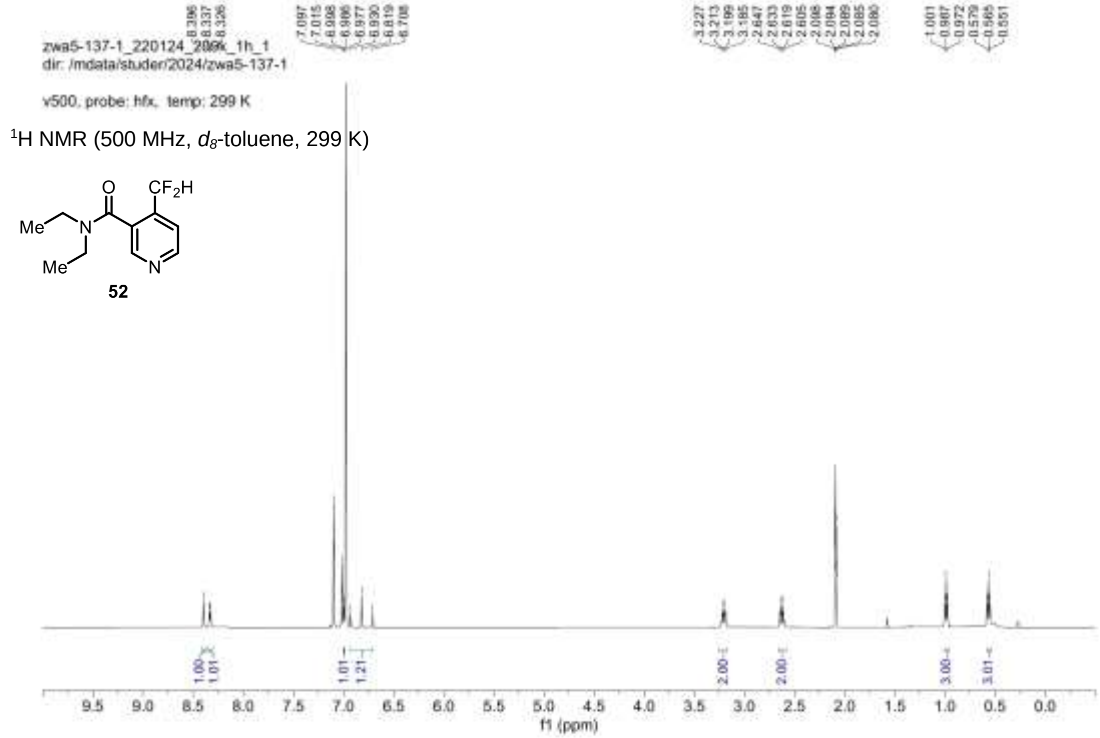

Supplementary Figure 197. ^{1}H NMR (500 MHz, d_8-toluene, 299 K) spectrum of compound **52**

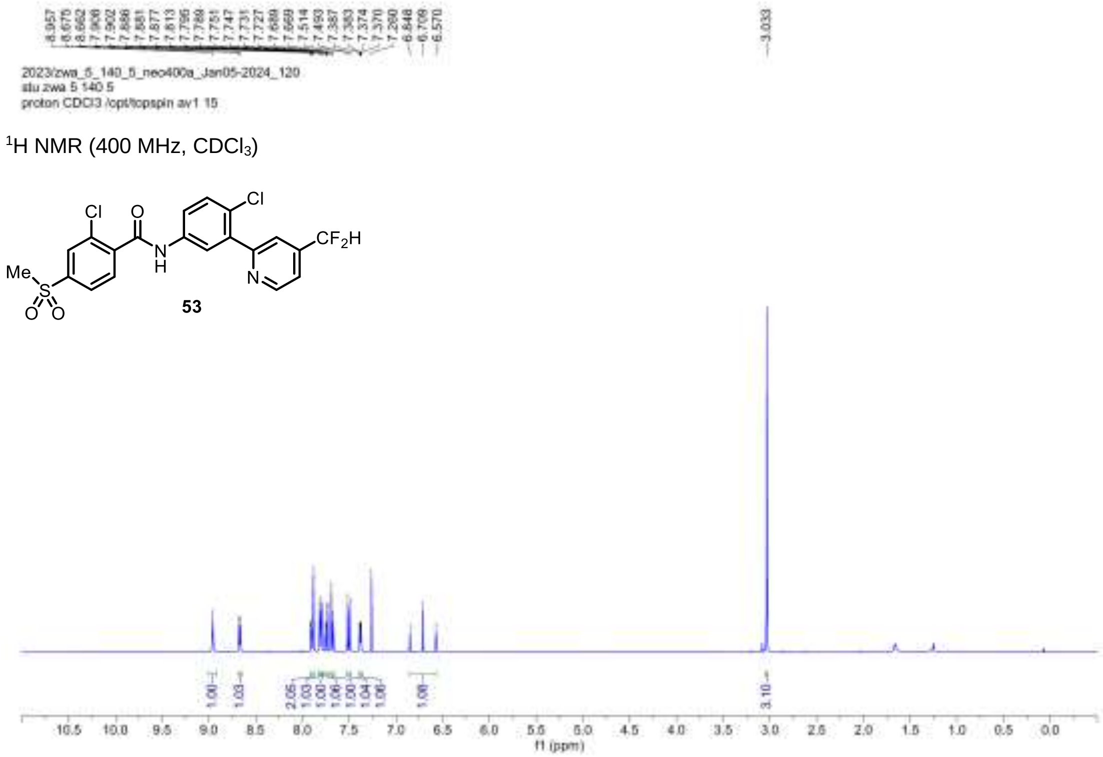

Supplementary Figure 198. ^{1}H NMR (400 MHz, $CDCl_3$) spectrum of compound **53**

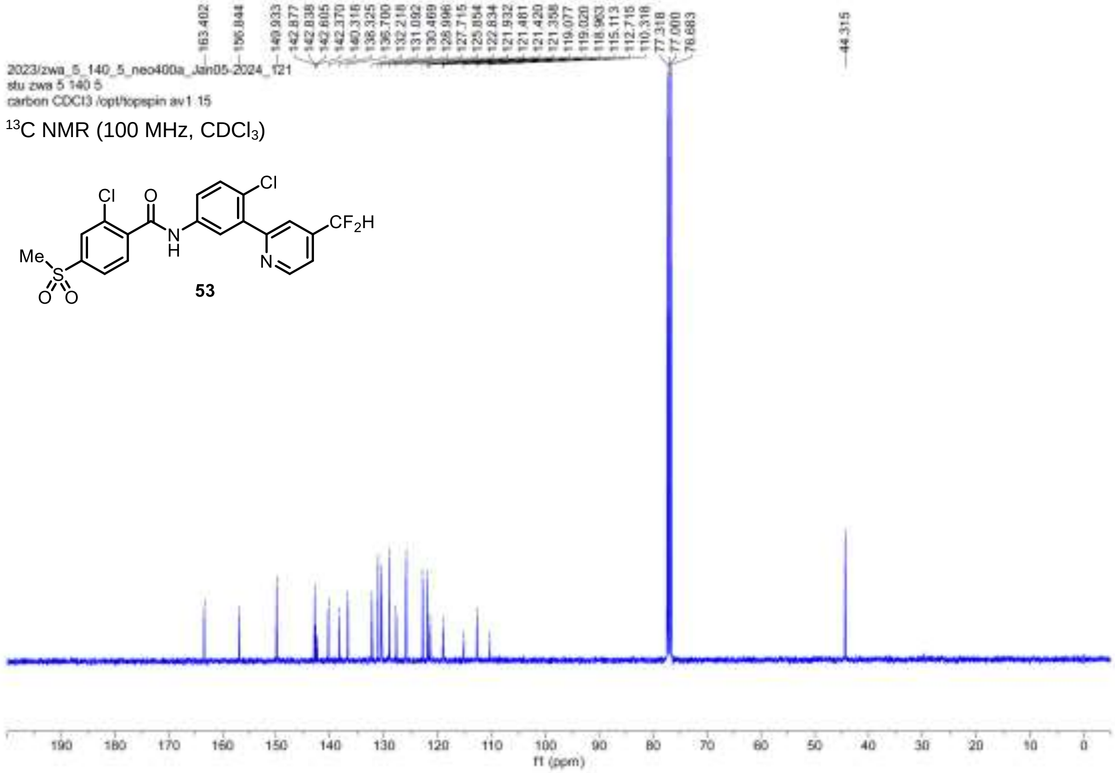

Supplementary Figure 199. ^{13}C NMR (100 MHz, $CDCl_3$) spectrum of compound **53**

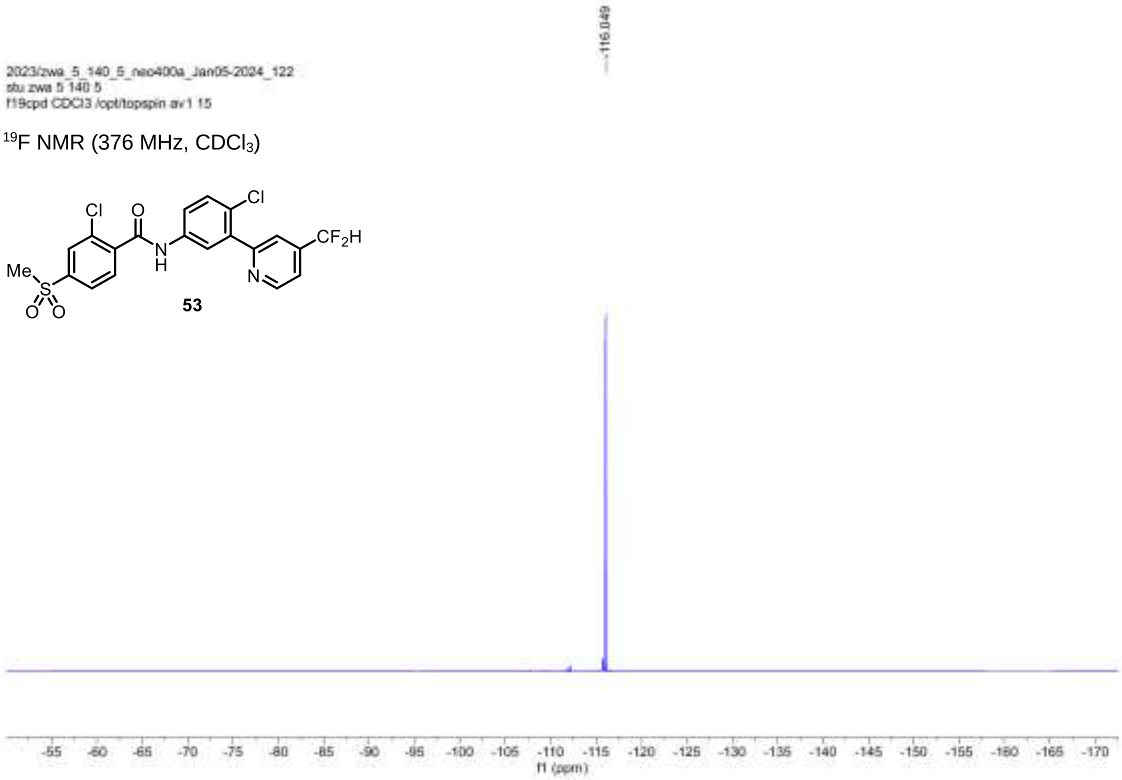

Supplementary Figure 200. ^{19}F NMR (376 MHz, $CDCl_3$) spectrum of compound **53**

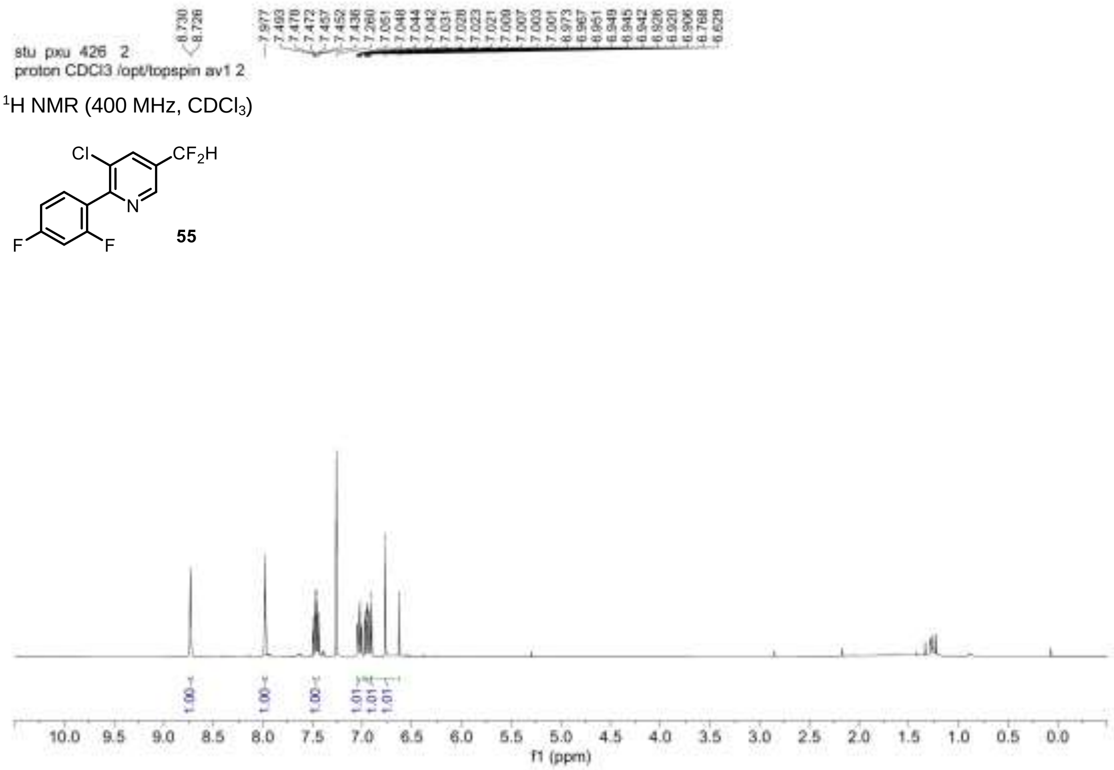

Supplementary Figure 201. ^{1}H NMR (400 MHz, $CDCl_3$) spectrum of compound **55**

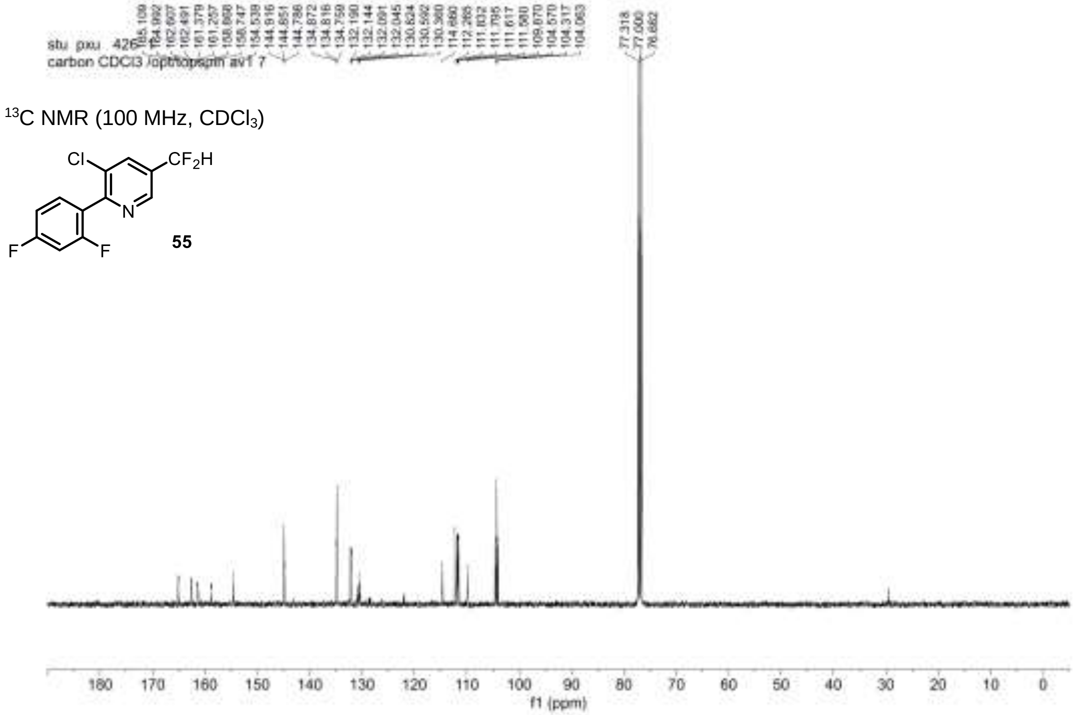

Supplementary Figure 202. ^{13}C NMR (100 MHz, $CDCl_3$) spectrum of compound **55**

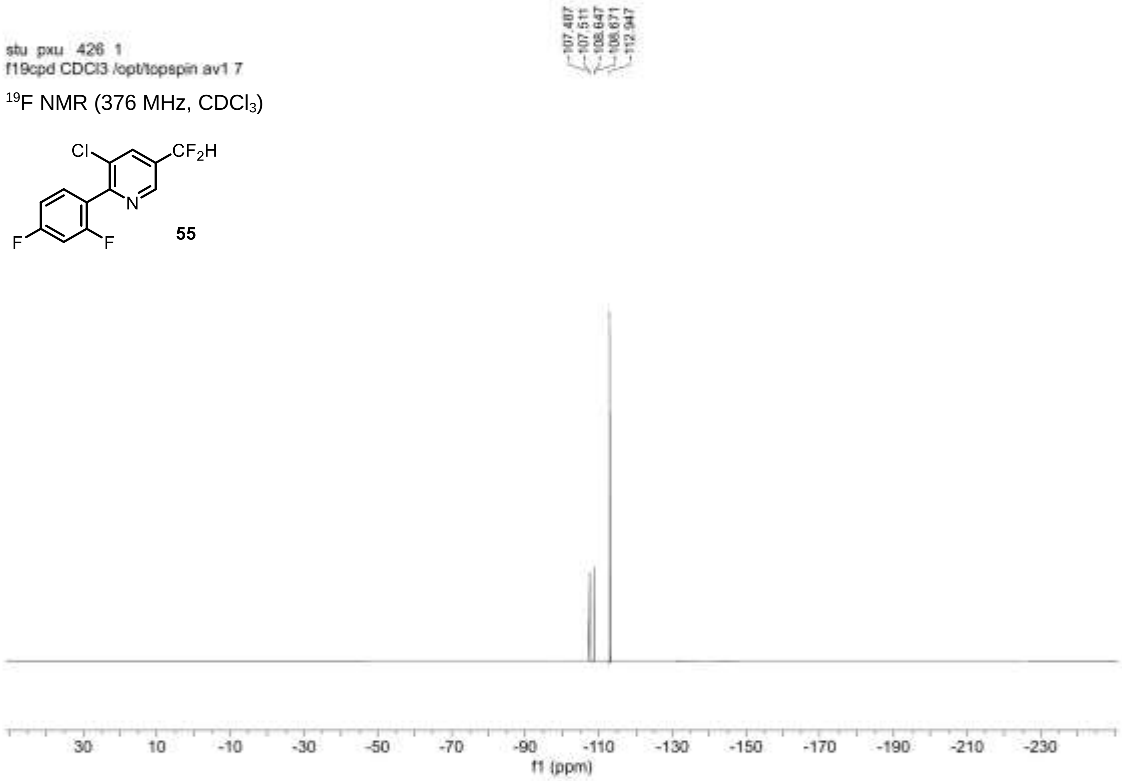

Supplementary Figure 203. ^{19}F NMR (376 MHz, $CDCl_3$) spectrum of compound **55**

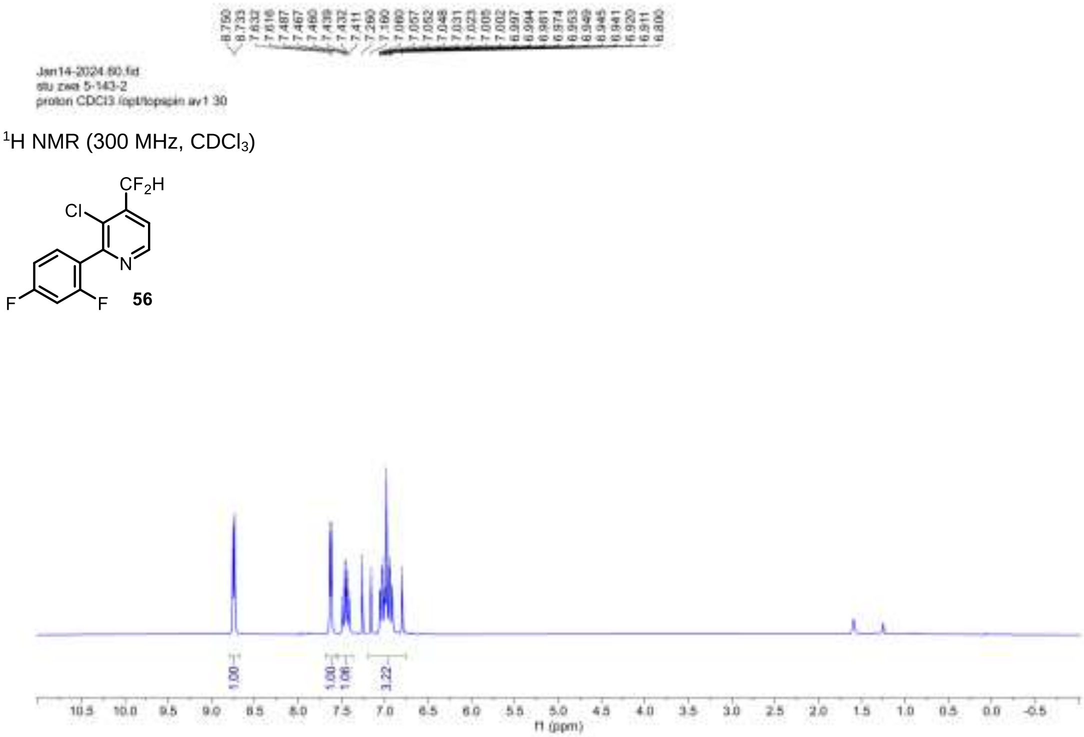

Supplementary Figure 204. ^{1}H NMR (300 MHz, $CDCl_3$) spectrum of compound **56**

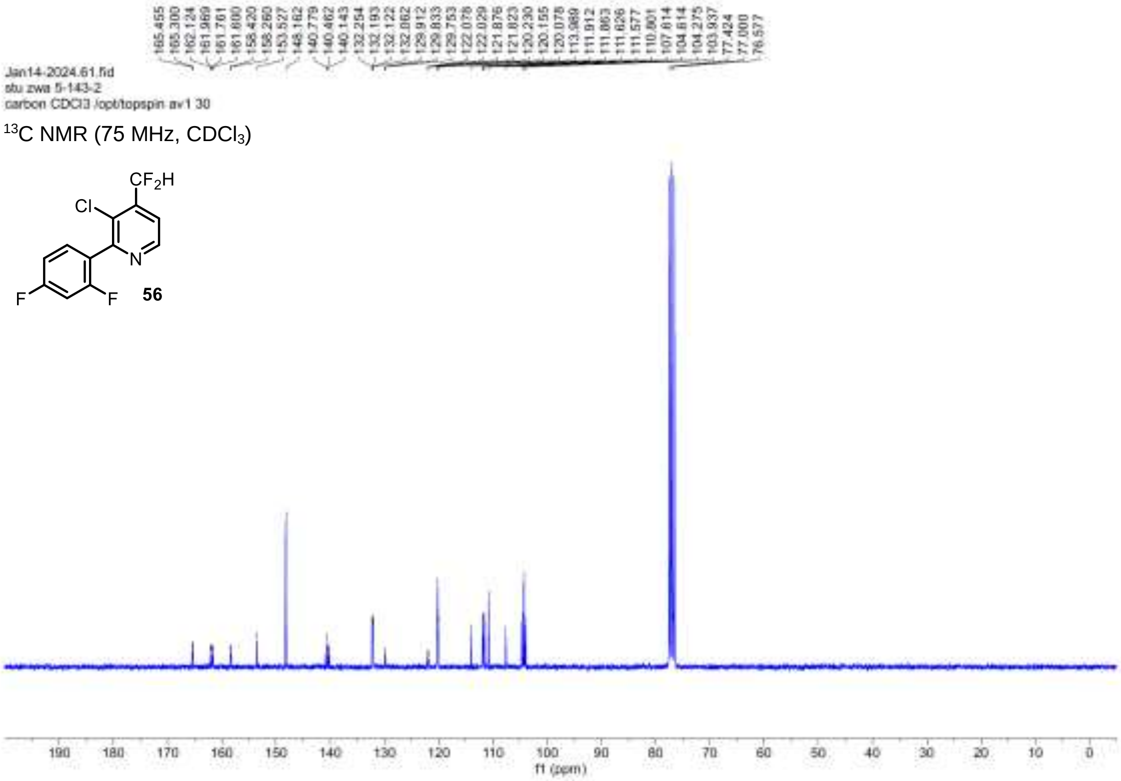

Supplementary Figure 205. ^{13}C NMR (75 MHz, $CDCl_3$) spectrum of compound **56**

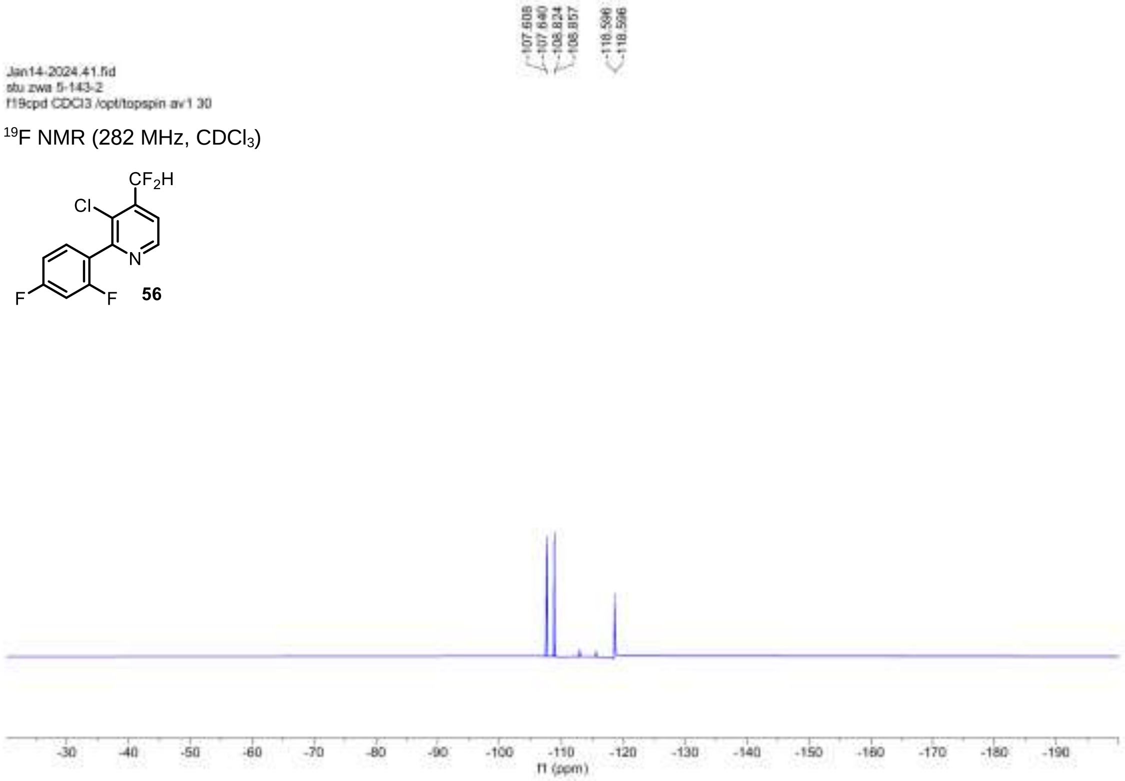

Supplementary Figure 206. ^{19}F NMR (282 MHz, $CDCl_3$) spectrum of compound **56**

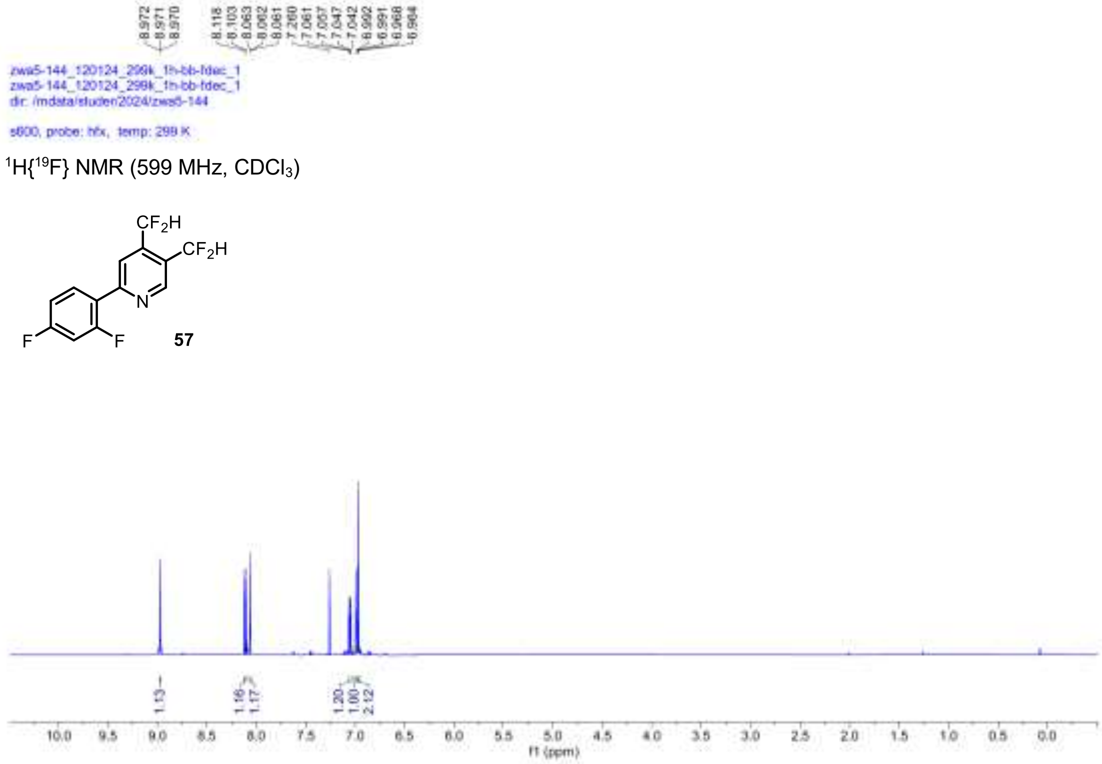

Supplementary Figure 207. ^{1}H{^{19}F} NMR (599 MHz, $CDCl_3$) spectrum of compound **57**

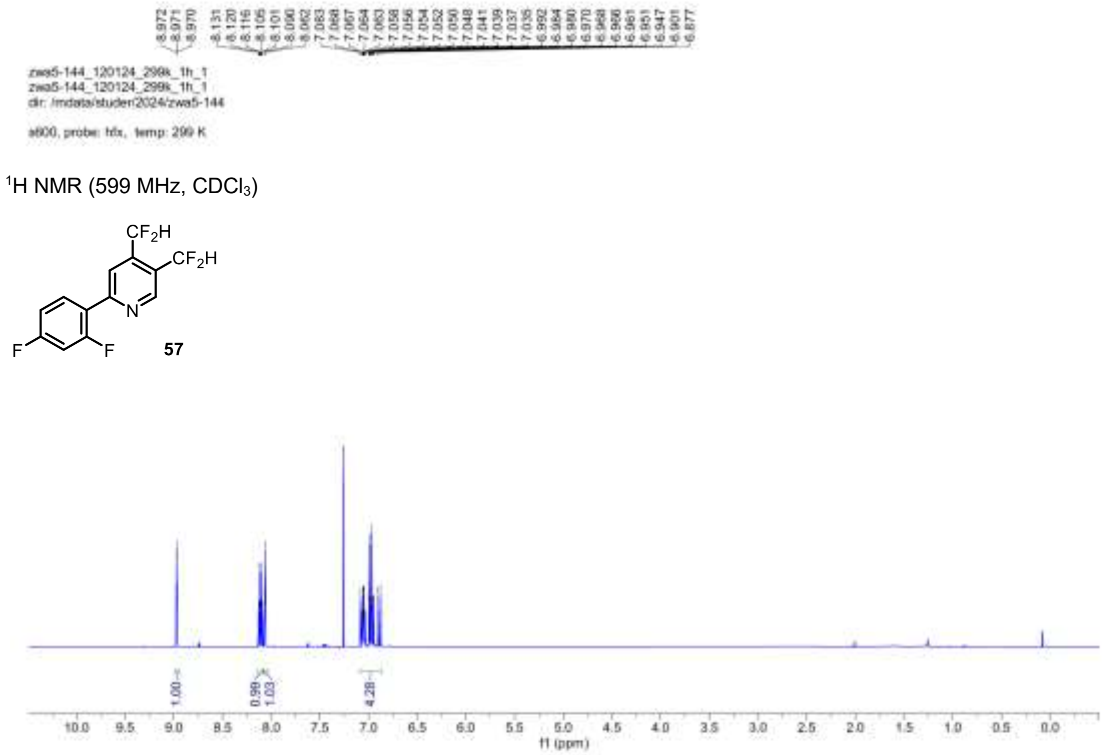

Supplementary Figure 208. ^{1}H NMR (599 MHz, $CDCl_3$) spectrum of compound **57**

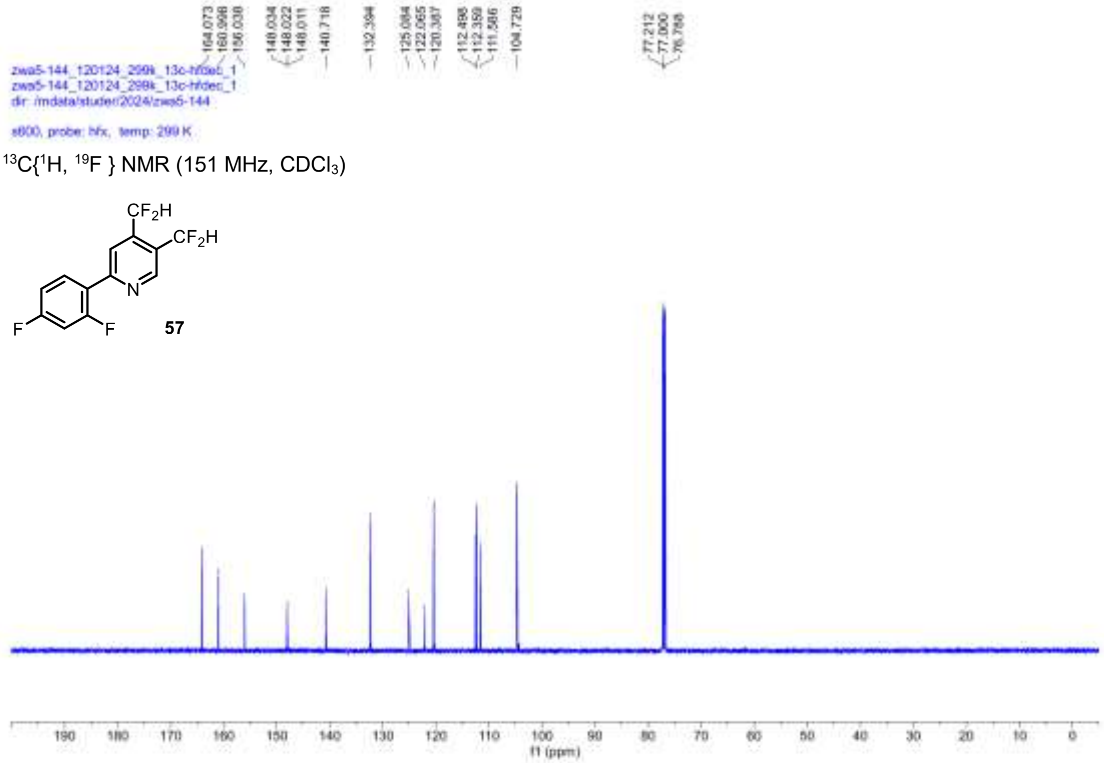

Supplementary Figure 209. $^{13}C\{^{1}H,^{19}F\}$ NMR (151 MHz, $CDCl_3$) spectrum of compound **57**

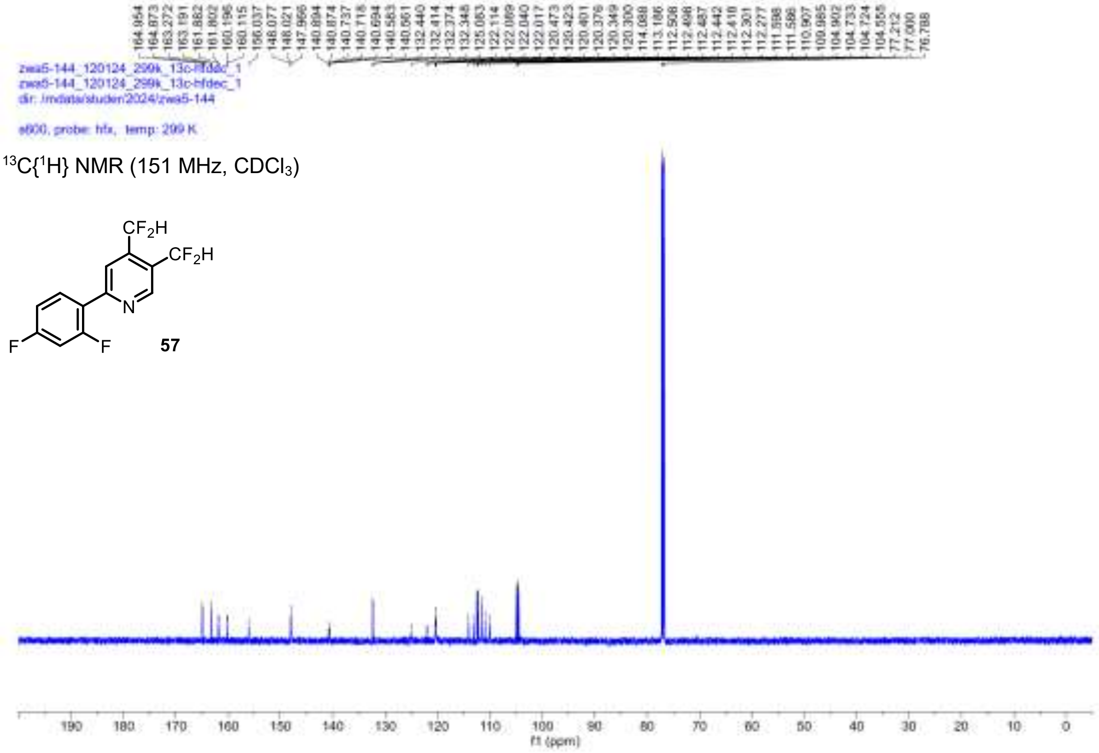

Supplementary Figure 210. $^{13}C\{^{1}H\}$ NMR (151 MHz, $CDCl_3$) spectrum of compound **57**

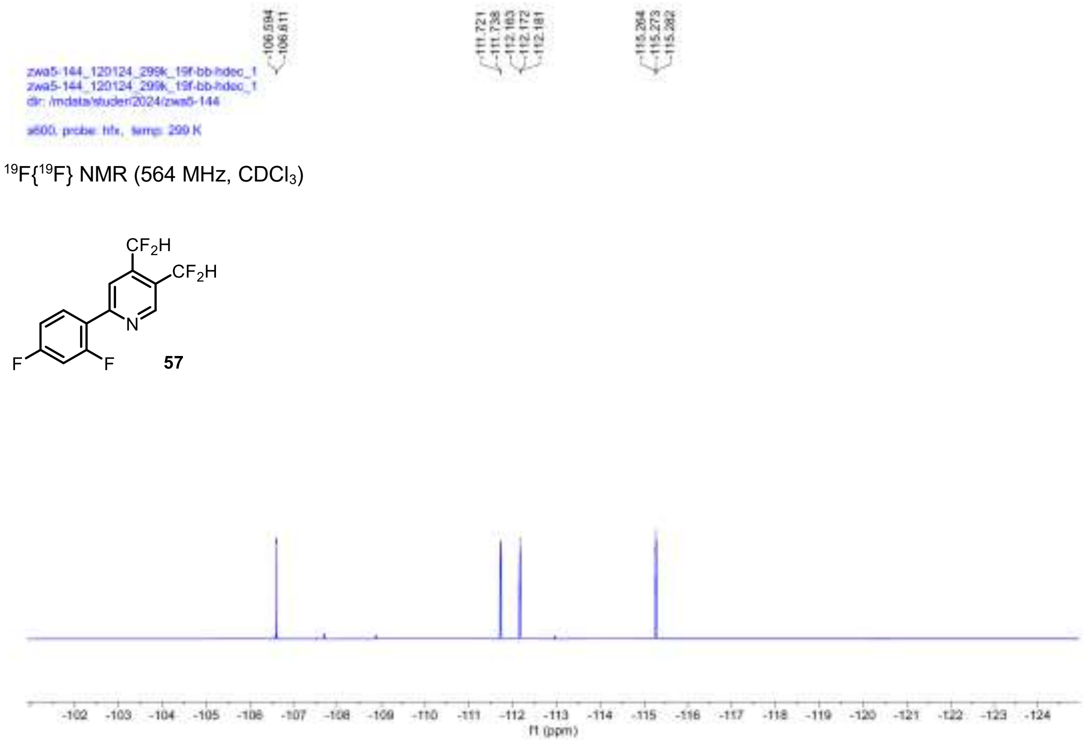

Supplementary Figure 211. $^{19}F\{^{19}F\}$ NMR (564 MHz, $CDCl_3$) spectrum of compound **57**

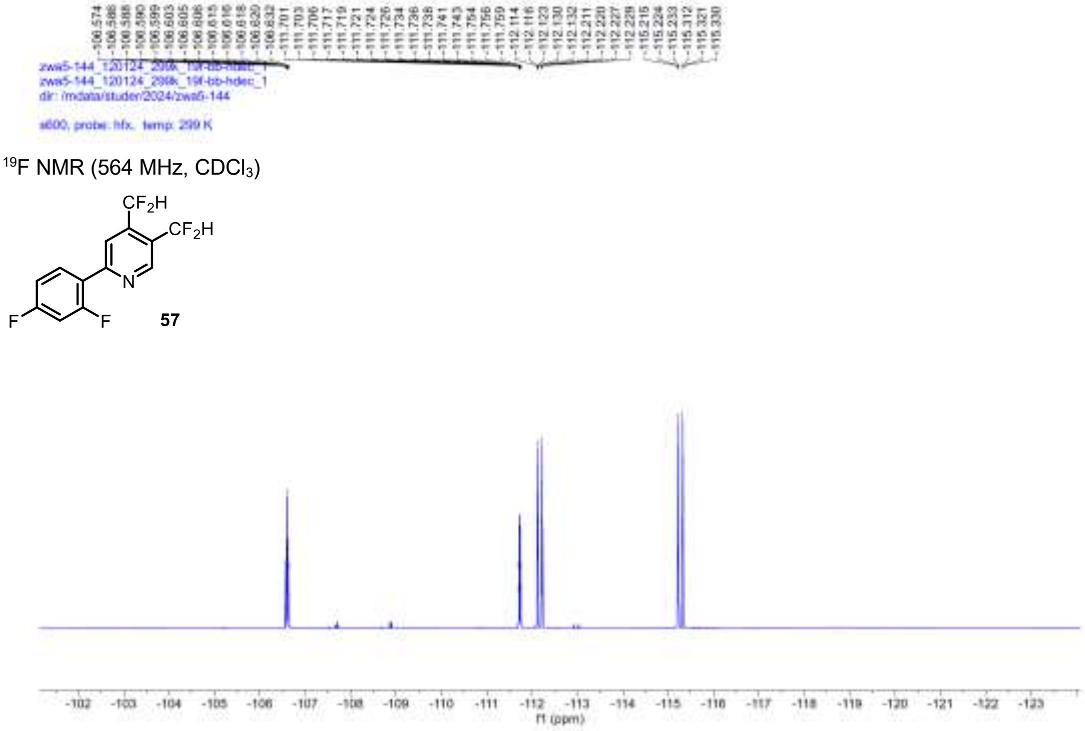

Supplementary Figure 212. ^{19}F NMR (564 MHz, $CDCl_3$) spectrum of compound **57**

Section 11. Supplementary References

1 Han, C., Kim, E. H. & Colby, D. A. Cleavage of Carbon−Carbon Bonds through the Mild Release of Trifluoroacetate: Generation of α,α-Difluoroenolates for Aldol Reactions. *J. Am. Chem. Soc.* **133**, 5802-5805 (2011).

2 John, J. P. & Colby, D. A. Synthesis of α-Halo-α,α-difluoromethyl Ketones by a Trifluoroacetate Release/Halogenation Protocol. *J. Org. Chem.* **76**, 9163-9168 (2011).

3 Qiu, Z.-M. & Burton, D. J. Synthesis of .alpha.,.alpha.-Difluoro-Functionalized Ketones. *J. Org. Chem.* **60**, 5570-5578 (1995).

4 Cao, H., Cheng, Q. & Studer, A. Radical and ionic meta-C-H functionalization of pyridines, quinolines, and isoquinolines. *Science* **378**, 779-785 (2022).

5 Cao, H., Bhattacharya, D., Cheng, Q. & Studer, A. C–H Functionalization of Pyridines via Oxazino Pyridine Intermediates: Switching to para-Selectivity under Acidic Conditions. *J. Am. Chem. Soc.* **145**, 15581-15588 (2023).

6 Ge, S., Chaladaj, W. & Hartwig, J. F. Pd-catalyzed alpha-arylation of alpha,alpha-difluoroketones with aryl bromides and chlorides. A route to difluoromethylarenes. *J. Am. Chem. Soc.* **136**, 4149-4152 (2014).

7 Zou, Z. *et al.* Electrochemical-Promoted Nickel-Catalyzed Oxidative Fluoroalkylation of Aryl Iodides. *Org. Lett.* **23**, 8252-8256 (2021).

8 Nottingham, K., Patel, C., Levy, J., Mcnally, A. & Zhang, X. Phosphine reagents for azine fluoroalkylation. (2022).

9 Bacauanu, V. *et al.* Metallaphotoredox Difluoromethylation of Aryl Bromides. *Angew. Chem. Int. Ed.* **57**, 12543-12548 (2018).

10 Zhang, X. *et al.* Phosphorus-mediated sp(2)-sp(3) couplings for C-H fluoroalkylation of azines. *Nature* **594**, 217-222 (2021).
